# Effect of Salt Additives on Dinitrogen Activation Mediated by Boron-Based Compounds: Insights from Theory

**DOI:** 10.1021/acs.inorgchem.5c05891

**Published:** 2026-05-20

**Authors:** Shailja Jain, Johannes Kästner

**Affiliations:** † Institute for Theoretical Chemistry, University of Stuttgart, Stuttgart 70569, Germany; ‡ Department of Chemistry, School of Advanced Sciences, 684926 VIT-AP University, Amaravati 522241, Andhra Pradesh, India

## Abstract

Dinitrogen (N_2_) is an inert molecule, and its activation under metal-free conditions presents a longstanding challenge with potential for new pathways to transform N_2_ into valuable products. Herein, we employ density functional theory (DFT) to investigate the reactivity and reaction mechanism of two boron-based compounds, cyclic (alkyl)­(amino)­carbene (CAAC), diboracumulene (**1**), and *N*-heterocyclic carbene (NHC)-diboryne (**2**) toward N_2_. Interestingly, we demonstrated that noncovalently bound lithium chloride (LiCl) enhances the reactivity by acting as a promoter, creating a local electric field that facilitates N_2_ binding and activation. While salts like LiCl are often used empirically in bond activation, their role as a local electric field source for N_2_ activation is rarely explored. Our calculations reveal that the free energy barrier for N_2_ coordination and activation is significantly reduced with the **1**/LiCl combination, and a process that is endergonic without LiCl becomes exergonic in its presence. Furthermore, the effect of heavier alkali metal salts, such as KCl, and bulkier ion-pairs containing 1-butyl-1-methylpyrrolidinium cation, (I) [C_4_mpyr]^+^[eFAP]^−^, and (II) [C_4_mpyr]^+^[PF_6_]^−^ on N_2_ coordination was explored to gain comparative insights into how variations in electrostatic environments influence N_2_ binding to **1**, relative to LiCl, as presented in the later sections.

## Graphical abstract

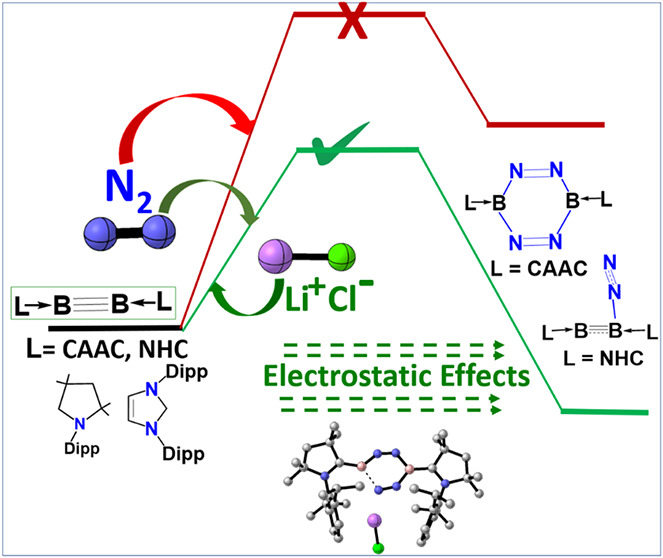

## 1 Introduction

Nitrogen-containing compounds are essential in a wide range of applications, from fertilizers to materials science.^1–7^ Although these compounds can be synthesized from various nitrogen sources, atmospheric dinitrogen (N_2_) is the most abundant and widely available nitrogen source. However, N_2_ is a remarkably stable and inert molecule due to its strong triple bond, characterized by a high bond dissociation energy (225.4 kcal/mol), a large HOMO–LUMO gap (10.82 eV), and no dipole moment.^8^ These factors make its activation particularly challenging, with both fundamental and technological significance. Unlocking the potential of N_2_ for conversion into valuable products requires the design of efficient strategies and systems.^9,10^ Molecular systems for N_2_ activation hold particular relevance in the development of small-scale electrified ammonia synthesis plants and for direct N incorporation into value-added compounds.^11–13^

Owing to the availability of both vacant and filled d orbitals in transition metals, various transition metal-based homogeneous and heterogeneous systems are widely used to activate N_2_.^14–21^ The coordination of N_2_ to transition metals follows a synergistic mechanism involving σ donation and π back-donation, which weakens the N≡N bond and facilitates its activation.^22,23^ Moreover, alkali metals are known to facilitate N_2_ activation across both heterogeneous and molecular systems. For instance, alkali promoters facilitate N_2_ dissociation on Ru surfaces.^24^ In molecular chemistry, alkali cations have been shown to modulate metal-N_2_ reactivity, as demonstrated by Holland and co-workers,^25–28^ and recently, Mazzanti and co-workers reported the role of alkali cations in dinitrogen reduction in uranium systems.^29^

In comparison, the reactivity of main-group species toward N_2_ has been less explored. Nevertheless, recent advances in main-group chemistry, especially involving frustrated Lewis pairs,^30–39^ multiple-bonded systems,,^40–45^ and low-valent compounds,,^46–51^ have demonstrated reactivity similar to that of transition metal complexes. These species have been shown to activate various small molecules such as H_2_,^35,36,42,44,48^ CO_2_,^37,38,49,51,52^ N_2_O,^39^ and even N_2_,^53–57^ owing to their combination of electron-rich and electron-poor sites that allows them to engage and weaken otherwise inert small molecules. This demonstrates that main-group systems add new possibilities for N_2_ activation, like transition metal-based chemistry.^43,58^ One particularly intriguing example of metal-free N_2_ reduction was reported by Braunschweig and co-workers, involving a low-valent boron system (borylene), which facilitates N_2_ fixation and reduction.^53^

Inspired by boron’s demonstrated ability to activate N_2_ ^53,59–61^ the present study investigates the reactivity of boron–boron multiple-bonded compounds toward N_2_. In particular, we focus on two such systems: diboracumulene (**1**) (Scheme 1), stabilized by cyclic (alkyl)­(amino)­carbene (CAAC), and diboryne (**2**) (Scheme 1), stabilized by N-heterocyclic carbene (NHC), isolated and characterized by Braunschweig and co-workers.^62–64^ These electron-donating ligands help compensate for boron’s electron deficiency, resulting in species with an electron-rich B–B core. Moreover, both **1** and **2** differ in their structures, **1** exhibiting longer B–B bonds (1.485 Å in **1** and 1.452 Å in **2**) and shorter B–C bonds compared to **2** (1.448 Å in **1** and 1.480 Å in **2**). Notably, both systems **1** and **2** have been shown to activate stable small molecules such as H_2_, acetylene, and CO under mild conditions.^65–69^ As such, we anticipated that compounds **1** and **2** might also interact with N_2_. However, as will be shown in the results and discussion section below, even the potent multiple-bonded B–B systems **1** and **2** may not be sufficient to coordinate with or activate N_2_, suggesting that an additional strategy is required. Therefore, we investigate the effect of a *salt additive* aiming to make metal-free N_2_ activation both thermodynamically and kinetically favorable.

**Scheme 1:**
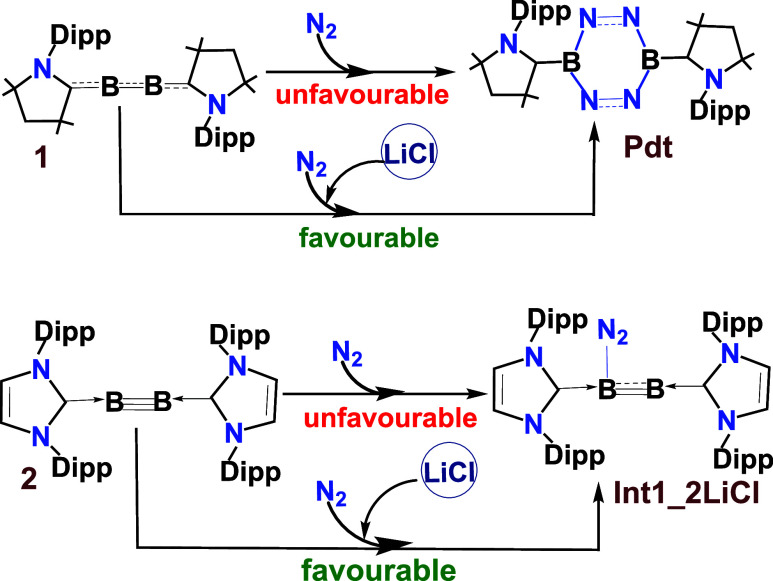
Schematic Representation of the Role of LiCl in CAAC-diboracumulene (**1**)-Mediated N_2_ Activation and NHC-diboryne (**2**)-Mediated N_2_ Binding

Specifically, we propose that the noncovalently interacting lithium chloride (LiCl) additive can generate a local electric field (LEF), thereby inducing dipole moments and polarizing reactant molecules. Therefore, LiCl may facilitate the metal-free N_2_ activation. A previously reported increase in the ionization rate of *t*-butyl chloride was observed in highly concentrated lithium perchlorate (LiClO_4_)-diethyl ether solutions compared to pure diethyl ether, suggesting that lithium salts can modulate chemical reactivity via electrostatic effects.^70^ Moreover, early studies demonstrated that alkali metal ions, positioned using crown ether frameworks, can modulate the emission properties of photoexcited naphthalene derivatives, providing one of the first examples of metal ions acting as a designed local electric field (D-LEFs) source.^71^

Previous independent studies by Shaik and Cotte have demonstrated that internal electric fields, created by the deliberate positioning of salt or ionic additives and referred to as D-LEFs,^72^ which can significantly enhance chemical reactivity. Interestingly, D-LEFs, produced by oriented local field sources, can modulate reactivity in a manner analogous to oriented external electric fields (OEEFs).^73,74^ For instance, Shaik and co-workers reported that the chloride anion additive acts as an internal electric field source, stabilizing the transition state in chlorobenzene oxidative addition to (PMe_3_)_2_Pd.^75^ Moreover, Cotte and co-workers demonstrated that nonconjugated charged functional groups can act as internal oriented electric field sources to tune excited-state energies and reaction pathways^76^ selectively, and protonation-induced fields in pH-switchable organocatalysts can lower Diels–Alder activation barriers via electrostatic transition state stabilization.^77^ The present study shows that the electrostatic effect of LiCl additive facilitates the reactivity of **1** and **2** with N_2_, similar to an OEEF applied along the reaction axis.

Simple and commercially available, LiCl has been found to be a promising promoter or catalyst in a variety of reactions. Most notably, the LiCl-mediated activation of Grignard reagents has been shown to significantly enhance halogen-magnesium exchange reactivity^78,79^ and bimetallic reactive species.^80^ Furthermore, in organozinc chemistry, LiCl promotes the direct insertion of alkyl and aryl iodides into commercial zinc by lowering the energetic barrier from solubilization to oxidative addition.^81^ Additionally, LiCl enables room-temperature ring-opening polymerization of lactide by forming heterometallic complexes in situ.^82^ Moreover, LiCl has been shown to enhance the catalytic activity of RuCl_3_/Ru^0^ systems in the hydrogenation of CO_2_ to produce C_5_^+^ hydrocarbons.^83^

Even though the electrostatic effects of lithium salts are well established, and lithium is known to be particularly effective among the alkali metals for N_2_ activation,^84,85^ interestingly, the first structurally characterized Li–N_2_ interaction was reported^86^ by Stephan and co-workers as a dicationic complex featuring N_2_ bridging two (THF)_3_Li units, [((THF)_3_Li)_2_(μ-N_2_)]^2+^. Furthermore, recent studies have demonstrated that Lewis acids, including boranes and alkali metal cations, induce polarization in metal-coordinated N_2_ and enhance metal-N_2_ interactions through a push–pull mechanism.^87–89^

However, to the best of our knowledge, the combination of B–B multiple-bonded systems with LiCl additives for N_2_ activation has not yet been explored.

In this study, DFT calculations were performed to examine the effect of LiCl on the reaction mechanisms of N_2_ binding and activation by diboryne and diboracumulene species.

To further elucidate the effects of heavier alkali metal salts and bulkier ion pairs on the energetics of N_2_ binding, we extended this study to examine the influence of KCl and the ionic liquids (ILs),^90^ composed of bulkier ions.

## 2 Computational Details

All the reactants, intermediates, and products reported here were preoptimized using GFN2-xTB, and possible conformers were explored using the Conformer and Rotamer Ensemble Sampling Tool (CREST).^91^ Next, for each geometry, crest-best conformers obtained from CREST were reoptimized using the B3LYP^92–94^ functional with Ahlrichs’ def2-TZVP^95^ basis set, including Grimme’s D3 dispersion correction in all calculations.^96^ Since LiCl interacts noncovalently, the CREST nci mode was employed to identify the most stable conformer and favorable position for LiCl. However, it was observed that different positions of the LiCl around system **1** (or **2**) were missing in the CREST conformational search. Therefore, in addition to the conformers generated by CREST, plausible geometries were explored by manually placing LiCl at different positions around the **1**/N_2_ (**Int**_**1**_) species to identify the most stable conformer (Figure S1). As such, the most stable conformer for each system, in both the absence and presence of explicit salt LiCl, was selected and reported here. Transition state search calculations were then performed at the same level of theory, i.e., at the B3LYP-D3/def2-TZVP. Frequency calculations were conducted to confirm the nature of the optimized geometries, ensuring that they were either local minima (no imaginary frequencies) or transition state structures (one imaginary frequency corresponding to the correct normal mode). Intrinsic reaction coordinate (IRC)^97^ calculations were performed to verify that the transition states connect to their respective reactants and products. The resolution of identity (RI),^98^ along with the multipole-accelerated resolution of identity (MARI-J)^99^ approximations, was used for an accurate and efficient treatment of the electronic Coulomb term in the DFT calculations. Geometry optimizations were carried out in both gas and solvent phases. Energy values are reported as Δ*G* at 298.15 K for both phases, with solvent effects included via the COSMO model using a dielectric constant of ε = 2.38 for toluene.^100^ Toluene was selected as the solvent because the reactivity of diboryne and diboracumulene has been predominantly studied in aromatic solvents such as benzene and toluene.^66,69,101^ These two solvents have closely comparable dielectric constants (ε = 2.38 for toluene vs ε = 2.27 for benzene), and the similarity in polarity justifies using toluene as a representative for modeling solvent effects in the case of both systems. Furthermore, we performed calculations to examine the effect of THF-coordinated LiCl on N_2_ binding/activation. To better approximate the solvation effect, we employed a hybrid solvation model combining implicit THF (ε = 7.58) with explicit THF molecules. However, given the system size, the full energy profile was further evaluated at PBE/TZVP/D3/COSMO (ε = 7.58).^102,103^

All DFT calculations were carried out using the TURBOMOLE 7.4.1 quantum chemistry package.^104^ Dipole moment values were computed at the B3LYP-D3/def2-TZVP level of theory.

The local electric field generated at the reaction center by the LiCl additive was determined using the Python code TITAN-2.0.8. Where the electric field strengths are determined using Coulomb’s law.^105^ The charge distribution generated from NBO calculations is utilized for quantifying the associated electric fields at the reaction center. We found that LEF generated by LiCl is 0.012 au (1au = 51.4 VÅ^–1^) on the reaction center. To verify this, we applied an oriented external electric field (OEEF) of 0.012 au Along the reaction axis (*F*_*x*_ > 0) for N_2_ activation by system **1** in the gas phase. Geometry optimizations were carried out under OEEF at the B3LYP-D3/def2-TZVP level of theory, with the “Field” keyword implemented in Gaussian16.^106^ Also, NBO calculations have been carried out at the B3LYP-D3/def2-TZVP level of theory using the Gaussian16.

## 3 Results and Discussion

### 3.1 Reactivity of CAAC-diboracumulene (1) toward N_2_

The N_2_ activation by system **1** is investigated by exploring different mechanistic pathways. As presented in Figure 1, in the first step, the formation of a reactant complex (**RC**) between N_2_ and compound **1** takes place, which is slightly endergonic in both the gas phase and implicit solvent, with energy values of 5.5 and 5.2 kcal/mol, respectively. This is mainly attributed to entropic effects. In the subsequent step, N_2_ binding to the boron atom of **1** can proceed through two possible coordination modes: (a) end-on and (b) side-on. Our calculations show that formation of intermediate **Int**_**1**_ (end-on) is favored by 25.0 kcal/mol over **Int**_**1**_ **′** (side-on). Although the barrier for the formation of **Int**_**1**_ is 25.7 kcal/mol, the process is endergonic, with a free energy change of 15.9 kcal/mol in the solvent phase, suggesting that N_2_ coordination to boron in **1** is unlikely to occur under mild conditions. Therefore, before continuing the mechanism study for the activation of N_2_ from **Int**_**1**_, we investigated the effect of a LiCl additive on the coordination of N_2_ to **1**.

**Figure 1:**
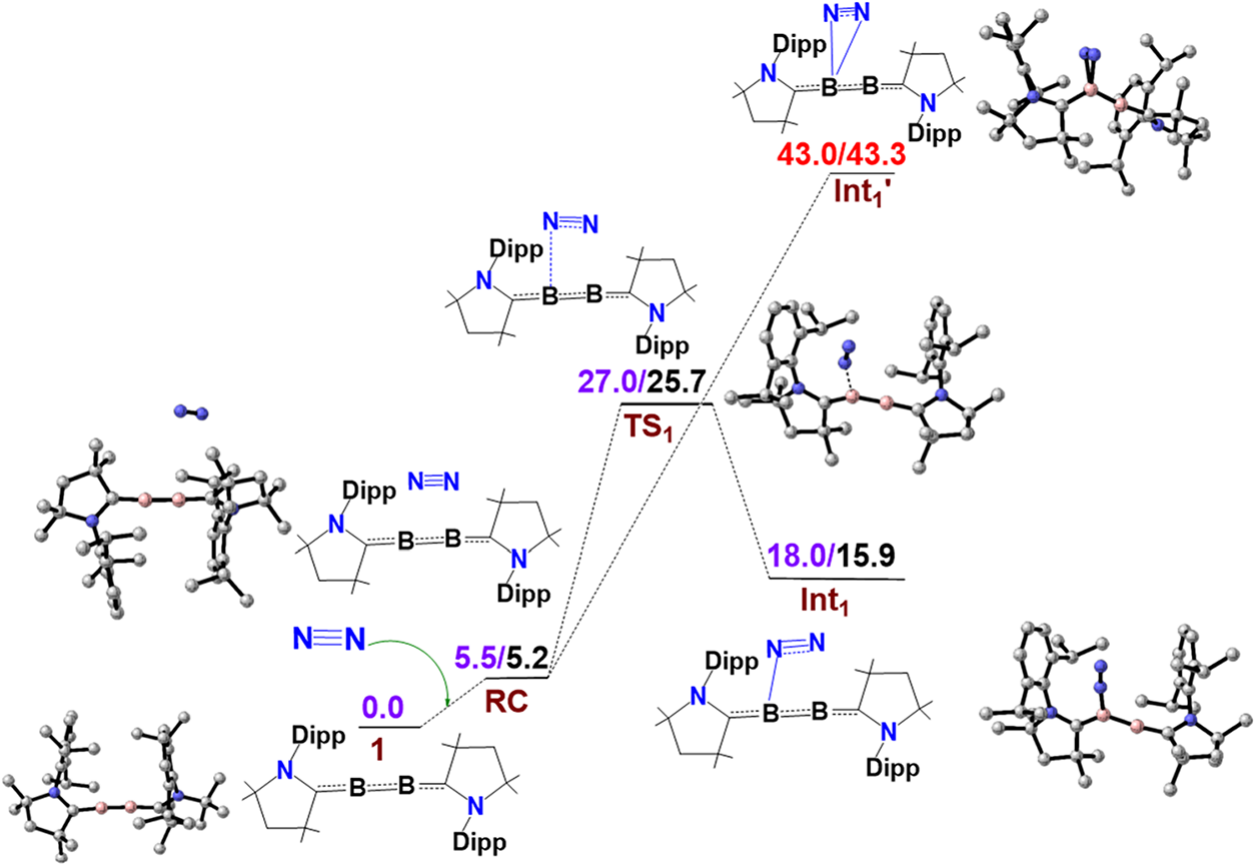
Free energy profile for N_2_-binding modes to **1**. Energy values are in kcal/mol, and two values are in gas phase (purple)/implicit toluene (black), respectively.

### 3.2 Effect of LiCl on the Activation of N_2_ by 1

The LiCl additive is proposed to polarize the N_2_, thereby it can enhance interaction with the boron center and facilitating the formation of the N_2_ adduct. When LiCl is introduced, calculations suggest that formation of the reactant complex (**RC**_**LiCl**_; Figure 2) is exergonic by 10.9 kcal/mol. Moving to the next step in the reaction mechanism, which is the formation of **Int**_**1**_ upon N_2_ binding, here, an important question arises: which conformation of the resulting complex is the most favorable? Specifically, what is the preferred position of LiCl among the possible sites around **Int**_**1**_? One plausible rationale is that LiCl positions itself near the B–N bond (the reaction axis), where Li^+^ can interact with the partially negative boron atom and thereby stabilize the formation of **Int**_**1**_, ultimately leading to a more stable **Int**_**1LiCl**_ species. To confirm this reasoning and identify stable conformation in the presence of explicit LiCl, we explored different possible positions of LiCl around the **Int**_**1**_ (Figure S1). The calculations show that separated Li^+^ and Cl^–^ ions are much more unstable, 50.9 kcal/mol (**Int**_**1LiCl’4**_), higher in energy than the most stable conformation, **Int**_**1LiCl**_, where LiCl is near the B–N bond. This stability arises from the electrostatic interaction between the LiCl salt and the B–N dipole.

**Figure 2:**
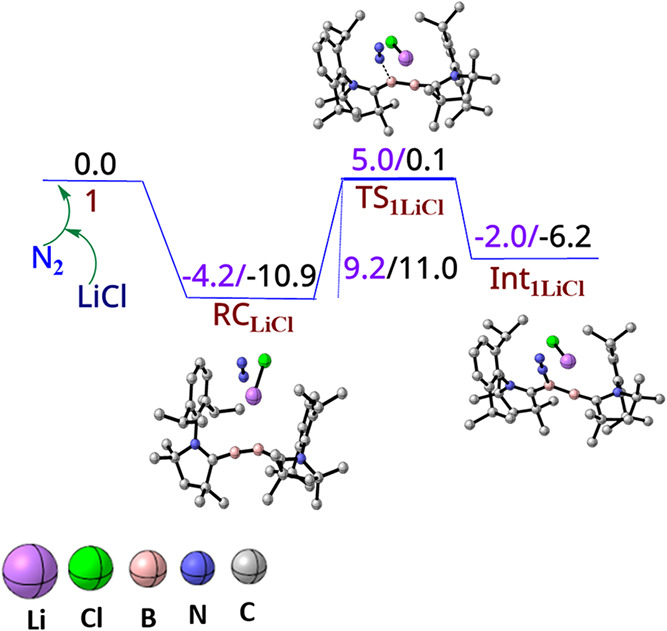
Free energy profile for the coordination of N_2_ to system **1** in the presence of LiCl additive at B3LYP-D3/def2-TZVP level of theory. Two values are in the gas phase (purple)/implicit toluene (black), respectively. Energy values are in kcal/mol, scaled in the solvent phase in the free energy profile.

As shown in Figure 2, in the presence of LiCl, end-on coordination of N_2_ to **1** became both thermodynamically and kinetically more favorable compared to its absence. This is evident in both the gas and solvent phases, as reflected by the exergonic formation of **Int**_**1LiCl**_ with a Δ*G* value of −6.2 kcal/mol and the reduction of Δ*G* ^‡^ to 11.1 kcal/mol (in implicit toluene). Following the favorable *coordination of N*_2_ to **1** in the presence of LiCl, a key question arises: Is the *activation of N*_2_ by system **1** also feasible? To address this, we investigated pathways for N_2_ activation in the presence and absence of LiCl. We first examine the free energy profile for N_2_ activation *without* LiCl, as this enables us to isolate the intrinsic reactivity of the **1** and thereby establish a reference for evaluating the specific impact of the LiCl additive.

After N_2_ coordination to system **1**, we now focus on the energetics of *N*_2_ *-activated intermediates* and products in the absence of LiCl. After the formation of **Int**_**1**_, the remaining boron center can still engage with N_2_, giving rise to different possibilities: (I) *End-on* bridging coordination of the distal nitrogen atom to the other boron center, leading to a μ-η^1^:η^1^-N_2_ intermediate, **Int**_**1**_″ (Figure S2) via transition state **Ts**_**1**_″. However, the formation of **Int**_**1**_″ is highly unfavorable both thermodynamically and kinetically, with Δ*G* and Δ*G* ^‡^ values of 60.1 and 70.0 kcal/mol, respectively. (II) The N_2_ molecule coordinates in a *side-on* fashion to both boron atoms (μ-η^1^:η^1^), forming **Int**_**2**_ **′** (Figure S3), which represents a distinct N_2_-activated intermediate, and (III) end-on binding of another N_2_ molecule to the remaining boron atom, forming the *bis-N*_2_ adduct **Int**_**2**_ (Figure 3). Our calculations suggest that **Int**_**2**_ is more stable than **Int**_**2**_ **′** Δ*G* = 19.7 kcal/mol for **Int**_**2**_ (Figure 3 below) and 23.1 kcal/mol for **Int**_**2**_ **′** (Section S.2 and Figure S3). Even though the formation of **Int**_**2**_ **′** is less favorable, we examined its subsequent reactivity to ensure that various plausible N_2_-activated intermediates and products were considered (Figure S3). Considering studied various N_2_-coordination mechanisms, the formation of the bis-N_2_ adduct (**Int**_**2**_) represents the most favorable route; however, its formation is still endergonic, and the energy barrier is 32.7 kcal/mol (Figure 3). Therefore, it is important to investigate whether the presence of LiCl can make this pathway both kinetically and thermodynamically favorable. As presented in Figure 4, the results indicate that noncovalently interacting LiCl significantly stabilizes both the transition state **TS**_**2LiCl**_ and the intermediate **Int**_**2LiCl**_. This stabilization lowers the energy barrier to 24.4 kcal/mol and, interestingly, makes the process exergonic by 2.7 kcal/mol.

**Figure 3:**
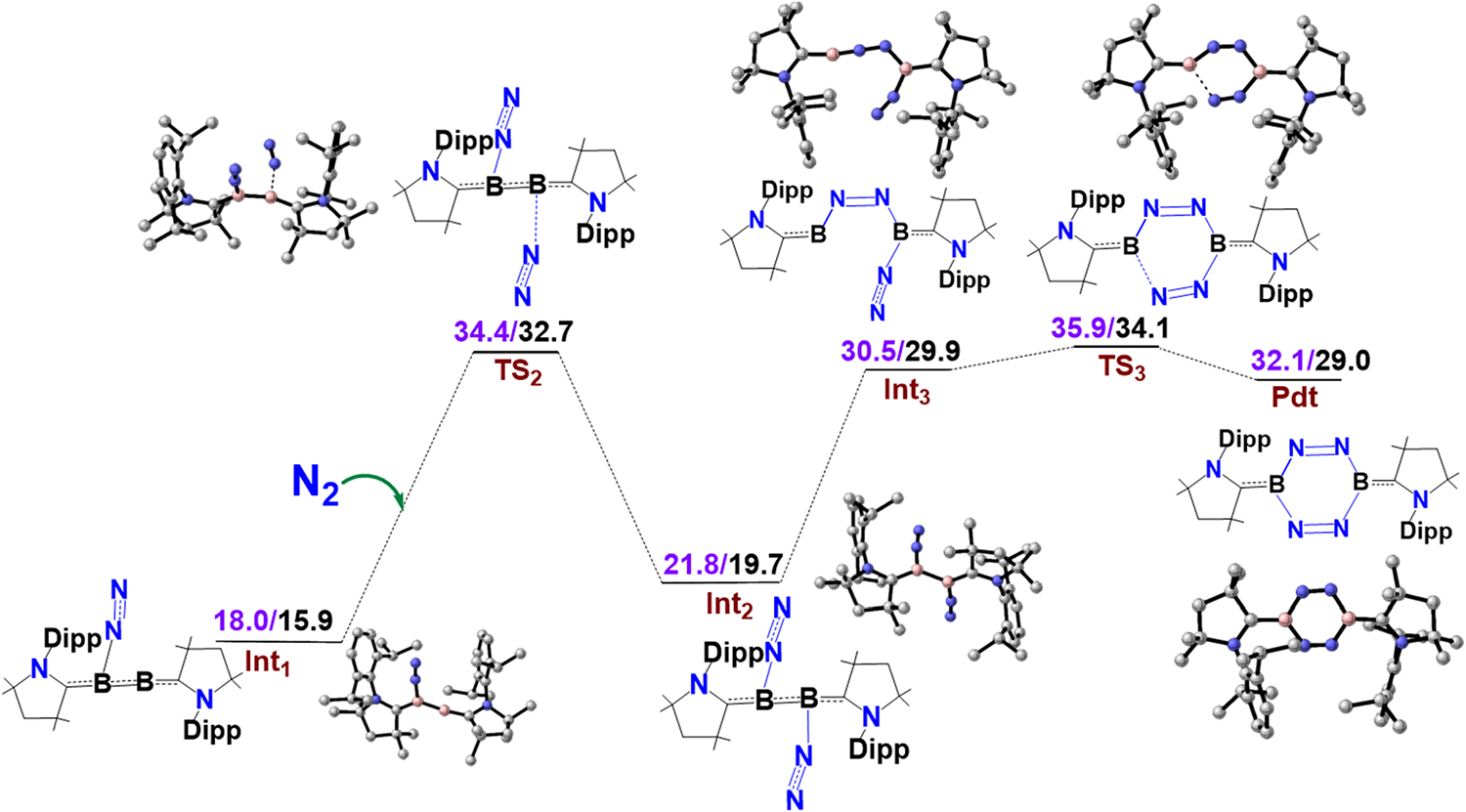
Free energy profile for N_2_ activation by system **1** in the absence of LiCl at B3LYP-D3/def2-TZVP level of theory. Two values are in the gas phase (purple)/implicit toluene (black), respectively. Energy values are in kcal/mol, scaled in solvent phase in free energy profile, and calculated with respect to **1** + N_2_ for the **Int**_**1**_, **Int**_**2**_ **′**, and **1** + N_2_ + N_2_ for subsequent steps.

**Figure 4:**
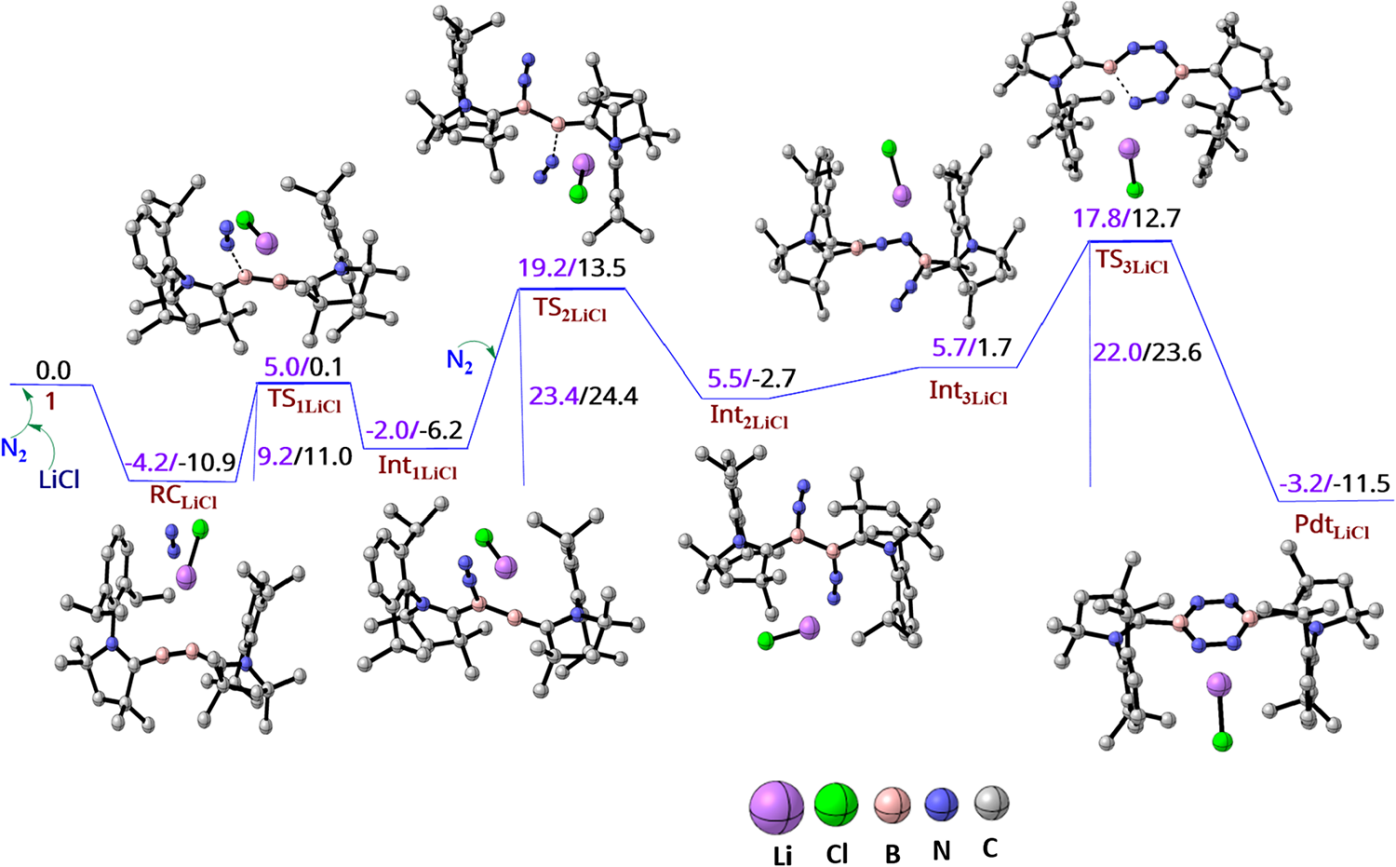
Free energy profile for N_2_ activation by system **1** in the presence of LiCl at B3LYP-D3/def2-TZVP level of theory. Two values are in the gas phase (purple)/implicit toluene (black), respectively. Energy values are in kcal/mol, scaled in the solvent phase in the free energy profile.

In the next step, **Int**_**2LiCl**_ converts into **Int**_**3LiCl**_ through the insertion of one of the coordinated N_2_ molecules into the B–B bond. Despite multiple attempts, a transition state for this transformation could not be located; notably, thermodynamic calculations indicate that **Int**_**2LiCl**_ (Δ*G* = −2.7 kcal/mol) and **Int**_**3LiCl**_ (Δ*G* = 1.7 kcal/mol) are close in free energy, suggesting these intermediates may be in equilibrium and that interconversion between them is feasible. In addition to that, similar to **Int**_**3**_ **LiCl**, in which one N_2_ inserts into the B–B^107^ bond in the presence of another *end-on* coordinated N_2_, a related scenario was reported in an experimental study by Braunschweig and co-workers. In that case, an intermediate formed by CO insertion into the B=B bond of a dihydrodiborene, in the presence of another coordinated CO, was isolated, characterized, and found to be in equilibrium with a species bearing only a single CO coordinated to the dihydrodiborene.^108^

Subsequently, from **Int**_**3LiCl**_, the reaction proceeds to form a six-membered ring product (**Pdt**_**LiCl**_, Figure 4) via **TS**_**3LiCl**_, which involves B–N bond formation. This step has a Δ*G* ^‡^ of 23.6 kcal/mol and is strongly exergonic, with a Δ*G* of −11.5 kcal/mol. By contrast, in the absence of LiCl, formation of the corresponding product (**Pdt**) is much less favorable: it is endergonic by 29.0 kcal/mol, with a significantly higher energy barrier of 34.1 kcal/mol (Figure 3). These results highlight the strong thermodynamic and kinetic favorability for forming the six-membered N_2_-activated product (**Pdt**_**LiCl**_) in the presence of LiCl. Moreover, in a previous study, system **1** was found to react with acetylene, resulting in the formation of a six-membered acetylene-activated product, diborabenzene.^109^

The influence of LiCl on the reaction energetics was evaluated by comparing dipole moments in transition states, intermediates, and products involved in reaction profiles in the presence and absence of LiCl additive. As shown in Table 1 below, we found that LiCl induces a larger dipole moment, indicative of increased charge separation and enhanced molecular polarity. Such polarization stabilizes the transition states and intermediates by facilitating electron flow and promoting bond cleavage. As such, the presence of LiCl facilitates N≡N bond activation by **1**, making the process both kinetically and thermodynamically favorable. Furthermore, to investigate the electrostatic contribution of LiCl, an oriented external electric field was applied along the reaction axis, simulating its polarizing effect.

**Table 1:** Molecular Dipole Moment (μ in *D*) of B3LYP-D3/def2-TZVP Optimized Geometries for Transition States, Intermediates, and Products in the Gas Phase

species without LiCl	μ	species with LiCl additive	μ
**TS**_**1**_	2.7632	TS1LiCl	5.6715
**Int**_**1**_	4.6455	Int1LiCl	5.3244
**TS**_**2**_	2.9290	TS2LiCl	6.9356
**Int**_**2**_	2.6599	Int2LiCl	9.2868
**Int**_**3**_	1.8560	Int3LiCl	6.7656
**TS**_**3**_	0.8983	TS3LiCl	7.5078
**Pdt**	2.0873	PdtLiCl	6.6244

### 3.3 Electrostatic Effect in Activation of N_2_ by 1

A key question regarding the enhanced reactivity in the presence of the LiCl additive is whether it arises from an electrostatic effect exerted by LiCl on the N_2_-adduct. To investigate this, we quantified the local electric field (LEF) generated by the point charges of LiCl in the transition state (**TS**_**1LiCl**_), along the reaction axis. While the approach of N_2_ and B–N bond formation occurs along the *z*-axis, the B–N formation leads to reorganization within the B=B unit, which overall defines the reaction axis. Consequently, the reaction axis lies along the *x*-direction (Figure 5a), corresponding to the direction of electron reorganization from N≡N to B = B in the transition state, leading to the formation of the N≡N–B=B (reaction moiety in **Int**_**1**_). Our calculations show that LiCl can induce LEF as high as 0.012 au (0.6 VÅ^–1^) at the reaction center, and to verify this, we applied an oriented external electric field (OEEF) of 0.012 au along the reaction axis (*F_x_* > 0).

**Figure 5:**
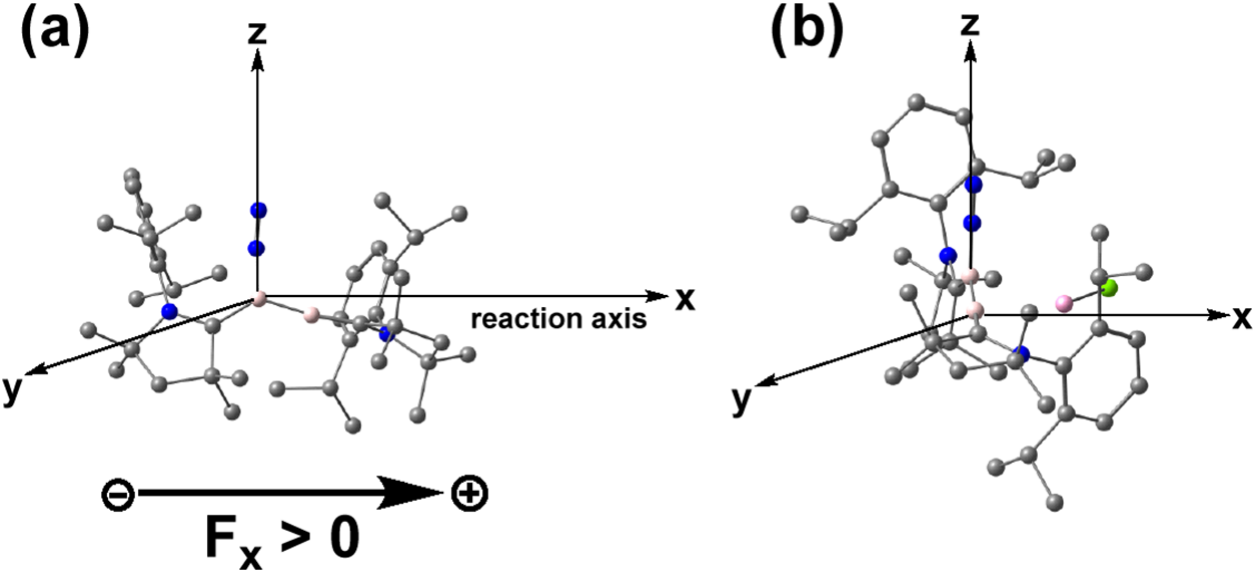
(a) Representation of the reaction axis and the direction of the applied oriented external electric field (OEEF, *F_X_* > 0, according to Gaussian convention), shown in the optimized geometries of transition states **TS**_**1**_; (b) Orientation of the LiCl additive along the reaction axis, in **TS**_**1LiCl**_.

Table 2 presents the free energy barriers with and without LiCl, and in the presence of an OEEF (*F_X_* = 0.012 au). Both the reactant complexes and transition states were optimized under this field (Figure S4). Δ*G* ^‡^ for the first step under *F_X_* > 0 is calculated relative to the reactant complex (**RC**_**OEEF**_). In the presence of LiCl, the barrier is calculated relative to **RC**_**LiCl**_ (as shown in Figure 2), allowing direct comparison of Δ*G* ^‡^ in the presence of both OEEF and LiCl. Furthermore, to enable consistent comparison of Δ*G* ^‡^, the reference state for the subsequent steps in the energy profiles shown in Figures 3 and 4 should be the reactant complex of **1**, two N_2_ molecules, and LiCl (**RC**_**2N2LiCl**_; Figure S5d). However, formation of **RC**_**2N2LiCl**_ is endergonic by 4.0 kcal/mol relative to the **RC**_**LiCl**_ + N_2_. Therefore, ΔG^‡^ for the subsequent steps (for **Int**_**2**_ and **Pdt**) is calculated relative to **RC**_**OEEF**_ + N_2_ in the presence of OEEF, and to **RC**_**LiCl**_ + N_2_ in the presence of the LiCl additive (as shown in Figure 4). Also, Δ*G* ^‡^ without LiCl (or without the external field) are calculated with respect to the separated reactant species (**1** + N_2_) for the formation of the first intermediate (**Int**_**1**_), and to (**1** + 2N_2_) for the subsequent steps (**Int**_**2**_, **Pdt** in Figure 3), as the formation of the reactant complex (**RC**, and **RC**_**2N2**_) is endergonic (Figure S5) by 5.5 and 18.0 kcal/mol due to entropy factor.

**Table 2:** Free Energy Barriers (Δ*G* ^‡^) Shown Here are Calculated at the B3LYP-D3/def2-TZVP Level of Theory in the Absence and Presence of LiCl and an Oriented External Electric Field (OEEF, *F_X_* = 0.012 a.u.)

reaction’s steps	field (a.u.)	Δ*G* ^‡^ (kcal/mol)
	0.00	27.0
**Int**_**1**_	Explicit LiCl	9.2
	*F_X_* = 0.012	11.8
	0.00	34.4
**Int**_**2**_	Explicit LiCl	23.4
	*F_X_* = 0.012	22.1
	0.00	35.9
**Pdt**	Explicit LiCl	22.0
	*F_X_* = 0.012	22.7

Our calculations under OEEF reveal that the Δ*G* ^‡^ for the formation of **Int**_**1**_ is reduced to 11.8 kcal/mol, closely aligning with the Δ*G* ^‡^ observed in the presence of LiCl (9.2 kcal/mol). Both are significantly lower than the barrier in the field-free scenario (27.0 kcal/mol, see Table 2). For the formation of **Int**_**2**_, the barriers are 22.1 kcal/mol with OEEF and 23.4 kcal/mol with LiCl, compared to 34.4 kcal/mol without either. Similarly, the barrier for product (**Pdt**) formation is 22.7 kcal/mol under OEEF and 22.0 kcal/mol with LiCl, whereas it is 35.9 kcal/mol in the absence of both. These results suggest that LiCl can effectively mimic the effect of an OEEF.

### 3.4 NBO Analysis of B_2_–N_2_ Interactions

To gain insight into electron-transfer processes and orbital interactions during *end-on* N_2_ coordination to the B–B unit of system **1**, we performed Natural Bond Orbital (NBO) analysis of the N_2_-bound transition states **TS**_**1**_ and **TS**_**2**_. The role of LiCl in modulating these interactions was elucidated by examining second-order perturbation energies (E(2)) in both the absence and presence of LiCl. As shown in Figures 6 and 7, E(2) reveals a trend in which LiCl facilitates N_2_ binding primarily by enhancing the σ donation (LP N → LP* B). For **TS**_**1**_, this interaction energy increases from 106.8 to 112.69 kcal/mol upon the addition of LiCl. Therefore, the addition of LiCl increases the E(2) of the LP(1) N → LP* (1) B interaction by 5.89 kcal/mol. Similarly, in **TS**_**2**_, the σ donation rises from 91.88 to 105.99 kcal/mol (**TS**_**2LiCl**_). This indicates Li^+^ induced electron withdrawal from the dinitrogen unit, which polarizes the N≡N bond and enhances the nucleophilicity of the nitrogen lone pair toward the boron empty orbital (LP* of B). In **TS**_**1**_, the B → N_2_ back-donation (BD B–B → BD* N_2_) remains relatively stable, shifting only slightly from 4.62 to 4.55 kcal/mol. In **TS**_**2**_, it decreases more from 4.80 to 2.71 kcal/mol. Therefore, transition states with LiCl-additive are stabilized by the gain in σ-bonding and secondary π-type donations (BD N_2_ → LP* B). Specifically, the total π-type donation from N_2_ to boron increases from 7.00 to 8.00 kcal/mol in **TS**_**1**_ and from approximately 7.60 to 12.57 kcal/mol in **TS**_**2**_.

**Figure 6:**
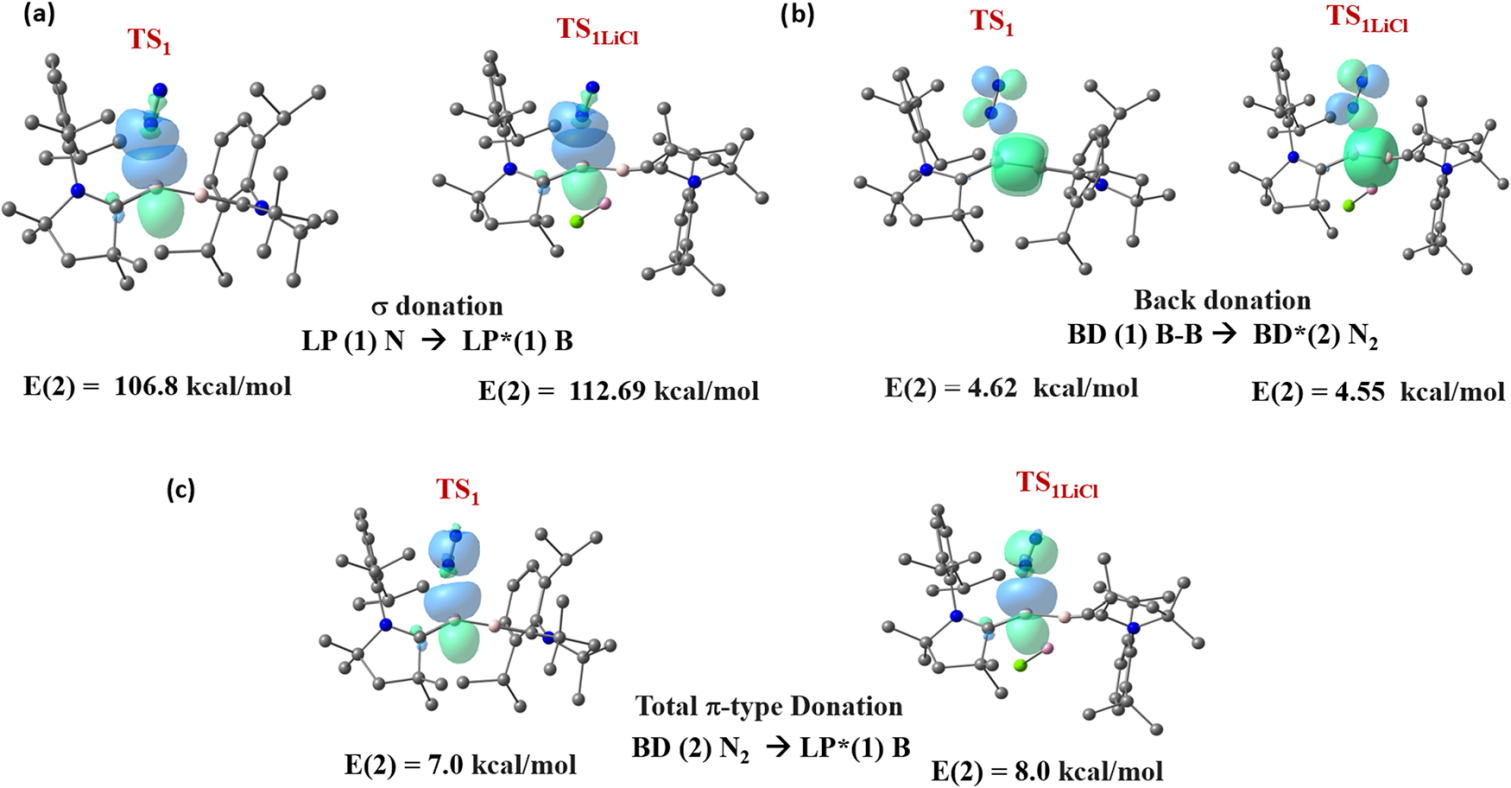
NBO second-order perturbation energies (E(2), kcal/mol) quantifying the B–N_2_ orbital interactions in transition states **TS**_**1**_ and **TS**_**1LiCl**_, highlighting the effect of LiCl on (a) σ-donation, (b) back-donation. and (c) π-type donation.

**Figure 7:**
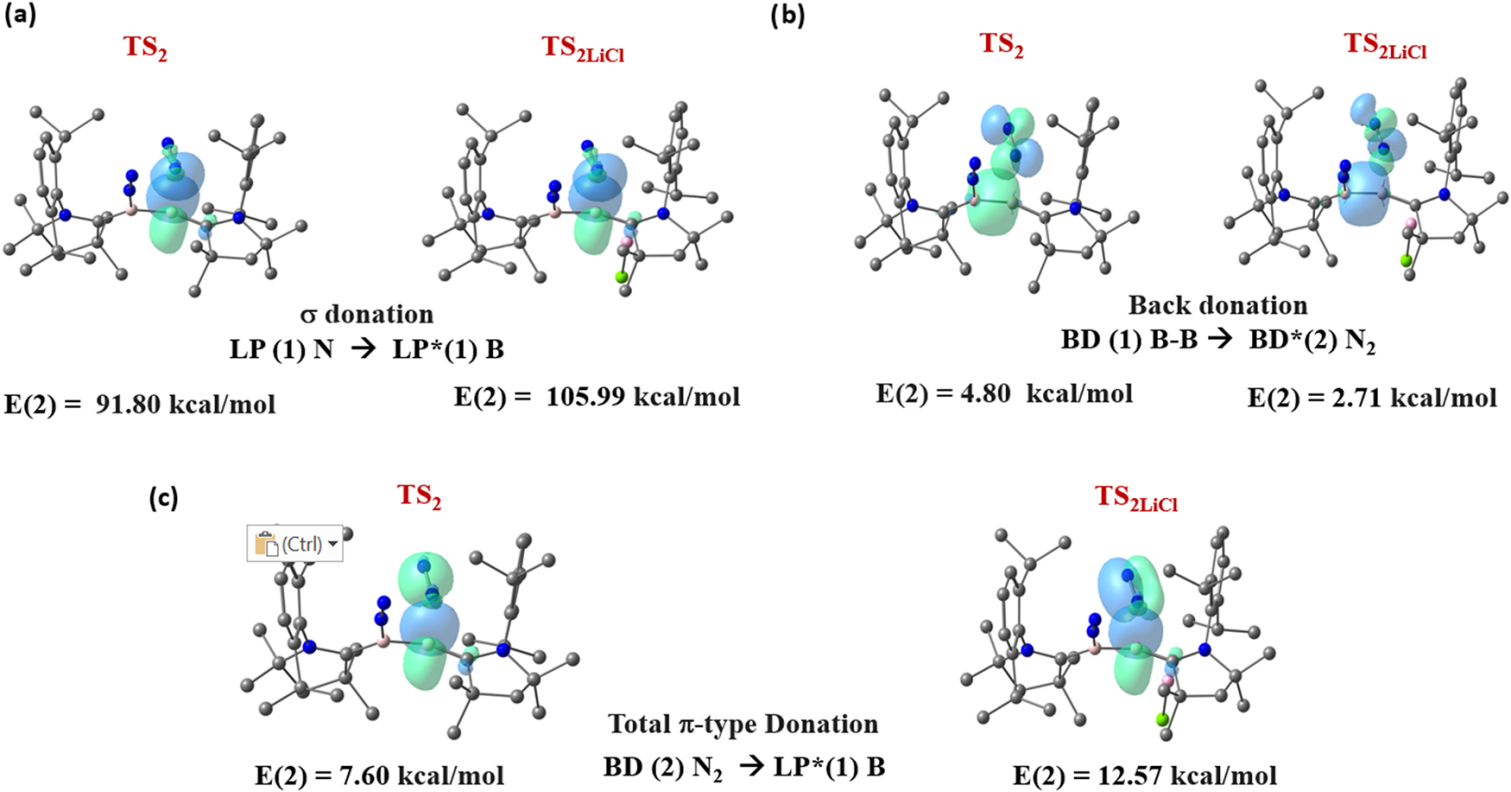
NBO second-order perturbation energies (E(2), kcal/mol) quantifying the B–N_2_ orbital interactions in transition states **TS**_**2**_ and **TS**_**2LiCl**_, highlighting the effect of LiCl on (a) σ-donation, (b) back-donation. and (c) π-type donation.

Furthermore, the effect of LiCl on N_2_ binding by system **1** is analyzed by comparing the bond distances in the presence and absence of LiCl in the transition states (Table 3 below). Interestingly, the presence of LiCl consistently shortens the B–N bond in **TS**_**1LiCl**_ and **TS**_**2LiCl**_, while the N–N bond remains almost unchanged (≈1.10 Å). This indicates that LiCl primarily promotes N_2_ binding by strengthening the B–N donor–acceptor interaction, consistent with the NBO analysis presented above, which supports enhanced σ-donation from N_2_ to B in the presence of LiCl. Notably, in **TS**_**3**_, elongation of the N–N bond becomes more pronounced in the presence of LiCl, indicating enhanced activation of N_2_. Further, supported by the change in N–N stretching frequencies from 1916.0 cm^–1^ and 1730.3 cm^–1^ in **TS**_**3**_ to 1785.6 cm^–1^ and 1698.6 cm^–1^ in **TS**_**3LiCl**_, consistent with increased population of N_2_ antibonding orbitals induced by LiCl.

**Table 3:** Comparison of Key Bond Distances (Å) for Transition States Optimized at the B3LYP-D3/def2-TZVP Level in the Gas Phase, in the Absence and Presence of LiCl, Highlighting the Effect of LiCl on B–N, N≡N, and B–B Bonding along the Reaction Pathway

transition states	B–N (Å)	N≡N (Å)	B–B (Å)
TS_1_	1.976	1.099	1.535
TS_1LiCl_	1.956	1.099	1.529
TS_2_	2.049	1.099	1.645
TS_2LiCl_	1.988	1.097	1.643
TS_3_	2.011	1.155	3.229
TS_3LiCl_	2.032	1.184	3.216

### 3.5 Effect of THF-Solvated LiCl on N_2_ Activation by 1

LiCl forms *contact ion pairs* in THF,^110^ and its experimental solubility in THF is 54 g/L. In this study, we therefore included explicit THF molecules coordinated to the Li–Cl to investigate its electrostatic effect in N_2_ activation. Because adding explicit solvent molecules substantially increases the computational cost at the B3LYP-D3/def2-TZVP level, the hybrid solvation model is computed at the PBE/TZVP/D3 level of theory.

To assess THF coordination to LiCl,^111^ we calculated the Gibbs free energy for LiCl­(THF)_4_ and LiCl­(THF)_3_, which was found to be identical in both cases (Δ*G* = −14.6 kcal/mol; Figure S8). This raises the question of whether LiCl­(THF)_4_ or LiCl­(THF)_3_ should be incorporated into the transition states and intermediates to investigate the effect of THF-coordinated LiCl on N_2_ activation.

Although cooperative activation could, in principle, proceed via a coordinatively saturated LiCl­(THF)_3_ species with subsequent THF dissociation, structural analysis reveals that in LiCl­(THF)_4_ one THF molecule is already weakly bound (Li–O = 4.26 Å: Figure S9), and this elongation becomes even more pronounced in **Int**_**1LiCl[THF]4**_, where the Li–O distance extends to 4.5 Å during interaction with the B–N_2_ fragment, thereby polarizing the N≡N bond (N–N = 1.13 Å).

Moreover, the Δ*G* of formation for **Int**_**1[LiCl(THF)**_**3**_ **]**_ and **Int**_**1[LiCl(THF)**_**4**_ **]**,_ relative to LiCl­(THF)_4_, is 5.1 and 12.9 kcal/mol, respectively (Figure S10). This indicates that treating THF as a separate species (as in the case of Δ*G* for **Int**_**1[LiCl(THF)**_**3**_ **]**_) is more favorable due to the entropy factor than retaining all four THF bound in **Int**_**1[LiCl(THF)**_**4**_ **].**_ Since THF is a solvent, retention of THF coordination in intermediates and transition states is more consistent (as in **Int**_**1[LiCl(THF)**_**4**_ **]**_). Therefore, we computed the free-energy profile for N_2_ binding and activation in the presence of LiCl­(THF)_4_ to represent the first coordination sphere of Li^+^. Calculations presented in Figure S11, indicate that formation of the reactant complex (**RC**_**LiCl[THF]4**_) and the first intermediate (**Int**_**1LiCl[THF]4**_) is endergonic, primarily due to entropic contributions, even though Δ*E* calculations suggest that N_2_ coordination is exothermic. For N_2_ binding, Δ*G* ^‡^ and Δ*E* ^‡^ are 21.6 and 8.6 kcal/mol, respectively. Interestingly, while formation of the N_2_-activated product (**Pdt**_**LiCl[THF]4**_) is slightly endergonic, the barrier for activation remains modest (Δ*G* ^‡^ = 15.1 kcal/mol), highlighting that N_2_ activation is kinetically favorable in the presence of explicit LiCl­[THF]_4_.

Thus, both gas-phase and solvated calculations demonstrate that the qualitative effect of LiCl on N_2_ coordination/activation to system **1** is favorable.

Since LiCl interacts noncovalently with the 1-_N2_ system, a detailed follow-up study will be carried out to explore the effects of solvated Li^+^ ions and Li salt on N_2_-activation.

### 3.6 Effect of KCl and Ionic Liquids (ILs) Additive on N_2_ Binding to 1

The influence of KCl and, specifically, two ILs containing the 1-butyl-1-methylpyrrolidinium cation: (i) [C_4mpyr_]^+^[eFAP]^−^, selected because it is known for good N_2_ solubility,^112^ and (ii) [C_4mpyr_]­[PF_6_]^−^, featuring a smaller anion for comparison on the formation of **Int**_**1**_ have studied. Our calculations show that the free energy of formation of **Int**_**KCl**_ (Figure S12) is 0.2 kcal/mol, with an associated free energy barrier of 16.0 kcal/mol. These results indicate that N_2_ binding is more favorable in the presence of LiCl (Figure 2). Therefore, we did not investigate subsequent steps of the N_2_ activation mechanism in the presence of explicit KCl.

Furthermore, in the presence of explicit ILs [C_4_mpyr]^+^[eFAP]^−^ and [C_4_mpyr]^+^[PF_6_]^−^: Δ*G* ^‡^ for **Int**_**1IL**_ is 24.9 kcal/mol, and 21.5 kcal/mol, respectively (Figure S13). However, unlike the presence of LiCl, where N_2_-binding was exergonic, here in the presence of both ILs, the process remains endergonic by 7.1 and 10.8 kcal/mol, respectively. These findings suggest that KCl and bulkier ion pairs, such as [C_4_mpyr]^+^ and [eFAP]^−^ or [PF_6_]^−^, exert only a minor electrostatic effect on polarizing N_2_ to promote its coordination with the **1**, compared to LiCl.

### 3.7 Reactivity of NHC-Diboryne (2) toward N_2_

As outlined in the introduction, the reactivity of NHC-diboryne (**2**) toward N_2_ is also explored. The results (Figure 8) show that the Δ*G* and Δ*G* ^‡^ for the first step, leading to formation of the intermediate (**Int1_2**) upon N_2_ coordination to **2**, are 19.5 and 29.9 kcal/mol, respectively, in implicit toluene. Therefore, formation of **Int1_2** is unlikely to occur under mild conditions, particularly since it is endergonic. Subsequently, the coordination of the second N_2_ to another boron center possesses a high barrier (40.2 kcal/mol) and is also endergonic by 19.4 kcal/mol in the implicit toluene. Next, similar to N_2_ binding and activation by system **1**, the effect of LiCl is investigated to assess whether N_2_ binding to diboryne (**2**) could be favored.

**Figure 8:**
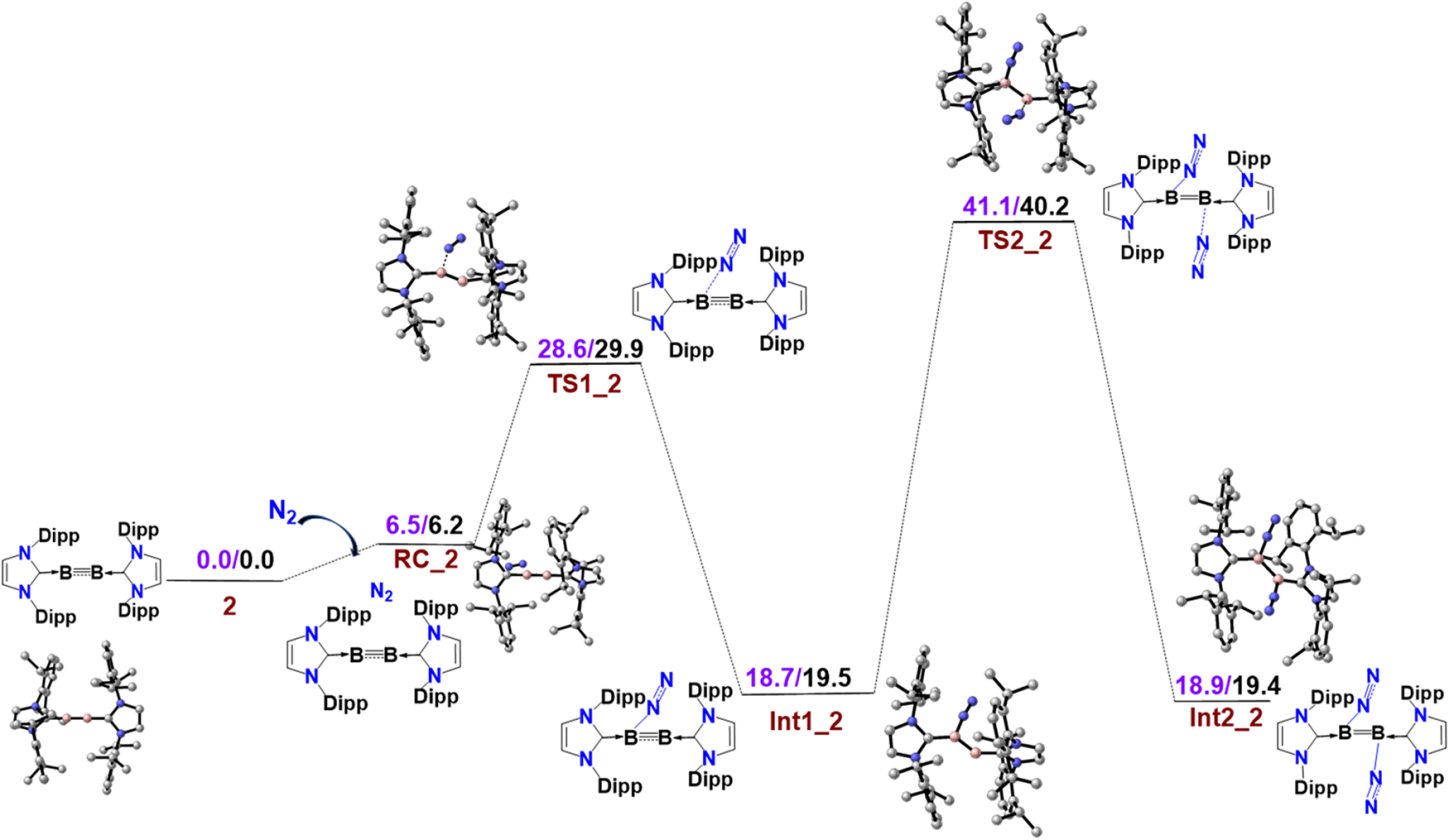
Free energy profile for N_2_ binding with system **2** at the B3LYP-D3/def2-TZVP level of theory. Two values are shown for gas phase (purple)/implicit toluene (black), respectively. Free energies are given in kcal/mol.

Figure 9 below presents the LiCl-mediated free energy profile for N_2_ binding. The formation of the reactant complex of **2**, LiCl, and N_2_ (labeled **RC**_**2LiCl**_) is highly favorable, with Δ*G* = −24.0 kcal/mol. As a result, although the transition state is also stabilized in the presence of LiCl, the Δ*G* ^‡^ for **Int1_2LiCl** is 26.9 kcal/mol, reduced by only 3 kcal/mol compared to the absence of LiCl. Therefore, N_2_ binding to diboryne **2** is less favorable than to diboracumulene **1**.

**Figure 9:**
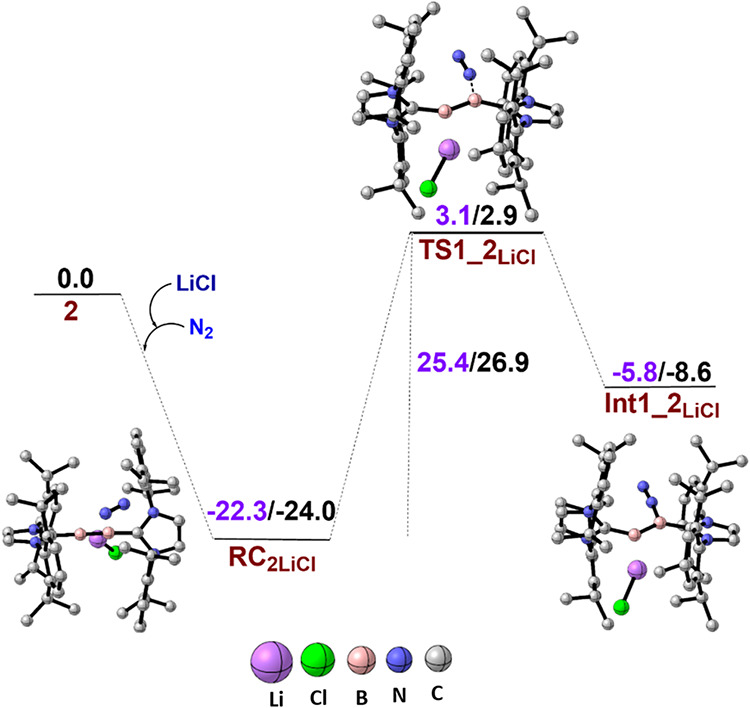
Free energy profiles in the presence of LiCl salt additive in N_2_ binding with system **2** at B3LYP-D3/def2-TZVP level of theory in implicit toluene. Energy values are in kcal/mol.

Since the Δ*G* ^‡^ for the next step, formation of **Int2_2** upon binding of the second N_2_ to **2**, is high in the absence of LiCl (40.2 kcal/mol), and the effect of LiCl on the first step is less pronounced than in the case of system **1**, the effect of LiCl on the second N_2_ binding is expected to be similarly limited. Therefore, after investigating the role of LiCl in N_2_ binding to **2**, the reaction mechanism for the next step or for N_2_ activation by system **2** was not explored.

The higher reactivity of system **1** arises from the stronger π-donor character of the CAAC ligand, whereas the weaker π-donating ability of NHC ligands renders system **2** less reactive. The B–N bond distances in **TS1_2** (1.895 Å) and **TS1_2LiCl** (1.918 Å) indicate that LiCl does not significantly strengthen the B–N interaction in system **2**. This can be rationalized by steric hindrance from the NHC ligand, which restricts the approach of LiCl to the B–N_2_ unit, and by the very small dipole moment (0.09 D) of **2**, which might limit its ability to respond to electrostatic perturbations. Because N_2_ is nonpolar, the potent ionic additive, such as LiCl, might not be sufficient to modulate the reactivity of the NHC diboryne toward N_2_. As highlighted in the literature,^113^ effective reactivity modulation by oriented external electric fields requires reactants to possess appreciable dipole moments; consequently, LiCl does not effectively facilitate N_2_ activation by system **2**.

## 4 Conclusion

This study highlights the significant influence of explicit electrostatic environment in promoting the activation of the inert molecule, N_2,_ under metal-free and mild conditions. Through a detailed mechanistic study of the coordination and activation of N_2_ by CAAC-diboracumulene (**1**) and NHC-diboryne (**2**), we found that the introduction of LiCl additive induces a substantial reduction in activation barriers and transforms an otherwise endergonic reaction into an exergonic one via electric field effects. Our calculations reveal that the free energy barrier for N_2_ binding to system **1** drops from 25.7 to 11.0 kcal/mol with LiCl. Interestingly, for the six-membered N_2_-activated product (**Pdt**), Δ*G* ^‡^ decreases from 34.1 to 23.6 kcal/mol with the **1**/LiCl combination. Additionally, ΔG changes from 29.0 kcal/mol without LiCl to −11.5 kcal/mol with LiCl, indicating a significant thermodynamic favorability induced by the LiCl. In contrast, for system **2**, the Δ*G* ^‡^ for N_2_ binding is 29.9 kcal/mol with LiCl and 26.9 kcal/mol without it. These results indicate that compound **2** is less reactive toward N_2_ than compound **1**. Notably, the effect of LiCl on facilitating N_2_ coordination is considerably less pronounced in compound **2** than in the case of **1**. As a result, the weaker N_2_ binding observed for **2** suggests that N_2_ activation by this system is unlikely to occur. Moreover, THF-coordinated-LiCl calculations show that N_2_ activation is favorable.

Furthermore, we examined the impact of explicit IL, ionic liquids, [C_4_mpyr]^+^[eFAP]^−^ and [C_4_mpyr]^+^[PF_6_]^−^, on the coordination of N_2_ to **1**. These bulkier ion pairs showed a less significant influence on promoting N_2_ binding compared to LiCl, emphasizing that not only the presence of ions but their size and coordination behavior are key to tuning reactivity. These insights provide valuable guidance for experimental chemists in designing more efficient and tunable main-group-mediated N_2_ activation systems through the strategic use of salt additives.

## Supplementary Material



## Notes

Figures and descriptions mentioned in the manuscript, and *XYZ* coordinates of all optimized geometries; the B3LYP-D3/def2-TZVP and PBE/TZVP/D3/COSMO­(THF) optimized geometries for various possible positions for LiCl around the **Int_1_** in the gas phase; free energy values for THF coordination to LiCl: LiCl­[THF]_3_ and LiCl­[THF]_4_ computed at PBE/TZVP/D3/COSMO­(THF) (PDF)
