# Supplementary material for: Effect of Salt Additives on Dinitrogen Activation Mediated by Boron-Based Compounds: Insights from Theory
Source: Inorg Chem. 2026 May 20;65(22):12140–52. doi: 10.1021/acs.inorgchem.5c05891 (PMC13250978; doi:10.1021/acs.inorgchem.5c05891)
Supplement: Supplementary file 1 [file ic5c05891_si_001.pdf]

Supporting Information

Effect of Salt Additives on Dinitrogen Activation Mediated by Boron-Based Compounds: Insights from Theory

Shailja Jain[a,b*] and Johannes Kästner[a]

[a] Institute for Theoretical Chemistry, University of Stuttgart, Stuttgart, 70569, Germany.

[b] Department of Chemistry, School of Advanced Sciences, VIT-AP University, Amaravati, 522241, Andhra Pradesh, India.

Email: shailja.jain@vitap.ac.in

Table of Contents

S.1 Conformations of **Int$_{1LiCl}$** ………………………………………………………....S2-S2

S.2 Activation of N_2 by **1** in the Absence of LiCl……………………………………….S3-S4

S.3 Optimized Geometries of the Reactant Complex and Transition States in the Presence of an Oriented External Electric Field (OEEF) with F_X = 0.012……………………………S5-S5

S.4 Formation of the Reactant Complexes………………………………………………..S6-S6

S.5 Energy Profiles (ΔE, kcal/mol) in Implicit solvents)………………………….…………………………………………………….S7-S7

S.6 Effect of THF-solvated LiCl on N_2 activation by **1**…………………………………S8-S9

S.7 Effect of KCl and Ionic Liquids (ILs) additive on N_2 binding to **1**………………..S10-S11

S.8 XYZ coordinates of B3LYP-D3/def2-TZVP optimized geometries in the gas and implicit toluene phases, and PBE/TZVP/D3 optimized geometries in hybrid solvation model………………………………………………………………………….S11-S152

S.1 Conformations of **Int$_{1LiCl}$**

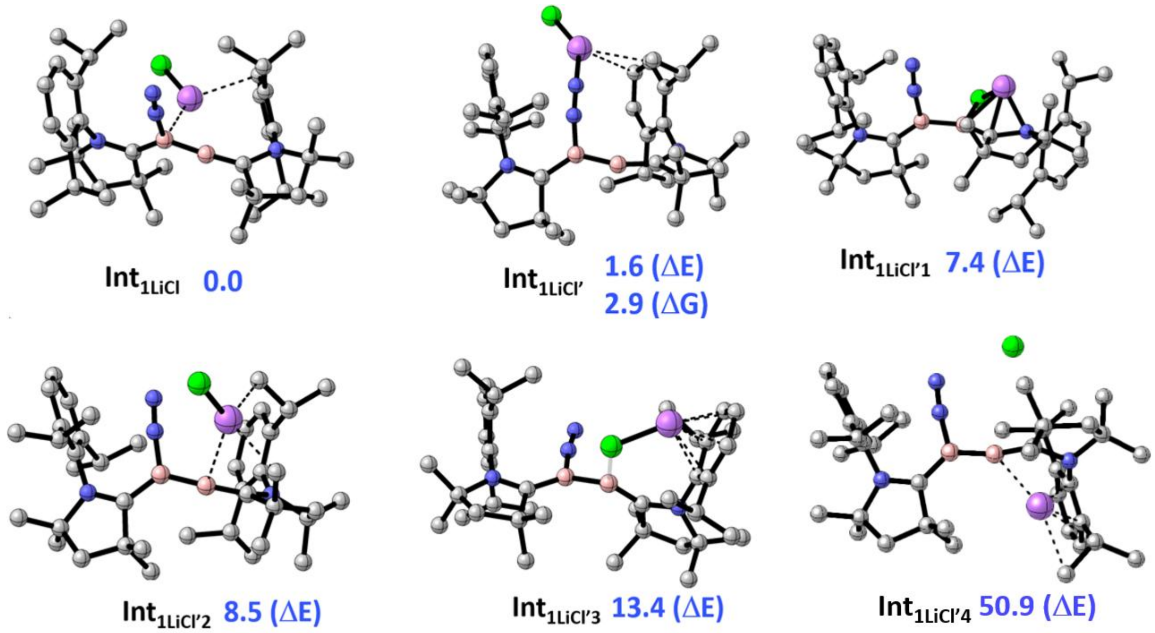

Figure S1. The B3LYP-D3/def2-TZVP optimized geometries for various possible positions for LiCl around the **Int$_1$** in the gas phase. Hydrogen atoms are omitted from the optimized geometries for clarity. Energy values shown in blue are in kcal/mol, relative to the most stable intermediate (**Int$_{1LiCl}$**).

S.2 Activation of N_2 by **1** in the Absence of LiCl

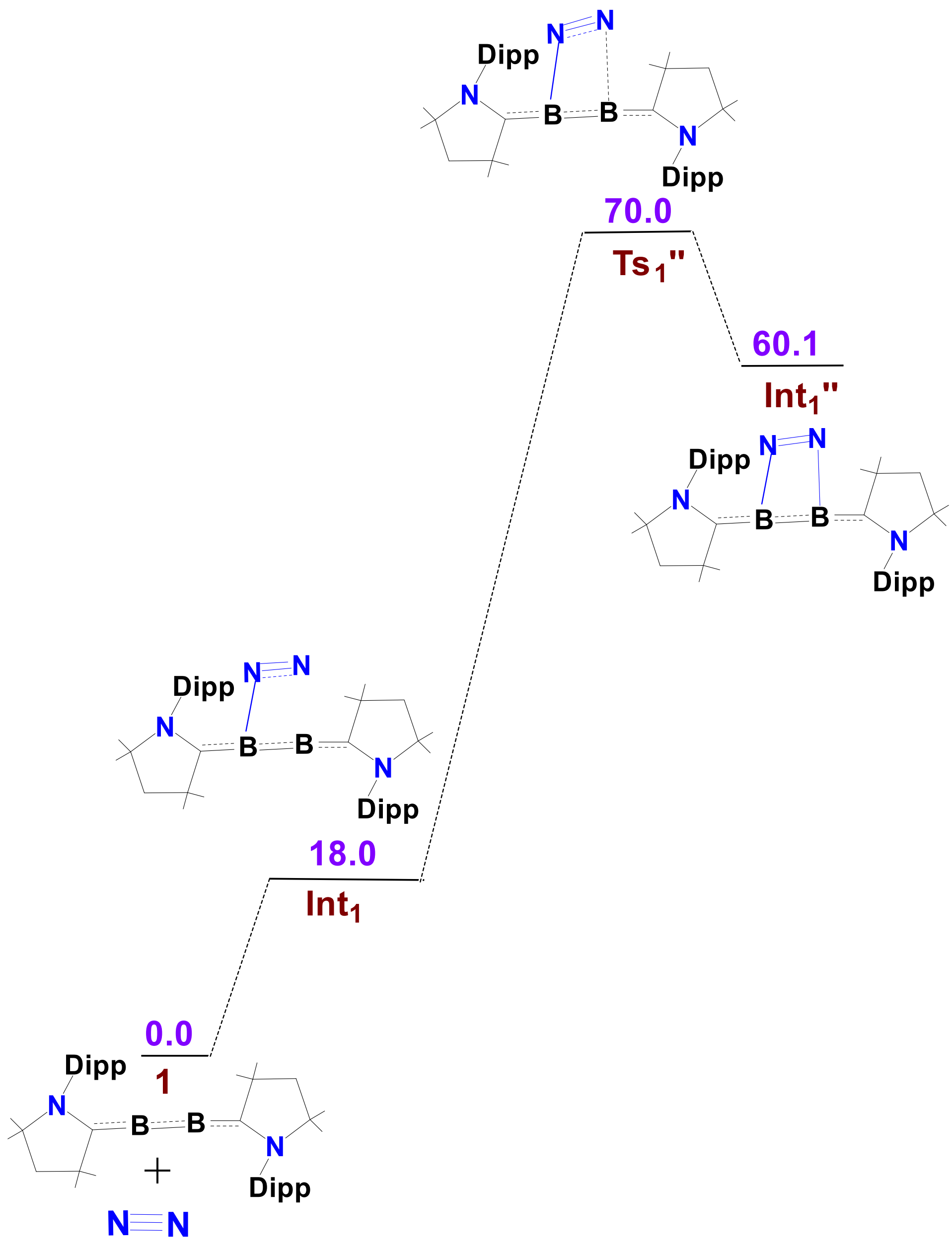

Figure S2. Free energy profile for the formation of **Int1**'' *via* end-on coordination of the distal nitrogen atom to the other boron at B3LYP-D3/def2-TZVP level of theory in the gas phase. Energy values are in kcal/mol.

As shown in Figure S3 below, from intermediate **Int2′**, the N_2-activated species **Int1a** is formed *via* transition state **TS1a**, with a $\Delta G^{\ddagger}$ of 43.8 kcal/mol. This transformation is exergonic by 22.8 kcal/mol and involves complete cleavage of the N≡N bond, with each nitrogen atom forming a B-N bond to separate boron centres. Subsequently, two possible pathways emerge

from **Int$_{1a}$**: i) Insertion of one nitrogen into the CAAC carbon, forming **Pdt$_a$**, in which the nitrogen bridges a boron atom and the CAAC carbon (*via* N-B and N-C coordination). This transformation is highly exergonic, with a ΔG of -51.2 kcal/mol, indicating **Pdt$_a$** as the thermodynamically controlled product; and ii) Formation of **Pdt$_b$**, in which both nitrogen atoms bridge the two boron centers, is endergonic by 16.3 kcal/mol and proceeds *via* a free energy barrier of 47.8 kcal/mol.

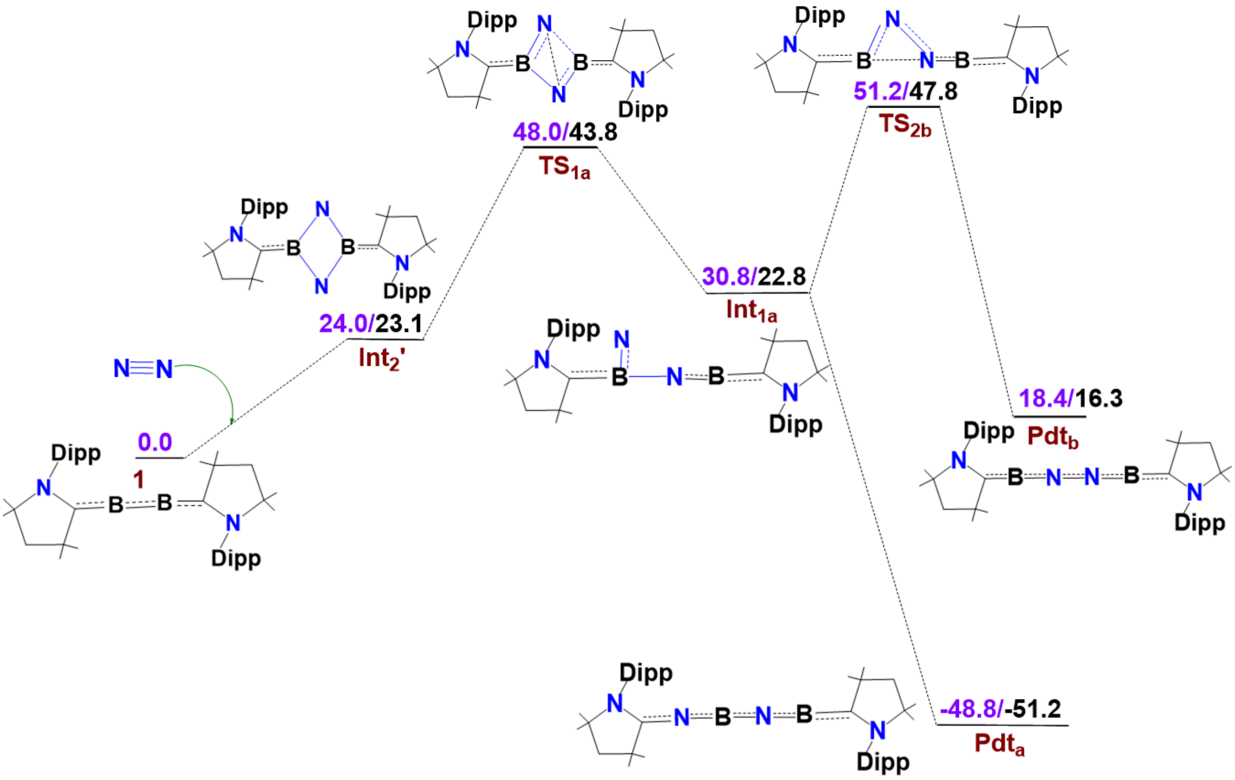

Figure S3. Free energy profile for N_2 activation by system **1** in the absence of LiCl at B3LYP-D3/def2-TZVP level of theory. Two values are in the gas phase (purple)/implicit toluene (black), respectively. Energy values are in kcal/mol.

S.3 Optimized Geometries of the Reactant Complex and Transition States in the Presence of an Oriented External Electric Field (OEEF) with $F_X = 0.012$

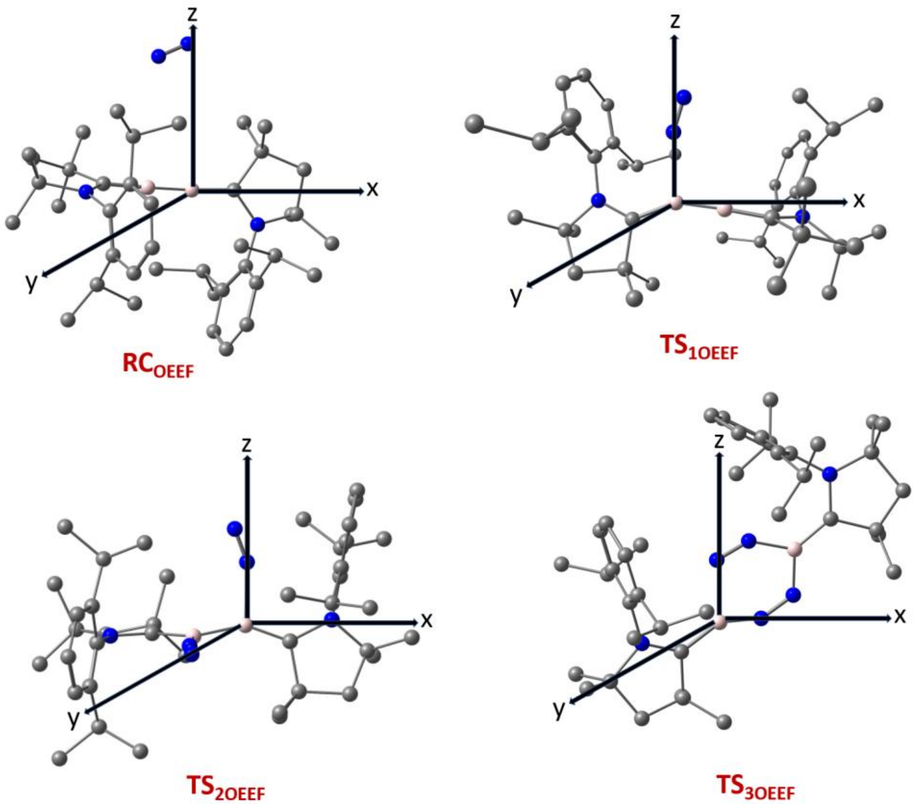

Figure S4. B3LYP-D3/def2-TZVP optimized geometries of the reactant complex (**RC$_{OEEF}$**) and transition states (**TS$_{1OEEF}$**, **TS$_{2OEEF}$**, **TS$_{3OEEF}$**) in the presence of an OEEF with $F_X = 0.012$ a.u. Free energy barriers for formation of **Int$_1$** calculated as G(**TS$_{1OEEF}$**)-G(**RC$_{OEEF}$**) and for the subsequent step calculated as G(**TS$_{2OEEF}$**)-G(**RC$_{OEEF}$**)-G(N_2).

S.4 Formation of the Reactant Complexes

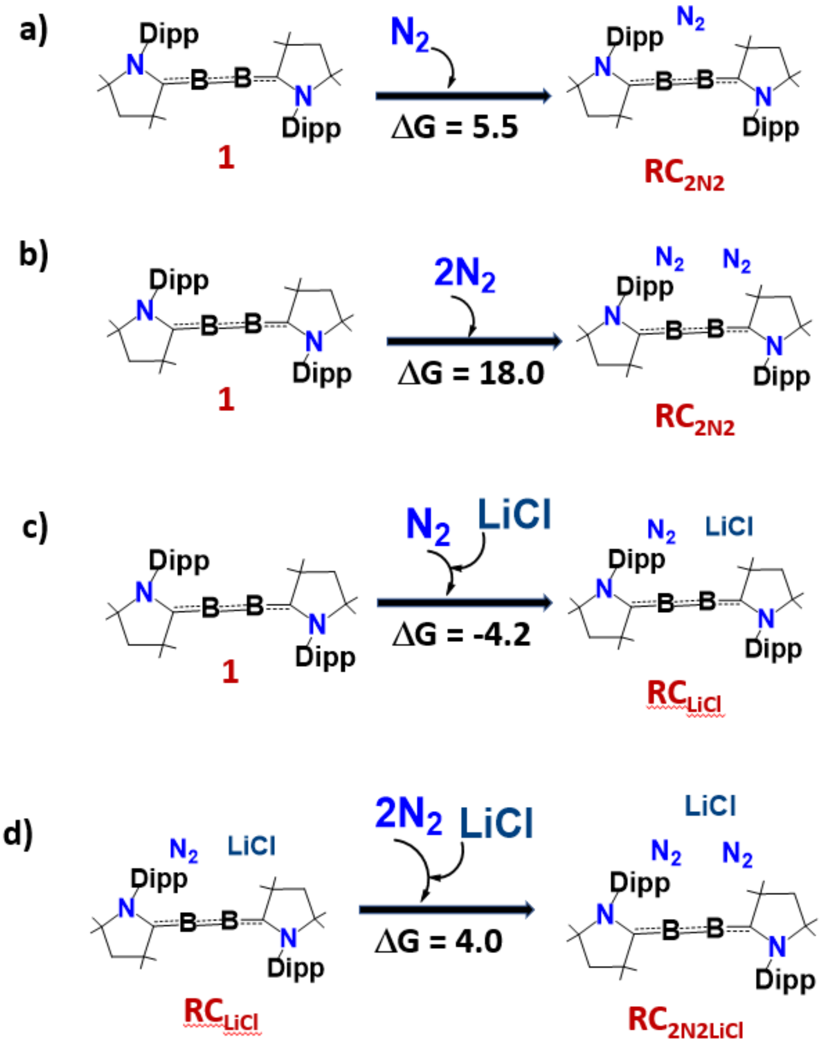

Figure S5. Free energy values (in kcal/mol) for the formation of the reactant complex with system **1** containing: (**a**) one N_2, (**b**) two N_2 molecules, (**c**) one N_2 and LiCl, and (**d**) two N_2 molecules and LiCl.

S.5 Energy Profiles (ΔE, kcal/mol) in Implicit Solvents

To obtain the energetics of the whole energy profile at B3LYP-D3/def2-TZVP single-point calculations in implicit THF were carried out. A comparison of the ΔE profiles for N_2 activation by system **1** in implicit toluene and implicit THF is presented in Figures S6 and S7 below. Our results indicate that the trend in $\Delta E^{\ddagger}$ values is similar in both solvents. For example, the energy barrier for the formation of **Int$_{1LiCl}$** is 9.8 kcal/mol in implicit toluene and 10.0 kcal/mol in implicit THF; for **Int$_{2LiCl}$**, 9.2 and 8.4 kcal/mol, respectively; and for **Pdt$_{LiCl}$**, 17.2 and 19.1 kcal/mol, respectively.

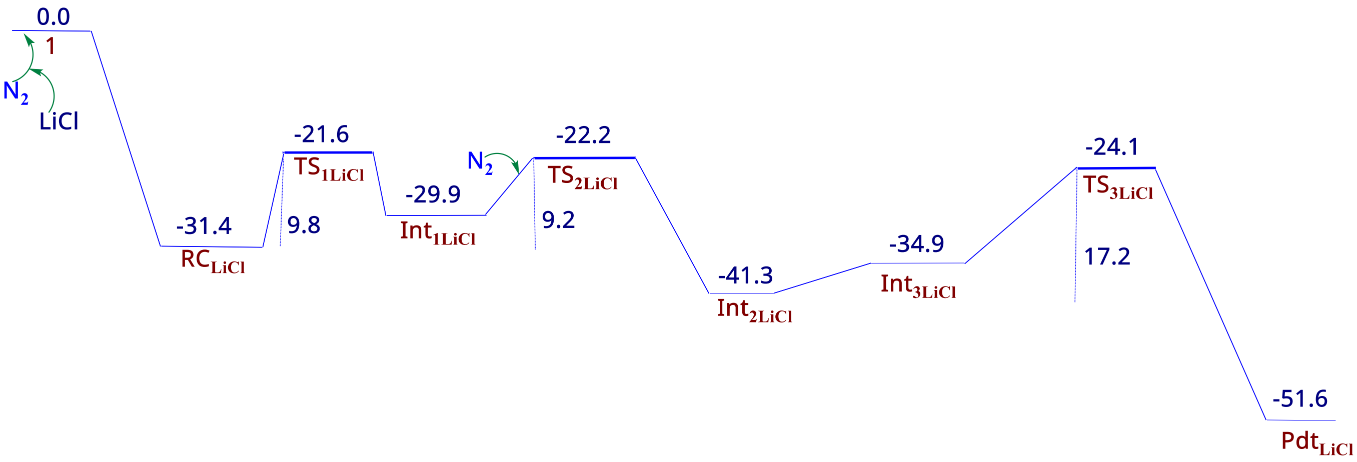

Figure S6. The energy profile (ΔE in kcal/mol) for the formation of the **Pdt$_{LiCl}$** in implicit toluene at the B3LYP-D3/def2-TZVP level of theory.

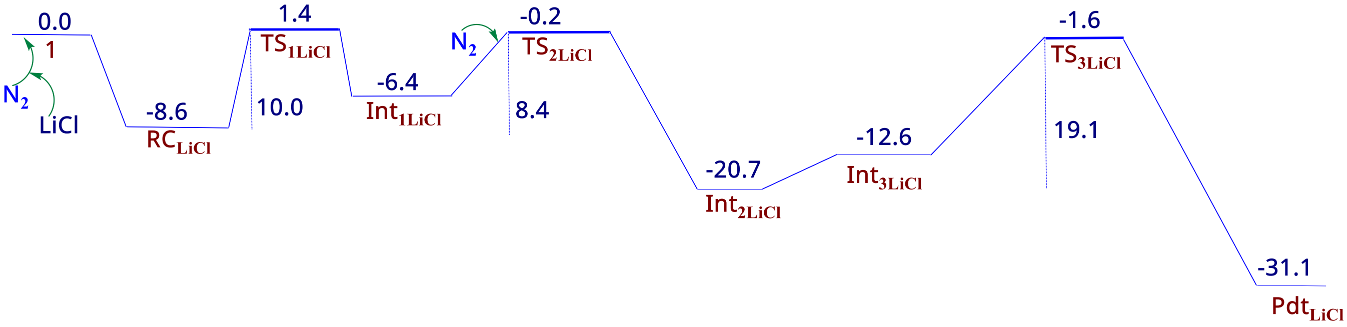

Figure S7. The energy profile (ΔE in kcal/mol) for the formation of the **Pdt$_{LiCl}$** at B3LYP-D3/def2-TZVP (**THF**)//B3LYP-D3/def2-TZVP (**Gas-Phase**) level of theory.

S.6 Effect of THF-solvated LiCl on N_2 activation by 1

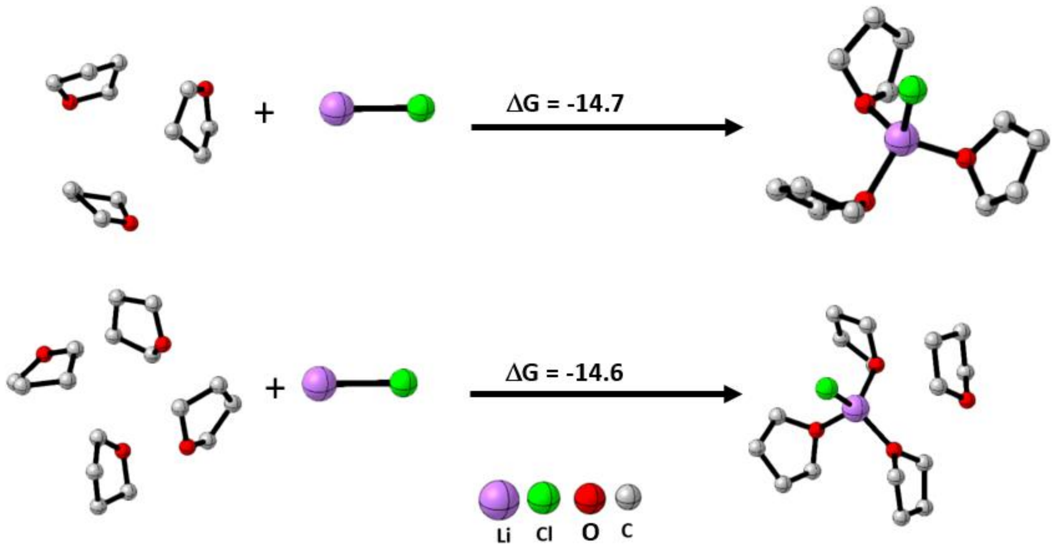

Figure S8. Free energy values for THF coordination to LiCl: LiCl[THF]$_3$ and LiCl[THF]$_4$ computed at PBE/TZVP/D3/COSMO(THF). ΔG values reported here are in kcal/mol.

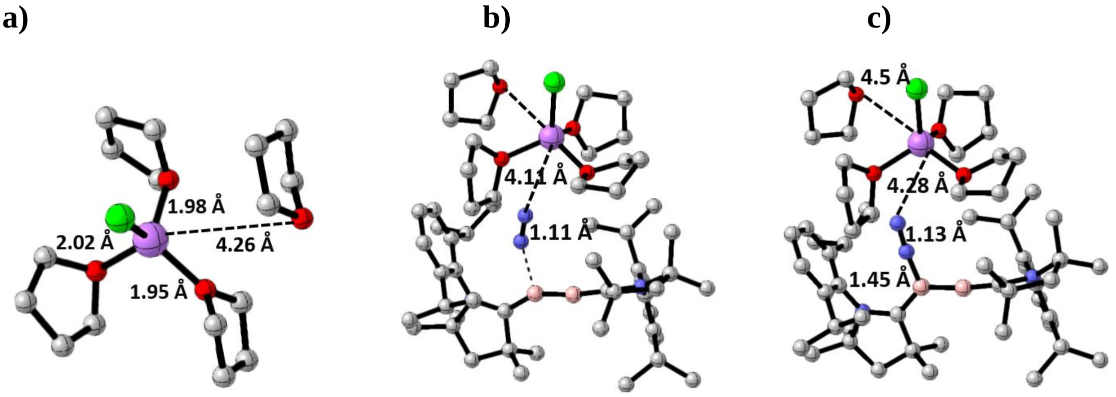

Figure S9. Comparison of key bond distances (Å) in **a)** LiCl[THF]$_4$, **b)** transition states **TS$_{1LiCl[THF]4}$**, and **c) Int$_{1LiCl[THF]4}$** optimized at the PBE/TZVP/D3/COSMO(THF) level of theory, in the presence of LiCl[THF]$_4$, highlighting it's effect on N$\equiv$N.

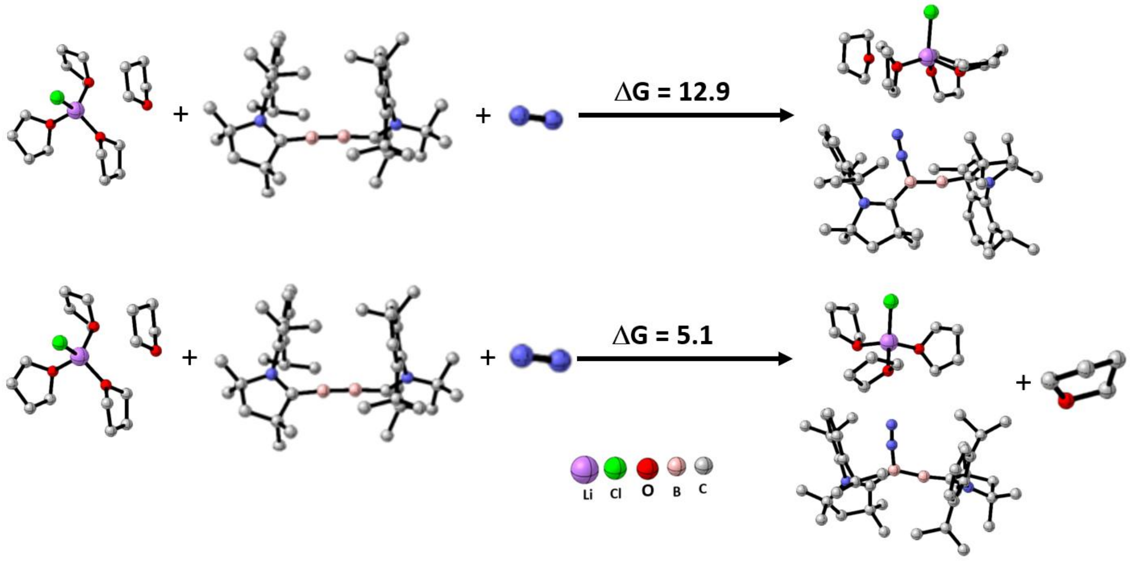

Figure S10. Free energy values for the formation of **Int$_1$** in the presence of explicit LiCl[THF]$_4$ and LiCl[THF]$_3$ computed at PBE/TZVP/D3/COSMO(THF). ΔG values reported here are in kcal/mol.

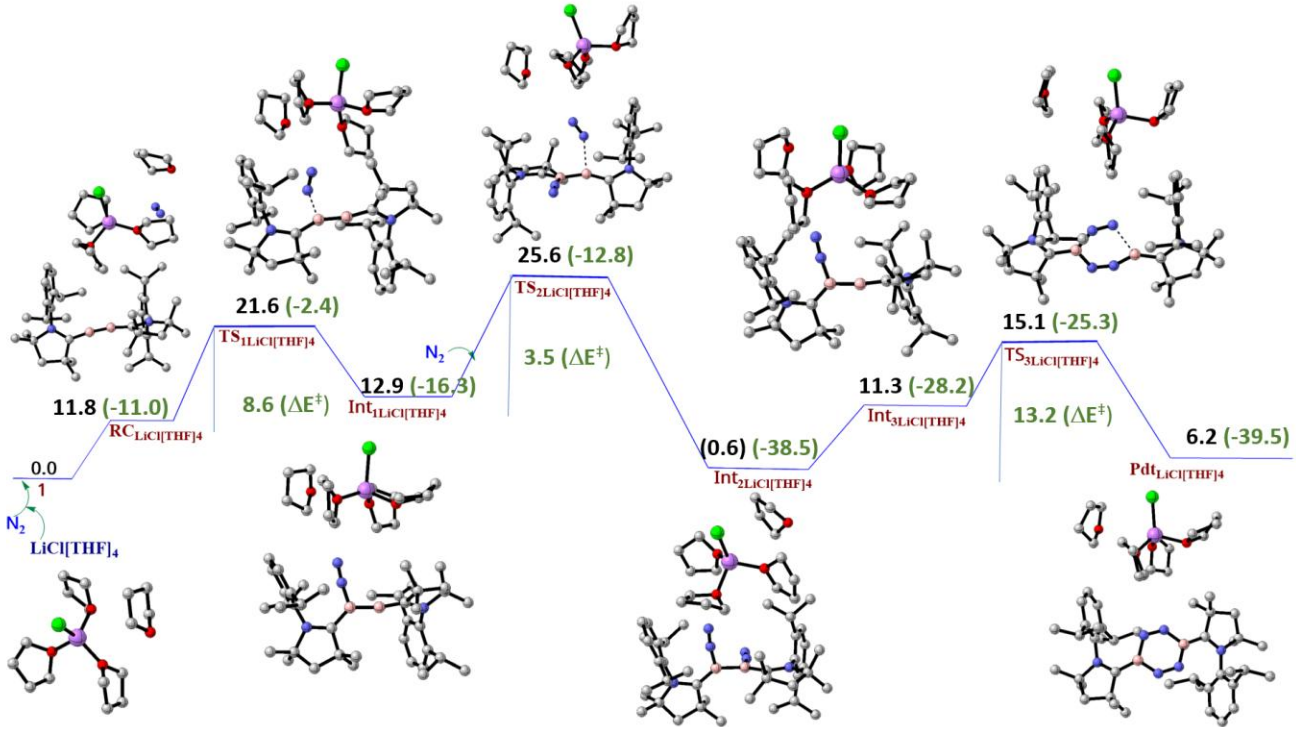

Figure S11. Free energy profile calculated at the PBE/TZVP/D3/COSMO(THF) level of theory in the presence of explicit LiCl[THF]$_4$. ΔG values are shown in black, and ΔE values in green; all energies are reported in kcal/mol.

S.7 Effect of KCl and Ionic Liquids (ILs) additive on N_2 binding to 1

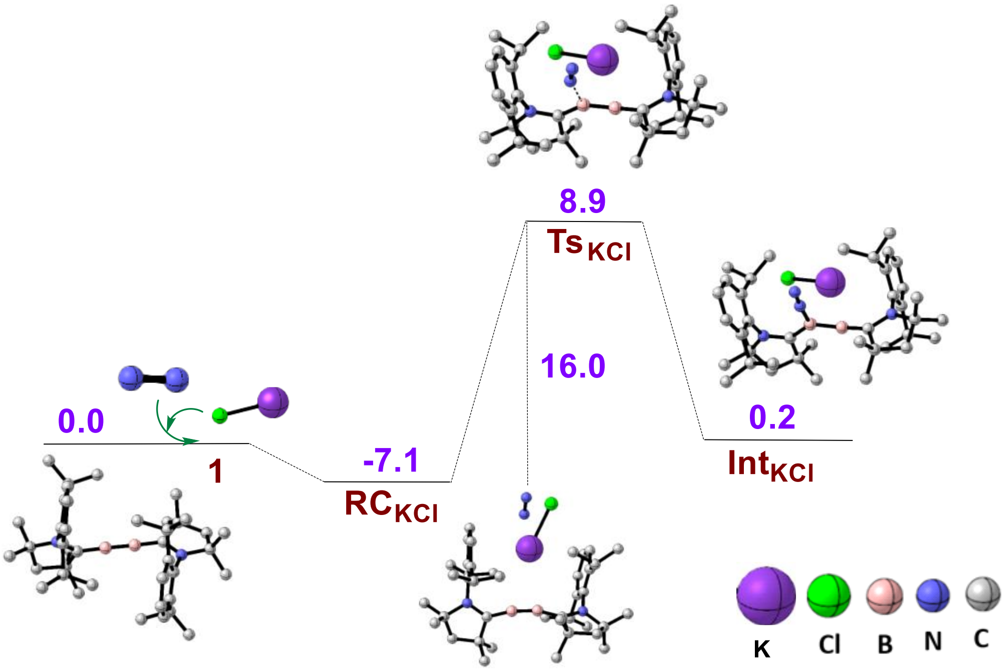

Figure S12. Free energy profile for formation of **Int$_{KCl}$** at B3LYP-D3/def2-TZVP in the gas phase. ΔG values reported here are in kcal/mol.

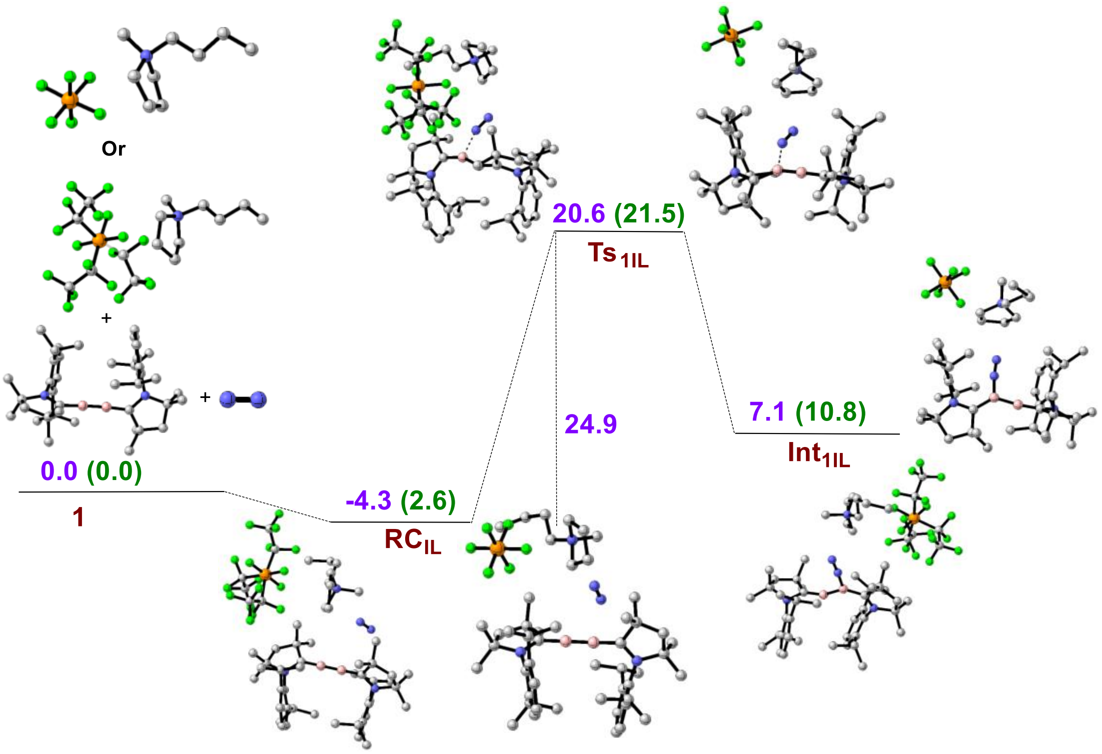

Figure S13. Free energy profile for N_2 binding to system **1** in the presence of explicit ILs: (i) $[C_4mpyr]^+[eFAP]^-$ (purple) and (ii) $[C_4mpyr]^+[PF_6]^-$ (green), computed at the B3LYP-D3/def2-TZVP level of theory. ΔG values reported here are in kcal/mol.

S.8 XYZ coordinates of B3LYP-D3/def2-TZVP optimized geometries in the gas and implicit toluene phases, and PBE/TZVP/D3 optimized geometries in the hybrid solvation model.

In Gas Phase

System (**1**)

106

C	-3.943620	-1.745306	-2.183960
C	-4.419437	-0.852840	-1.022303
C	-2.070972	-0.475269	-1.380033
C	-2.554932	-1.225926	-2.622799
H	-4.659396	-1.754846	-3.007270
H	-3.845565	-2.771240	-1.823780
N	-3.113980	-0.334524	-0.514056
C	-5.333547	0.287103	-1.498152
H	-6.248314	-0.128669	-1.925524
H	-5.613970	0.926612	-0.661978
H	-4.855918	0.904087	-2.256110
C	-2.648055	-0.263091	-3.821569
H	-1.656182	0.109796	-4.078489
H	-3.066500	-0.780800	-4.689473
H	-3.277672	0.598795	-3.602979
C	-1.618569	-2.383081	-2.990611
H	-2.026722	-2.945741	-3.835066
H	-0.633773	-2.008073	-3.270387
H	-1.488740	-3.066142	-2.149634
C	-5.175697	-1.655538	0.037917
H	-5.398531	-1.043616	0.913248
H	-6.121456	-2.003644	-0.381065
H	-4.610887	-2.527060	0.360217
C	-2.940639	0.231860	0.791485
C	-3.087682	1.617176	0.989629
C	-2.586382	-0.619190	1.859477
C	-2.994930	2.112990	2.290887
C	-2.504045	-0.073506	3.138660
C	-2.729919	1.275570	3.360956
H	-3.110843	3.175451	2.463701
H	-2.236648	-0.710956	3.971508
H	-2.663083	1.679487	4.363783
C	-3.235486	2.597571	-0.162514
H	-3.345161	2.022736	-1.078980
C	-2.202908	-2.076953	1.653417
H	-2.447032	-2.346783	0.627371
C	-1.949901	3.428881	-0.311463
H	-2.019274	4.079995	-1.186686
H	-1.783830	4.058997	0.566082
H	-1.088041	2.773514	-0.436001
C	-4.457756	3.513690	-0.019812
H	-4.572421	4.132673	-0.912731
H	-5.378258	2.945486	0.119137
H	-4.352808	4.186480	0.833849
C	-0.684196	-2.253915	1.813227
H	-0.139411	-1.607316	1.126476
H	-0.366746	-2.014778	2.830004
H	-0.396983	-3.287395	1.605824
C	-2.956629	-3.032093	2.589733
H	-4.037013	-2.891741	2.531862
H	-2.731103	-4.068685	2.328691
H	-2.659760	-2.887702	3.630600
B	0.688250	0.483436	-1.110333
B	-0.718029	0.011870	-1.202155
C	2.044275	0.990897	-1.107841
C	2.500900	2.232661	-1.874465
C	4.034629	2.044690	-1.914740
C	4.420537	1.139583	-0.728111

H	4.567207	2.995882	-1.884237
H	4.307950	1.547280	-2.847526
N	3.112602	0.480462	-0.430005
C	2.966381	-0.557268	0.549801
C	2.652526	-0.214607	1.881995
C	3.105039	-1.905539	0.167457
C	2.615672	-1.231468	2.834668
C	3.058073	-2.884985	1.159779
C	2.842784	-2.553061	2.486663
H	2.380656	-0.986449	3.862677
H	3.169853	-3.925862	0.883901
H	2.812039	-3.327575	3.243213
C	1.906234	2.304006	-3.284331
H	2.141336	1.402467	-3.851890
H	0.819866	2.394533	-3.239699
H	2.305555	3.169486	-3.820646
C	2.101378	3.504338	-1.100188
H	1.016585	3.594987	-1.054009
H	2.475961	3.495068	-0.077529
H	2.503664	4.389731	-1.600407
C	5.489354	0.118843	-1.130076
H	6.418714	0.639631	-1.369505
H	5.692708	-0.577681	-0.315636
H	5.186224	-0.450338	-2.006887
C	4.954313	1.933100	0.472885
H	5.111811	1.273654	1.326377
H	5.914388	2.384143	0.214571
H	4.279488	2.730611	0.774878
C	3.187790	-2.338126	-1.287547
H	3.267118	-1.440763	-1.898464
C	2.249660	1.192270	2.295023
H	2.430038	1.852091	1.451564
C	0.741638	1.246444	2.585040
H	0.434521	2.268926	2.816250
H	0.164895	0.908210	1.725257
H	0.481592	0.617688	3.438730
C	3.047526	1.724185	3.492887
H	4.123180	1.678538	3.319905
H	2.778874	2.764936	3.688825
H	2.831878	1.156355	4.400329
C	1.880918	-3.038260	-1.695349

H	1.748209	-3.973210	-1.144872
H	1.024536	-2.395240	-1.495838
H	1.893881	-3.274890	-2.762326
C	4.396407	-3.236175	-1.584228
H	4.452347	-3.450330	-2.654183
H	5.333981	-2.769971	-1.281076
H	4.317733	-4.192840	-1.063840

N_2

2

N	0.000000	0.000000	0.164470
N	0.000000	0.000000	1.255530

RC

108

C	-3.966030	-1.642910	-2.267031
C	-4.440287	-0.793600	-1.072926
C	-2.091359	-0.409854	-1.410172
C	-2.570936	-1.117313	-2.679489
H	-4.677601	-1.614121	-3.093545
H	-3.877451	-2.683044	-1.947214
N	-3.133353	-0.308830	-0.537904
C	-5.337839	0.374588	-1.509668
H	-6.253836	-0.013410	-1.959765
H	-5.617341	0.984122	-0.650986
H	-4.846344	1.014968	-2.239154
C	-2.645302	-0.120579	-3.850992
H	-1.649126	0.256384	-4.083868
H	-3.051735	-0.612950	-4.739013
H	-3.277301	0.735504	-3.616636
C	-1.638919	-2.270438	-3.072019
H	-2.046458	-2.808716	-3.932525
H	-0.651874	-1.893847	-3.338978
H	-1.516696	-2.975421	-2.248124
C	-5.210214	-1.628158	-0.048047
H	-5.433102	-1.046641	0.847769
H	-6.156305	-1.951984	-0.485370
H	-4.654625	-2.515625	0.245909
C	-2.958589	0.192643	0.793813
C	-3.101739	1.566546	1.062111
C	-2.606999	-0.712521	1.817427
C	-3.010655	1.994782	2.387545
C	-2.525514	-0.233184	3.123076

C	-2.750019	1.103161	3.413799
H	-3.123261	3.047544	2.613732
H	-2.259673	-0.912897	3.922349
H	-2.684125	1.454913	4.436228
C	-3.240368	2.606446	-0.038197
H	-3.346636	2.080877	-0.984352
C	-2.225483	-2.158607	1.536963
H	-2.478745	-2.378406	0.501069
C	-1.950803	3.439109	-0.134867
H	-2.010743	4.135517	-0.975321
H	-1.788506	4.021572	0.775702
H	-1.091299	2.786779	-0.287420
C	-4.459716	3.519570	0.145722
H	-4.565298	4.185733	-0.713844
H	-5.383966	2.949516	0.248109
H	-4.357053	4.145211	1.034806
C	-0.705417	-2.342064	1.675741
H	-0.166200	-1.665096	1.014170
H	-0.379664	-2.147472	2.699380
H	-0.419900	-3.365391	1.421120
C	-2.971368	-3.158810	2.431532
H	-4.051984	-3.012939	2.392927
H	-2.751833	-4.181022	2.114743
H	-2.662073	-3.068528	3.474805
B	0.654783	0.562722	-1.076330
B	-0.743286	0.078234	-1.206705
C	2.005199	1.078992	-1.029574
C	2.460852	2.378940	-1.693786
C	3.991707	2.181579	-1.769032
C	4.383434	1.189935	-0.654638
H	4.534125	3.123519	-1.678086
H	4.247954	1.749009	-2.738119
N	3.072419	0.525280	-0.383375
C	2.925125	-0.559471	0.544875
C	2.617399	-0.278345	1.893323
C	3.059067	-1.889605	0.102212
C	2.582641	-1.337204	2.799208
C	3.018375	-2.912657	1.050307
C	2.809283	-2.641676	2.391497
H	2.350169	-1.138593	3.837788
H	3.127909	-3.939803	0.726377
H	2.781521	-3.449828	3.112408
C	1.845325	2.585289	-3.080297
H	2.086400	1.754629	-3.742951
H	0.758346	2.652396	-3.012442
H	2.222862	3.508188	-3.529101
C	2.083699	3.580934	-0.804152
H	1.000559	3.676289	-0.734712
H	2.472214	3.480231	0.207915
H	2.487526	4.502913	-1.231428
C	5.430642	0.187093	-1.147193
H	6.360123	0.712935	-1.375393
H	5.645012	-0.561739	-0.383678
H	5.099127	-0.321288	-2.050918
C	4.946573	1.888198	0.591415
H	5.099369	1.168091	1.395579
H	5.912931	2.336310	0.352534
H	4.292443	2.677042	0.956140
C	3.124078	-2.261363	-1.370315
H	3.188572	-1.339563	-1.944735
C	2.218351	1.108254	2.372442
H	2.404645	1.807093	1.562771
C	0.709149	1.152428	2.658153
H	0.403925	2.161947	2.942669
H	0.135394	0.862655	1.778663
H	0.443543	0.478859	3.475096
C	3.014218	1.579797	3.596443
H	4.090145	1.538663	3.423298
H	2.748472	2.611158	3.840218
H	2.794148	0.970607	4.475580
C	1.815868	-2.955735	-1.782716
H	1.689352	-3.901700	-1.250395
H	0.959835	-2.318910	-1.562754
H	1.820598	-3.173057	-2.853133
C	4.332346	-3.138271	-1.723987
H	4.372946	-3.302392	-2.803339
H	5.272063	-2.680928	-1.414422
H	4.265112	-4.117567	-1.245654
N	1.484064	-0.776417	-4.871669
N	2.516163	-1.026580	-4.622602

TS_1

108

C	-4.373160	2.615386	-0.575170
C	-4.245733	1.551682	-1.686087
C	-2.212376	1.543046	-0.432667
C	-2.958218	2.810122	0.029832
H	-5.055694	2.246001	0.193062
H	-4.787782	3.551205	-0.952664
N	-3.090040	0.764510	-1.188951
C	-5.507847	0.704803	-1.832963
H	-5.803790	0.249439	-0.889341
H	-6.329547	1.335125	-2.177898
H	-5.362866	-0.092562	-2.563970
C	-3.021155	2.884323	1.565026
H	-3.612144	3.748605	1.884035
H	-3.472751	1.986641	1.986446
H	-2.018055	2.981864	1.985094
C	-2.288951	4.094286	-0.486399
H	-1.293390	4.200974	-0.054126
H	-2.181024	4.087748	-1.570378
H	-2.878614	4.971667	-0.204707
C	-3.932244	2.212706	-3.042334
H	-3.890320	1.471598	-3.838836
H	-4.708536	2.937289	-3.299029
H	-2.975061	2.731675	-3.012210
C	-2.968577	-0.656432	-1.273647
C	-3.466205	-1.451739	-0.214664
C	-2.350906	-1.264711	-2.381623
C	-3.348478	-2.836764	-0.302111
C	-2.285188	-2.658395	-2.438220
C	-2.775647	-3.442747	-1.410073
H	-3.716525	-3.454665	0.507143
H	-1.822997	-3.134237	-3.294286
H	-2.704096	-4.522303	-1.464758
C	-4.097472	-0.846008	1.027899
H	-4.311903	0.197332	0.809632
C	-1.715256	-0.460250	-3.500390
H	-1.785258	0.591505	-3.225672
C	-3.110410	-0.865158	2.202782
H	-2.219552	-0.292921	1.950905
H	-3.561801	-0.423253	3.094835
H	-2.810339	-1.887098	2.445537
C	-5.423522	-1.515283	1.414636

H	-6.115534	-1.555866	0.571125
H	-5.274836	-2.536940	1.770048
H	-5.901475	-0.956426	2.222535
C	-0.223895	-0.797034	-3.643866
H	0.238217	-0.155085	-4.396811
H	0.295722	-0.646507	-2.698536
H	-0.076417	-1.833494	-3.955398
C	-2.443311	-0.668149	-4.836160
H	-2.331584	-1.697634	-5.185519
H	-3.511956	-0.467890	-4.747581
H	-2.034123	-0.008509	-5.605500
B	0.639954	0.979147	0.137325
B	-0.843071	1.256511	-0.145792
C	2.017097	1.162731	-0.340661
C	2.310964	2.268042	-1.376608
C	3.856325	2.286435	-1.428744
C	4.344839	0.929097	-0.913189
H	4.228993	3.071802	-0.767806
H	4.228567	2.499185	-2.431374
N	3.184885	0.528258	-0.048164
C	3.285884	-0.512795	0.931699
C	3.662196	-0.160485	2.244864
C	2.954549	-1.842903	0.600802
C	3.771147	-1.172376	3.195810
C	3.086232	-2.816048	1.591613
C	3.499630	-2.492455	2.872889
H	4.052887	-0.922039	4.210560
H	2.835929	-3.843464	1.360077
H	3.588326	-3.265321	3.626534
C	1.693063	1.951156	-2.747137
H	1.946789	2.742084	-3.458365
H	2.053771	1.007053	-3.149285
H	0.608770	1.889275	-2.661777
C	1.785814	3.634463	-0.909490
H	2.123719	3.858630	0.103815
H	2.150229	4.419433	-1.579053
H	0.697980	3.646693	-0.913930
C	4.567188	-0.079481	-2.050992
H	5.362021	0.280535	-2.706929
H	4.873902	-1.045303	-1.653087
H	3.672193	-0.223174	-2.652288

C	5.653644	1.047423	-0.130990
H	5.916740	0.103760	0.349173
H	6.457007	1.308265	-0.821598
H	5.604821	1.822618	0.629956
C	2.381815	-2.257647	-0.747409
H	2.368979	-1.383166	-1.392442
C	3.866586	1.283624	2.683310
H	3.864694	1.907541	1.792286
C	2.700037	1.769202	3.560638
H	2.588693	1.144623	4.449804
H	2.880953	2.795431	3.889501
H	1.760329	1.755044	3.010097
C	5.204031	1.491971	3.408715
H	5.213911	0.991132	4.378583
H	6.043201	1.106836	2.828044
H	5.372633	2.556103	3.589683
C	0.925000	-2.737005	-0.620730
H	0.841757	-3.583743	0.063700
H	0.268249	-1.943376	-0.269195
H	0.550858	-3.056491	-1.593799
C	3.228821	-3.346017	-1.425819
H	3.163618	-4.291527	-0.883260
H	2.867934	-3.524005	-2.441295
H	4.282718	-3.071993	-1.482492
N	0.231476	-0.781773	2.565605
N	0.624588	-0.239008	1.693933

Int$_1$

108

C	-4.569084	2.205471	0.455975
C	-4.568341	1.043278	-0.562015
C	-2.330587	1.325781	0.233816
C	-3.086491	2.579390	0.722329
H	-5.034274	1.862228	1.382616
H	-5.147536	3.060741	0.102140
N	-3.276675	0.394427	-0.249192
C	-5.752061	0.098987	-0.364937
H	-5.800009	-0.276942	0.655524
H	-6.682270	0.628800	-0.578720
H	-5.688051	-0.757633	-1.038407
C	-2.845618	2.837086	2.219891
H	-3.440895	3.687487	2.568217
H	-3.110035	1.966771	2.819757
H	-1.792926	3.061019	2.405843
C	-2.680307	3.847415	-0.047525
H	-1.642313	4.105474	0.165900
H	-2.774305	3.716858	-1.124890
H	-3.304511	4.694561	0.252792
C	-4.599473	1.587820	-2.004698
H	-4.674127	0.777537	-2.728472
H	-5.460905	2.244575	-2.147848
H	-3.696077	2.155804	-2.224269
C	-3.049217	-1.009938	-0.166494
C	-3.195844	-1.673947	1.075102
C	-2.654418	-1.739166	-1.305699
C	-2.952301	-3.043960	1.141220
C	-2.457998	-3.116954	-1.198196
C	-2.598064	-3.769261	0.013537
H	-3.052826	-3.556674	2.089752
H	-2.169233	-3.683446	-2.075130
H	-2.425562	-4.836543	0.083270
C	-3.605855	-0.943492	2.341842
H	-3.929977	0.051681	2.049679
C	-2.401534	-1.068231	-2.643003
H	-2.485572	0.005771	-2.483274
C	-2.414758	-0.773073	3.294459
H	-1.627334	-0.195746	2.813978
H	-2.720495	-0.246068	4.202225
H	-2.004818	-1.742076	3.588693
C	-4.783835	-1.618600	3.058952
H	-5.622014	-1.790776	2.381377
H	-4.499216	-2.582982	3.484767
H	-5.132817	-0.988973	3.881168
C	-0.979390	-1.347444	-3.150445
H	-0.778350	-0.768494	-4.054909
H	-0.241854	-1.074322	-2.396941
H	-0.839689	-2.402832	-3.395619
C	-3.438180	-1.490069	-3.694516
H	-3.346547	-2.554208	-3.925705
H	-4.456963	-1.318642	-3.344254
H	-3.298170	-0.931327	-4.623534
B	0.629583	0.816999	0.363557
B	-0.923633	1.152235	0.232383

C	1.780592	1.137617	-0.561036
C	1.675946	2.168871	-1.688962
C	3.140875	2.323517	-2.161557
C	3.896634	1.070713	-1.704853
H	3.583126	3.200690	-1.685305
H	3.210391	2.467702	-3.239998
N	3.022643	0.622084	-0.563562
C	3.468245	-0.330144	0.417628
C	4.087828	0.157320	1.586409
C	3.213804	-1.703898	0.240426
C	4.520966	-0.766679	2.534667
C	3.674786	-2.583277	1.220598
C	4.333124	-2.126951	2.349607
H	4.992858	-0.415196	3.442940
H	3.489220	-3.643558	1.108175
H	4.675149	-2.829354	3.099621
C	0.756090	1.674956	-2.818024
H	0.721637	2.422897	-3.614828
H	1.106657	0.739249	-3.248982
H	-0.252877	1.519049	-2.438431
C	1.146025	3.514379	-1.171971
H	1.710640	3.852769	-0.300849
H	1.237220	4.269991	-1.957616
H	0.100007	3.429523	-0.889201
C	3.963389	-0.002282	-2.799968
H	4.546324	0.378447	-3.640423
H	4.454394	-0.900589	-2.430554
H	2.976785	-0.274093	-3.168911
C	5.325024	1.384673	-1.260671
H	5.803504	0.512750	-0.813009
H	5.909823	1.679117	-2.133749
H	5.358429	2.205241	-0.547960
C	2.390489	-2.271729	-0.907851
H	2.128802	-1.454845	-1.575713
C	4.224286	1.642267	1.897228
H	3.930655	2.202995	1.011292
C	3.265383	2.064602	3.023482
H	3.469457	1.511522	3.942802
H	3.385798	3.129427	3.236741
H	2.226594	1.892695	2.746057
C	5.666561	2.035224	2.251085
H	5.974528	1.601122	3.204300
H	6.375485	1.703065	1.491763
H	5.748372	3.120324	2.345797
C	1.068514	-2.878328	-0.407228
H	1.245325	-3.680131	0.311761
H	0.430652	-2.133006	0.063187
H	0.510626	-3.297388	-1.245077
C	3.169279	-3.321474	-1.715881
H	3.339787	-4.224223	-1.125753
H	2.600297	-3.611064	-2.602111
H	4.142239	-2.952939	-2.041553
N	0.933629	-0.648744	2.462899
N	0.872701	-0.028783	1.534860

Int$_1$'

108

C	4.575522	-1.630164	2.482357
C	4.841622	-0.273846	2.552042
C	4.282562	0.624538	1.644228
C	3.453809	0.108214	0.625397
C	3.115016	-1.261499	0.580073
C	3.705553	-2.107821	1.518396
N	2.945838	0.999653	-0.399034
C	1.686848	1.429953	-0.474586
C	1.492530	2.197624	-1.783473
C	2.933448	2.370727	-2.305184
C	3.777587	1.281842	-1.639852
C	0.620734	1.412015	-2.780649
C	0.841457	3.563088	-1.524434
C	3.901860	0.012004	-2.489245
C	5.184821	1.774101	-1.323267
C	4.544023	2.111230	1.864873
C	6.044084	2.453442	1.886784
C	2.091591	-1.869398	-0.371559
C	2.675289	-3.002649	-1.230053
C	-2.431743	1.095927	0.045975
C	-3.301804	2.329526	0.393419
C	-4.738795	1.769981	0.248042
C	-4.652478	0.528531	-0.660820
N	-3.283543	0.044970	-0.358724
C	-5.722345	-0.505736	-0.315410
C	-4.808048	0.920892	-2.143791

C	-3.048834	2.812802	1.832509
C	-3.060107	3.516923	-0.554617
C	-2.947272	-1.329343	-0.189450
C	-2.992490	-1.918795	1.096564
C	-2.729374	-3.280896	1.224646
C	-2.443646	-4.069097	0.120181
C	-2.372453	-3.484381	-1.131585
C	-2.591401	-2.115975	-1.304000
C	-3.309654	-1.116842	2.346182
C	-4.444002	-1.731132	3.179147
C	-2.378472	-1.515197	-2.681220
C	-3.359328	-2.073025	-3.722246
C	-2.051302	-0.930745	3.205586
C	-0.930002	-1.730893	-3.147703
C	0.867348	-2.393731	0.398858
C	3.904546	2.601483	3.176633
H	-5.109423	1.461714	1.227926
H	-5.431407	2.519085	-0.140110
H	-5.684327	-0.782717	0.736277
H	-6.710360	-0.091613	-0.525307
H	-5.602703	-1.413198	-0.909785
H	-3.729073	3.630588	2.092156
H	-3.193549	2.004621	2.549905
H	-2.024712	3.175345	1.945505
H	-2.060566	3.925897	-0.403787
H	-3.145717	3.230032	-1.601551
H	-3.780258	4.316973	-0.357265
H	-4.834832	0.038190	-2.779872
H	-5.739176	1.471516	-2.298346
H	-3.981324	1.548774	-2.472011
H	-2.762441	-3.737795	2.206020
H	-2.119362	-4.096006	-1.989319
H	-2.261859	-5.130477	0.239203
H	-3.632840	-0.130318	2.025098
H	-2.538124	-0.441780	-2.592250
H	-1.260292	-0.454750	2.630091
H	-2.267288	-0.300956	4.072504
H	-1.681375	-1.892302	3.571152
H	-5.337971	-1.909281	2.579256
H	-4.148603	-2.684498	3.622405
H	-4.712180	-1.059539	3.998319

H	-0.226082	-1.375005	-2.396955
H	-0.722513	-2.788370	-3.327026
H	-0.741449	-1.190869	-4.078790
H	-4.394966	-1.967651	-3.396806
H	-3.247102	-1.551905	-4.676486
H	-3.179365	-3.136040	-3.899867
H	3.312607	3.350082	-2.006452
H	2.989122	2.315962	-3.392511
H	5.484113	0.100217	3.338313
H	3.462530	-3.161983	1.500711
H	5.022989	-2.310025	3.196958
H	0.530032	1.987298	-3.705782
H	1.052061	0.445005	-3.030173
H	-0.373649	1.247754	-2.367728
H	-0.187396	3.440091	-1.195843
H	1.383303	4.120667	-0.758163
H	0.845053	4.149895	-2.446509
H	4.448106	0.246472	-3.404216
H	4.459771	-0.752387	-1.950399
H	2.935425	-0.399064	-2.767671
H	5.752989	1.027422	-0.769178
H	5.704771	1.959292	-2.264860
H	5.174257	2.704011	-0.760610
H	1.735351	-1.089779	-1.041414
H	4.072621	2.664174	1.052715
H	2.829533	2.441089	3.191760
H	4.337698	2.085827	4.036348
H	4.090364	3.671186	3.301431
H	6.180731	3.537029	1.912666
H	6.521252	2.045347	2.779829
H	6.578194	2.063546	1.022325
H	1.145275	-3.216496	1.060565
H	0.406462	-1.615661	0.999493
H	0.118588	-2.768298	-0.297790
H	3.564063	-2.696588	-1.780715
H	2.946634	-3.862111	-0.613825
H	1.928143	-3.340433	-1.951300
N	0.923643	1.729755	2.081798
N	1.009349	0.567496	2.003112
B	0.537902	1.222247	0.532469
B	-1.025188	1.129347	0.181937

Int$_2$'

108

C	-4.433066	-1.226566	-2.607975
C	-4.723307	-0.950610	-1.119347
C	-2.514366	-0.177263	-1.644714
C	-3.172632	-0.414165	-2.989822
H	-5.290094	-0.980469	-3.235972
H	-4.227608	-2.290970	-2.739720
N	-3.384247	-0.476864	-0.645198
C	-5.798219	0.127730	-0.920298
H	-6.756623	-0.240943	-1.290315
H	-5.911701	0.360548	0.138181
H	-5.562675	1.046860	-1.451882
C	-3.524298	0.937240	-3.646435
H	-2.616329	1.512149	-3.831632
H	-4.035984	0.771799	-4.599168
H	-4.175064	1.538320	-3.012103
C	-2.267229	-1.205779	-3.943857
H	-2.794759	-1.420282	-4.877671
H	-1.365744	-0.639050	-4.180127
H	-1.960672	-2.152069	-3.494522
C	-5.182516	-2.213324	-0.386781
H	-5.274367	-2.033507	0.685309
H	-6.162191	-2.511984	-0.764097
H	-4.495116	-3.041786	-0.543931
C	-3.082798	-0.211350	0.734495
C	-3.246624	1.088015	1.257127
C	-2.565852	-1.251541	1.529364
C	-2.992232	1.286190	2.613817
C	-2.325477	-0.998622	2.879531
C	-2.556082	0.252392	3.425790
H	-3.113185	2.275733	3.035312
H	-1.926744	-1.787133	3.504880
H	-2.362304	0.431839	4.476312
C	-3.596460	2.296126	0.401880
H	-3.787474	1.949804	-0.610683
C	-2.149948	-2.593466	0.949533
H	-2.466411	-2.622998	-0.091554
C	-2.402573	3.263487	0.322258
H	-2.630376	4.080956	-0.365891
H	-2.188590	3.699019	1.301485
H	-1.505821	2.751844	-0.023562
C	-4.847705	3.035173	0.897590
H	-5.102023	3.845179	0.209925
H	-5.710949	2.374131	0.976311
H	-4.680754	3.481132	1.880395
C	-0.619454	-2.707854	0.956581
H	-0.157958	-1.871422	0.434003
H	-0.230653	-2.713484	1.975356
H	-0.304705	-3.635364	0.472406
C	-2.780390	-3.787273	1.679257
H	-3.867558	-3.716585	1.714345
H	-2.515006	-4.719447	1.174638
H	-2.418729	-3.858985	2.707113
B	1.006860	0.836063	-1.296969
B	-1.119308	0.299681	-1.468930
C	2.471049	1.056814	-1.300564
C	3.239694	1.660454	-2.465668
C	4.715294	1.439208	-2.049447
C	4.724977	1.211249	-0.524863
H	5.355995	2.273491	-2.337002
H	5.101422	0.545674	-2.543928
N	3.332695	0.710039	-0.304603
C	2.966195	-0.191043	0.749055
C	2.529047	0.292987	1.993013
C	3.038950	-1.577871	0.489689
C	2.261666	-0.634085	3.003259
C	2.758820	-2.458386	1.530327
C	2.393310	-1.993390	2.785106
H	1.928564	-0.280536	3.970608
H	2.807474	-3.524973	1.354489
H	2.181272	-2.695219	3.582567
C	2.922238	0.925354	-3.779076
H	3.112230	-0.145169	-3.683784
H	1.875703	1.059588	-4.053416
H	3.545253	1.315132	-4.589635
C	2.893048	3.151843	-2.640493
H	1.831562	3.264819	-2.863259
H	3.107520	3.725378	-1.739025
H	3.471856	3.581225	-3.463354
C	5.786248	0.199693	-0.093391
H	6.775955	0.619088	-0.282345

H	5.710694	-0.023472	0.971674
H	5.706179	-0.733161	-0.646528
C	4.962411	2.522455	0.242010
H	4.970708	2.348704	1.316226
H	5.931391	2.941444	-0.038179
H	4.194960	3.261668	0.019554
C	3.348210	-2.132795	-0.893867
H	3.749060	-1.321673	-1.498848
C	2.274943	1.765948	2.264638
H	2.535206	2.320175	1.364552
C	0.783373	2.023468	2.537432
H	0.609169	3.093378	2.678281
H	0.168565	1.681138	1.705827
H	0.451146	1.509689	3.442482
C	3.131629	2.295242	3.424385
H	4.193067	2.088270	3.278135
H	3.005711	3.375518	3.526966
H	2.836510	1.837329	4.371089
C	2.063418	-2.613364	-1.588967
H	1.619294	-3.448376	-1.044037
H	1.322112	-1.815789	-1.649948
H	2.286434	-2.949769	-2.604541
C	4.395751	-3.254088	-0.873110
H	4.673957	-3.525116	-1.894097
H	5.299587	-2.953871	-0.340214
H	4.011533	-4.155349	-0.391694
N	-0.151771	0.695016	-0.451147
N	-0.116460	1.020995	-2.210652

TS_2

110

C	-3.727349	2.677105	1.166187
C	-4.455610	1.987636	0.004301
C	-2.196425	1.140521	0.097155
C	-2.214743	2.421009	0.973350
H	-4.052439	2.220927	2.104063
H	-3.957217	3.742107	1.227121
N	-3.511636	0.877507	-0.298787
C	-5.834590	1.473070	0.417790
H	-5.776276	0.834011	1.296231
H	-6.481452	2.319607	0.655906
H	-6.304041	0.905850	-0.387589
C	-1.556644	2.193507	2.343874
H	-1.731724	3.055902	2.994448
H	-1.960900	1.305884	2.832398
H	-0.480663	2.061968	2.243386
C	-1.509401	3.607178	0.295474
H	-0.443622	3.401958	0.201818
H	-1.896812	3.801245	-0.704033
H	-1.633079	4.515258	0.893099
C	-4.638873	2.956954	-1.181015
H	-5.225465	2.503240	-1.976939
H	-5.170018	3.851467	-0.847528
H	-3.681536	3.267231	-1.596003
C	-3.929487	-0.410879	-0.739226
C	-4.127363	-1.436352	0.216115
C	-4.100655	-0.678598	-2.112325
C	-4.513387	-2.698382	-0.227622
C	-4.507404	-1.954315	-2.504461
C	-4.711096	-2.959915	-1.575469
H	-4.659941	-3.493711	0.492440
H	-4.645046	-2.168652	-3.557040
H	-5.015489	-3.947815	-1.899173
C	-3.895080	-1.224828	1.704668
H	-3.720006	-0.162453	1.863508
C	-3.807004	0.355089	-3.186409
H	-3.456662	1.253134	-2.683855
C	-2.637713	-1.967660	2.183044
H	-1.760756	-1.639504	1.628470
H	-2.457309	-1.771564	3.242585
H	-2.746489	-3.047235	2.053737
C	-5.109835	-1.633323	2.551569
H	-6.025701	-1.151221	2.206243
H	-5.274590	-2.712457	2.521833
H	-4.949856	-1.355613	3.596067
C	-2.680038	-0.097466	-4.128007
H	-2.460433	0.687816	-4.855189
H	-1.765978	-0.307871	-3.575306
H	-2.953781	-0.998080	-4.682283
C	-5.064741	0.702394	-3.996987
H	-5.391766	-0.151045	-4.595620
H	-5.896250	0.984645	-3.349893
H	-4.865727	1.531275	-4.680460

B	0.656259	0.310299	0.250312
B	-0.934805	0.489015	-0.127744
C	1.902750	0.546143	-0.549562
C	1.894827	1.133883	-1.965374
C	3.383184	1.454060	-2.221638
C	4.207033	0.561450	-1.286488
H	3.571201	2.500727	-1.973790
H	3.660441	1.312577	-3.266569
N	3.188222	0.245130	-0.229574
C	3.549088	-0.400883	1.001026
C	3.895103	0.393407	2.113264
C	3.487958	-1.806552	1.101573
C	4.262278	-0.248414	3.294611
C	3.865986	-2.394806	2.308183
C	4.264643	-1.629914	3.391433
H	4.528622	0.344190	4.160360
H	3.824475	-3.471947	2.408063
H	4.549803	-2.109406	4.319757
C	1.357768	0.085923	-2.959403
H	1.478249	0.446792	-3.984197
H	1.872634	-0.870668	-2.872091
H	0.299215	-0.086736	-2.781276
C	1.055340	2.409757	-2.094781
H	1.374434	3.160931	-1.370601
H	1.178260	2.828300	-3.098149
H	-0.000224	2.203925	-1.926911
C	4.717982	-0.707690	-1.982948
H	5.428262	-0.429168	-2.763053
H	5.236044	-1.350490	-1.272771
H	3.916895	-1.278704	-2.445775
C	5.421989	1.301555	-0.722490
H	5.936789	0.703405	0.030332
H	6.125179	1.500488	-1.532990
H	5.143754	2.255615	-0.281429
C	2.947598	-2.706735	-0.001248
H	2.723989	-2.085047	-0.864471
C	3.798781	1.913214	2.111898
H	3.558164	2.233838	1.099980
C	2.648964	2.394318	3.012445
H	2.810343	2.100650	4.051838
H	2.575606	3.483916	2.977529
H	1.695415	1.982211	2.689747
C	5.113718	2.586652	2.533559
H	5.338616	2.390136	3.583709
H	5.959941	2.236062	1.942801
H	5.037987	3.669848	2.413359
C	1.627068	-3.374810	0.419357
H	1.772053	-4.028917	1.281426
H	0.871184	-2.637739	0.677705
H	1.237681	-3.981008	-0.401423
C	3.956796	-3.783758	-0.429032
H	4.128226	-4.503168	0.374270
H	3.573807	-4.337731	-1.289069
H	4.922160	-3.358581	-0.702289
N	0.716832	-0.643577	2.636462
N	0.774469	-0.249677	1.587962
N	-1.288058	-1.298085	-1.067279
N	-1.117778	-2.322371	-1.426603

Int$_2$

110

C	-3.469629	2.925337	0.926192
C	-4.306598	2.185816	-0.122216
C	-2.138658	1.129484	-0.003786
C	-2.004107	2.463915	0.745531
H	-3.815219	2.639130	1.921675
H	-3.573324	4.008074	0.845579
N	-3.452837	0.978808	-0.356116
C	-5.697656	1.818710	0.394046
H	-5.649083	1.284364	1.339711
H	-6.272153	2.732914	0.553230
H	-6.237615	1.200430	-0.324593
C	-1.373166	2.272952	2.133779
H	-1.484778	3.191269	2.717164
H	-1.856138	1.460292	2.678368
H	-0.314632	2.050510	2.061132
C	-1.157684	3.477213	-0.040854
H	-0.138004	3.107269	-0.138799
H	-1.548443	3.653113	-1.043055
H	-1.131554	4.433592	0.488365
C	-4.474423	3.034189	-1.393716
H	-5.076922	2.519382	-2.138157
H	-4.985786	3.965165	-1.139846

H	-3.514376	3.288455	-1.839595
C	-3.980857	-0.273237	-0.810151
C	-4.330578	-1.240307	0.157445
C	-4.078462	-0.557311	-2.184749
C	-4.816312	-2.469518	-0.281967
C	-4.585692	-1.798533	-2.570072
C	-4.955598	-2.747270	-1.632567
H	-5.076746	-3.228258	0.444820
H	-4.667024	-2.034865	-3.623328
H	-5.333946	-3.710279	-1.953115
C	-4.148157	-1.021208	1.653463
H	-3.878603	0.021863	1.810649
C	-3.583263	0.390615	-3.265005
H	-3.193676	1.278263	-2.773074
C	-2.993258	-1.872093	2.207652
H	-2.055060	-1.631002	1.710733
H	-2.867314	-1.685802	3.277042
H	-3.191257	-2.937562	2.072222
C	-5.437832	-1.296092	2.442216
H	-6.286145	-0.740854	2.039422
H	-5.699042	-2.355886	2.422277
H	-5.306888	-1.011814	3.488677
C	-2.420075	-0.221477	-4.059911
H	-2.028023	0.507330	-4.773602
H	-1.609117	-0.518853	-3.400715
H	-2.737554	-1.102725	-4.621532
C	-4.707673	0.812507	-4.223086
H	-5.042403	-0.033891	-4.826549
H	-5.577742	1.197636	-3.689900
H	-4.355908	1.588343	-4.907142
B	0.679902	0.325316	0.103164
B	-0.993829	0.201966	-0.243694
C	1.846932	0.524507	-0.808198
C	1.718304	0.791878	-2.314299
C	3.130076	1.272695	-2.707100
C	4.106921	0.694645	-1.675690
H	3.163236	2.363141	-2.657135
H	3.395426	0.984490	-3.724986
N	3.183165	0.435334	-0.523839
C	3.676434	0.017005	0.756290
C	4.001894	0.989552	1.724182
C	3.772061	-1.359717	1.053204
C	4.509046	0.561464	2.950194
C	4.280882	-1.730960	2.296923
C	4.664299	-0.785076	3.232937
H	4.762678	1.294625	3.705287
H	4.359903	-2.781488	2.544847
H	5.057051	-1.099212	4.192240
C	1.360363	-0.522298	-3.037384
H	1.349927	-0.362702	-4.119117
H	2.072707	-1.316598	-2.821104
H	0.379219	-0.875483	-2.736004
C	0.682109	1.858186	-2.681465
H	0.898961	2.802254	-2.180905
H	0.701997	2.032276	-3.761014
H	-0.322607	1.550938	-2.397376
C	4.799785	-0.586855	-2.162421
H	5.443413	-0.350969	-3.011651
H	5.426074	-1.001107	-1.373031
H	4.094807	-1.350427	-2.478147
C	5.207001	1.700064	-1.325547
H	5.836575	1.330087	-0.515384
H	5.841918	1.855051	-2.199736
H	4.794423	2.664053	-1.036763
C	3.274664	-2.456237	0.120049
H	2.952927	-1.987748	-0.805578
C	3.735318	2.476055	1.530978
H	3.376221	2.620935	0.513587
C	2.617357	2.958149	2.469619
H	2.912772	2.868295	3.517216
H	2.386282	4.007810	2.272382
H	1.708223	2.379578	2.328355
C	4.992333	3.335902	1.733198
H	5.325677	3.307917	2.772707
H	5.822279	3.002460	1.111109
H	4.779686	4.379008	1.487171
C	2.046144	-3.176772	0.700861
H	2.284737	-3.663735	1.648856
H	1.222686	-2.487377	0.870013
H	1.701966	-3.945520	0.005344
C	4.366749	-3.484220	-0.215619
H	4.645959	-4.066287	0.665010

H	4.002353	-4.185924	-0.969353
H	5.270281	-3.013128	-0.601010
N	0.963197	-0.304401	2.568296
N	0.927163	-0.032571	1.477572
N	-1.268810	-1.112651	-0.769076
N	-1.337384	-2.175865	-1.131525

Int_3

110

C	-5.069458	2.391300	-0.808285
C	-5.314845	0.906354	-0.491862
C	-2.941040	1.294598	-0.619126
C	-3.583057	2.539968	-1.214927
H	-5.255575	2.983541	0.089936
H	-5.744628	2.755214	-1.583323
N	-3.917833	0.440488	-0.217082
C	-6.229401	0.717583	0.720048
H	-6.284777	-0.332247	1.012231
H	-5.890342	1.298397	1.574778
H	-7.237276	1.050835	0.466608
C	-2.998994	3.836536	-0.631715
H	-1.954956	3.953984	-0.915307
H	-3.568318	4.694670	-1.000333
H	-3.057174	3.830593	0.458823
C	-3.390308	2.544067	-2.744445
H	-3.902342	3.404726	-3.183444
H	-2.329003	2.607042	-2.983908
H	-3.789220	1.639910	-3.205369
C	-5.942771	0.162322	-1.679551
H	-6.045454	-0.899732	-1.460961
H	-6.940025	0.563385	-1.870474
H	-5.356275	0.274736	-2.588951
C	-3.596934	-0.810068	0.407243
C	-3.415744	-0.842051	1.806319
C	-3.356165	-1.953658	-0.381862
C	-3.074235	-2.055446	2.400768
C	-3.018341	-3.141731	0.266039
C	-2.888230	-3.200369	1.643329
H	-2.929410	-2.099653	3.472606
H	-2.829093	-4.030498	-0.322227
H	-2.617906	-4.132379	2.124326
C	-3.496152	0.399470	2.683565
H	-3.815700	1.231979	2.059386
C	-3.360865	-1.937052	-1.903541
H	-3.655573	-0.943031	-2.227512
C	-2.114483	0.773611	3.246238
H	-2.189766	1.676063	3.857602
H	-1.713328	-0.024503	3.874900
H	-1.400743	0.967923	2.447924
C	-4.510823	0.238477	3.825602
H	-4.625353	1.181794	4.364775
H	-5.490882	-0.065832	3.457044
H	-4.180215	-0.512454	4.546105
C	-1.954093	-2.188010	-2.471923
H	-1.247117	-1.433247	-2.134487
H	-1.572876	-3.167081	-2.174412
H	-1.981411	-2.157534	-3.563708
C	-4.353380	-2.951490	-2.491773
H	-5.355076	-2.831920	-2.077667
H	-4.416883	-2.833704	-3.575810
H	-4.035022	-3.976554	-2.290214
B	2.008759	1.470816	-0.786353
B	-1.471175	1.082202	-0.536288
C	3.438584	1.285834	-0.662482
C	4.487142	2.234354	-1.228620
C	5.772162	1.777321	-0.499292
C	5.555629	0.316533	-0.054507
H	5.920197	2.399023	0.386039
H	6.660802	1.881183	-1.123185
N	4.066672	0.233788	-0.049763
C	3.337840	-0.856123	0.527534
C	2.941930	-0.782969	1.877735
C	2.989856	-1.965859	-0.267124
C	2.315265	-1.891996	2.444694
C	2.363797	-3.049493	0.348617
C	2.051923	-3.027771	1.697388
H	2.005245	-1.855287	3.481241
H	2.092239	-3.913074	-0.245171
H	1.562781	-3.878689	2.155804
C	4.594401	2.053001	-2.755967
H	5.398015	2.677270	-3.157371
H	4.799790	1.018016	-3.027623
H	3.658152	2.341277	-3.235674

C	4.159181	3.700898	-0.924212
H	3.223835	3.995735	-1.403741
H	4.049009	3.859803	0.149620
H	4.953883	4.355205	-1.293299
C	6.181943	-0.693538	-1.028800
H	7.268977	-0.592119	-1.014789
H	5.934612	-1.712216	-0.730867
H	5.843258	-0.543337	-2.051539
C	6.147134	0.058025	1.334196
H	5.893996	-0.941994	1.689015
H	7.235002	0.135007	1.285462
H	5.790594	0.784130	2.061590
C	3.166716	-1.978781	-1.777470
H	3.748387	-1.103103	-2.053893
C	3.079341	0.487962	2.702420
H	3.674088	1.197124	2.129091
C	1.699087	1.133774	2.913198
H	1.036323	0.470397	3.472589
H	1.797846	2.066218	3.474836
H	1.227558	1.359126	1.957271
C	3.777361	0.255474	4.049482
H	4.741957	-0.238583	3.925805
H	3.943636	1.208261	4.557910
H	3.169676	-0.364912	4.711512
C	1.799602	-1.839259	-2.468561
H	1.150975	-2.684786	-2.231027
H	1.297756	-0.925665	-2.151443
H	1.924953	-1.802382	-3.553625
C	3.904382	-3.222598	-2.289600
H	3.318936	-4.129928	-2.126442
H	4.083872	-3.138869	-3.363955
H	4.867043	-3.356133	-1.794450
N	-0.445369	1.986861	-1.042481
N	0.771830	1.757183	-0.932086
N	-0.895725	-0.101300	0.076273
N	-0.132785	-0.830369	0.460376

TS$_3$

110

C	-4.839915	2.738843	-1.329536
C	-5.080652	1.328486	-0.762891
C	-2.708420	1.704129	-0.960708
C	-3.359210	2.816188	-1.772046
H	-5.015567	3.474182	-0.542001
H	-5.525323	2.967889	-2.146275
N	-3.684165	0.912838	-0.424752
C	-5.985730	1.357828	0.471252
H	-6.049005	0.372651	0.935625
H	-5.631013	2.069430	1.213260
H	-6.992677	1.656522	0.174061
C	-2.779264	4.202317	-1.453783
H	-1.739344	4.276189	-1.768303
H	-3.354956	4.973769	-1.973161
H	-2.823834	4.403577	-0.381784
C	-3.185387	2.534356	-3.278208
H	-3.711242	3.290936	-3.867477
H	-2.128447	2.559195	-3.543743
H	-3.576892	1.555190	-3.555490
C	-5.727422	0.393892	-1.796285
H	-5.818265	-0.615515	-1.397524
H	-6.730872	0.753194	-2.032613
H	-5.159977	0.351438	-2.723110
C	-3.361356	-0.237552	0.368245
C	-3.191816	-0.083628	1.759654
C	-3.118033	-1.478638	-0.255094
C	-2.864241	-1.207338	2.516820
C	-2.794531	-2.570859	0.549586
C	-2.679031	-2.444934	1.923640
H	-2.731058	-1.106431	3.586474
H	-2.607742	-3.532187	0.087870
H	-2.420868	-3.305270	2.528989
C	-3.272879	1.265685	2.459208
H	-3.576281	2.007316	1.722536
C	-3.111121	-1.665283	-1.765399
H	-3.409714	-0.724876	-2.220060
C	-1.893827	1.698369	2.981782
H	-1.958551	2.681730	3.453918
H	-1.513114	0.994166	3.724952
H	-1.168930	1.758230	2.172579
C	-4.300638	1.271714	3.600784
H	-4.406707	2.279681	4.008907
H	-5.281675	0.933888	3.265921
H	-3.986864	0.619037	4.418109

C	-1.696324	-1.976243	-2.280393
H	-0.999342	-1.182439	-2.020778
H	-1.316460	-2.909632	-1.860460
H	-1.705872	-2.076722	-3.368455
C	-4.089652	-2.756143	-2.225842
H	-5.097492	-2.589415	-1.844396
H	-4.139329	-2.783650	-3.316724
H	-3.768530	-3.743254	-1.886608
B	1.967886	1.340712	-0.678753
B	-1.253796	1.518364	-0.825760
C	3.399058	1.178002	-0.501104
C	4.424017	2.050497	-1.225345
C	5.718717	1.752773	-0.431959
C	5.549440	0.372982	0.231164
H	5.838457	2.508870	0.346479
H	6.606569	1.792211	-1.064507
N	4.062991	0.251342	0.263992
C	3.353811	-0.732739	1.026828
C	2.930175	-0.414938	2.332458
C	2.998410	-1.958274	0.431785
C	2.222186	-1.375388	3.053080
C	2.290638	-2.885877	1.195519
C	1.914700	-2.607030	2.498733
H	1.885595	-1.145913	4.056258
H	2.006256	-3.831941	0.752419
H	1.358974	-3.338428	3.072923
C	4.539776	1.620385	-2.701541
H	5.346215	2.169206	-3.196469
H	4.743131	0.554331	-2.799121
H	3.606340	1.827266	-3.226113
C	4.081586	3.544179	-1.170816
H	3.155485	3.752401	-1.707452
H	3.954625	3.876891	-0.139227
H	4.882232	4.131742	-1.628728
C	6.207886	-0.750957	-0.585854
H	7.290030	-0.606703	-0.599362
H	6.005213	-1.721502	-0.133718
H	5.860166	-0.770906	-1.616000
C	6.161790	0.340699	1.635375
H	5.923175	-0.593547	2.146413
H	7.247899	0.414923	1.558500
H	5.811744	1.168724	2.247205
C	3.266076	-2.271320	-1.032404
H	3.866085	-1.463700	-1.441553
C	3.134566	0.959939	2.951372
H	3.751978	1.543385	2.271525
C	1.797789	1.706109	3.083449
H	1.105943	1.167999	3.734238
H	1.958587	2.699640	3.509727
H	1.322697	1.825544	2.111589
C	3.847845	0.891561	4.309700
H	4.785052	0.337501	4.246885
H	4.068754	1.898436	4.672062
H	3.224072	0.403020	5.061265
C	1.955249	-2.296478	-1.834494
H	1.278645	-3.070356	-1.466256
H	1.440584	-1.340097	-1.765996
H	2.160112	-2.500897	-2.888476
C	4.037702	-3.584449	-1.223748
H	3.437441	-4.445834	-0.922689
H	4.299973	-3.719574	-2.275787
H	4.957985	-3.602707	-0.639047
N	-0.153760	2.305120	-1.442799
N	1.024484	2.106336	-1.276114
N	-0.539214	0.496678	-0.067999
N	0.550281	0.188172	0.162572

Pdt

110

C	-5.086631	-1.149605	-2.370232
C	-5.151980	-0.720903	-0.895927
C	-2.869536	-0.632829	-1.624883
C	-3.688408	-0.759035	-2.899836
H	-5.889042	-0.699095	-2.955298
H	-5.205441	-2.233136	-2.433709
N	-3.686063	-0.629395	-0.556701
C	-5.851488	0.630908	-0.702690
H	-6.906840	0.527422	-0.959243
H	-5.786792	0.951389	0.336866
H	-5.427457	1.409652	-1.331996
C	-3.700497	0.593176	-3.643485
H	-2.686056	0.872563	-3.926268
H	-4.307752	0.509950	-4.548751

H	-4.118732	1.391259	-3.029854
C	-3.141227	-1.840419	-3.843194
H	-3.830115	-1.984271	-4.680485
H	-2.166509	-1.552665	-4.233377
H	-3.031750	-2.793802	-3.321772
C	-5.879294	-1.754712	-0.037396
H	-5.852398	-1.480197	1.017320
H	-6.924692	-1.800092	-0.347803
H	-5.450499	-2.747692	-0.152853
C	-3.242754	-0.317911	0.781867
C	-3.012982	1.028532	1.136349
C	-3.033430	-1.360730	1.702862
C	-2.676055	1.307527	2.459464
C	-2.698381	-1.021762	3.013497
C	-2.542198	0.298699	3.398396
H	-2.492226	2.332574	2.753529
H	-2.531064	-1.808999	3.737148
H	-2.280704	0.540203	4.421361
C	-3.022660	2.176746	0.134512
H	-3.376495	1.795512	-0.819119
C	-3.043774	-2.831826	1.312553
H	-3.367011	-2.899704	0.274480
C	-1.598063	2.705813	-0.106389
H	-1.611255	3.473248	-0.884162
H	-1.186634	3.150677	0.801236
H	-0.921819	1.914934	-0.425913
C	-3.947070	3.328932	0.553369
H	-3.990712	4.081626	-0.237608
H	-4.962915	2.987119	0.751184
H	-3.581974	3.824633	1.455041
C	-1.619342	-3.411014	1.375495
H	-0.937135	-2.822542	0.762615
H	-1.243529	-3.408588	2.401867
H	-1.621691	-4.445747	1.022987
C	-3.995767	-3.675212	2.173742
H	-5.010460	-3.277893	2.182156
H	-4.033377	-4.699769	1.796298
H	-3.650618	-3.723533	3.208603
B	1.326599	0.347944	-1.472200
B	-1.371862	-0.493877	-1.588820
C	2.683692	0.993545	-1.317448
C	3.247274	2.024688	-2.284156
C	4.732091	2.121582	-1.871022
C	4.847638	1.562887	-0.444673
H	5.110251	3.142197	-1.934591
H	5.332703	1.510867	-2.546962
N	3.568105	0.773169	-0.329022
C	3.393611	-0.214279	0.709974
C	2.890760	0.160003	1.968229
C	3.727647	-1.554496	0.426871
C	2.825347	-0.813125	2.965884
C	3.626339	-2.487991	1.455897
C	3.200646	-2.122387	2.722531
H	2.441790	-0.544269	3.941803
H	3.872175	-3.523415	1.257890
H	3.135250	-2.863437	3.509772
C	3.137620	1.596438	-3.754745
H	3.609728	0.624621	-3.912365
H	2.101522	1.515300	-4.075109
H	3.650421	2.331728	-4.380850
C	2.496855	3.357849	-2.087177
H	1.445341	3.241255	-2.350001
H	2.548224	3.703056	-1.053513
H	2.932405	4.129427	-2.727912
C	6.076869	0.667384	-0.282649
H	6.975670	1.277072	-0.390127
H	6.101021	0.194332	0.699275
H	6.106404	-0.109269	-1.043459
C	4.922740	2.680940	0.600571
H	4.972162	2.270024	1.605854
H	5.829345	3.264742	0.431790
H	4.070560	3.355257	0.540064
C	4.108973	-2.047375	-0.962233
H	4.230521	-1.182142	-1.612638
C	2.301267	1.530711	2.264792
H	2.455692	2.161424	1.391882
C	0.783392	1.421528	2.479027
H	0.349843	2.415397	2.611712
H	0.296992	0.939249	1.636269
H	0.553354	0.837151	3.371976
C	2.944784	2.214270	3.480935
H	4.030823	2.264272	3.406798

H	2.564328	3.233285	3.585119
H	2.703734	1.680407	4.402489
C	2.979904	-2.904699	-1.560777
H	2.829562	-3.812170	-0.971319
H	2.038660	-2.358836	-1.586656
H	3.233702	-3.203315	-2.580754
C	5.429908	-2.832535	-0.970385
H	5.720216	-3.067864	-1.997030
H	6.240930	-2.272025	-0.504141
H	5.333203	-3.778824	-0.434501
N	-0.636150	0.013088	-2.717007
N	0.586003	0.258055	-2.752242
N	0.645956	-0.344217	-0.417335
N	-0.478258	-0.876195	-0.467633

LiCl

Li	-1.607037	0.000930	-1.815455
Cl	-1.243769	1.743825	-0.865686

RC_{LiCl}

110

C	4.008453	-4.675557	1.883389
C	4.298357	-4.193131	0.450399
C	2.030447	-3.666514	0.948418
C	2.470198	-4.619779	2.068134
H	4.487441	-3.998767	2.593619
H	4.407323	-5.674212	2.063100
N	3.111573	-3.305085	0.222374
C	5.628902	-3.457572	0.308432
H	5.757589	-2.677523	1.054337
H	6.444391	-4.172490	0.427373
H	5.718432	-3.005428	-0.680511
C	2.099315	-4.025242	3.437357
H	2.487046	-4.660083	4.238622
H	2.518491	-3.025634	3.559753
H	1.016049	-3.954564	3.546150
C	1.803224	-5.997982	1.933256
H	2.017328	-6.465417	0.972503
H	2.157565	-6.664206	2.723859
H	0.721840	-5.895362	2.018598
C	4.293562	-5.371035	-0.540556
H	4.517077	-5.035770	-1.551326
H	5.058948	-6.093317	-0.250213
H	3.331627	-5.880153	-0.554277
C	3.073561	-2.134654	-0.617114
C	3.398094	-0.898073	-0.016574
C	2.634662	-2.206251	-1.949184
C	3.241875	0.260733	-0.770908
C	2.524253	-1.014469	-2.668135
C	2.808224	0.207230	-2.085548
H	3.418144	1.223639	-0.313546
H	2.181648	-1.045617	-3.694252
H	2.668842	1.123476	-2.644248
C	3.861836	-0.779611	1.430381
H	4.117998	-1.774764	1.785062
C	2.285568	-3.513868	-2.638735
H	2.298426	-4.301673	-1.887315
C	2.743431	-0.269201	2.345786
H	1.923615	-0.989101	2.362407
H	3.106328	-0.161644	3.371462
H	2.349959	0.689809	2.007442
C	5.118686	0.091542	1.566315
H	5.496738	0.047098	2.590509
H	5.910640	-0.240928	0.892545
H	4.907319	1.138123	1.341569
C	0.874920	-3.499007	-3.241691
H	0.655265	-4.467865	-3.696293
H	0.131425	-3.302128	-2.471169
H	0.775720	-2.739875	-4.019962
C	3.323909	-3.851312	-3.721193
H	3.274819	-3.129929	-4.540356
H	4.341983	-3.827200	-3.329056
H	3.139000	-4.844409	-4.137987
B	-0.766796	-3.536272	0.314569
B	0.636495	-3.278003	0.721975
C	-2.087989	-4.005342	-0.082753
C	-2.352538	-5.425190	-0.592507
C	-3.763426	-5.292934	-1.210392
C	-4.454006	-4.100711	-0.522274
H	-4.344981	-6.209668	-1.109681
H	-3.666548	-5.081372	-2.276995
N	-3.264739	-3.337676	-0.024137
C	-3.318060	-2.000054	0.494280
C	-3.501374	-1.799596	1.877407

C	-3.109563	-0.912730	-0.382637
C	-3.570474	-0.487638	2.346646
C	-3.160386	0.376460	0.147646
C	-3.409801	0.591419	1.493380
H	-3.715853	-0.310609	3.404914
H	-2.957578	1.224053	-0.491081
H	-3.429187	1.601640	1.881072
C	-1.327261	-5.887963	-1.630890
H	-0.321414	-5.886814	-1.208189
H	-1.562767	-6.901844	-1.965535
H	-1.326526	-5.230817	-2.499283
C	-2.331255	-6.405809	0.597635
H	-2.642400	-7.400881	0.268330
H	-1.324215	-6.473101	1.007713
H	-2.994403	-6.089911	1.401845
C	-5.287131	-3.277057	-1.507261
H	-4.714499	-3.002288	-2.389740
H	-6.146341	-3.866101	-1.832877
H	-5.661473	-2.366095	-1.038264
C	-5.382842	-4.539612	0.619335
H	-6.220444	-5.103576	0.205057
H	-4.880353	-5.173878	1.345770
H	-5.787260	-3.672066	1.138742
C	-2.777458	-1.083625	-1.859878
H	-2.838490	-2.144342	-2.097019
C	-3.537005	-2.943673	2.880321
H	-3.533564	-3.875624	2.322974
C	-2.270121	-2.952118	3.751413
H	-2.265471	-3.832381	4.399221
H	-1.374499	-2.982420	3.129855
H	-2.224187	-2.067839	4.389982
C	-4.791154	-2.919373	3.764535
H	-4.824191	-3.807916	4.399452
H	-4.796340	-2.047167	4.421568
H	-5.706683	-2.890805	3.173069
C	-1.340132	-0.638526	-2.174211
H	-1.134942	-0.770691	-3.239292
H	-1.166933	0.405922	-1.914305
H	-0.616012	-1.242283	-1.626887
C	-3.771037	-0.330793	-2.758573
H	-3.576525	-0.560289	-3.808970
H	-4.805465	-0.595688	-2.536603
H	-3.669961	0.749026	-2.635374
N	-0.473520	0.266579	4.087723
N	-0.432609	-0.178078	3.093507
Li	-0.092680	-0.561554	0.877334
Cl	0.167099	1.512894	0.389098

TS_{1LiCl}

110

Li	0.393210	-1.851943	-0.141751
Cl	-0.436335	-3.792664	-0.368097
C	-3.060712	-0.759799	2.783420
C	-3.818065	-1.163645	1.514472
C	-1.773429	-0.104312	0.849428
C	-1.617317	-0.406362	2.357540
H	-3.080294	-1.548979	3.534868
H	-3.535759	0.120761	3.220444
N	-3.029862	-0.403571	0.470669
C	-3.746151	-2.679268	1.279828
H	-4.351996	-2.977056	0.427986
H	-2.729200	-3.027897	1.104863
H	-4.143192	-3.190389	2.159370
C	-0.637898	-1.560688	2.627605
H	-0.916601	-2.476283	2.107713
H	0.370444	-1.281967	2.318414
H	-0.601717	-1.770791	3.699119
C	-1.145279	0.846212	3.117627
H	-1.162384	0.646996	4.192655
H	-0.136289	1.126444	2.825079
H	-1.799043	1.696730	2.916778
C	-5.280584	-0.729793	1.545730
H	-5.778810	-0.941866	0.599086
H	-5.794871	-1.287485	2.329764
H	-5.386707	0.331422	1.761344
C	-3.641971	0.111132	-0.727589
C	-3.764679	-0.683515	-1.881888
C	-4.097786	1.449055	-0.706525
C	-4.379863	-0.121481	-3.001871
C	-4.684683	1.962312	-1.860100
C	-4.834143	1.185448	-2.998756
H	-4.481180	-0.717387	-3.899458
H	-5.028161	2.988534	-1.870081

H	-5.297080	1.603287	-3.884362
C	-3.206570	-2.089927	-1.992387
H	-2.699144	-2.340854	-1.065380
C	-3.941947	2.368212	0.498550
H	-3.616515	1.766661	1.345060
C	-2.148558	-2.198969	-3.100645
H	-2.587118	-2.062918	-4.092395
H	-1.364300	-1.454840	-2.976805
H	-1.676998	-3.180479	-3.053525
C	-4.315086	-3.126099	-2.232827
H	-5.125525	-3.036548	-1.507324
H	-4.754274	-3.009068	-3.226673
H	-3.901257	-4.133607	-2.162098
C	-2.858046	3.432070	0.255815
H	-2.757896	4.077036	1.132302
H	-1.889523	2.971026	0.066286
H	-3.116158	4.062192	-0.598165
C	-5.266053	3.039155	0.896100
H	-6.068768	2.310692	1.018358
H	-5.144804	3.576484	1.839492
H	-5.589065	3.764298	0.146921
B	0.803869	1.025866	0.206124
B	-0.611581	0.453832	0.114811
C	2.081519	1.665207	0.108490
C	2.299144	3.175895	0.267841
C	3.845873	3.295124	0.261008
C	4.399615	2.022162	-0.411529
H	4.208086	3.335093	1.290579
H	4.185126	4.202921	-0.239792
N	3.309957	1.060356	-0.100938
C	3.400428	-0.358402	0.042705
C	3.617597	-0.893220	1.332437
C	3.130798	-1.212613	-1.049611
C	3.558257	-2.276720	1.502103
C	3.078086	-2.591485	-0.824946
C	3.280892	-3.122449	0.440288
H	3.708758	-2.698346	2.488079
H	2.851432	-3.260159	-1.644674
H	3.198041	-4.189834	0.595978
C	1.642146	3.956563	-0.883493
H	1.847245	5.026531	-0.786290

H	2.001766	3.628198	-1.857534
H	0.560153	3.812813	-0.866766
C	1.719384	3.681052	1.598537
H	2.125113	3.115464	2.439155
H	1.958146	4.738711	1.744790
H	0.633441	3.570150	1.613570
C	4.580291	2.230656	-1.928037
H	3.636842	2.467748	-2.416595
H	5.271588	3.057046	-2.107648
H	4.996204	1.341268	-2.399120
C	5.746450	1.585456	0.165402
H	6.026811	0.598016	-0.206738
H	6.520531	2.290506	-0.141619
H	5.735418	1.552547	1.252057
C	2.894558	-0.689693	-2.456932
H	2.862285	0.394618	-2.400204
C	3.883011	-0.019335	2.548475
H	3.946057	1.010897	2.205658
C	2.727053	-0.074943	3.555280
H	2.937628	0.571074	4.411146
H	1.804395	0.265672	3.090448
H	2.569950	-1.088672	3.930662
C	5.212782	-0.380496	3.227768
H	5.435973	0.332572	4.024868
H	5.172124	-1.374655	3.677731
H	6.042447	-0.372532	2.519293
C	1.547873	-1.145581	-3.031197
H	1.479608	-2.230224	-3.125501
H	0.728923	-0.808924	-2.395402
H	1.387957	-0.712315	-4.021069
C	4.043557	-1.097982	-3.391444
H	4.061133	-2.180627	-3.535187
H	3.925158	-0.630963	-4.371940
H	5.013778	-0.804364	-2.987749
N	-1.163517	0.931590	-1.700497
N	-1.095138	1.451095	-2.666862

Int$_{1LiCl}$

110

Li	0.454396	-1.908613	-0.238543
Cl	-0.441424	-3.828759	-0.381876
C	-2.767392	-0.761161	2.893764

C	-3.665039	-1.163518	1.718077
C	-1.720277	-0.083246	0.827636
C	-1.397561	-0.353835	2.302390
H	-2.672485	-1.567336	3.620351
H	-3.208693	0.093874	3.409546
N	-2.990579	-0.414525	0.586536
C	-3.625792	-2.678108	1.479110
H	-4.299188	-2.970205	0.677888
H	-2.627565	-3.036328	1.229038
H	-3.958380	-3.182323	2.388822
C	-0.341094	-1.458204	2.467559
H	-0.645069	-2.397930	2.006485
H	0.609780	-1.139072	2.034930
H	-0.159380	-1.641837	3.528622
C	-0.901612	0.931224	2.990696
H	-0.809032	0.751102	4.065141
H	0.064583	1.238975	2.598687
H	-1.603237	1.753943	2.841922
C	-5.112315	-0.718647	1.905320
H	-5.710322	-0.926026	1.017410
H	-5.543889	-1.273296	2.739783
H	-5.185889	0.342560	2.133182
C	-3.711723	0.042373	-0.578555
C	-3.886940	-0.787055	-1.699988
C	-4.197611	1.368800	-0.558898
C	-4.599614	-0.273520	-2.785148
C	-4.882904	1.832034	-1.679364
C	-5.094857	1.018104	-2.780941
H	-4.744189	-0.895163	-3.658886
H	-5.252323	2.848952	-1.692689
H	-5.633721	1.397291	-3.640403
C	-3.285633	-2.174600	-1.823378
H	-2.712558	-2.393069	-0.927268
C	-3.976130	2.330980	0.602165
H	-3.563113	1.769812	1.438582
C	-2.295598	-2.263864	-2.995147
H	-2.800362	-2.151307	-3.957671
H	-1.527445	-1.495201	-2.931610
H	-1.794780	-3.231352	-2.968253
C	-4.365579	-3.256171	-1.978269
H	-5.134720	-3.183169	-1.207367

H	-4.866538	-3.176451	-2.946025
H	-3.908396	-4.245071	-1.913949
C	-2.953696	3.421167	0.238980
H	-2.803619	4.096027	1.085127
H	-1.988632	2.988447	-0.020967
H	-3.303079	4.016032	-0.607775
C	-5.287079	2.972590	1.083828
H	-6.052137	2.223723	1.291818
H	-5.109983	3.545728	1.996682
H	-5.692132	3.661287	0.340015
B	0.773645	1.079299	0.097573
B	-0.693239	0.508459	-0.125966
C	2.044456	1.708233	0.089032
C	2.291549	3.202860	0.365398
C	3.838950	3.312528	0.273715
C	4.342751	2.066028	-0.485147
H	4.263577	3.306000	1.279919
H	4.152754	4.240987	-0.205980
N	3.292231	1.093291	-0.113253
C	3.385157	-0.320266	0.025011
C	3.649784	-0.859910	1.304820
C	3.080934	-1.180952	-1.056579
C	3.587589	-2.242957	1.480977
C	3.032235	-2.560536	-0.829008
C	3.269881	-3.090256	0.432073
H	3.773417	-2.663137	2.461717
H	2.790119	-3.231980	-1.642567
H	3.189253	-4.157225	0.591860
C	1.581606	4.096731	-0.664508
H	1.794608	5.152614	-0.471587
H	1.892503	3.866673	-1.682690
H	0.500358	3.952395	-0.610003
C	1.812299	3.597505	1.772127
H	2.245641	2.940224	2.527792
H	2.099224	4.627743	2.004620
H	0.725539	3.523553	1.846754
C	4.394293	2.342408	-2.002445
H	3.403867	2.560778	-2.399349
H	5.037623	3.202021	-2.203941
H	4.800587	1.489971	-2.544808
C	5.732463	1.618625	-0.034921

H	5.989741	0.651383	-0.472273
H	6.477761	2.343592	-0.366126
H	5.802925	1.534492	1.047144
C	2.828499	-0.657453	-2.461209
H	2.757117	0.424280	-2.392676
C	3.987120	0.014834	2.501667
H	4.031832	1.042292	2.147808
C	2.900210	-0.034847	3.583225
H	3.174206	0.607226	4.424054
H	1.948235	0.312611	3.188951
H	2.762376	-1.048243	3.967648
C	5.356364	-0.349388	3.096352
H	5.634393	0.366371	3.873695
H	5.340484	-1.341658	3.552425
H	6.138328	-0.348712	2.335465
C	1.506141	-1.156457	-3.056649
H	1.479175	-2.241343	-3.168976
H	0.662718	-0.859194	-2.431917
H	1.342239	-0.716169	-4.042350
C	4.000367	-1.018309	-3.387166
H	4.057461	-2.099198	-3.535515
H	3.875985	-0.549794	-4.366180
H	4.954567	-0.692544	-2.970641
N	-1.123540	0.843339	-1.502378
N	-1.322518	1.168376	-2.548949

TS_{2LiCl}

112

Li	1.933229	-1.526759	-1.754246
Cl	2.305774	-3.530460	-2.270586
C	3.900396	0.240064	-0.080420
C	3.149621	0.269086	1.257966
B	0.181440	0.114777	-1.075676
C	1.500174	0.195082	-0.502460
C	1.694171	-0.174396	0.993824
H	3.149671	1.291696	1.641819
H	3.631759	-0.357417	2.009219
N	2.776395	0.464611	-1.074972
C	4.962733	1.335337	-0.151021
H	4.544642	2.320351	0.039402
H	5.721698	1.137125	0.607519
H	5.455108	1.355373	-1.123626
C	0.736428	0.604513	1.909764
H	1.011706	0.444587	2.955937
H	0.768435	1.675635	1.708447
H	-0.290602	0.267352	1.778496
C	1.491640	-1.676029	1.270872
H	0.449496	-1.949332	1.108392
H	2.102438	-2.320243	0.637740
H	1.742207	-1.899388	2.311374
C	4.595409	-1.112985	-0.311439
H	5.181438	-1.104772	-1.228572
H	5.279669	-1.314296	0.515320
H	3.902441	-1.951394	-0.377850
C	2.931279	1.426629	-2.140528
C	2.555277	2.779753	-1.947758
C	3.458303	1.012272	-3.382516
C	2.691392	3.669475	-3.010152
C	3.588194	1.951284	-4.407223
C	3.203607	3.267630	-4.233720
H	2.395754	4.701987	-2.874910
H	3.987195	1.636118	-5.362673
H	3.303683	3.978879	-5.044302
C	1.986624	3.321918	-0.644825
H	2.017083	2.526774	0.093894
C	3.867826	-0.418592	-3.688915
H	3.686141	-1.036414	-2.812763
C	0.517921	3.737055	-0.814182
H	-0.074270	2.920843	-1.220294
H	0.082799	4.021913	0.145649
H	0.428352	4.589513	-1.490887
C	2.801188	4.501331	-0.088649
H	3.858604	4.256875	0.017412
H	2.730358	5.377353	-0.736135
H	2.418384	4.787381	0.893710
C	3.051165	-1.023544	-4.841612
H	3.267128	-2.088539	-4.925747
H	1.977864	-0.924183	-4.671935
H	3.283647	-0.537001	-5.791544
C	5.371680	-0.531792	-3.979192
H	5.637078	-0.005341	-4.898809
H	5.969263	-0.105944	-3.171171
H	5.651528	-1.580309	-4.097249

B	-1.447967	0.026617	-0.879324
C	-2.460464	-0.969651	-1.386932
C	-2.090808	-2.338736	-1.969281
C	-3.460665	-3.045018	-2.109787
C	-4.534721	-1.955336	-2.124095
H	-3.619320	-3.692342	-1.245649
H	-3.508125	-3.673280	-2.999098
N	-3.803629	-0.835799	-1.432295
C	-4.492166	0.304334	-0.893218
C	-4.909775	0.257466	0.452461
C	-4.675277	1.461044	-1.676251
C	-5.576640	1.364114	0.973616
C	-5.353401	2.537247	-1.103802
C	-5.811556	2.490410	0.201832
H	-5.899513	1.352398	2.006377
H	-5.503439	3.437697	-1.685260
H	-6.330169	3.341230	0.626144
C	-1.381079	-2.201090	-3.328126
H	-1.236190	-3.188236	-3.769871
H	-1.946619	-1.584696	-4.027612
H	-0.393676	-1.761818	-3.207331
C	-1.204389	-3.144066	-1.010657
H	-1.651222	-3.187205	-0.014913
H	-1.084314	-4.162300	-1.386138
H	-0.208816	-2.721733	-0.926990
C	-4.935941	-1.558736	-3.551225
H	-5.399387	-2.415077	-4.043757
H	-5.661512	-0.747472	-3.539370
H	-4.079353	-1.252018	-4.148029
C	-5.800056	-2.377435	-1.377067
H	-6.502025	-1.548104	-1.279143
H	-6.292787	-3.171539	-1.940008
H	-5.576606	-2.763692	-0.385833
C	-4.096540	1.633468	-3.073335
H	-3.615512	0.700815	-3.357201
C	-4.592782	-0.907875	1.381106
H	-4.169490	-1.712667	0.782965
C	-3.523415	-0.518642	2.415824
H	-3.855732	0.318903	3.032749
H	-3.316403	-1.363918	3.076038
H	-2.591219	-0.234161	1.931012
C	-5.844636	-1.448285	2.088267
H	-6.239484	-0.725506	2.805066
H	-6.640481	-1.682341	1.380913
H	-5.600447	-2.356791	2.643395
C	-3.008995	2.721270	-3.097919
H	-3.418447	3.697985	-2.833116
H	-2.204436	2.495139	-2.402139
H	-2.578752	2.797120	-4.098713
C	-5.176837	1.958531	-4.116793
H	-5.606396	2.947325	-3.942620
H	-4.744865	1.958902	-5.119729
H	-5.995598	1.238906	-4.099780
N	-1.919112	1.205955	-0.181194
N	-2.151037	2.119849	0.425861
N	0.312338	0.777066	-2.945714
N	-0.031900	1.146853	-3.920088

Int$_{2LiCl}$

112

Li	-0.169759	3.823363	-0.399396
Cl	1.674727	4.764443	-0.109901
C	-3.438712	-3.196150	0.918869
C	-4.357793	-2.173853	0.237046
C	-2.118588	-1.277691	0.322515
C	-1.984224	-2.771604	0.617965
H	-3.648330	-4.213358	0.587823
H	-3.605359	-3.162222	1.997126
N	-3.422236	-1.002798	0.106725
C	-4.862257	-2.650985	-1.131260
H	-5.443748	-1.869990	-1.619042
H	-4.053155	-2.945778	-1.794632
H	-5.513170	-3.515264	-0.990331
C	-1.425307	-3.497415	-0.623134
H	-1.992050	-3.269307	-1.525178
H	-0.391197	-3.218274	-0.804727
H	-1.462087	-4.577737	-0.462744
C	-1.078606	-3.071497	1.816490
H	-1.083395	-4.145721	2.019388
H	-0.054796	-2.758675	1.619889
H	-1.425172	-2.553473	2.711634
C	-5.580026	-1.844150	1.094590
H	-6.159800	-1.028061	0.661357

H	-6.223853	-2.723703	1.147234
H	-5.302188	-1.574704	2.110761
C	-3.869297	0.307000	-0.279371
C	-3.843914	0.681519	-1.638801
C	-4.240088	1.222555	0.725931
C	-4.278196	1.964349	-1.970589
C	-4.664509	2.491662	0.335662
C	-4.698611	2.858739	-0.999755
H	-4.268103	2.274309	-3.007451
H	-4.954521	3.210053	1.091436
H	-5.033203	3.849373	-1.282608
C	-3.290992	-0.203365	-2.748439
H	-3.022637	-1.162543	-2.313833
C	-4.113323	0.925003	2.213910
H	-3.818329	-0.116623	2.325917
C	-2.002611	0.382989	-3.351637
H	-2.186720	1.355049	-3.813644
H	-1.227214	0.506019	-2.599203
H	-1.615429	-0.286370	-4.122737
C	-4.320574	-0.451265	-3.862095
H	-4.536936	0.468032	-4.410183
H	-3.930964	-1.176520	-4.579875
H	-5.264069	-0.832305	-3.471910
C	-2.996638	1.768563	2.849102
H	-2.891401	1.519964	3.907537
H	-2.041747	1.579389	2.364359
H	-3.212655	2.836302	2.774851
C	-5.433394	1.135626	2.970321
H	-6.254658	0.576735	2.521629
H	-5.327336	0.813591	4.008604
H	-5.718048	2.189494	2.983046
B	0.742142	-0.574240	0.157820
B	-0.949380	-0.330337	0.228973
C	1.835680	-0.078607	1.056863
C	1.601638	0.508593	2.456269
C	3.021761	0.525821	3.063157
C	4.007443	0.572422	1.890939
H	3.182520	-0.396070	3.626674
H	3.166042	1.360323	3.749249
N	3.167635	-0.042128	0.807134
C	3.732183	-0.517886	-0.423160
C	4.117503	-1.871969	-0.507660
C	3.822117	0.339319	-1.537792
C	4.666121	-2.328898	-1.703788
C	4.383821	-0.171480	-2.708616
C	4.813509	-1.484870	-2.793035
H	4.964521	-3.365744	-1.790971
H	4.464869	0.470034	-3.576447
H	5.241588	-1.858429	-3.715328
C	1.017857	1.930414	2.372661
H	0.994236	2.375727	3.370687
H	1.589546	2.591242	1.724947
H	-0.003148	1.901888	2.002609
C	0.672680	-0.357945	3.313344
H	1.032333	-1.386440	3.371289
H	0.625504	0.046965	4.328079
H	-0.336258	-0.374588	2.903672
C	4.438514	2.002909	1.535641
H	3.602484	2.659550	1.306820
H	4.981741	2.431295	2.380090
H	5.109578	1.996327	0.678239
C	5.269579	-0.242749	2.177102
H	5.901017	-0.320637	1.291065
H	5.845128	0.258808	2.956893
H	5.036175	-1.245344	2.528476
C	3.271228	1.757153	-1.559261
H	2.901261	2.007814	-0.570206
C	3.882176	-2.873346	0.616072
H	3.524466	-2.325416	1.485939
C	2.778773	-3.876860	0.240688
H	2.606228	-4.573408	1.064930
H	1.841691	-3.365452	0.028398
H	3.056237	-4.458769	-0.640736
C	5.163744	-3.619845	1.015020
H	4.980773	-4.241837	1.894566
H	5.503680	-4.279175	0.213961
H	5.977215	-2.932034	1.245626
C	2.071252	1.882568	-2.514088
H	2.340563	1.609794	-3.536885
H	1.244963	1.244702	-2.203098
H	1.730820	2.919513	-2.515440
C	4.336722	2.797335	-1.936312

H	4.621917	2.708739	-2.987608
H	3.935337	3.797730	-1.770384
H	5.242587	2.687286	-1.339073
N	1.175927	-1.581579	-2.152293
N	1.071145	-1.112529	-1.134092
N	-1.160528	1.025380	-0.101184
N	-1.130087	2.138687	-0.341755

Int$_{3LiCl}$

112

C	4.143390	5.939278	-2.087945
C	4.354526	4.873145	-2.944437
C	4.434751	3.567956	-2.467655
C	4.316720	3.363842	-1.079323
C	4.017658	4.424165	-0.203376
C	3.957999	5.711289	-0.736016
N	4.459994	2.027026	-0.563913
C	3.408604	1.202076	-0.415149
C	3.863292	-0.138313	0.143576
C	5.403903	-0.043926	0.019529
C	5.768448	1.447285	-0.117538
B	2.017873	1.540223	-0.715923
N	0.794409	1.796484	-0.916565
N	-0.431567	2.084295	-1.124482
B	-1.466251	1.075066	-0.856063
N	-0.922293	-0.189528	-0.425266
N	-0.322629	-1.072051	-0.065988
C	3.398018	-0.278514	1.605525
C	3.312939	-1.311073	-0.678650
C	6.217622	2.062695	1.214809
C	6.873517	1.677614	-1.149900
C	4.525124	2.421650	-3.464607
C	5.675065	2.595561	-4.466052
C	3.643447	4.217323	1.257007
C	4.474658	5.076113	2.220011
C	-2.963954	1.139238	-0.885065
C	-3.901750	-0.056444	-0.658996
C	-5.279783	0.509794	-1.075123
C	-5.200053	2.036491	-0.972861
N	-3.715582	2.246581	-1.073647
C	-5.967699	2.725106	-2.103919
C	-5.755419	2.567811	0.356206
C	-3.577058	-1.270833	-1.544331
C	-3.896234	-0.484270	0.822687
C	-3.155713	3.553992	-1.284163
C	-2.943325	3.989969	-2.609310
C	-2.433276	5.272127	-2.804180
C	-2.126864	6.094215	-1.729893
C	-2.296957	5.631057	-0.436446
C	-2.796925	4.351634	-0.181678
C	-3.152111	3.088573	-3.820352
C	-4.001937	3.760004	-4.908658
C	-2.832016	3.859636	1.258475
C	-3.651078	4.777013	2.178824
C	-1.806005	2.622971	-4.403087
C	-1.407836	3.694315	1.815017
C	2.142247	4.485000	1.461259
C	3.184030	2.249423	-4.198523
Li	0.034842	3.780737	-2.038903
Cl	0.916769	5.134908	-3.436387
H	-5.481467	0.233465	-2.111429
H	-6.087944	0.102364	-0.467127
H	-5.800683	3.802740	-2.098189
H	-5.690301	2.336451	-3.080380
H	-7.035477	2.548570	-1.963915
H	-2.666083	-1.782800	-1.241882
H	-4.394652	-1.994335	-1.480582
H	-3.466849	-0.971205	-2.587614
H	-4.641780	-1.267040	0.985619
H	-2.925218	-0.878847	1.119381
H	-4.130458	0.348834	1.484662
H	-5.621933	3.646333	0.421769
H	-6.825230	2.357757	0.407620
H	-5.280442	2.107977	1.219467
H	-2.213151	5.615443	-3.804352
H	-2.006079	6.261875	0.393927
H	-1.700515	7.072029	-1.909103
H	-3.675777	2.193904	-3.488490
H	-3.289018	2.873217	1.268310
H	-1.976824	1.999568	-5.284240
H	-1.178887	3.467859	-4.693955
H	-1.252003	2.028191	-3.675185
H	-4.229835	3.043476	-5.700919

H	-4.943291	4.142583	-4.511442
H	-3.469218	4.594702	-5.367092
H	-0.823445	3.012617	1.200308
H	-0.884980	4.651951	1.847111
H	-1.444806	3.297936	2.832763
H	-4.655418	4.955756	1.794535
H	-3.741448	4.330443	3.171637
H	-3.167636	5.748535	2.299782
H	5.725197	-0.575927	-0.877428
H	5.909561	-0.502938	0.869387
H	4.404385	5.054441	-4.008871
H	3.723868	6.543858	-0.085140
H	4.060215	6.941954	-2.485985
H	3.769474	-1.215576	2.027755
H	3.759299	0.542565	2.225330
H	2.308202	-0.286043	1.659580
H	2.225950	-1.364514	-0.612984
H	3.585134	-1.210197	-1.730601
H	3.721286	-2.253557	-0.304708
H	7.160940	1.608403	1.523064
H	6.378121	3.134474	1.105941
H	5.489746	1.899892	2.007228
H	7.039246	2.742397	-1.317819
H	7.803382	1.242441	-0.780057
H	6.638493	1.210098	-2.103584
H	3.811714	3.172478	1.508396
H	4.703819	1.500374	-2.911466
H	2.891297	3.163080	-4.716884
H	3.256922	1.436321	-4.926106
H	2.384478	2.013747	-3.496910
H	6.629955	2.754177	-3.961895
H	5.763377	1.706711	-5.095437
H	5.501501	3.446557	-5.126541
H	1.891713	5.521313	1.224396
H	1.541490	3.837818	0.824147
H	1.858138	4.294847	2.499291
H	4.270806	6.139548	2.080485
H	4.229230	4.828182	3.255415
H	5.545756	4.924711	2.079940

TS_{3LiCl}

112

C	-4.799281	2.514178	-1.824290
C	-5.047537	1.089624	-1.301547
C	-2.682497	1.512432	-1.312182
C	-3.289648	2.633578	-2.145256
H	-5.055495	3.231943	-1.042918
H	-5.421070	2.740876	-2.690848
N	-3.661654	0.698275	-0.873214
C	-6.032383	1.064393	-0.131369
H	-6.094653	0.068252	0.308340
H	-5.754074	1.769614	0.647276
H	-7.024716	1.337233	-0.494179
C	-2.773542	4.023443	-1.743220
H	-1.718906	4.144695	-1.983134
H	-3.341521	4.790006	-2.276812
H	-2.898099	4.188957	-0.671436
C	-2.985833	2.391369	-3.637282
H	-3.477986	3.153463	-4.247107
H	-1.911827	2.444473	-3.817244
H	-3.334026	1.412942	-3.968523
C	-5.592872	0.162343	-2.397127
H	-5.690272	-0.857718	-2.029528
H	-6.584281	0.508409	-2.694113
H	-4.962341	0.155218	-3.283702
C	-3.367722	-0.436354	-0.039017
C	-3.271467	-0.244243	1.355392
C	-3.066342	-1.676253	-0.631169
C	-2.925722	-1.338019	2.145388
C	-2.730856	-2.739337	0.208717
C	-2.662128	-2.576753	1.581813
H	-2.797701	-1.213528	3.210640
H	-2.493908	-3.702830	-0.225096
H	-2.368526	-3.400479	2.219666
C	-3.456060	1.108333	2.031465
H	-3.767836	1.827871	1.276014
C	-3.003420	-1.890114	-2.136156
H	-3.324882	-0.973201	-2.621425
C	-2.137621	1.630482	2.629132
H	-2.308724	2.591146	3.121622
H	-1.726359	0.937847	3.365597
H	-1.389680	1.785531	1.851560
C	-4.542094	1.056065	3.117490

H	-4.734144	2.058384	3.507676
H	-5.480297	0.651671	2.735258
H	-4.226941	0.432048	3.955298
C	-1.561408	-2.144781	-2.598316
H	-0.914028	-1.315182	-2.320586
H	-1.156726	-3.055265	-2.152624
H	-1.523945	-2.257436	-3.684810
C	-3.923211	-3.026669	-2.605268
H	-4.948504	-2.890513	-2.258939
H	-3.936016	-3.079077	-3.696396
H	-3.577600	-3.993247	-2.233328
B	1.972826	1.340509	-0.695421
B	-1.225186	1.363520	-1.038924
C	3.428042	1.230870	-0.756454
C	4.284301	2.304503	-1.438207
C	5.708757	1.947872	-0.947114
C	5.697655	0.467986	-0.529396
H	5.948087	2.560467	-0.075221
H	6.465070	2.150625	-1.705755
N	4.241992	0.268278	-0.245479
C	3.723445	-0.810677	0.540383
C	3.560318	-0.614830	1.929528
C	3.325783	-2.006753	-0.081206
C	3.057380	-1.672024	2.688182
C	2.822331	-3.032791	0.722394
C	2.696942	-2.875140	2.092769
H	2.892576	-1.545403	3.748334
H	2.503397	-3.960044	0.263448
H	2.283929	-3.668175	2.702428
C	4.147416	2.197070	-2.968905
H	4.815873	2.910216	-3.458835
H	4.390352	1.198631	-3.330925
H	3.123605	2.420942	-3.270244
C	3.915534	3.730628	-1.005104
H	2.920886	4.004158	-1.355497
H	3.928017	3.822839	0.082168
H	4.634323	4.442327	-1.420731
C	6.193372	-0.453079	-1.656240
H	7.239830	-0.229137	-1.871689
H	6.130494	-1.498893	-1.357861
H	5.625930	-0.320950	-2.575107
C	6.568914	0.208035	0.701993
H	6.443612	-0.813078	1.065670
H	7.617925	0.342173	0.433108
H	6.340913	0.893989	1.513501
C	3.331402	-2.190138	-1.589646
H	3.815021	-1.323154	-2.030159
C	3.836048	0.717785	2.614123
H	4.341003	1.364059	1.898582
C	2.524967	1.423496	3.001808
H	1.940016	0.826151	3.704955
H	2.742267	2.384652	3.474115
H	1.914110	1.622944	2.119050
C	4.744154	0.564157	3.842334
H	5.662088	0.025056	3.602060
H	5.016374	1.547387	4.232546
H	4.239026	0.023165	4.644152
C	1.896108	-2.215860	-2.132115
H	1.332191	-3.056611	-1.723005
H	1.368982	-1.299873	-1.871228
H	1.898782	-2.310826	-3.220465
C	4.101491	-3.442073	-2.030268
H	3.599037	-4.354808	-1.703277
H	4.171600	-3.478378	-3.119639
H	5.113442	-3.459682	-1.623446
N	-0.143533	2.327907	-1.429408
N	1.029605	2.126913	-1.266540
N	-0.542516	0.421106	-0.220039
N	0.546944	0.186286	0.180526
Li	0.524916	-0.855305	1.988147
Cl	-0.044850	-1.293004	4.003513

Pdt_{LiCl}

112

C	2.912630	-3.639270	0.107318
C	2.979462	-2.936714	1.297215
C	3.253604	-1.570012	1.316854
C	3.482231	-0.935513	0.084437
C	3.354224	-1.611402	-1.142668
C	3.079891	-2.975863	-1.095321
N	3.899220	0.453959	0.092293
C	3.111451	1.490943	-0.027347
C	3.933249	2.775648	-0.001783

C	5.330932	2.291213	0.441761
C	5.387944	0.785494	0.173160
B	1.534151	1.498977	-0.201417
N	0.983139	2.769344	-0.584107
N	-0.310269	2.897676	-0.645029
B	-1.136912	1.768929	-0.321393
N	-0.596526	0.466983	-0.124770
N	0.729917	0.334850	-0.065396
C	3.958233	3.400499	-1.414060
C	3.379917	3.800382	1.001972
C	6.076943	0.439342	-1.149860
C	6.087773	0.023901	1.295814
C	3.184835	-0.828324	2.646102
C	4.033026	-1.484752	3.745492
C	3.377098	-0.922458	-2.502498
C	4.333533	-1.601216	-3.494476
C	-2.692089	2.086895	-0.272691
C	-3.248231	3.499382	-0.416504
C	-4.739441	3.334944	-0.044944
C	-5.071457	1.845013	-0.161748
N	-3.673557	1.235832	-0.120338
C	-5.940291	1.348745	0.991732
C	-5.771262	1.498599	-1.479041
C	-2.577441	4.498868	0.540485
C	-3.058112	3.971317	-1.873421
C	-3.512662	-0.193043	0.063442
C	-3.397323	-0.682328	1.375007
C	-3.314878	-2.064551	1.540161
C	-3.324648	-2.919970	0.452494
C	-3.387171	-2.405686	-0.831224
C	-3.470683	-1.034843	-1.061974
C	-3.261012	0.212164	2.601178
C	-4.236204	-0.166098	3.725598
C	-3.396965	-0.532527	-2.498913
C	-4.435442	-1.209032	-3.406700
C	-1.815507	0.199165	3.124967
C	-1.987398	-0.723701	-3.086238
C	1.959484	-0.857253	-3.100186
C	1.723573	-0.699899	3.107141
Li	-0.095028	-1.426915	-0.224900
Cl	-0.254693	-3.412526	-1.106405

H	-4.895129	3.662157	0.984496
H	-5.385223	3.940673	-0.679949
H	-6.072133	0.267527	0.958946
H	-5.525721	1.623415	1.958077
H	-6.922946	1.814566	0.905847
H	-1.540878	4.663504	0.258886
H	-3.122855	5.445561	0.499837
H	-2.609520	4.136234	1.570924
H	-3.530855	4.948699	-1.995992
H	-1.996835	4.053988	-2.098451
H	-3.515403	3.283491	-2.586703
H	-5.907463	0.423502	-1.579074
H	-6.756932	1.965805	-1.478142
H	-5.226203	1.866194	-2.345707
H	-3.212675	-2.474908	2.536574
H	-3.294049	-3.081901	-1.667770
H	-3.217943	-3.986405	0.596528
H	-3.476237	1.239783	2.308497
H	-3.596539	0.536738	-2.504441
H	-1.719386	0.858784	3.991095
H	-1.521880	-0.806739	3.431898
H	-1.120524	0.530000	2.356695
H	-4.185274	0.569561	4.531862
H	-5.266572	-0.217416	3.372533
H	-3.986032	-1.137257	4.156155
H	-1.256669	-0.121176	-2.550786
H	-1.663829	-1.763425	-3.021810
H	-1.979376	-0.416579	-4.135210
H	-5.444551	-1.153605	-2.995737
H	-4.440075	-0.733591	-4.390019
H	-4.196905	-2.263416	-3.555043
H	5.456252	2.472744	1.510597
H	6.129785	2.821846	-0.075400
H	2.782162	-3.452063	2.228121
H	2.926666	-3.518834	-2.016544
H	2.659592	-4.690179	0.109542
H	4.616927	4.272394	-1.404003
H	4.335927	2.701342	-2.161480
H	2.954176	3.707043	-1.698666
H	2.419264	4.184457	0.668286
H	3.257024	3.353766	1.991850

H	4.088797	4.627568	1.092335
H	7.131091	0.709108	-1.073242
H	6.014582	-0.628413	-1.350556
H	5.651002	0.978541	-1.992769
H	6.028203	-1.054311	1.149669
H	7.140839	0.309285	1.297403
H	5.675263	0.270661	2.270670
H	3.712508	0.104708	-2.369761
H	3.556785	0.185785	2.499043
H	1.281543	-1.685477	3.269869
H	1.665250	-0.142946	4.045558
H	1.127061	-0.183615	2.358589
H	5.063716	-1.644059	3.427529
H	4.042444	-0.855945	4.638770
H	3.624059	-2.454227	4.035133
H	1.508293	-1.848622	-3.163537
H	1.306297	-0.241319	-2.486763
H	1.996079	-0.422574	-4.102235
H	3.971746	-2.591120	-3.776530
H	4.405628	-1.008114	-4.408650
H	5.337822	-1.719378	-3.086108

With OEEF: Gas Phase

RC$_{OEEF}$

108

B	0.000000	0.000000	0.000000
N	0.000000	0.000000	-4.687963
C	0.562601	3.745620	-0.476459
C	1.004384	3.515067	0.983852
C	0.281911	1.405991	0.074277
C	0.403995	2.350010	-1.129555
H	1.266476	4.381872	-1.022620
H	-0.406350	4.253258	-0.471859
N	0.481784	2.167769	1.234401
C	2.541126	3.587054	1.119063
H	2.905752	4.576379	0.814654
H	2.844642	3.410820	2.150400
H	3.023637	2.838152	0.494208
C	1.620400	1.980787	-1.995377
H	1.508214	0.965774	-2.373430
H	1.714470	2.664810	-2.847948
H	2.547026	2.011376	-1.421209
C	-0.857173	2.298657	-2.002535
H	-0.787477	3.010065	-2.834959
H	-0.992578	1.301166	-2.415089
H	-1.746421	2.530333	-1.413444
C	0.398018	4.558269	1.930273
H	0.650648	4.336591	2.968824
H	0.789204	5.551426	1.679931
H	-0.686762	4.580486	1.845478
C	0.112661	1.687690	2.528336
C	1.054076	1.044033	3.351375
C	-1.227530	1.830903	2.951007
C	0.670832	0.673411	4.642091
C	-1.565036	1.434590	4.242530
C	-0.619777	0.883370	5.094910
H	1.376845	0.156379	5.279297
H	-2.593988	1.506330	4.572095
H	-0.907979	0.551704	6.084630
C	2.417565	0.617306	2.836965
H	2.530920	1.016514	1.831019
C	-2.325465	2.292756	2.006005
H	-1.847460	2.682317	1.107649
C	2.474797	-0.914522	2.714123
H	3.419631	-1.226093	2.263651
H	2.392231	-1.398109	3.689264
H	1.660246	-1.271197	2.084933
C	3.573240	1.135516	3.703984
H	4.532829	0.871757	3.252652
H	3.535809	2.222535	3.826597
H	3.550128	0.690162	4.700553
C	-3.194887	1.098471	1.578631
H	-2.585205	0.318353	1.126853
H	-3.716762	0.661268	2.431104
H	-3.944425	1.406469	0.845547
C	-3.198703	3.409706	2.598001
H	-2.598389	4.259465	2.938606
H	-3.911354	3.765127	1.847710
H	-3.778790	3.051748	3.450860
B	-0.279675	-1.455696	-0.155802
C	-0.455951	-2.905396	-0.348050
C	0.677163	-3.848316	-0.754959
C	-0.095623	-5.105689	-1.216219

C	-1.482523	-5.074328	-0.548951
H	0.439132	-6.023138	-0.975835
H	-0.216801	-5.077165	-2.299777
N	-1.581237	-3.595686	-0.189034
C	-2.776335	-2.990286	0.341456
C	-2.971401	-2.960780	1.737084
C	-3.721126	-2.434232	-0.540926
C	-4.183597	-2.465993	2.216455
C	-4.913237	-1.948187	-0.005336
C	-5.158090	-1.988114	1.356881
H	-4.368828	-2.471447	3.282379
H	-5.667933	-1.550074	-0.671184
H	-6.108463	-1.647572	1.749290
C	1.540992	-3.270308	-1.881109
H	0.931724	-3.016929	-2.749186
H	2.059222	-2.366497	-1.551431
H	2.287269	-4.005590	-2.187770
C	1.566327	-4.134326	0.471357
H	2.077832	-3.227657	0.796918
H	0.992425	-4.518852	1.312575
H	2.316661	-4.882876	0.211688
C	-2.590977	-5.480933	-1.516427
H	-2.473310	-6.535798	-1.764635
H	-3.576329	-5.360441	-1.065805
H	-2.547624	-4.915366	-2.445203
C	-1.575478	-5.961531	0.693701
H	-2.536189	-5.834494	1.190096
H	-1.503513	-7.005300	0.388968
H	-0.779278	-5.772043	1.409689
C	-3.468744	-2.271646	-2.031739
H	-2.532773	-2.770860	-2.274342
C	-1.902188	-3.366326	2.739170
H	-1.079298	-3.817313	2.192815
C	-1.341289	-2.116353	3.436661
H	-0.530551	-2.396139	4.113011
H	-0.952797	-1.399222	2.712392
H	-2.114245	-1.622281	4.029785
C	-2.396248	-4.385343	3.774325
H	-2.837211	-5.264195	3.307977
H	-1.565239	-4.721650	4.396314
H	-3.146967	-3.952096	4.437863
C	-3.277007	-0.782337	-2.365758
H	-4.188755	-0.216148	-2.154861
H	-2.462578	-0.359370	-1.776587
H	-3.042163	-0.661140	-3.426503
C	-4.575219	-2.883928	-2.899847
H	-4.300139	-2.830406	-3.955232
H	-4.752878	-3.928956	-2.653961
H	-5.520444	-2.351094	-2.777167
N	-0.296755	-0.989132	-4.335694

TS_{1OEEF}

108

B	0.000000	0.000000	0.000000
N	0.000000	0.000000	2.151445
N	0.354804	0.000000	3.187504
B	1.502165	-0.111491	-0.237075
C	4.999282	-1.245022	-1.259318
C	4.985692	0.283567	-1.497766
C	2.887500	-0.298659	-0.517401
C	3.553150	-1.648830	-0.857072
H	5.689257	-1.466704	-0.438433
H	5.348158	-1.795723	-2.137722
N	3.912039	0.695488	-0.591173
C	6.327471	0.934512	-1.149768
H	6.625201	0.710115	-0.125221
H	7.101071	0.560445	-1.829225
H	6.271021	2.020299	-1.255288
C	3.569847	-2.590936	0.359209
H	4.088231	-3.529639	0.124738
H	4.066065	-2.126853	1.212516
H	2.549574	-2.830371	0.659940
C	2.829533	-2.367995	-2.005014
H	1.801671	-2.588250	-1.719088
H	2.789943	-1.752792	-2.903681
H	3.328603	-3.312345	-2.251495
C	4.658224	0.591882	-2.975249
H	4.696410	1.662950	-3.169656
H	5.368743	0.094525	-3.644854
H	3.656753	0.242078	-3.220166
C	3.887623	1.891437	0.174963
C	4.412004	1.874072	1.490358
C	3.312967	3.075215	-0.328118

C	4.393079	3.040761	2.249545
C	3.329587	4.226752	0.463554
C	3.865049	4.218221	1.738760
H	4.755789	3.022561	3.270975
H	2.869226	5.132037	0.089135
H	3.826050	5.111609	2.350851
C	4.911088	0.586727	2.121319
H	5.049050	-0.130554	1.315761
C	2.627683	3.132391	-1.680353
H	2.621027	2.121405	-2.083892
C	3.849080	0.008580	3.065776
H	2.927297	-0.174503	2.517133
H	4.181401	-0.939547	3.498842
H	3.628108	0.700739	3.881362
C	6.260963	0.743431	2.836855
H	7.015673	1.178702	2.173783
H	6.179315	1.386332	3.717065
H	6.619284	-0.233763	3.177515
C	1.164524	3.577409	-1.550856
H	0.681722	3.572750	-2.528494
H	0.617306	2.897178	-0.900786
H	1.080360	4.587305	-1.145267
C	3.384198	4.050767	-2.653113
H	3.339809	5.090450	-2.318703
H	4.439375	3.773273	-2.735596
H	2.936095	4.004034	-3.648069
C	-1.403243	0.117595	-0.486889
C	-1.649975	-0.035235	-2.004201
C	-3.189515	-0.133445	-2.084261
C	-3.761688	0.494601	-0.811343
H	-3.481482	-1.183372	-2.125866
H	-3.583217	0.346632	-2.978963
N	-2.558974	0.349862	0.124374
C	-2.695944	0.518885	1.549093
C	-3.033226	-0.607505	2.325688
C	-2.487696	1.785496	2.129226
C	-3.223396	-0.420964	3.692996
C	-2.694662	1.910048	3.501850
C	-3.073874	0.826385	4.276784
H	-3.512163	-1.262236	4.308014
H	-2.572390	2.877533	3.970568

H	-3.266329	0.955450	5.334064
C	-1.099491	1.178712	-2.770983
H	-1.377672	1.100560	-3.824059
H	-1.494316	2.118297	-2.388772
H	-0.012309	1.209547	-2.694170
C	-1.007319	-1.309216	-2.570925
H	-1.288387	-2.184324	-1.983995
H	-1.352532	-1.461530	-3.596309
H	0.079550	-1.231670	-2.572215
C	-4.124114	1.972393	-0.985596
H	-4.940459	2.051740	-1.703033
H	-4.476252	2.396382	-0.048171
H	-3.292077	2.570345	-1.352772
C	-4.999666	-0.239786	-0.305418
H	-5.315075	0.125925	0.671153
H	-5.820079	-0.055938	-0.999180
H	-4.853268	-1.314760	-0.253699
C	-1.995015	3.002834	1.357261
H	-1.951298	2.743997	0.301972
C	-3.139077	-2.015864	1.756239
H	-3.116664	-1.947886	0.670501
C	-1.921057	-2.862725	2.166210
H	-1.840429	-2.927804	3.253774
H	-2.028708	-3.877505	1.776870
H	-0.992906	-2.443874	1.773026
C	-4.442464	-2.719792	2.160755
H	-4.458379	-2.957713	3.225165
H	-5.318851	-2.109752	1.948860
H	-4.547624	-3.659610	1.616763
C	-0.565361	3.386574	1.780803
H	-0.522361	3.612511	2.848526
H	0.146182	2.588812	1.566867
H	-0.242855	4.277814	1.236691
C	-2.928310	4.212593	1.513978
H	-2.904298	4.603988	2.532272
H	-2.620578	5.017509	0.843214
H	-3.963763	3.966279	1.287835

TS_{2OEEF}

110

B	0.000000	0.000000	0.000000
N	0.000000	0.000000	1.916160

C	3.218204	0.000000	-2.003673
C	3.678575	0.714270	-0.720359
C	1.357416	0.117013	-0.459611
C	1.671613	-0.034983	-1.966895
H	3.602569	-1.024150	-1.988052
H	3.597307	0.480128	-2.909983
N	2.599633	0.352308	0.216254
C	5.045021	0.203202	-0.252734
H	5.030802	-0.876600	-0.109136
H	5.799698	0.446751	-1.008168
H	5.338287	0.665926	0.692816
C	1.174799	-1.371321	-2.544157
H	1.533841	-1.501989	-3.571364
H	1.518675	-2.217800	-1.947682
H	0.088150	-1.402585	-2.564567
C	1.049370	1.091568	-2.808907
H	-0.035911	1.036075	-2.747220
H	1.343000	2.081508	-2.461479
H	1.337708	0.992564	-3.860828
C	3.787103	2.238073	-0.961525
H	4.229443	2.746465	-0.105730
H	4.412343	2.441843	-1.837319
H	2.804948	2.668848	-1.141874
C	2.834025	-0.024643	1.561425
C	2.923215	-1.394729	1.914555
C	2.953206	0.960187	2.568109
C	3.163429	-1.738353	3.241848
C	3.220529	0.567297	3.880566
C	3.326727	-0.769754	4.223456
H	3.198451	-2.785271	3.519103
H	3.285809	1.321088	4.656575
H	3.482647	-1.059523	5.256214
C	2.729654	-2.503710	0.895536
H	2.635644	-2.035060	-0.079595
C	2.716443	2.432905	2.281179
H	2.452867	2.512642	1.229620
C	1.432705	-3.282443	1.155169
H	0.576539	-2.613176	1.169819
H	1.256518	-4.016627	0.367078
H	1.470456	-3.810325	2.111022
C	3.929599	-3.463056	0.837966

H	4.867093	-2.927666	0.657818
H	4.038457	-4.023297	1.770339
H	3.786937	-4.188740	0.032842
C	1.536124	3.001074	3.083179
H	1.361542	4.044485	2.811668
H	0.620359	2.451026	2.879730
H	1.717798	2.960584	4.159529
C	3.979998	3.270807	2.536286
H	4.229692	3.277570	3.601455
H	4.842703	2.872187	1.995107
H	3.824305	4.306986	2.223368
B	-1.544375	-0.435448	-0.353468
C	-2.843076	0.370409	-0.342176
C	-2.881758	1.894091	-0.176712
C	-4.343583	2.245954	-0.529442
C	-5.179530	0.978821	-0.324223
H	-4.399406	2.543894	-1.577540
H	-4.724454	3.076606	0.062878
N	-4.087107	-0.083136	-0.426450
C	-4.413524	-1.483110	-0.561945
C	-4.570438	-2.014219	-1.856442
C	-4.550210	-2.281766	0.589430
C	-4.937962	-3.352895	-1.969850
C	-4.922273	-3.613484	0.412288
C	-5.133175	-4.143370	-0.849262
H	-5.093506	-3.780097	-2.951218
H	-5.065280	-4.243395	1.279891
H	-5.458828	-5.169571	-0.959192
C	-2.532575	2.276482	1.274533
H	-2.726326	3.339839	1.429940
H	-3.122872	1.719591	2.000448
H	-1.477914	2.089736	1.476916
C	-1.919977	2.619130	-1.125242
H	-2.094584	2.321142	-2.160291
H	-2.086344	3.697140	-1.052843
H	-0.880112	2.406955	-0.876355
C	-5.863011	0.931414	1.045149
H	-6.605037	1.728006	1.093728
H	-6.391043	-0.009013	1.182005
H	-5.168408	1.068619	1.871363
C	-6.254923	0.812740	-1.394282

H	-6.754479	-0.151952	-1.314697
H	-7.013192	1.582235	-1.249133
H	-5.858268	0.927674	-2.399503
C	-4.257690	-1.794443	2.001846
H	-4.054478	-0.727011	1.959993
C	-4.306849	-1.219650	-3.128766
H	-4.152485	-0.177312	-2.855021
C	-3.010413	-1.692014	-3.809486
H	-3.076875	-2.745903	-4.086822
H	-2.835757	-1.113986	-4.720681
H	-2.149500	-1.565871	-3.151972
C	-5.479832	-1.283320	-4.117819
H	-5.596552	-2.283502	-4.536917
H	-6.424747	-1.015858	-3.648785
H	-5.310507	-0.598013	-4.950564
C	-2.991551	-2.464927	2.563863
H	-3.120188	-3.546837	2.631091
H	-2.122027	-2.266172	1.937219
H	-2.784696	-2.087931	3.568306
C	-5.436220	-2.019614	2.960952
H	-5.599341	-3.081558	3.149091
H	-5.238643	-1.542485	3.922661
H	-6.368554	-1.615322	2.571957
N	-1.460889	-2.963832	-0.744627
N	-1.585689	-1.862262	-0.558859
N	-0.381610	-0.219548	2.926994

TS_{3OEEF}

B	0.000000	0.000000	0.000000
N	0.000000	0.000000	2.006630
C	5.911157	0.000000	3.681363
C	5.040703	0.362964	4.901826
C	3.644496	0.199488	2.929810
C	5.071963	0.282152	2.412681
H	6.141628	-1.068550	3.720059
H	6.862872	0.543576	3.682420
N	3.689949	0.266961	4.341105
C	5.265295	-0.622385	6.059831
H	4.557517	-0.431701	6.867657
H	5.132963	-1.651287	5.732332
H	6.279751	-0.511946	6.459920
C	5.379161	-0.762149	1.328071
H	4.757507	-0.593567	0.448989
H	6.437294	-0.712795	1.029595
H	5.164713	-1.767044	1.696821
C	5.360260	1.683557	1.832505
H	6.412756	1.778196	1.520951
H	4.720687	1.855043	0.965971
H	5.145814	2.467065	2.558734
C	5.382423	1.771751	5.438679
H	4.709453	2.037552	6.252427
H	6.409896	1.790909	5.823432
H	5.292417	2.532438	4.666636
C	2.487168	0.299288	5.114502
C	1.905101	-0.914585	5.538479
C	1.821268	1.522777	5.344806
C	0.719556	-0.871865	6.270873
C	0.636432	1.512362	6.079808
C	0.092220	0.330064	6.553553
H	0.254546	-1.794498	6.593889
H	0.107772	2.440243	6.256497
H	-0.838516	0.342134	7.105702
C	2.467222	-2.272751	5.144497
H	3.387935	-2.097912	4.587950
C	2.290361	2.840950	4.747343
H	3.219637	2.648966	4.215763
C	1.508371	-3.013998	4.199623
H	1.934444	-3.975033	3.898247
H	0.548424	-3.212143	4.678331
H	1.316562	-2.433575	3.300864
C	2.786965	-3.149791	6.365057
H	3.267761	-4.081145	6.049677
H	3.445924	-2.640988	7.071474
H	1.877632	-3.421565	6.903188
C	1.288748	3.372623	3.710241
H	1.142089	2.657720	2.904636
H	0.315216	3.575782	4.157118
H	1.653970	4.305468	3.272379
C	2.549164	3.909567	5.820440
H	3.232868	3.555645	6.594883
H	2.976105	4.809093	5.367077
H	1.621931	4.204122	6.314137
B	2.466216	0.140135	2.073030

C	-1.256206	-0.116422	-0.794100
C	-1.244338	-0.004723	-2.318534
C	-2.648722	-0.528095	-2.695519
C	-3.549776	-0.368714	-1.459608
H	-2.581690	-1.585601	-2.955500
H	-3.061561	-0.006731	-3.557857
N	-2.496217	-0.286555	-0.358060
C	-2.829548	-0.412140	1.043876
C	-2.780526	-1.688398	1.633037
C	-3.149392	0.736978	1.786424
C	-3.128160	-1.796726	2.977885
C	-3.495506	0.565989	3.126665
C	-3.501497	-0.685981	3.716588
H	-3.129205	-2.770503	3.449312
H	-3.780069	1.428791	3.713515
H	-3.810047	-0.797845	4.747974
C	-1.041927	1.469632	-2.719059
H	-1.119543	1.570463	-3.803925
H	-1.792045	2.117255	-2.268887
H	-0.058349	1.820761	-2.401052
C	-0.143035	-0.858952	-2.957637
H	0.848449	-0.495628	-2.680391
H	-0.226198	-1.900783	-2.643993
H	-0.238201	-0.821044	-4.045580
C	-4.409430	0.897472	-1.509372
H	-5.127617	0.798708	-2.323129
H	-4.978529	1.019311	-0.590845
H	-3.827270	1.799464	-1.685381
C	-4.478180	-1.564533	-1.259787
H	-5.017339	-1.497903	-0.315492
H	-5.220778	-1.567918	-2.057615
H	-3.944304	-2.510741	-1.299440
C	-3.074055	2.151330	1.230685
H	-2.892548	2.089595	0.161052
C	-2.321433	-2.938106	0.894478
H	-2.199713	-2.689433	-0.158435
C	-0.943623	-3.395332	1.401575
H	-0.982145	-3.638939	2.465012
H	-0.621794	-4.290515	0.862326
H	-0.190312	-2.619219	1.257946
C	-3.337466	-4.084963	0.992357
H	-4.334445	-3.771155	0.688072
H	-3.034253	-4.916157	0.352217
H	-3.413820	-4.466729	2.011355
C	-1.878179	2.906705	1.833871
H	-1.964218	2.971105	2.920338
H	-0.936247	2.410376	1.597056
H	-1.840123	3.924725	1.439038
C	-4.372257	2.942539	1.446339
H	-4.537142	3.163916	2.501556
H	-4.327704	3.895188	0.916126
H	-5.247687	2.401778	1.090193
N	2.370972	0.204876	0.591864
N	1.319843	0.108303	-0.032340
N	1.033439	0.057865	2.503922

With explicit ILs :

$[C_4mpyr]^+[eFAP]^-$

55

N	1.428068	-1.579785	-0.005160
C	0.222116	-1.468905	0.940320
C	-0.927211	-2.276696	0.324617
C	-0.337798	-3.015504	-0.891756
C	0.883514	-2.202319	-1.289840
H	1.683097	-2.768573	-1.763266
H	-0.071493	-4.040156	-0.638126
H	-1.350130	-2.972199	1.048746
H	0.537711	-1.831373	1.915228
H	-0.026513	-0.418305	1.021241
H	-1.712901	-1.595147	0.011846
H	-1.041729	-3.048792	-1.721576
H	0.605042	-1.380702	-1.934698
C	1.998891	-0.217532	-0.295954
H	2.802659	-0.326414	-1.020864
H	1.221461	0.417823	-0.704438
H	2.382892	0.203662	0.630517
C	2.536321	-2.412818	0.608381
H	2.819720	-1.896150	1.526752
C	2.217718	-3.870304	0.901793
H	3.379479	-2.347754	-0.080615
H	2.033516	-4.411588	-0.028250
H	1.310743	-3.955523	1.505574
C	3.382895	-4.535263	1.642347

C	3.126530	-6.015506	1.916057
H	4.298456	-4.424104	1.052293
H	3.559392	-4.010435	2.586853
H	3.965925	-6.467983	2.445602
H	2.232198	-6.153508	2.528154
H	2.982594	-6.568629	0.984979
P	-1.447277	1.542045	-2.176674
F	-2.016835	2.727140	-3.113271
C	-0.411989	0.761967	-3.746016
C	-0.753565	-0.450453	-4.667731
F	-0.183688	1.784347	-4.606133
F	0.828524	0.383023	-3.248647
F	0.200941	-0.567087	-5.610644
F	-0.746909	-1.612056	-3.978817
F	-1.917822	-0.318352	-5.294792
F	-0.019319	2.298803	-1.920213
C	-3.128044	0.502298	-2.344568
F	-2.846371	-0.727552	-2.869490
F	-3.642119	0.243068	-1.108478
C	-4.303119	1.060098	-3.203761
F	-4.780052	2.206654	-2.724045
F	-3.922438	1.250958	-4.470842
F	-5.311342	0.165474	-3.207700
C	-2.061529	2.560253	-0.584655
F	-1.565440	3.825729	-0.678696
F	-3.412532	2.697763	-0.518156
C	-1.624730	2.067344	0.824899
F	-0.281763	1.958001	0.916580
F	-2.020187	2.931551	1.768217
F	-2.148580	0.868979	1.134914
F	-0.920255	0.254165	-1.237395

RC_{IL} (with $[C_4mpyr]^+[eFAP]^-$)

163

N	-0.196504	-2.340258	-0.529950
C	0.434153	-1.071795	-1.091519
C	0.333929	-1.215156	-2.603618
C	-0.920781	-2.094822	-2.840418
C	-1.386719	-2.544753	-1.449582
H	-2.178487	-1.905676	-1.080666
H	-1.721146	-1.528403	-3.311428
H	0.235956	-0.232155	-3.056535
H	-0.180104	-0.249175	-0.737575
H	1.442155	-0.959049	-0.697793
H	1.229803	-1.676990	-3.017893
H	-0.689451	-2.944532	-3.482065
H	-1.703955	-3.582494	-1.388057
C	0.750472	-3.489375	-0.647434
H	0.230490	-4.409861	-0.401610
H	1.125922	-3.546246	-1.663888
H	1.577565	-3.335027	0.037501
C	-0.588129	-2.114071	0.916979
H	-1.184565	-1.207297	0.920117
C	-1.344460	-3.254202	1.581376
H	0.345501	-1.914122	1.443424
H	-0.818624	-4.202163	1.445329
H	-2.332943	-3.355012	1.129326
C	-1.490635	-3.001724	3.091506
C	-2.292902	-1.752248	3.456505
H	-1.970210	-3.880381	3.530661
H	-0.491317	-2.946066	3.537380
H	-3.292228	-1.783383	3.023755
H	-2.395273	-1.669804	4.539798
H	-1.818718	-0.834782	3.104093
P	-3.460086	1.167652	-0.528319
F	-2.124919	0.745901	0.376902
C	-2.238213	2.265971	-1.636520
C	-2.793912	2.842775	-2.970760
F	-1.680798	3.308814	-0.966873
F	-1.166368	1.482805	-2.012878
F	-3.749331	3.745587	-2.763466
F	-1.797341	3.454035	-3.647054
F	-3.269684	1.869732	-3.756250
F	-3.139794	-0.028876	-1.622300
C	-3.941929	2.630102	0.724753
F	-3.965889	3.808042	0.041765
F	-5.215618	2.432170	1.162609
C	-3.093566	2.904127	2.003636
F	-1.835885	3.253675	1.699648
F	-3.639027	3.920178	2.694178
F	-3.039506	1.843786	2.817264
C	-4.460730	-0.231629	0.457676
F	-3.716468	-1.397112	0.428756

F	-4.624854	0.036938	1.777022
C	-5.860116	-0.651463	-0.076485
F	-5.797982	-1.034374	-1.356806
F	-6.311184	-1.703789	0.639499
F	-6.750900	0.330487	0.043730
F	-4.766334	1.605399	-1.387767
C	6.445073	-2.461016	-2.194752
C	7.460770	-2.287035	-1.053410
C	5.637048	-0.775818	-0.666594
C	5.147265	-1.749365	-1.750097
H	6.279031	-3.511774	-2.437931
H	6.834394	-1.975227	-3.091376
N	6.959386	-1.027403	-0.429328
C	7.415614	-3.452679	-0.051005
H	8.137823	-3.295973	0.748646
H	6.431757	-3.561312	0.401374
H	7.667761	-4.388393	-0.554742
C	4.118429	-2.733185	-1.177484
H	3.751137	-3.401918	-1.961926
H	4.532667	-3.345002	-0.377460
H	3.283490	-2.171517	-0.764088
C	4.500857	-0.994348	-2.919663
H	5.169761	-0.225421	-3.307982
H	4.260084	-1.687006	-3.731671
H	3.582787	-0.499674	-2.602042
C	8.893575	-2.163400	-1.572397
H	8.985699	-1.389917	-2.331186
H	9.590094	-1.937616	-0.764234
H	9.192027	-3.112401	-2.021501
C	7.811443	-0.136637	0.300232
C	7.972030	-0.270942	1.689669
C	8.493212	0.869739	-0.417069
C	8.912948	0.539805	2.327466
C	9.419397	1.653700	0.266370
C	9.649313	1.475698	1.622267
H	9.055849	0.446823	3.396499
H	9.957136	2.427880	-0.265003
H	10.377199	2.092661	2.134908
C	7.098628	-1.188246	2.530270
H	6.470567	-1.763903	1.853277
C	8.183954	1.177673	-1.874759
H	7.625587	0.337244	-2.285019
C	6.158740	-0.349228	3.412490
H	6.725132	0.255703	4.124485
H	5.551235	0.316213	2.799374
H	5.491647	-0.999065	3.984123
C	7.908250	-2.170161	3.387776
H	7.239028	-2.870789	3.892606
H	8.615739	-2.746400	2.789751
H	8.478084	-1.646784	4.158241
C	7.271564	2.410907	-1.967445
H	6.352316	2.266705	-1.401306
H	7.774706	3.295328	-1.573545
H	7.004360	2.612262	-3.007672
C	9.439522	1.371062	-2.735261
H	9.976472	2.282245	-2.463784
H	10.134914	0.535778	-2.636195
H	9.160910	1.462797	-3.787483
B	3.897615	1.268435	0.526184
B	4.799419	0.229118	-0.039291
C	3.056834	2.267427	1.177626
C	2.049635	1.959765	2.290230
C	1.659998	3.365519	2.801090
C	1.994557	4.368895	1.682726
H	2.254863	3.604765	3.685265
H	0.609762	3.419155	3.085983
N	3.016738	3.593075	0.904875
C	3.889719	4.195389	-0.063157
C	5.133984	4.698357	0.369377
C	3.511544	4.255883	-1.417378
C	5.940082	5.351427	-0.560454
C	4.361697	4.912131	-2.308939
C	5.553008	5.475742	-1.885118
H	6.897022	5.748573	-0.246988
H	4.086757	4.973168	-3.354114
H	6.194740	5.986312	-2.592934
C	0.841713	1.204689	1.712039
H	1.168911	0.231787	1.351358
H	0.077505	1.058912	2.479236
H	0.377132	1.733559	0.882669
C	2.678002	1.115136	3.405092
H	1.956328	0.958352	4.211247

H	2.988146	0.141455	3.023863
H	3.559960	1.604591	3.820157
C	0.778440	4.713440	0.815735
H	1.066408	5.382396	0.005921
H	0.309964	3.833907	0.385558
H	0.027930	5.221239	1.422136
C	2.562060	5.673526	2.244702
H	3.375644	5.494240	2.943043
H	2.922368	6.325136	1.447268
H	1.770808	6.202381	2.778080
C	2.261337	3.581581	-1.958385
H	1.689344	3.202200	-1.115791
C	5.662392	4.479594	1.778598
H	4.855868	4.061999	2.378664
C	6.792845	3.441084	1.766806
H	7.132834	3.234067	2.783834
H	6.460975	2.503543	1.322705
H	7.650662	3.801747	1.196926
C	6.134117	5.778130	2.448465
H	6.400913	5.587660	3.490728
H	7.020675	6.180749	1.954539
H	5.364322	6.550554	2.428277
C	2.638319	2.363939	-2.816342
H	3.195219	2.663608	-3.707856
H	3.252917	1.672105	-2.241581
H	1.733811	1.844505	-3.143685
C	1.363595	4.541858	-2.748996
H	1.846987	4.867814	-3.672618
H	0.430929	4.048350	-3.018505
H	1.117367	5.433876	-2.172076
N	3.019974	-2.546006	2.181687
N	3.942875	-2.912394	2.632780

Ts_{1IL} (with $[C_4mpyr]^+[eFAP]^-$)

163

N	-1.890505	-2.943812	3.379905
C	-1.321063	-3.101003	1.976246
C	-2.043070	-4.302178	1.368834
C	-3.366565	-4.437708	2.163082
C	-3.352247	-3.303326	3.186719
H	-3.833447	-2.414186	2.795160
H	-4.237706	-4.324824	1.522850
H	-2.226478	-4.111587	0.314792
H	-1.566613	-2.190257	1.443328
H	-0.241901	-3.190437	2.050247
H	-1.442566	-5.208727	1.441503
H	-3.434404	-5.410583	2.650064
H	-3.789226	-3.556490	4.149196
C	-1.217645	-3.905123	4.303112
H	-1.708082	-3.884653	5.271559
H	-1.281894	-4.906051	3.885388
H	-0.173139	-3.618209	4.406581
C	-1.716805	-1.513748	3.846667
H	-2.185768	-0.905766	3.080514
C	-2.310323	-1.199331	5.211430
H	-0.644343	-1.317671	3.829111
H	-1.811883	-1.779149	5.992134
H	-3.369890	-1.468145	5.228003
C	-2.175220	0.296067	5.541536
C	-2.995181	1.215196	4.635168
H	-2.488910	0.435044	6.579355
H	-1.118455	0.578183	5.502109
H	-2.924242	2.249224	4.976279
H	-2.658664	1.192419	3.600021
H	-4.050205	0.932965	4.637597
P	-4.478693	-0.506887	0.205771
F	-3.587065	-1.910980	0.301593
C	-6.080294	-1.580560	0.666854
C	-7.401289	-0.820971	0.985721
F	-6.384075	-2.516836	-0.266803
F	-5.815564	-2.300994	1.812633
F	-7.887447	-0.190575	-0.082337
F	-8.334143	-1.702086	1.400389
F	-7.218611	0.065787	1.971459
F	-4.317390	-0.276898	1.835655
C	-4.698329	-0.705048	-1.758085
F	-6.025048	-0.734266	-2.057049
F	-4.208637	0.407900	-2.373958
C	-4.068195	-1.917767	-2.511190
F	-2.733684	-1.942460	-2.393314
F	-4.541439	-3.087747	-2.067519
F	-4.357770	-1.831099	-3.820500
C	-2.760435	0.484213	0.073528

F	-1.933522	0.068861	1.101537
F	-2.053805	0.226537	-1.053897
C	-2.809276	2.033296	0.219315
F	-1.546845	2.521756	0.259277
F	-3.427113	2.614448	-0.805047
F	-3.406097	2.397227	1.360284
F	-5.344144	0.862931	0.080577
C	1.207322	-2.193646	-2.030434
C	1.820376	-3.419644	-2.746217
C	3.205134	-3.664902	-2.128833
C	2.446606	-1.483968	-1.449199
H	1.177086	-4.298127	-2.677365
H	1.943302	-3.188368	-3.806184
N	3.551265	-2.295410	-1.663086
C	3.129209	-4.663755	-0.958312
H	2.725941	-5.617172	-1.307482
H	4.117837	-4.852104	-0.543752
H	2.489262	-4.293003	-0.159147
C	0.241767	-2.595657	-0.903881
H	-0.128517	-1.702593	-0.400796
H	-0.615856	-3.134962	-1.312439
H	0.729139	-3.229138	-0.162718
C	0.435929	-1.333938	-3.039679
H	-0.310291	-1.943346	-3.555716
H	-0.090437	-0.522966	-2.544035
H	1.109781	-0.904193	-3.782249
C	4.204821	-4.205281	-3.151877
H	5.204548	-4.289660	-2.723264
H	3.889046	-5.199203	-3.474103
H	4.262011	-3.567923	-4.030997
C	4.894606	-1.816079	-1.590295
C	5.669180	-1.999741	-0.429559
C	5.439183	-1.165782	-2.721186
C	7.013848	-1.623905	-0.457862
C	6.780445	-0.793517	-2.692768
C	7.572852	-1.042614	-1.581944
H	7.625312	-1.769934	0.423697
H	7.213216	-0.297096	-3.551858
H	8.617628	-0.756357	-1.584099
C	5.083206	-2.517106	0.874081
H	4.040032	-2.766923	0.689931

C	4.595157	-0.812823	-3.936215
H	3.656548	-1.357908	-3.858687
C	5.103750	-1.419458	1.950896
H	4.631216	-1.780506	2.867746
H	6.127101	-1.125834	2.195179
H	4.569091	-0.532094	1.613479
C	5.802049	-3.773487	1.386373
H	5.284171	-4.180470	2.258321
H	5.851810	-4.553060	0.624994
H	6.827044	-3.546193	1.687752
C	4.252120	0.684071	-3.925062
H	3.740781	0.962836	-3.004869
H	5.156529	1.289873	-4.008599
H	3.598876	0.934847	-4.764339
C	5.255318	-1.204368	-5.265494
H	5.562207	-2.251721	-5.276117
H	4.556891	-1.044075	-6.089908
H	6.140867	-0.598683	-5.469476
B	2.167488	0.776943	0.377069
B	2.414044	-0.222923	-0.763195
C	2.008726	2.098207	1.025793
C	1.580359	2.410005	2.475338
C	1.225256	3.908922	2.402155
C	1.998410	4.511868	1.229738
H	1.436712	4.422045	3.341250
H	0.155491	4.005344	2.209078
N	2.284037	3.282847	0.412343
C	2.923160	3.426938	-0.872252
C	4.331986	3.413977	-0.951528
C	2.138758	3.660784	-2.019107
C	4.927684	3.743302	-2.168017
C	2.787575	3.984381	-3.210680
C	4.167826	4.053926	-3.283072
H	6.007360	3.738456	-2.246281
H	2.197914	4.168982	-4.099251
H	4.651822	4.311743	-4.217168
C	0.344461	1.664467	3.001777
H	-0.429268	1.595894	2.241769
H	0.576639	0.662417	3.350746
H	-0.059870	2.213500	3.856050
C	2.766269	2.153358	3.427796

H	3.031135	1.096537	3.443946
H	3.655633	2.708918	3.135569
H	2.496602	2.456861	4.443364
C	1.139384	5.528132	0.473715
H	0.934010	6.374502	1.131958
H	1.654924	5.905951	-0.409087
H	0.187113	5.100042	0.169854
C	3.285859	5.222906	1.666965
H	3.841298	5.574242	0.798290
H	3.026105	6.092337	2.273205
H	3.936931	4.583209	2.256522
C	0.632051	3.470037	-2.036411
H	0.300116	3.326473	-1.011990
C	5.227106	2.972549	0.195766
H	4.606381	2.842600	1.079146
C	5.845261	1.605758	-0.126086
H	6.419585	1.235085	0.725226
H	5.076448	0.873485	-0.363113
H	6.519812	1.670764	-0.980732
C	6.336998	3.979687	0.531532
H	5.947136	4.977362	0.729909
H	6.886427	3.647165	1.415652
H	7.057458	4.061739	-0.284936
C	0.297360	2.178518	-2.793745
H	0.549992	2.267425	-3.853362
H	0.863745	1.348097	-2.379630
H	-0.766767	1.949542	-2.713098
C	-0.141606	4.651426	-2.636638
H	-1.214958	4.484483	-2.522377
H	0.108729	5.598106	-2.158235
H	0.058615	4.755924	-3.705144
N	1.750717	-1.521721	2.235476
N	1.820572	-0.521084	1.764602

Int_{1IL} (with $[C_4mpyr]^+[eFAP]^-$)

163

N	-1.777474	-3.231319	-0.403514
C	-1.353124	-1.926519	-1.063237
C	-1.582871	-2.140444	-2.556950
C	-2.686081	-3.225490	-2.652682
C	-3.003363	-3.619669	-1.208272
H	-3.827008	-3.032320	-0.818227
H	-3.587549	-2.840635	-3.124276
H	-1.898487	-1.205811	-3.010105
H	-2.012987	-1.159013	-0.673195
H	-0.329018	-1.700995	-0.777767
H	-0.668527	-2.457750	-3.056990
H	-2.345905	-4.081375	-3.235521
H	-3.205750	-4.676444	-1.055011
C	-0.693113	-4.250815	-0.559747
H	-1.043881	-5.209520	-0.189591
H	-0.432580	-4.340067	-1.609860
H	0.174059	-3.924073	0.008171
C	-2.087319	-2.993096	1.060936
H	-2.726329	-2.116214	1.086213
C	-2.747935	-4.154206	1.789026
H	-1.133445	-2.745068	1.523716
H	-2.201007	-5.084171	1.615344
H	-3.765119	-4.294864	1.417696
C	-2.785793	-3.892079	3.303565
C	-3.587031	-2.656165	3.716248
H	-3.212094	-4.776063	3.784800
H	-1.757834	-3.809221	3.672584
H	-4.612371	-2.707393	3.349979
H	-3.619424	-2.569924	4.803566
H	-3.153182	-1.732082	3.329840
P	-5.006429	0.314877	-0.267781
F	-3.636679	0.019712	0.626913
C	-3.885674	1.437274	-1.458390
C	-4.504846	1.899292	-2.809123
F	-3.390643	2.553260	-0.859459
F	-2.766654	0.714927	-1.815497
F	-5.520889	2.739376	-2.631162
F	-3.566615	2.544525	-3.536748
F	-4.917280	0.851930	-3.532336
F	-4.621085	-0.915743	-1.304935
C	-5.552174	1.813808	0.917027
F	-5.683233	2.945052	0.169157
F	-6.792941	1.555072	1.413035
C	-4.686135	2.226856	2.146786
F	-3.472565	2.665164	1.777816
F	-5.291874	3.229465	2.803884
F	-4.513156	1.221037	3.011787

C	-5.904618	-1.083822	0.810742
F	-5.113480	-2.217618	0.799168
F	-6.024184	-0.764107	2.123051
C	-7.300934	-1.587809	0.347735
F	-7.260381	-2.023411	-0.917673
F	-7.677897	-2.627775	1.121224
F	-8.232749	-0.644261	0.456267
F	-6.352890	0.624040	-1.121949
C	4.820786	-2.997314	-2.525048
C	5.474145	-3.221717	-1.148409
C	4.061358	-1.320274	-0.946284
C	3.722179	-1.917026	-2.332071
H	4.415773	-3.921113	-2.941706
H	5.577016	-2.632468	-3.222562
N	5.284832	-1.886951	-0.536109
C	4.720504	-4.321654	-0.370583
H	5.198386	-4.524025	0.585851
H	3.690228	-4.022719	-0.176295
H	4.709566	-5.252410	-0.943168
C	2.315707	-2.536879	-2.357555
H	2.105451	-2.975868	-3.337696
H	2.213487	-3.317603	-1.603076
H	1.559829	-1.777143	-2.159291
C	3.823224	-0.855728	-3.440729
H	4.801533	-0.376000	-3.426692
H	3.678332	-1.310295	-4.426764
H	3.075172	-0.076779	-3.304741
C	6.945401	-3.617386	-1.251383
H	7.517533	-2.903435	-1.841126
H	7.403376	-3.685918	-0.263235
H	7.025973	-4.595081	-1.729988
C	6.201221	-1.276836	0.368868
C	6.109107	-1.472139	1.760601
C	7.226310	-0.461345	-0.165201
C	7.074628	-0.893631	2.587594
C	8.158053	0.104728	0.700505
C	8.095056	-0.117746	2.068000
H	7.017848	-1.046458	3.658334
H	8.944574	0.733594	0.303190
H	8.832745	0.325206	2.726222
C	4.982511	-2.261986	2.404100
H	4.301995	-2.568177	1.611930
C	7.297945	-0.142810	-1.649001
H	6.685441	-0.877519	-2.166102
C	4.178559	-1.397486	3.387563
H	4.787923	-1.090185	4.240171
H	3.800518	-0.500187	2.899268
H	3.325406	-1.960271	3.772698
C	5.503726	-3.520141	3.115162
H	4.671231	-4.133382	3.469087
H	6.122175	-4.130975	2.456288
H	6.114060	-3.253841	3.981187
C	6.673255	1.230792	-1.926450
H	5.643816	1.266016	-1.574486
H	7.235269	2.021001	-1.421221
H	6.675667	1.448137	-2.997911
C	8.715742	-0.231245	-2.225714
H	9.363113	0.559626	-1.840543
H	9.186749	-1.188266	-1.992110
H	8.684186	-0.122551	-3.312245
B	2.039823	0.020120	0.720586
B	3.237084	-0.387349	-0.258310
C	1.319898	1.230323	1.307151
C	0.300552	1.185075	2.451342
C	0.108202	2.674097	2.811738
C	0.563856	3.496773	1.602772
H	0.734752	2.919724	3.671218
H	-0.923458	2.894835	3.082454
N	1.443958	2.488776	0.888400
C	2.315053	2.878879	-0.195178
C	3.631755	3.275291	0.107749
C	1.842438	2.844495	-1.519598
C	4.445906	3.694503	-0.941590
C	2.709537	3.257841	-2.531675
C	3.992900	3.691626	-2.250083
H	5.462927	3.998239	-0.733324
H	2.369957	3.234716	-3.558889
H	4.648185	4.009009	-3.051651
C	-1.007642	0.554017	1.946496
H	-0.856846	-0.487100	1.674878
H	-1.770016	0.592609	2.725769
H	-1.406974	1.069339	1.077344

C	0.802437	0.430316	3.692635
H	0.111942	0.614168	4.519313
H	0.856016	-0.645280	3.545095
H	1.791519	0.780418	3.991729
C	-0.611618	3.943220	0.727530
H	-0.258050	4.478248	-0.151567
H	-1.241856	3.118651	0.408248
H	-1.236964	4.625064	1.304727
C	1.342053	4.742810	2.021563
H	2.139194	4.507723	2.721427
H	1.769178	5.254520	1.158762
H	0.654967	5.431509	2.515342
C	0.458847	2.350072	-1.912987
H	-0.106521	2.146913	-1.008112
C	4.227619	3.200555	1.505985
H	3.432920	2.933407	2.200878
C	5.284213	2.091940	1.588241
H	5.647711	1.984242	2.611698
H	4.879361	1.134776	1.267853
H	6.141524	2.314970	0.952598
C	4.833499	4.536984	1.961200
H	5.150762	4.468624	3.004230
H	5.715504	4.789801	1.369371
H	4.127853	5.362644	1.871025
C	0.533018	1.024815	-2.681244
H	1.084839	1.134967	-3.616811
H	1.027958	0.264843	-2.078725
H	-0.474544	0.684654	-2.922864
C	-0.316185	3.389044	-2.736431
H	0.123435	3.511566	-3.728720
H	-1.348109	3.068882	-2.868366
H	-0.322916	4.367660	-2.257018
N	1.585009	-1.255278	1.238158
N	1.289429	-2.290180	1.578247

[C_4mpyr]$^+$[PF_6]$^-$

37

N	1.478000	-1.505892	-0.129722
C	0.294193	-1.271992	0.804996
C	-0.743478	-2.350072	0.488311
C	-0.384723	-2.899504	-0.913498
C	0.848173	-2.121991	-1.375896
H	1.606486	-2.721225	-1.876894
H	-0.193806	-3.970753	-0.874026
H	-0.737505	-3.146495	1.231353
H	0.665279	-1.280034	1.828256
H	-0.092525	-0.286750	0.563806
H	-1.730687	-1.896233	0.485912
H	-1.193753	-2.723985	-1.617423
H	0.559350	-1.291240	-2.011893
C	2.140760	-0.199682	-0.471629
H	2.951078	-0.395837	-1.170819
H	1.411913	0.465849	-0.920282
H	2.535010	0.237906	0.443306
C	2.541132	-2.392451	0.487268
H	2.930359	-1.833948	1.339865
C	2.132941	-3.788429	0.928615
H	3.337494	-2.457454	-0.255649
H	1.752563	-4.362575	0.082063
H	1.333699	-3.738079	1.669774
C	3.331583	-4.526442	1.535819
C	2.971309	-5.938536	1.992501
H	4.141349	-4.573195	0.800227
H	3.719397	-3.953267	2.384275
H	3.836269	-6.444857	2.423375
H	2.184531	-5.918366	2.750559
H	2.612451	-6.543344	1.156333
P	-1.381055	1.262274	-1.921571
F	-1.086529	2.748575	-2.437691
F	-0.484407	1.554345	-0.561677
F	-1.603402	-0.281210	-1.366701
F	0.006971	0.731378	-2.649839
F	-2.704502	1.727102	-1.145362
F	-2.212795	0.901948	-3.243304

RC_{IL} (with [C_4mpyr]$^+$[PF_6]$^-$)

145

N	-0.232466	-2.202620	-0.601706
C	0.691235	-1.140666	-1.188056
C	0.762100	-1.470200	-2.673176
C	-0.602302	-2.122189	-3.007104
C	-1.313968	-2.321933	-1.663489
H	-2.022916	-1.524464	-1.473565
H	-1.208475	-1.469448	-3.630751

H	0.931775	-0.560680	-3.242732
H	0.201755	-0.186152	-1.025191
H	1.638237	-1.148615	-0.653970
H	1.588084	-2.146016	-2.891248
H	-0.468180	-3.065311	-3.536810
H	-1.807465	-3.284001	-1.549428
C	0.499061	-3.497089	-0.457405
H	-0.188153	-4.257121	-0.097020
H	0.899187	-3.798959	-1.420690
H	1.311840	-3.364890	0.251252
C	-0.765624	-1.746096	0.742073
H	-1.052878	-0.710371	0.607804
C	-1.938252	-2.544218	1.288287
H	0.082848	-1.784734	1.423762
H	-1.701682	-3.610633	1.338691
H	-2.800358	-2.424318	0.629653
C	-2.315921	-2.054354	2.696199
C	-2.768793	-0.593420	2.755058
H	-3.117733	-2.698490	3.067131
H	-1.465441	-2.209066	3.369782
H	-3.569324	-0.396649	2.040905
H	-3.131247	-0.348743	3.755106
H	-1.960640	0.100204	2.520434
P	-2.472126	1.538375	-1.059846
F	-1.132027	1.361519	-0.099633
F	-1.657616	0.716171	-2.233918
F	-3.755495	1.642348	-2.010136
C	6.483304	-2.585958	-2.113984
C	7.581255	-2.307016	-1.073457
C	5.702994	-0.901999	-0.568253
C	5.185743	-1.928675	-1.587064
H	6.360103	-3.653911	-2.299575
H	6.766386	-2.121061	-3.060195
N	7.054218	-1.063510	-0.439376
C	7.699789	-3.447356	-0.047824
H	8.475012	-3.227857	0.684017
H	6.764807	-3.607881	0.486898
H	7.968473	-4.376744	-0.554840
C	4.250321	-2.951320	-0.924119
H	3.875126	-3.663044	-1.666179
H	4.753585	-3.514395	-0.139252

H	3.407445	-2.435547	-0.466494
C	4.431839	-1.222641	-2.722987
H	5.054937	-0.457535	-3.187629
H	4.136925	-1.942757	-3.492163
H	3.538409	-0.727909	-2.345588
C	8.949549	-2.099625	-1.721755
H	8.916189	-1.338298	-2.497944
H	9.697394	-1.806979	-0.983960
H	9.274327	-3.035262	-2.180510
C	7.899143	-0.085913	0.179377
C	8.214017	-0.171133	1.546158
C	8.405389	0.961693	-0.618949
C	9.131300	0.739879	2.072395
C	9.312131	1.849084	-0.044389
C	9.694176	1.727379	1.282916
H	9.390068	0.686516	3.122360
H	9.714419	2.658825	-0.639313
H	10.404326	2.425321	1.709431
C	7.519414	-1.143207	2.484310
H	6.882687	-1.788957	1.883116
C	7.934679	1.198695	-2.045587
H	7.397379	0.309628	-2.373528
C	6.595555	-0.371688	3.440414
H	7.169842	0.284616	4.098960
H	5.891142	0.237872	2.875356
H	6.028195	-1.065056	4.065566
C	8.498900	-2.025087	3.270348
H	7.951692	-2.768374	3.854671
H	9.192378	-2.552152	2.612838
H	9.094794	-1.432464	3.967725
C	6.939063	2.368658	-2.083781
H	6.093679	2.189070	-1.420842
H	7.420130	3.297965	-1.773763
H	6.554576	2.512285	-3.096489
C	9.086924	1.439783	-3.030445
H	9.589940	2.389039	-2.835166
H	9.840026	0.651166	-2.979907
H	8.702483	1.481775	-4.051978
B	3.944547	1.072529	0.707879
B	4.862064	0.074262	0.096475
C	3.086327	2.016680	1.413834

C	2.279975	1.663053	2.668472
C	1.748935	3.037742	3.137666
C	1.820646	3.994240	1.933888
H	2.390346	3.417851	3.935484
H	0.736225	2.969726	3.535689
N	2.872490	3.316993	1.103515
C	3.652560	3.991528	0.103782
C	4.881318	4.568813	0.490075
C	3.209089	4.048597	-1.228909
C	5.606946	5.274722	-0.466269
C	3.979698	4.763274	-2.148443
C	5.155623	5.387640	-1.771837
H	6.550170	5.728141	-0.189142
H	3.652682	4.822285	-3.178503
H	5.734955	5.941226	-2.500928
C	1.147247	0.687727	2.319082
H	1.577281	-0.239844	1.940953
H	0.552924	0.460885	3.208923
H	0.481425	1.087677	1.558042
C	3.173928	1.027190	3.741018
H	2.601445	0.864751	4.658739
H	3.565611	0.068382	3.403304
H	4.025135	1.668461	3.974836
C	0.480965	4.085206	1.190673
H	0.556663	4.745927	0.328341
H	0.121631	3.119277	0.846248
H	-0.274603	4.501361	1.859932
C	2.241131	5.404457	2.348018
H	3.143568	5.397574	2.954194
H	2.412205	6.037901	1.476521
H	1.440877	5.853995	2.938027
C	1.971875	3.318108	-1.721702
H	1.451932	2.904934	-0.862164
C	5.476543	4.388595	1.878422
H	4.719479	3.930527	2.512953
C	6.668381	3.421095	1.826750
H	7.056283	3.236727	2.831387
H	6.379443	2.464682	1.393004
H	7.481024	3.833873	1.226573
C	5.893492	5.717284	2.525960
H	6.204143	5.549730	3.560050
H	6.738634	6.167736	2.001801
H	5.079775	6.443971	2.526538
C	2.369071	2.126208	-2.605566
H	2.895982	2.455382	-3.504871
H	3.019435	1.444344	-2.057091
H	1.469974	1.590773	-2.918765
C	0.997678	4.241295	-2.465384
H	1.425585	4.594073	-3.406602
H	0.073232	3.709791	-2.687098
H	0.742616	5.118976	-1.869077
N	2.969119	-2.714947	2.326538
N	3.894205	-2.758831	2.902077
F	-1.878750	2.924112	-1.615540
F	-3.009574	0.089379	-0.483104
F	-3.222076	2.289264	0.142553

Ts_{1IL} (with $[C_4mpyr]^+[PF_6]^-$)

145

N	0.356349	-5.428960	1.011087
C	-0.076924	-4.411607	2.057113
C	0.767824	-4.702462	3.299213
C	1.964188	-5.559301	2.812547
C	1.830479	-5.603000	1.292069
H	2.335961	-4.755285	0.840194
H	2.923738	-5.122748	3.085550
H	1.095161	-3.762004	3.737628
H	0.143693	-3.433304	1.646482
H	-1.150241	-4.491936	2.186550
H	0.190667	-5.231441	4.056471
H	1.928696	-6.561152	3.240017
H	2.183383	-6.520604	0.828174
C	-0.389070	-6.712830	1.231483
H	0.037029	-7.485377	0.597752
H	-0.290752	-7.000364	2.274973
H	-1.435182	-6.551858	0.989212
C	0.084648	-4.865106	-0.371029
H	0.693425	-3.964708	-0.442187
C	0.373392	-5.797789	-1.533980
H	-0.957485	-4.572312	-0.364034
H	-0.284235	-6.668197	-1.489980
H	1.406351	-6.156258	-1.496263

C	0.135396	-5.073434	-2.869433
C	-1.313992	-4.636275	-3.095099
H	0.798885	-4.205601	-2.929101
H	0.442638	-5.748684	-3.672697
H	-1.630637	-3.864723	-2.392947
H	-1.435446	-4.233506	-4.102269
H	-2.004917	-5.472575	-2.976032
P	-3.897618	-4.590941	0.440496
F	-5.067825	-5.659014	0.661186
F	-4.695248	-3.457186	1.256294
F	-2.643649	-3.527898	0.233175
F	-2.999357	-5.719833	-0.357485
F	-4.525233	-4.101903	-0.953172
F	-3.174436	-5.084713	1.839475
C	6.374468	-0.459319	-3.499565
C	7.115910	-0.613904	-2.151503
C	4.817853	-0.139300	-1.684484
C	4.976636	0.140798	-3.185667
H	6.258602	-1.444673	-3.956243
H	6.933776	0.158075	-4.203831
N	5.969315	-0.781287	-1.227512
C	8.059174	-1.814333	-2.128265
H	7.555703	-2.734353	-2.418817
H	8.882402	-1.644969	-2.824316
H	8.483890	-1.957472	-1.132911
C	3.876262	-0.558162	-4.000748
H	4.028745	-0.397517	-5.072294
H	3.868742	-1.632357	-3.815209
H	2.893054	-0.166051	-3.733383
C	4.921010	1.647920	-3.489184
H	5.669323	2.202781	-2.924777
H	5.089318	1.830092	-4.554577
H	3.943012	2.051150	-3.223823
C	7.926772	0.655354	-1.825373
H	8.488342	0.535301	-0.900153
H	8.639886	0.862903	-2.626354
H	7.274081	1.520598	-1.716443
C	5.893378	-1.641140	-0.090472
C	5.401819	-2.956231	-0.263235
C	6.220799	-1.168177	1.194148
C	5.267337	-3.773172	0.857282

C	6.077360	-2.028076	2.285078
C	5.604782	-3.319246	2.124200
H	4.899850	-4.785362	0.737400
H	6.328763	-1.674387	3.277096
H	5.499844	-3.970985	2.983710
C	5.000572	-3.494658	-1.627146
H	5.347693	-2.783130	-2.370915
C	6.709468	0.250044	1.426788
H	6.620392	0.779064	0.480068
C	3.476617	-3.581683	-1.776538
H	3.011019	-2.616609	-1.576762
H	3.209637	-3.888274	-2.790095
H	3.064485	-4.322913	-1.091334
C	5.649654	-4.849474	-1.942922
H	5.435494	-5.138184	-2.974428
H	6.732746	-4.816070	-1.814817
H	5.266092	-5.642032	-1.296021
C	5.841578	1.001726	2.444844
H	6.168340	2.041661	2.517711
H	4.796370	0.990511	2.137895
H	5.914828	0.561808	3.441792
C	8.183619	0.262402	1.857769
H	8.305548	-0.207462	2.836847
H	8.814434	-0.281798	1.152785
H	8.556011	1.287238	1.928893
B	2.622629	0.580485	0.173479
B	3.725751	0.217183	-0.832628
C	1.854347	1.683263	0.777958
C	2.216883	3.137758	0.417261
C	1.413768	3.952784	1.452093
C	0.245285	3.074355	1.915284
H	1.068403	4.904213	1.047176
H	2.054644	4.174254	2.307741
N	0.779244	1.701941	1.602516
C	0.137739	0.528911	2.130214
C	-0.900656	-0.096158	1.406033
C	0.553560	0.028079	3.382255
C	-1.593490	-1.143154	2.016041
C	-0.165689	-1.028575	3.940053
C	-1.248722	-1.588593	3.281600
H	-2.398076	-1.634826	1.489181

H	0.125398	-1.414294	4.908963
H	-1.814214	-2.392274	3.737536
C	3.713833	3.447886	0.537823
H	4.294695	2.852577	-0.166136
H	3.886459	4.507333	0.328991
H	4.077328	3.233951	1.543119
C	1.762817	3.442394	-1.025247
H	1.891602	4.506772	-1.239331
H	2.359594	2.866009	-1.729876
H	0.717156	3.184968	-1.190811
C	-0.036951	3.261508	3.406730
H	0.864219	3.144176	4.002785
H	-0.416498	4.271587	3.569647
H	-0.789866	2.558025	3.763592
C	-1.054293	3.363668	1.152514
H	-1.382407	4.380751	1.373517
H	-0.933491	3.275482	0.075933
H	-1.842764	2.682321	1.468193
C	1.780022	0.543347	4.125889
H	2.146314	1.426136	3.603750
C	-1.255128	0.260991	-0.031748
H	-0.699494	1.154327	-0.308026
C	-0.820225	-0.857291	-0.998571
H	-1.094196	-0.588420	-2.021333
H	0.258533	-1.006057	-0.975642
H	-1.320004	-1.795812	-0.755668
C	-2.755123	0.538751	-0.217419
H	-2.942366	0.916109	-1.225089
H	-3.343582	-0.371348	-0.094760
H	-3.127164	1.275665	0.494783
C	2.916907	-0.493049	4.105928
H	3.262249	-0.699793	3.096325
H	3.771340	-0.123123	4.676569
H	2.594629	-1.434218	4.558832
C	1.467781	0.934822	5.579313
H	2.330584	1.432010	6.028432
H	0.611655	1.604967	5.648853
H	1.247237	0.054958	6.187336
N	2.035236	-1.135439	0.816310
N	2.196066	-2.198286	1.073008

Int_{1IL} (with $[C_4mpyr]^+[PF_6]^-$)

145

N	-1.233831	-4.501038	-0.883879
C	-1.765932	-3.581226	0.208019
C	-1.311213	-4.206628	1.527671
C	-0.133528	-5.148891	1.172104
C	0.095272	-4.955309	-0.325466
H	0.798345	-4.147806	-0.502913
H	0.775819	-4.895607	1.714360
H	-1.005151	-3.417070	2.209280
H	-1.308650	-2.611324	0.045981
H	-2.837198	-3.474048	0.082705
H	-2.120789	-4.755035	2.008135
H	-0.374615	-6.186205	1.404628
H	0.428219	-5.844661	-0.854827
C	-2.171448	-5.655936	-1.055092
H	-1.725074	-6.386268	-1.724023
H	-2.350109	-6.113101	-0.085524
H	-3.104992	-5.278640	-1.463248
C	-1.091947	-3.722322	-2.176640
H	-0.407810	-2.905547	-1.949877
C	-0.600492	-4.515069	-3.375990
H	-2.069608	-3.295915	-2.364267
H	-1.331648	-5.277361	-3.656207
H	0.337691	-5.027750	-3.147023
C	-0.383606	-3.578641	-4.575891
C	-1.661004	-2.899297	-5.075445
H	0.359327	-2.822133	-4.305877
H	0.059381	-4.166071	-5.384849
H	-2.090609	-2.218931	-4.339236
H	-1.455763	-2.318960	-5.976792
H	-2.428584	-3.636988	-5.320964
P	-4.745384	-1.881609	-1.757439
F	-6.286453	-2.299008	-1.649687
F	-5.087573	-0.323611	-1.528460
F	-3.140117	-1.498459	-1.844307
F	-4.317381	-3.469249	-1.950970
F	-4.851976	-1.722161	-3.353993
F	-4.565284	-2.080729	-0.139357
C	6.944087	-1.004748	-2.569338
C	7.159686	-1.672445	-1.190548
C	5.049228	-0.539177	-1.153277

C	5.744755	-0.028330	-2.425312
H	6.702963	-1.777735	-3.301935
H	7.842739	-0.496099	-2.922175
N	5.787400	-1.629750	-0.645370
C	7.687899	-3.100998	-1.309545
H	7.058258	-3.713327	-1.953029
H	8.693551	-3.085701	-1.733150
H	7.741412	-3.580742	-0.330531
C	4.817727	-0.103224	-3.649441
H	5.343307	0.213234	-4.555690
H	4.449282	-1.116826	-3.804989
H	3.951550	0.548841	-3.516135
C	6.222397	1.426524	-2.273081
H	6.866663	1.552550	-1.404071
H	6.777645	1.743294	-3.161135
H	5.370793	2.096814	-2.150602
C	8.144916	-0.851614	-0.334302
H	8.350986	-1.350007	0.612049
H	9.094247	-0.725579	-0.860293
H	7.741864	0.136526	-0.114956
C	5.126876	-2.646541	0.099875
C	4.359972	-3.623165	-0.580832
C	5.177119	-2.646746	1.507690
C	3.688823	-4.589912	0.164669
C	4.497177	-3.643344	2.211475
C	3.759722	-4.610671	1.550864
H	3.114961	-5.352930	-0.347525
H	4.539729	-3.654632	3.293502
H	3.246489	-5.382274	2.112720
C	4.241587	-3.639320	-2.095883
H	4.981745	-2.944598	-2.483223
C	5.931668	-1.578990	2.278643
H	6.279212	-0.848014	1.551192
C	2.868451	-3.134316	-2.558289
H	2.672885	-2.135617	-2.169486
H	2.821154	-3.093421	-3.648741
H	2.078459	-3.805448	-2.220077
C	4.539312	-5.018259	-2.700960
H	4.565539	-4.952929	-3.791220
H	5.499001	-5.408486	-2.358597
H	3.772591	-5.750840	-2.437414

C	5.022445	-0.837431	3.268070
H	5.567379	-0.013003	3.733926
H	4.150347	-0.429050	2.757575
H	4.673956	-1.496107	4.066537
C	7.151432	-2.167607	3.002357
H	6.841748	-2.867503	3.782298
H	7.801954	-2.710625	2.314747
H	7.738885	-1.376924	3.475666
B	2.540948	0.359147	0.235784
B	3.898314	-0.019689	-0.508774
C	2.134234	1.604026	0.996661
C	3.049869	2.822535	1.120955
C	2.323048	3.702805	2.160772
C	0.855017	3.257858	2.187054
H	2.416991	4.765229	1.935252
H	2.767493	3.539132	3.144341
N	0.959488	1.882370	1.571596
C	-0.163221	0.975865	1.593153
C	-1.098195	0.992179	0.537913
C	-0.294772	0.085656	2.677474
C	-2.227969	0.181560	0.653129
C	-1.447165	-0.696773	2.741798
C	-2.418534	-0.634021	1.755622
H	-2.963499	0.166867	-0.137042
H	-1.582152	-1.371765	3.577928
H	-3.312876	-1.240632	1.812459
C	4.463058	2.481986	1.610897
H	4.988542	1.851045	0.895484
H	5.028834	3.407277	1.749682
H	4.429409	1.956902	2.565647
C	3.143393	3.520404	-0.251297
H	3.722553	4.442483	-0.158234
H	3.636899	2.868872	-0.969952
H	2.160223	3.773106	-0.648513
C	0.308004	3.199766	3.612966
H	0.948818	2.607968	4.262668
H	0.267123	4.212995	4.016195
H	-0.699938	2.784944	3.637609
C	-0.052741	4.175100	1.361117
H	-0.079658	5.160497	1.828780
H	0.301189	4.297461	0.340114

H	-1.069994	3.789329	1.332558
C	0.786512	-0.127250	3.729722
H	1.571847	0.611189	3.570580
C	-0.910767	1.780576	-0.752176
H	-0.041565	2.424683	-0.638591
C	-0.623978	0.841197	-1.940039
H	-0.511339	1.430778	-2.853083
H	0.297296	0.280038	-1.793584
H	-1.445047	0.140224	-2.090833
C	-2.129534	2.657126	-1.082935
H	-1.908321	3.295238	-1.941123
H	-2.994463	2.045131	-1.343250
H	-2.413933	3.297847	-0.248307
C	1.436332	-1.513380	3.569038
H	2.210914	-1.655579	4.324575
H	0.695578	-2.305951	3.698890
H	1.899786	-1.637669	2.592736
C	0.260425	0.032621	5.164693
H	1.088702	-0.007773	5.876037
H	-0.265516	0.976182	5.305703
H	-0.431054	-0.771451	5.423296
N	1.616497	-0.750304	0.120737
N	1.021438	-1.694787	0.003720

System (2)

132

B	5.544146	11.990010	3.869584
B	4.100213	12.122266	3.953928
C	7.013053	11.852159	3.768097
N	7.991339	12.718938	4.227787
C	9.256675	12.230157	3.935758
H	10.145319	12.773492	4.199140
C	9.097792	11.057065	3.292415
H	9.820767	10.357871	2.914576
N	7.734437	10.823288	3.185084
C	2.629186	12.262755	4.043747
N	1.842133	13.321895	3.623419
C	0.503748	13.075456	3.891161
H	-0.260538	13.789118	3.642822
C	0.426631	11.865103	4.480832
H	-0.419899	11.306151	4.836057
N	1.716811	11.366684	4.573541
C	7.751644	14.031606	4.743981
C	7.707774	14.214685	6.132917
C	7.575422	15.518104	6.607770
H	7.545986	15.701030	7.673499
C	7.454189	16.586520	5.730258
H	7.341037	17.591555	6.118805
C	7.454261	16.371897	4.360574
H	7.330386	17.210732	3.687634
C	7.611762	15.089445	3.835784
C	7.812939	13.031518	7.081086
H	7.496635	12.145888	6.526463
C	6.880773	13.159045	8.291013
H	7.205835	13.946377	8.975025
H	6.869354	12.222430	8.851998
H	5.861503	13.378163	7.976417
C	9.264826	12.812861	7.535470
H	9.928667	12.640998	6.686980
H	9.333549	11.945932	8.196952
H	9.632579	13.686292	8.079978
C	7.607464	14.865742	2.332870
H	7.849067	13.819760	2.145272
C	8.673952	15.709816	1.621767
H	8.473210	16.778934	1.720687
H	8.689132	15.474379	0.555176
H	9.669553	15.516848	2.027524
C	6.208735	15.118093	1.757092
H	5.472360	14.483441	2.251329
H	6.191706	14.898345	0.686539
H	5.910101	16.158869	1.891817
C	7.178281	9.600326	2.692889
C	6.866286	9.500359	1.329509
C	6.431818	8.263637	0.857991
H	6.186497	8.143364	-0.188446
C	6.282629	7.184833	1.719101
H	5.936685	6.233357	1.333561
C	6.547354	7.326368	3.072275
H	6.394176	6.486827	3.738278
C	7.005354	8.538375	3.589509
C	7.005065	10.701420	0.407483
H	6.885851	11.593795	1.024870
C	5.913556	10.754616	-0.666484

H	6.039470	9.968384	-1.414671
H	5.954057	11.711663	-1.189980
H	4.923553	10.653050	-0.223884
C	8.399217	10.751155	-0.237687
H	9.187661	10.802028	0.514100
H	8.492265	11.629684	-0.880744
H	8.570950	9.862085	-0.849871
C	7.264175	8.693586	5.078484
H	7.713512	9.672945	5.243598
C	8.252857	7.646880	5.608755
H	7.854235	6.634027	5.518932
H	8.458967	7.826713	6.666536
H	9.200072	7.683883	5.066100
C	5.939644	8.667175	5.853279
H	5.264146	9.438646	5.481737
H	6.116735	8.845521	6.917077
H	5.443412	7.700463	5.751354
C	2.343449	14.576420	3.151003
C	2.373809	14.818052	1.769854
C	2.822941	16.066869	1.344173
H	2.864473	16.290441	0.286739
C	3.234149	17.026605	2.258538
H	3.586669	17.988596	1.906358
C	3.207010	16.756134	3.617483
H	3.541841	17.508146	4.320429
C	2.754223	15.526659	4.095117
C	1.986516	13.736042	0.774799
H	1.327015	13.033726	1.287752
C	3.230942	12.947737	0.336427
H	3.926289	13.597035	-0.202052
H	2.945623	12.130557	-0.330394
H	3.758366	12.528522	1.195327
C	1.217528	14.273685	-0.437584
H	0.351606	14.865157	-0.132795
H	0.864856	13.442836	-1.052358
H	1.846878	14.900393	-1.072875
C	2.716873	15.252688	5.588835
H	2.296790	14.258786	5.741675
C	1.807604	16.248564	6.323070
H	2.188741	17.269357	6.248084
H	1.749385	15.993517	7.383822

H	0.794938	16.238147	5.913851
C	4.132187	15.237745	6.177031
H	4.744471	14.490267	5.671625
H	4.096695	14.996069	7.241971
H	4.619067	16.208476	6.070945
C	2.038547	10.053556	5.041491
C	2.260083	9.857972	6.411454
C	2.530410	8.560037	6.842151
H	2.714346	8.371781	7.891755
C	2.585170	7.506810	5.940993
H	2.802867	6.506358	6.294978
C	2.379050	7.731307	4.588397
H	2.442523	6.904033	3.893113
C	2.097244	9.009677	4.108564
C	2.263497	11.021506	7.388080
H	1.783202	11.869796	6.898511
C	3.705839	11.442495	7.708029
H	4.243856	10.631914	8.206580
H	3.707546	12.309568	8.372618
H	4.247754	11.702121	6.796904
C	1.469575	10.728695	8.666762
H	0.448966	10.413067	8.439708
H	1.419189	11.625173	9.288634
H	1.937910	9.944230	9.265197
C	1.883635	9.243935	2.622946
H	1.599613	10.286751	2.482164
C	0.741013	8.382637	2.067191
H	0.973386	7.317281	2.130125
H	0.566975	8.621394	1.015340
H	-0.188517	8.555016	2.614394
C	3.188998	9.023083	1.849180
H	3.975525	9.671978	2.236198
H	3.044507	9.245733	0.788998
H	3.528455	7.989234	1.931399

RC_2

134

B	5.499044	12.537983	2.927409
B	4.053973	12.678591	2.961241
C	6.965916	12.363268	2.905331
N	7.936119	13.188090	3.456898
C	9.200902	12.646254	3.266394

H	10.085954	13.139858	3.623300
C	9.049780	11.497848	2.579103
H	9.773815	10.779321	2.241021
N	7.693284	11.329653	2.337886
C	2.582863	12.833342	3.002977
N	1.818204	13.901166	2.564532
C	0.471473	13.671118	2.803757
H	-0.278772	14.393483	2.538261
C	0.367552	12.461201	3.390552
H	-0.492506	11.912716	3.729292
N	1.649415	11.946362	3.507314
C	7.718318	14.542081	3.870721
C	7.675591	14.838865	5.238960
C	7.558181	16.177572	5.610621
H	7.531705	16.441039	6.659607
C	7.451613	17.175221	4.653919
H	7.346047	18.208681	4.961257
C	7.464182	16.852882	3.304970
H	7.365988	17.638693	2.566867
C	7.610693	15.532962	2.881856
C	7.771148	13.745195	6.287787
H	7.568923	12.798840	5.784717
C	6.722195	13.906401	7.393687
H	6.919246	14.782085	8.015709
H	6.727102	13.032609	8.048168
H	5.725015	14.006098	6.968698
C	9.186464	13.673512	6.880896
H	9.932995	13.480052	6.109029
H	9.255449	12.875146	7.623162
H	9.445774	14.615002	7.371316
C	7.653359	15.208475	1.397884
H	7.867323	14.146477	1.290494
C	8.780554	15.962558	0.678737
H	8.615977	17.042217	0.691391
H	8.832670	15.647592	-0.366074
H	9.749492	15.764667	1.142271
C	6.291907	15.468377	0.741097
H	5.510890	14.894818	1.241351
H	6.315835	15.174347	-0.311497
H	6.025617	16.525758	0.787670
C	7.146720	10.208619	1.633951
C	6.886853	10.332922	0.258314
C	6.457975	9.193940	-0.418787
H	6.249034	9.245148	-1.478001
C	6.273679	7.990229	0.249095
H	5.936215	7.118106	-0.297824
C	6.487044	7.909610	1.614308
H	6.298182	6.977413	2.130966
C	6.924469	9.019948	2.338188
C	7.055127	11.667838	-0.453840
H	6.736932	12.439428	0.250459
C	6.164796	11.806849	-1.692042
H	6.508491	11.172394	-2.512768
H	6.188723	12.838683	-2.047084
H	5.129644	11.551166	-1.469515
C	8.520121	11.937106	-0.834200
H	9.176478	11.930668	0.034686
H	8.612469	12.915133	-1.313109
H	8.877863	11.181007	-1.537938
C	7.071176	8.941725	3.847488
H	7.552233	9.858254	4.189005
C	7.950947	7.768876	4.297287
H	7.504840	6.806364	4.038055
H	8.077958	7.788911	5.381549
H	8.941047	7.814278	3.838752
C	5.679499	8.885664	4.492832
H	5.086173	9.755099	4.205939
H	5.755532	8.864372	5.581419
H	5.140839	7.989569	4.179482
C	2.348567	15.131728	2.063112
C	2.390928	15.332985	0.675510
C	2.886708	16.551580	0.215946
H	2.939334	16.742976	-0.847378
C	3.329561	17.521535	1.104653
H	3.719668	18.458893	0.726684
C	3.282384	17.293830	2.470851
H	3.637123	18.055267	3.153645
C	2.783981	16.095618	2.981554
C	1.970300	14.235954	-0.289055
H	1.280470	13.574435	0.237825
C	3.189975	13.392327	-0.689012
H	3.908919	13.998879	-1.244752

H	2.883944	12.559552	-1.327343
H	3.698587	12.988159	0.188253
C	1.232622	14.762356	-1.525368
H	0.384700	15.392111	-1.248116
H	0.857124	13.925364	-2.117964
H	1.889083	15.347143	-2.173152
C	2.720110	15.870410	4.482145
H	2.279328	14.889463	4.658996
C	1.817680	16.906788	5.167309
H	2.219203	17.917187	5.062366
H	1.739395	16.690278	6.235342
H	0.811282	16.900294	4.742832
C	4.123992	15.850293	5.096018
H	4.732748	15.078734	4.623753
H	4.065067	15.640275	6.166556
H	4.627894	16.810017	4.972281
C	1.941266	10.630740	3.987507
C	2.183530	10.448080	5.354449
C	2.388091	9.144682	5.806207
H	2.582966	8.968815	6.856416
C	2.360784	8.073570	4.926512
H	2.524197	7.068275	5.295509
C	2.154731	8.286754	3.571448
H	2.169647	7.445408	2.890588
C	1.945028	9.570072	3.070153
C	2.272511	11.620346	6.315266
H	1.971058	12.519275	5.776480
C	3.725339	11.832663	6.766039
H	4.103019	10.958785	7.302697
H	3.789671	12.692591	7.435900
H	4.370351	12.015909	5.904808
C	1.325847	11.466347	7.512857
H	0.293154	11.326122	7.186153
H	1.366112	12.358971	8.141234
H	1.598738	10.611943	8.136111
C	1.781712	9.797096	1.576593
H	1.501358	10.839262	1.421172
C	0.668125	8.931782	0.972275
H	0.903221	7.867300	1.038492
H	0.536922	9.173403	-0.085028
H	-0.284434	9.096811	1.480782
C	3.121560	9.576308	0.859684
H	3.890294	10.231979	1.271486
H	3.021963	9.787643	-0.207894
H	3.459617	8.543872	0.967918
N	8.059874	10.310587	6.911342
N	7.188011	10.248630	7.564726

TS1_2

134

B	5.439366	12.293191	3.264946
B	4.068139	12.151298	3.867585
C	6.846297	11.886940	3.357204
N	7.853243	12.518342	4.091149
C	9.033474	11.784487	4.031446
H	9.923763	12.112868	4.535804
C	8.807719	10.710069	3.257399
H	9.461791	9.911776	2.959375
N	7.488965	10.771954	2.812635
C	2.595054	12.462847	3.873294
N	2.009605	13.547029	3.257283
C	0.634574	13.531113	3.432482
H	0.009763	14.302472	3.021375
C	0.335411	12.443119	4.164753
H	-0.608105	12.054960	4.500100
N	1.529385	11.783754	4.438758
C	7.791578	13.850824	4.601773
C	7.569485	14.050141	5.971333
C	7.630493	15.356451	6.457457
H	7.476450	15.540005	7.513388
C	7.862943	16.424588	5.605965
H	7.895421	17.432983	6.000586
C	8.038619	16.207038	4.246587
H	8.204221	17.050511	3.588242
C	8.019848	14.918743	3.716807
C	7.266326	12.894451	6.907551
H	7.132206	12.002590	6.296736
C	5.956562	13.116650	7.675988
H	6.025981	13.971312	8.352858
H	5.718621	12.235077	8.274009
H	5.130171	13.293508	6.986865
C	8.436088	12.629777	7.866703
H	9.357028	12.421673	7.317915

H	8.219625	11.770514	8.505488
H	8.619279	13.492944	8.511685
C	8.238893	14.698396	2.228824
H	8.320528	13.626571	2.057245
C	9.547848	15.329752	1.735814
H	9.528712	16.418894	1.817096
H	9.710280	15.080463	0.684581
H	10.403472	14.964687	2.307676
C	7.033030	15.196360	1.421428
H	6.120923	14.695568	1.746157
H	7.176383	14.992372	0.356788
H	6.895555	16.273459	1.542282
C	6.910941	9.807038	1.931339
C	6.828220	10.101250	0.555235
C	6.333891	9.107091	-0.285851
H	6.253421	9.292199	-1.347484
C	5.947828	7.868488	0.211777
H	5.574280	7.109397	-0.464927
C	6.030008	7.606683	1.567591
H	5.714611	6.642691	1.944943
C	6.500269	8.573300	2.458046
C	7.282632	11.448539	0.006206
H	6.900057	12.211138	0.690648
C	6.730236	11.762776	-1.388242
H	7.213579	11.154287	-2.157250
H	6.927378	12.808825	-1.630414
H	5.656491	11.599921	-1.452534
C	8.817068	11.565696	-0.036151
H	9.271528	11.459712	0.946188
H	9.106553	12.543377	-0.429455
H	9.237906	10.800215	-0.693296
C	6.574829	8.267091	3.944430
H	6.732056	9.208352	4.470686
C	7.767678	7.349736	4.257719
H	7.658684	6.386755	3.752388
H	7.834094	7.163537	5.332235
H	8.710024	7.792964	3.932730
C	5.272215	7.662315	4.482448
H	4.414880	8.286377	4.239641
H	5.322814	7.574689	5.568948
H	5.090265	6.663940	4.077545
C	2.686259	14.584326	2.536566
C	2.797944	14.465797	1.146503
C	3.392249	15.520011	0.454915
H	3.502869	15.457754	-0.620341
C	3.852956	16.642553	1.126258
H	4.316550	17.449538	0.572175
C	3.736872	16.728724	2.505412
H	4.112967	17.603844	3.019859
C	3.150636	15.700683	3.241699
C	2.314898	13.232017	0.404944
H	1.847318	12.564780	1.127858
C	3.499055	12.475661	-0.206027
H	3.997923	13.085165	-0.962302
H	3.160063	11.553480	-0.684475
H	4.226029	12.220470	0.566468
C	1.254454	13.574480	-0.650578
H	0.402967	14.092384	-0.203812
H	0.887359	12.662566	-1.127199
H	1.662642	14.217267	-1.433700
C	3.037232	15.803961	4.751483
H	2.580867	14.884767	5.118969
C	2.130476	16.969533	5.171501
H	2.541495	17.928118	4.846826
H	2.031235	17.000177	6.259132
H	1.131456	16.871278	4.740912
C	4.423963	15.905168	5.393885
H	5.050611	15.071001	5.081444
H	4.344322	15.886662	6.482886
H	4.931096	16.830262	5.113588
C	1.553418	10.440159	4.941096
C	1.546378	10.232361	6.328531
C	1.533853	8.916178	6.783177
H	1.533472	8.716065	7.846222
C	1.546669	7.852861	5.890037
H	1.552493	6.836078	6.263157
C	1.559240	8.086910	4.523942
H	1.575776	7.248956	3.838688
C	1.552711	9.385784	4.015677
C	1.559979	11.403284	7.298195
H	1.961430	12.263911	6.759270
C	2.462104	11.168241	8.516070

H	2.053243	10.405217	9.182372
H	2.549673	12.090619	9.093989
H	3.462492	10.857457	8.217096
C	0.133380	11.756963	7.750106
H	-0.508489	12.003150	6.903346
H	0.146406	12.617251	8.423464
H	-0.319361	10.916028	8.281134
C	1.533799	9.615928	2.511851
H	1.473802	10.688436	2.333736
C	0.291088	8.984472	1.867102
H	0.301325	7.896000	1.958442
H	0.256242	9.227626	0.802759
H	-0.626923	9.351279	2.331343
C	2.823670	9.123721	1.843815
H	3.692819	9.643402	2.246823
H	2.790556	9.314278	0.769090
H	2.964559	8.050764	1.987989
N	4.302195	11.008380	5.361196
N	4.963590	10.449799	6.057792

Int1_2

134

B	5.593732	12.370225	3.736464
B	4.311577	11.849290	4.502000
C	7.043818	12.159092	3.694208
N	8.092679	12.899051	4.273469
C	9.326904	12.366870	3.886981
H	10.249347	12.796567	4.232956
C	9.096698	11.310117	3.093623
H	9.780734	10.637912	2.608983
N	7.718202	11.150949	2.977876
C	2.800516	12.112532	4.416424
N	2.181269	13.104950	3.703492
C	0.809791	13.075492	3.908226
H	0.158496	13.780795	3.426191
C	0.551557	12.058218	4.749753
H	-0.374142	11.679986	5.142039
N	1.768592	11.465294	5.061844
C	7.976377	14.169951	4.910538
C	7.624978	14.230469	6.267215
C	7.631706	15.477229	6.892709
H	7.383540	15.546997	7.943913
C	7.955837	16.627309	6.191499
H	7.955809	17.586921	6.694469
C	8.263748	16.553382	4.841056
H	8.487956	17.462518	4.298723
C	8.278079	15.330349	4.171767
C	7.288911	12.980290	7.058009
H	7.139751	12.168460	6.348864
C	5.986549	13.130478	7.850667
H	6.058133	13.909069	8.613638
H	5.751640	12.193764	8.354506
H	5.157891	13.375745	7.184046
C	8.456312	12.586114	7.975430
H	9.371703	12.430871	7.400710
H	8.226922	11.659462	8.506926
H	8.652387	13.364879	8.716804
C	8.539479	15.278380	2.674488
H	8.833222	14.261597	2.416514
C	9.681442	16.196310	2.223515
H	9.429059	17.252843	2.337252
H	9.896654	16.027552	1.166091
H	10.594467	16.005577	2.791503
C	7.241310	15.583576	1.913676
H	6.461714	14.865829	2.171734
H	7.407733	15.537167	0.834932
H	6.877997	16.584658	2.158324
C	7.076510	10.282754	2.047541
C	6.938802	10.706268	0.715786
C	6.330200	9.832789	-0.185511
H	6.218019	10.126236	-1.221654
C	5.864222	8.594008	0.227999
H	5.386832	7.931830	-0.484355
C	6.010348	8.195692	1.549803
H	5.645674	7.223784	1.853457
C	6.618379	9.029537	2.487169
C	7.495843	12.040226	0.242304
H	7.781208	12.612670	1.123015
C	6.465288	12.885627	-0.512773
H	6.088817	12.375808	-1.402424
H	6.919114	13.824140	-0.838358
H	5.622740	13.127582	0.132099
C	8.762194	11.821834	-0.600285

H	9.514401	11.259177	-0.044205
H	9.199829	12.780840	-0.888369
H	8.535155	11.265731	-1.513586
C	6.858495	8.575496	3.917493
H	6.919281	9.471015	4.534378
C	8.212481	7.851562	4.019149
H	8.213000	6.947738	3.404257
H	8.413477	7.560676	5.053138
H	9.030840	8.489610	3.683698
C	5.742310	7.699622	4.492363
H	4.773225	8.190385	4.429534
H	5.939358	7.499637	5.546663
H	5.675106	6.736170	3.980726
C	2.785297	14.003682	2.758586
C	2.730352	13.665951	1.402296
C	3.236709	14.592789	0.490858
H	3.220549	14.362826	-0.566893
C	3.766861	15.798093	0.921408
H	4.162218	16.502130	0.199676
C	3.807049	16.103089	2.275469
H	4.237262	17.042100	2.594934
C	3.323132	15.208893	3.225596
C	2.149679	12.348558	0.913718
H	1.829985	11.776360	1.783397
C	3.201985	11.499837	0.191620
H	3.561841	11.993017	-0.712822
H	2.777091	10.537637	-0.101357
H	4.058351	11.310264	0.837009
C	0.909389	12.572112	0.037188
H	0.146071	13.146347	0.566738
H	0.473285	11.614235	-0.256320
H	1.162452	13.116668	-0.875391
C	3.345521	15.534855	4.708772
H	3.329391	14.588081	5.249536
C	2.093179	16.331296	5.108093
H	2.061198	17.287703	4.580688
H	2.095642	16.535329	6.181618
H	1.178108	15.785107	4.870918
C	4.621983	16.259313	5.137200
H	5.506499	15.709813	4.820694
H	4.654746	16.348104	6.224134

H	4.680115	17.269569	4.726486
C	1.853034	10.175524	5.692320
C	2.077560	10.100386	7.074399
C	2.144523	8.831929	7.644287
H	2.326748	8.728435	8.704523
C	2.012365	7.689758	6.865739
H	2.086565	6.712726	7.327386
C	1.795368	7.793349	5.501746
H	1.703205	6.894629	4.905626
C	1.697872	9.040179	4.883667
C	2.254317	11.359124	7.909031
H	2.754741	12.097218	7.277489
C	3.134357	11.146701	9.144659
H	2.625051	10.549231	9.904542
H	3.374724	12.110620	9.596195
H	4.068341	10.648958	8.884785
C	0.893524	11.944178	8.323616
H	0.278308	12.194528	7.459471
H	1.034180	12.855770	8.909085
H	0.341682	11.228004	8.937342
C	1.441005	9.129451	3.386824
H	1.243455	10.170834	3.137110
C	0.194440	8.334331	2.973111
H	0.333694	7.260686	3.115012
H	-0.020193	8.501202	1.915331
H	-0.680808	8.637942	3.551118
C	2.671734	8.698098	2.579875
H	3.527116	9.338760	2.792264
H	2.467538	8.759410	1.509496
H	2.951267	7.667929	2.809723
N	4.716570	10.938135	5.559587
N	5.168705	10.280400	6.357393

TS2_2

C	1.968272	4.183461	-0.248661
C	1.930569	5.312511	-1.077041
C	1.165239	5.372452	-2.250169
C	0.394475	4.255372	-2.569444
C	0.409157	3.125731	-1.764897
C	1.195637	3.086954	-0.622333
N	2.717236	6.454814	-0.718494
C	2.328231	7.506378	0.094988

N	3.419362	8.355697	0.062266
C	4.419264	7.841137	-0.756297
C	3.986661	6.663536	-1.233646
B	1.040883	7.634968	0.913770
N	0.169193	6.488832	0.848862
N	-0.580199	5.645622	0.924000
C	3.513270	9.715638	0.518979
C	4.254896	10.001973	1.675319
C	4.427575	11.343619	2.011948
C	3.904256	12.355110	1.220041
C	3.208776	12.043984	0.062957
C	3.001667	10.718947	-0.316281
C	4.938582	8.911414	2.483952
C	6.426201	8.818943	2.100563
C	2.268575	10.404162	-1.609797
C	0.797913	10.819071	-1.524824
C	1.144583	6.610695	-3.134235
C	1.287575	6.270549	-4.623612
C	2.817689	4.155589	1.010749
C	2.096954	3.517147	2.202453
B	0.643335	8.505677	2.230657
C	-0.520217	8.847136	3.089605
N	-1.719288	9.448997	2.645696
C	-2.551277	9.738189	3.726495
C	-1.951971	9.303542	4.840608
N	-0.729027	8.738285	4.480413
C	-1.984098	10.003581	1.354343
C	-2.636962	9.216328	0.390285
C	-2.936650	9.807181	-0.835932
C	-2.630237	11.138535	-1.083036
C	-2.024137	11.907288	-0.103786
C	-1.687278	11.357559	1.133268
C	0.246626	8.411571	5.468163
C	1.060318	9.433107	5.980549
C	1.997165	9.087745	6.955772
C	2.103160	7.783196	7.412024
C	1.271137	6.792444	6.904713
C	0.333341	7.083961	5.918044
C	-3.098397	7.805747	0.716691
C	-4.486446	7.845277	1.382172
C	-1.025258	12.221087	2.194671

C	0.427510	12.525398	1.821065
C	0.902245	10.883849	5.551714
C	0.415056	11.745789	6.726802
C	-0.560640	6.009612	5.319642
C	0.104228	4.630313	5.260514
C	-3.129060	6.867006	-0.491772
C	-1.818090	13.503808	2.476283
C	2.177073	11.460121	4.927418
C	-1.900515	5.925169	6.069393
C	4.800457	9.093106	3.999428
C	2.966191	11.035160	-2.823147
C	-0.113527	7.452003	-2.876208
C	4.161954	3.461177	0.740719
N	1.868614	7.351721	3.482754
N	2.743636	6.701332	3.657609
H	-3.494122	10.233106	3.584158
H	-2.267679	9.334586	5.866936
H	5.330549	8.381967	-0.928114
H	4.448592	5.949631	-1.890526
H	-3.428868	9.226757	-1.604063
H	-2.873048	11.578745	-2.042773
H	-1.797777	12.946750	-0.304997
H	-2.399463	7.391494	1.441978
H	-3.914519	7.144504	-1.199183
H	-3.328681	5.847519	-0.159541
H	-2.177109	6.860960	-1.017322
H	-5.224616	8.278565	0.702187
H	-4.479352	8.436957	2.297081
H	-4.811743	6.834270	1.638687
H	-1.003437	11.654381	3.123192
H	-1.833274	14.170423	1.611227
H	-1.362040	14.050454	3.305106
H	-2.852807	13.280502	2.744886
H	0.996512	11.602702	1.717792
H	0.905623	13.133072	2.592332
H	0.486019	13.072107	0.877790
H	2.643661	9.852469	7.368028
H	2.835771	7.534818	8.170458
H	1.370948	5.779703	7.270708
H	0.133538	10.922518	4.782751
H	2.423810	10.935501	4.006324

H	3.029642	11.390678	5.606497
H	2.031171	12.514094	4.681167
H	1.158066	11.785305	7.527242
H	-0.510393	11.349792	7.149000
H	0.227074	12.769141	6.393635
H	-0.774674	6.311524	4.292180
H	-1.738120	5.654531	7.116057
H	-2.542551	5.165163	5.617689
H	-2.434848	6.874526	6.042050
H	0.213236	4.188919	6.254092
H	1.090664	4.681988	4.801025
H	-0.509624	3.949836	4.667568
H	4.985168	11.601584	2.902181
H	4.045263	13.390650	1.504246
H	2.814902	12.841110	-0.554119
H	4.470912	7.962564	2.223691
H	5.222197	8.227877	4.514168
H	3.760806	9.184944	4.300798
H	5.335875	9.978081	4.350944
H	6.560614	8.627344	1.035301
H	6.911801	8.010472	2.651901
H	6.942686	9.751876	2.339411
H	2.285702	9.325624	-1.758850
H	2.946160	12.125989	-2.775831
H	2.462074	10.734027	-3.744461
H	4.010323	10.721704	-2.887800
H	0.272579	10.566280	-2.447361
H	0.696806	11.894174	-1.367214
H	0.299002	10.309939	-0.701891
H	-0.226306	4.268970	-3.455685
H	-0.199110	2.269344	-2.028973
H	1.191942	2.198863	-0.004975
H	2.003565	7.227020	-2.867980
H	-0.110394	8.344535	-3.505434
H	-0.164925	7.773688	-1.835660
H	-1.014826	6.879111	-3.106037
H	0.416677	5.732603	-5.003565
H	2.169619	5.654140	-4.808366
H	1.383057	7.188513	-5.207332
H	3.031661	5.187569	1.289816
H	4.006946	2.420457	0.444886
H	4.783375	3.471319	1.638894
H	4.713261	3.959795	-0.058594
H	1.120311	3.974574	2.360604
H	2.690737	3.654508	3.107387
H	1.951986	2.443482	2.063865

Int2_2

136

B	-0.842838	0.118347	-0.182038
B	0.842543	-0.118453	-0.182307
C	-1.713998	1.283009	0.247234
N	-1.335380	2.451208	0.910391
C	-2.456768	3.216946	1.225588
H	-2.360856	4.153372	1.743120
C	-3.534383	2.591212	0.733484
H	-4.573693	2.860920	0.747308
N	-3.099892	1.425364	0.112155
C	1.713906	-1.282991	0.246738
N	3.099800	-1.425263	0.111511
C	3.534333	-2.591257	0.732572
H	4.573645	-2.860913	0.746287
C	2.456797	-3.217136	1.224658
H	2.360947	-4.153694	1.741981
N	1.335379	-2.451357	0.909755
C	-0.043492	3.085378	0.940600
C	0.253524	4.034029	-0.051193
C	1.440790	4.755619	0.073019
H	1.700489	5.484579	-0.683687
C	2.292914	4.550300	1.143878
H	3.208789	5.122644	1.225389
C	1.979775	3.603042	2.108685
H	2.651513	3.454306	2.942061
C	0.813331	2.847481	2.022883
C	-0.672852	4.316746	-1.224823
H	-1.531998	3.654854	-1.148908
C	0.000000	4.030755	-2.574140
H	0.891020	4.644912	-2.716177
H	-0.691040	4.250787	-3.390416
H	0.303035	2.987600	-2.652384
C	-1.213348	5.753749	-1.171424
H	-0.409714	6.485394	-1.280191
H	-1.718969	5.949067	-0.223834

H	-1.929696	5.919662	-1.979215
C	0.445549	1.850495	3.104997
H	-0.262842	1.149696	2.665591
C	-0.254322	2.565279	4.271429
H	-1.155234	3.080762	3.933957
H	0.409168	3.306129	4.724980
H	-0.543740	1.851541	5.045959
C	1.645264	1.035838	3.594988
H	2.122327	0.519383	2.761839
H	1.320785	0.288107	4.319801
H	2.392497	1.659801	4.088785
C	-3.998614	0.640593	-0.682683
C	-4.965953	-0.142310	-0.033058
C	-5.884574	-0.833930	-0.821175
H	-6.643958	-1.440538	-0.344821
C	-5.832358	-0.770230	-2.204434
H	-6.548634	-1.322245	-2.800492
C	-4.858272	-0.002897	-2.824296
H	-4.823838	0.041552	-3.905152
C	-3.928991	0.724250	-2.083439
C	-5.031621	-0.260918	1.480392
H	-4.135325	0.203856	1.890736
C	-5.029863	-1.726880	1.930992
H	-5.957914	-2.232988	1.656272
H	-4.930340	-1.787715	3.017225
H	-4.202041	-2.269468	1.478229
C	-6.248122	0.481924	2.053385
H	-7.178333	0.048576	1.678516
H	-6.241454	1.538517	1.781352
H	-6.259424	0.413935	3.143801
C	-2.916147	1.597642	-2.804505
H	-2.206100	1.967023	-2.070772
C	-3.610100	2.814181	-3.435823
H	-2.878106	3.460856	-3.924146
H	-4.134495	3.403198	-2.680330
H	-4.340544	2.505727	-4.187089
C	-2.097282	0.816639	-3.840930
H	-1.332427	1.464383	-4.274440
H	-2.722113	0.448581	-4.657702
H	-1.600106	-0.036438	-3.380582
C	3.998522	-0.640194	-0.682922

C	4.965874	0.142478	-0.033010
C	5.884532	0.834349	-0.820838
H	6.643870	1.440796	-0.344266
C	5.832374	0.771129	-2.204109
H	6.548700	1.323327	-2.799946
C	4.858359	0.003976	-2.824298
H	4.824000	-0.040127	-3.905128
C	3.929025	-0.723374	-2.083747
C	5.031540	0.260461	1.480515
H	4.135104	-0.204252	1.890651
C	5.030205	1.726237	1.931718
H	4.202619	2.269270	1.479085
H	5.958442	2.232163	1.657374
H	4.930543	1.786636	3.017977
C	6.247847	-0.482909	2.053201
H	7.178164	-0.049673	1.678523
H	6.240925	-1.539406	1.780771
H	6.259169	-0.415363	3.143654
C	2.916300	-1.596695	-2.805077
H	2.205630	-1.965445	-2.071630
C	3.610289	-2.813730	-3.435351
H	4.133834	-3.402684	-2.679185
H	4.341461	-2.505842	-4.186110
H	2.878457	-3.460345	-3.924022
C	2.098411	-0.815905	-3.842422
H	1.333403	-1.463525	-4.275880
H	2.723810	-0.448740	-4.659113
H	1.601471	0.037753	-3.382861
C	0.043593	-3.085703	0.939689
C	-0.253337	-4.033996	-0.052488
C	-1.440461	-4.755868	0.071524
H	-1.700092	-5.484606	-0.685475
C	-2.292585	-4.551190	1.142514
H	-3.208352	-5.123705	1.223849
C	-1.979582	-3.604186	2.107618
H	-2.651307	-3.455854	2.941086
C	-0.813264	-2.848361	2.022085
C	0.672868	-4.315908	-1.226449
H	1.532057	-3.654051	-1.150172
C	-0.000205	-4.028865	-2.575411
H	-0.891263	-4.642940	-2.717738

H	0.690701	-4.248264	-3.392011
H	-0.303285	-2.985650	-2.652782
C	1.213421	-5.752905	-1.174163
H	0.409817	-6.484523	-1.283350
H	1.719212	-5.948923	-0.226815
H	1.929650	-5.918215	-1.982216
C	-0.445656	-1.851760	3.104579
H	0.262811	-1.150801	2.665507
C	0.254053	-2.566916	4.270886
H	1.155024	-3.082293	3.933375
H	-0.409507	-3.307908	4.724111
H	0.543363	-1.853415	5.045692
C	-1.645458	-1.037254	3.594645
H	-1.321086	-0.289778	4.319767
H	-2.392767	-1.661383	4.088114
H	-2.122378	-0.520530	2.761593
N	1.589713	0.933666	-0.784082
N	2.118555	1.818937	-1.250128
N	-1.590361	-0.933693	-0.783417
N	-2.119512	-1.818987	-1.249184

RC_{2LiCl}

136

Li	1.851238	0.994509	-1.466155
Cl	3.361769	0.782472	-2.960677
B	0.490081	-0.332146	-0.077505
B	-0.384765	0.823479	-0.053617
C	1.354293	-1.550157	-0.135142
N	2.461991	-1.845018	0.630237
C	3.009401	-3.056638	0.235914
H	3.884021	-3.459024	0.711334
C	2.245875	-3.538940	-0.763759
H	2.320244	-4.447362	-1.331569
N	1.226806	-2.626370	-0.983798
C	-1.223381	2.053913	-0.074414
N	-0.818180	3.348679	-0.325487
C	-1.892706	4.220252	-0.255364
H	-1.774180	5.275600	-0.420278
C	-2.985571	3.485571	0.035876
H	-4.013862	3.770806	0.158103
N	-2.578517	2.164707	0.149664
C	2.962278	-1.092265	1.750814
C	2.294268	-1.199131	2.985415
C	2.865010	-0.556446	4.080475
H	2.381839	-0.610765	5.045462
C	4.050334	0.155991	3.958483
H	4.479173	0.640655	4.827294
C	4.673621	0.262934	2.728660
H	5.585824	0.837440	2.640559
C	4.139452	-0.348572	1.592225
C	1.016359	-2.012108	3.135298
H	0.438681	-1.878322	2.220833
C	0.122331	-1.532809	4.281027
H	-0.083298	-0.464800	4.202586
H	0.566147	-1.728665	5.259780
H	-0.828114	-2.067141	4.248753
C	1.324036	-3.509807	3.300575
H	1.894058	-3.904529	2.460370
H	0.395983	-4.081270	3.370914
H	1.900254	-3.683355	4.212872
C	4.838442	-0.194724	0.253009
H	4.152190	-0.494971	-0.538877
C	6.076542	-1.100346	0.160744
H	6.543417	-0.994783	-0.820458
H	5.822119	-2.152426	0.300843
H	6.812427	-0.830044	0.922285
C	5.223853	1.263434	-0.030734
H	6.045350	1.597179	0.608742
H	4.378265	1.932994	0.125811
H	5.527097	1.362797	-1.072043
C	0.075008	-2.935299	-1.790262
C	0.135888	-2.731867	-3.175218
C	-0.956623	-3.153082	-3.933561
H	-0.940959	-3.015432	-5.006244
C	-2.067016	-3.726591	-3.334742
H	-2.907564	-4.038453	-3.943117
C	-2.112543	-3.887826	-1.958225
H	-2.992048	-4.320274	-1.498630
C	-1.038739	-3.508100	-1.155263
C	1.339367	-2.091062	-3.839453
H	1.905454	-1.550472	-3.081518
C	0.941874	-1.059193	-4.901758
H	1.821193	-0.482380	-5.186529

H	0.199596	-0.362978	-4.511616
H	0.529498	-1.532102	-5.796807
C	2.276532	-3.150150	-4.440217
H	3.154040	-2.666880	-4.874036
H	1.770826	-3.718291	-5.225664
H	2.622436	-3.857736	-3.684445
C	-1.105240	-3.716181	0.348550
H	-0.132447	-3.467783	0.767959
C	-1.380049	-5.180645	0.717529
H	-0.635458	-5.846189	0.276001
H	-2.365308	-5.505235	0.377146
H	-1.347341	-5.305764	1.802368
C	-2.132885	-2.773006	0.988007
H	-1.909800	-1.734254	0.743230
H	-2.117506	-2.882020	2.074707
H	-3.143890	-2.996898	0.643103
C	0.534117	3.726643	-0.606015
C	0.913022	3.925859	-1.946029
C	2.248900	4.241561	-2.192060
H	2.595157	4.354906	-3.208705
C	3.154166	4.377627	-1.149807
H	4.190627	4.601066	-1.367383
C	2.743686	4.209204	0.163549
H	3.461058	4.323524	0.966246
C	1.425243	3.871252	0.467387
C	-0.079472	3.759372	-3.089976
H	-1.077620	3.964772	-2.698300
C	-0.079825	2.314217	-3.613243
H	-0.801859	2.204281	-4.426248
H	-0.355619	1.606681	-2.828400
H	0.907273	2.036172	-3.987650
C	0.161358	4.747380	-4.237316
H	1.083997	4.524540	-4.776255
H	0.218246	5.776549	-3.877018
H	-0.656981	4.681099	-4.957139
C	0.995742	3.687527	1.913248
H	-0.047435	3.372209	1.919042
C	1.080128	5.014978	2.682177
H	0.476623	5.790701	2.205634
H	2.109370	5.376880	2.733469
H	0.720711	4.883006	3.705352
C	1.801553	2.583473	2.607888
H	1.438138	2.434680	3.627209
H	2.861809	2.835421	2.670063
H	1.701296	1.640855	2.070167
C	-3.476525	1.056743	0.291871
C	-3.867973	0.665094	1.579187
C	-4.795884	-0.369346	1.683044
H	-5.127989	-0.698101	2.658074
C	-5.290482	-0.998294	0.548986
H	-6.004629	-1.806268	0.651655
C	-4.862206	-0.610125	-0.710818
H	-5.239992	-1.122401	-1.586412
C	-3.947057	0.430337	-0.869426
C	-3.313634	1.355360	2.814174
H	-2.356416	1.800585	2.535319
C	-3.039863	0.377139	3.961064
H	-3.964953	0.001837	4.403825
H	-2.478227	0.875322	4.753768
H	-2.456805	-0.474194	3.614859
C	-4.244414	2.485746	3.282345
H	-3.826438	2.988096	4.157822
H	-5.224097	2.085863	3.555076
H	-4.392515	3.235326	2.504267
C	-3.491460	0.844962	-2.257958
H	-2.794594	1.675438	-2.154525
C	-4.666540	1.343644	-3.111423
H	-4.305433	1.687792	-4.083150
H	-5.185315	2.173733	-2.626579
H	-5.396354	0.550741	-3.289455
C	-2.734436	-0.294451	-2.948754
H	-2.363425	0.031550	-3.922511
H	-3.377498	-1.160440	-3.109478
H	-1.882227	-0.610745	-2.346401
N	-4.514139	-2.987942	4.229687
N	-3.534396	-3.443712	4.378608

TS1_2$_{LiCl}$

136

Li	1.907887	1.429564	-1.541371
Cl	3.299461	1.779950	-3.143340
B	0.709330	-0.088258	-0.247158
B	-0.559590	0.564478	0.240268

C	1.597903	-1.276055	-0.388953
N	2.759751	-1.550943	0.321793
C	3.363650	-2.706459	-0.162151
H	4.275536	-3.081573	0.262171
C	2.604998	-3.171009	-1.166220
H	2.728177	-4.026856	-1.802701
N	1.514690	-2.316069	-1.306449
C	-1.502760	1.750941	0.292990
N	-1.161882	3.066358	0.072948
C	-2.268985	3.891084	0.204758
H	-2.191249	4.955165	0.078736
C	-3.319951	3.106678	0.500238
H	-4.355754	3.341790	0.657560
N	-2.858799	1.798016	0.553732
C	3.277690	-0.838332	1.457162
C	2.770478	-1.141375	2.733750
C	3.381354	-0.543121	3.833684
H	3.019843	-0.756500	4.829720
C	4.448292	0.328172	3.673810
H	4.910506	0.780461	4.543035
C	4.914695	0.630430	2.406247
H	5.738728	1.321251	2.293596
C	4.341593	0.058305	1.268774
C	1.620429	-2.117045	2.925050
H	1.006591	-2.073660	2.026584
C	0.717256	-1.752985	4.108755
H	0.372911	-0.719738	4.040981
H	1.229083	-1.880698	5.064786
H	-0.158155	-2.402639	4.118126
C	2.134542	-3.559012	3.069437
H	2.718087	-3.865398	2.201174
H	1.296301	-4.252435	3.171350
H	2.766262	-3.654510	3.955932
C	4.896280	0.390172	-0.105845
H	4.128926	0.175188	-0.851255
C	6.115588	-0.482745	-0.445140
H	6.483415	-0.232270	-1.441928
H	5.873724	-1.545627	-0.434657
H	6.922241	-0.311774	0.272692
C	5.263863	1.872262	-0.249255
H	6.168011	2.119201	0.313221

H	4.457158	2.515858	0.101844
H	5.432473	2.103332	-1.299695
C	0.429531	-2.644254	-2.194994
C	0.471375	-2.181208	-3.519948
C	-0.498189	-2.655842	-4.403372
H	-0.490734	-2.319239	-5.430361
C	-1.469702	-3.551773	-3.988157
H	-2.208013	-3.914199	-4.693384
C	-1.510651	-3.970311	-2.668287
H	-2.287753	-4.653198	-2.354153
C	-0.570348	-3.522574	-1.740731
C	1.539825	-1.214859	-3.994105
H	1.834478	-0.598091	-3.145322
C	1.045728	-0.262859	-5.088202
H	1.769647	0.542544	-5.206727
H	0.082378	0.178211	-4.828974
H	0.938523	-0.771913	-6.049552
C	2.805043	-1.943938	-4.473172
H	3.556870	-1.208793	-4.763785
H	2.581131	-2.579503	-5.334331
H	3.235944	-2.569085	-3.691192
C	-0.637830	-4.007195	-0.300748
H	-0.136615	-3.264157	0.320272
C	0.101590	-5.343358	-0.119705
H	1.153134	-5.263029	-0.390499
H	-0.353383	-6.118874	-0.741171
H	0.048240	-5.668129	0.922192
C	-2.080062	-4.139165	0.207832
H	-2.664791	-3.241290	0.006885
H	-2.079042	-4.305514	1.285544
H	-2.590129	-4.987349	-0.253761
C	0.159353	3.582751	-0.138458
C	0.514753	4.001185	-1.434743
C	1.800692	4.513038	-1.607832
H	2.139132	4.796224	-2.592125
C	2.673727	4.629435	-0.534499
H	3.673717	5.008404	-0.700459
C	2.278406	4.248078	0.737668
H	2.966799	4.356019	1.566132
C	1.009616	3.716666	0.967479
C	-0.459122	3.880271	-2.603083

H	-1.462347	4.070051	-2.215125
C	-0.455983	2.460897	-3.193400
H	-1.217165	2.371705	-3.972560
H	-0.659497	1.700545	-2.437058
H	0.514864	2.244913	-3.643583
C	-0.204863	4.909706	-3.709058
H	0.724878	4.703303	-4.241896
H	-0.154960	5.925836	-3.312594
H	-1.014012	4.868154	-4.441031
C	0.584359	3.340791	2.376636
H	-0.399250	2.875311	2.323491
C	0.452651	4.592445	3.257887
H	-0.255708	5.306419	2.831467
H	1.413531	5.100033	3.368333
H	0.101825	4.318524	4.255749
C	1.532209	2.309875	2.997952
H	1.164604	1.998809	3.978407
H	2.537397	2.711671	3.135437
H	1.602837	1.426907	2.364769
C	-3.754153	0.681042	0.653371
C	-4.239790	0.312194	1.917185
C	-5.116294	-0.768151	1.978756
H	-5.509288	-1.084626	2.935812
C	-5.476376	-1.461255	0.831039
H	-6.144850	-2.310428	0.902277
C	-4.986405	-1.069632	-0.405653
H	-5.282161	-1.612808	-1.293990
C	-4.125529	0.021005	-0.526718
C	-3.826731	1.054362	3.178944
H	-2.936977	1.640257	2.940399
C	-3.454114	0.116478	4.335496
H	-4.322077	-0.433331	4.704573
H	-3.058835	0.698643	5.170656
H	-2.697287	-0.608000	4.037849
C	-4.927859	2.033227	3.619231
H	-4.614093	2.592286	4.503769
H	-5.844396	1.492350	3.867437
H	-5.167217	2.750628	2.833559
C	-3.655800	0.475141	-1.898955
H	-2.977701	1.316299	-1.767106
C	-4.839882	0.976816	-2.740624
H	-4.482741	1.361639	-3.698366
H	-5.378832	1.778050	-2.230197
H	-5.550448	0.173302	-2.946709
C	-2.873473	-0.619694	-2.630269
H	-2.518482	-0.250786	-3.593898
H	-3.491142	-1.498896	-2.819846
H	-2.003399	-0.930119	-2.052184
N	-1.464633	-0.708773	1.354656
N	-1.518322	-1.656374	1.921265

Int1_2$_{LiCl}$

136

Li	2.116660	0.924517	-1.635456
Cl	3.488411	1.034706	-3.291502
B	0.675739	-0.194576	-0.215636
B	-0.677729	0.418254	0.366905
C	1.245761	-1.583010	-0.058975
N	2.294499	-1.955325	0.769534
C	2.642110	-3.285151	0.554799
H	3.439136	-3.750562	1.103687
C	1.838964	-3.761457	-0.405879
H	1.809264	-4.720029	-0.887919
N	0.968604	-2.737133	-0.782053
C	-1.436576	1.757376	0.225971
N	-0.943464	2.988709	-0.108642
C	-1.948741	3.944558	-0.083163
H	-1.744657	4.975409	-0.307257
C	-3.089629	3.315387	0.254980
H	-4.094893	3.677463	0.363470
N	-2.775424	1.978308	0.441302
C	2.912905	-1.171625	1.801896
C	2.397516	-1.252957	3.105276
C	3.101698	-0.613173	4.125195
H	2.736914	-0.668446	5.142205
C	4.259947	0.096398	3.854502
H	4.793552	0.587230	4.659516
C	4.732476	0.189899	2.554117
H	5.630562	0.758142	2.356480
C	4.076142	-0.443896	1.499537
C	1.135446	-2.033691	3.427127
H	0.608495	-2.219813	2.492264
C	0.181020	-1.247052	4.333674

H	-0.045701	-0.268944	3.908139
H	0.600583	-1.096591	5.330613
H	-0.756870	-1.791391	4.446229
C	1.475638	-3.399560	4.043359
H	2.100687	-3.991434	3.372872
H	0.562910	-3.966042	4.242902
H	2.013296	-3.277044	4.986922
C	4.639209	-0.375535	0.089837
H	3.809798	-0.501526	-0.609730
C	5.626986	-1.523288	-0.177412
H	5.999554	-1.457890	-1.201306
H	5.159163	-2.499802	-0.052829
H	6.478876	-1.462755	0.504891
C	5.305784	0.968888	-0.222877
H	6.262253	1.072018	0.295690
H	4.668500	1.805124	0.067476
H	5.481447	1.046068	-1.294898
C	-0.097332	-3.006917	-1.713772
C	0.054925	-2.632807	-3.062262
C	-0.917186	-3.060254	-3.965242
H	-0.830464	-2.788627	-5.006990
C	-1.990708	-3.836849	-3.556787
H	-2.725063	-4.167569	-4.281375
C	-2.130770	-4.177231	-2.223511
H	-2.980502	-4.769971	-1.912597
C	-1.196021	-3.766617	-1.271570
C	1.242251	-1.811443	-3.532966
H	1.434013	-1.060144	-2.764253
C	0.986592	-1.059639	-4.842293
H	1.796455	-0.349085	-5.001690
H	0.045536	-0.507623	-4.815109
H	0.957409	-1.740522	-5.697016
C	2.522207	-2.651819	-3.667628
H	3.348094	-1.996791	-3.948244
H	2.397094	-3.417439	-4.438301
H	2.791279	-3.147588	-2.735539
C	-1.395265	-4.173631	0.180583
H	-0.760189	-3.538586	0.798450
C	-0.977101	-5.634491	0.419818
H	0.070425	-5.805852	0.174919
H	-1.581463	-6.310042	-0.190472

H	-1.124560	-5.902455	1.468707
C	-2.845888	-3.971155	0.644455
H	-3.217156	-2.980151	0.388907
H	-2.908031	-4.079945	1.728416
H	-3.513299	-4.712996	0.199860
C	0.429475	3.331326	-0.355540
C	0.832395	3.555216	-1.684842
C	2.170621	3.886117	-1.894895
H	2.543874	4.011231	-2.899097
C	3.050799	4.014440	-0.827162
H	4.090124	4.245385	-1.020986
C	2.608846	3.835617	0.473352
H	3.303093	3.955737	1.295209
C	1.282949	3.492819	0.741155
C	-0.144741	3.415675	-2.847849
H	-1.125306	3.741695	-2.494366
C	-0.279862	1.951114	-3.293972
H	-1.048439	1.858376	-4.065361
H	-0.548859	1.293115	-2.466226
H	0.664034	1.600333	-3.715927
C	0.215929	4.300536	-4.045575
H	1.121488	3.949996	-4.543252
H	0.367617	5.340575	-3.749416
H	-0.591053	4.269603	-4.780623
C	0.808239	3.342958	2.175971
H	-0.219632	2.981378	2.160446
C	0.805607	4.703230	2.890948
H	0.179089	5.427335	2.365352
H	1.814728	5.116585	2.951648
H	0.424238	4.596659	3.909252
C	1.634484	2.306796	2.942672
H	1.220880	2.153505	3.942004
H	2.672210	2.621976	3.061757
H	1.631572	1.350833	2.422057
C	-3.773435	0.961511	0.626650
C	-4.245695	0.703611	1.920768
C	-5.219338	-0.282248	2.063193
H	-5.607786	-0.512674	3.046009
C	-5.680782	-0.992774	0.963708
H	-6.424546	-1.768495	1.097888
C	-5.188935	-0.720426	-0.303618

H	-5.553821	-1.284829	-1.151912
C	-4.229963	0.272314	-0.504795
C	-3.720039	1.465977	3.126555
H	-2.772532	1.925924	2.838118
C	-3.431903	0.558366	4.329454
H	-4.350192	0.150558	4.757148
H	-2.933614	1.130700	5.114554
H	-2.787065	-0.274423	4.051327
C	-4.686161	2.595795	3.518763
H	-4.292032	3.161183	4.366295
H	-5.657678	2.186433	3.805503
H	-4.847959	3.291867	2.693964
C	-3.730924	0.579459	-1.907008
H	-2.990153	1.375508	-1.841966
C	-4.870703	1.097112	-2.797744
H	-4.483423	1.370265	-3.781725
H	-5.346865	1.978064	-2.361843
H	-5.640856	0.336142	-2.941644
C	-3.034578	-0.631126	-2.536610
H	-2.644928	-0.372977	-3.522546
H	-3.719812	-1.470748	-2.659470
H	-2.197380	-0.965404	-1.924465
N	-1.365472	-0.533209	1.207402
N	-1.784210	-1.343254	1.867344

In Implicit Toluene:

System (1)

106

C	-3.944059	-1.745469	-2.184090
C	-4.420187	-0.853115	-1.022635
C	-2.071333	-0.475212	-1.380059
C	-2.555289	-1.225954	-2.622766
H	-4.659646	-1.754562	-3.007375
H	-3.845496	-2.771199	-1.823564
N	-3.113750	-0.334757	-0.514590
C	-5.333695	0.287414	-1.498323
H	-6.248205	-0.129101	-1.925453
H	-5.614146	0.926712	-0.661736
H	-4.855550	0.904177	-2.256450
C	-2.648223	-0.262995	-3.821643
H	-1.655619	0.109084	-4.078471
H	-3.066976	-0.781030	-4.689282
H	-3.278406	0.598660	-3.602793
C	-1.618690	-2.383184	-2.990701
H	-2.027547	-2.945639	-3.835144
H	-0.633727	-2.007536	-3.270056
H	-1.488991	-3.066021	-2.149325
C	-5.175845	-1.655948	0.038045
H	-5.398820	-1.043266	0.913038
H	-6.121638	-2.003693	-0.381218
H	-4.610609	-2.527598	0.360035
C	-2.940353	0.231640	0.791030
C	-3.087517	1.617027	0.989315
C	-2.586235	-0.619352	1.859311
C	-2.995143	2.113266	2.290793
C	-2.504285	-0.073880	3.138924
C	-2.730100	1.275651	3.361256
H	-3.110691	3.175887	2.464034
H	-2.236405	-0.710982	3.972073
H	-2.663025	1.679690	4.364213
C	-3.235575	2.597688	-0.162590
H	-3.345725	2.022857	-1.079052
C	-2.202915	-2.077167	1.653542
H	-2.447120	-2.347100	0.627588
C	-1.949715	3.428985	-0.311268
H	-2.020024	4.079819	-1.186789
H	-1.784188	4.059429	0.566412
H	-1.087972	2.773122	-0.435977
C	-4.457990	3.514054	-0.019612
H	-4.572248	4.132307	-0.913318
H	-5.378182	2.944790	0.118785
H	-4.352137	4.186638	0.834446
C	-0.684091	-2.253626	1.813561
H	-0.139323	-1.607217	1.126477
H	-0.367104	-2.014263	2.830604
H	-0.397529	-3.287060	1.604831
C	-2.956669	-3.032272	2.590314
H	-4.037203	-2.891945	2.531209
H	-2.731249	-4.068676	2.327900
H	-2.659485	-2.887123	3.631269
B	0.688119	0.483425	-1.110589
B	-0.717820	0.012213	-1.202246
C	2.044429	0.991051	-1.107929

C	2.501254	2.232718	-1.874455
C	4.035076	2.044839	-1.914837
C	4.421175	1.139907	-0.728273
H	4.567171	2.996084	-1.883930
H	4.307927	1.547061	-2.847574
N	3.112326	0.480837	-0.430296
C	2.966127	-0.556947	0.549358
C	2.652451	-0.214416	1.881863
C	3.104997	-1.905448	0.167164
C	2.615935	-1.231225	2.834963
C	3.058323	-2.885180	1.159589
C	2.843013	-2.553238	2.486896
H	2.380517	-0.986774	3.863171
H	3.169790	-3.926348	0.884028
H	2.812039	-3.327935	3.243530
C	1.906575	2.304153	-3.284466
H	2.141543	1.402232	-3.851769
H	0.819997	2.394650	-3.239227
H	2.306427	3.169782	-3.820316
C	2.101414	3.504627	-1.100178
H	1.016224	3.594647	-1.054364
H	2.476344	3.495040	-0.077547
H	2.504279	4.389666	-1.600711
C	5.489591	0.118634	-1.130340
H	6.418845	0.639889	-1.369410
H	5.692855	-0.577587	-0.315403
H	5.185897	-0.450239	-2.007429
C	4.954438	1.933419	0.473091
H	5.112104	1.273592	1.326504
H	5.914479	2.384239	0.214145
H	4.279160	2.730955	0.774874
C	3.187880	-2.338272	-1.287749
H	3.267494	-1.440838	-1.898557
C	2.249684	1.192402	2.295211
H	2.430269	1.852143	1.451723
C	0.741510	1.246078	2.585201
H	0.434915	2.268987	2.815623
H	0.164924	0.907944	1.725139
H	0.481711	0.617283	3.439155
C	3.047613	1.724152	3.493468
H	4.123313	1.678646	3.319381
H	2.778736	2.765238	3.688206
H	2.831440	1.155667	4.400715
C	1.880682	-3.038423	-1.695243
H	1.748606	-3.973601	-1.144581
H	1.024447	-2.394916	-1.495628
H	1.894604	-3.274548	-2.762489
C	4.396615	-3.236736	-1.584154
H	4.452224	-3.449685	-2.654498
H	5.333981	-2.769500	-1.281203
H	4.317300	-4.193403	-1.063279

N_2

N	0.000000	0.000000	0.164552
N	0.000000	0.000000	1.255448

RC

108

C	-3.966254	-1.643107	-2.267192
C	-4.440819	-0.793842	-1.073256
C	-2.091655	-0.409877	-1.410012
C	-2.571154	-1.117466	-2.679343
H	-4.677684	-1.614026	-3.093565
H	-3.877337	-2.683028	-1.947052
N	-3.132942	-0.308857	-0.538153
C	-5.337821	0.374783	-1.509780
H	-6.253629	-0.013743	-1.959690
H	-5.617319	0.984142	-0.650833
H	-4.846098	1.015025	-2.239314
C	-2.645372	-0.120615	-3.850963
H	-1.648765	0.256111	-4.083730
H	-3.051856	-0.613230	-4.738887
H	-3.277600	0.735421	-3.616583
C	-1.638825	-2.270523	-3.072091
H	-2.046839	-2.808677	-3.932541
H	-0.651617	-1.893667	-3.338720
H	-1.516874	-2.975343	-2.247959
C	-5.210221	-1.628310	-0.047927
H	-5.433240	-1.046399	0.847691
H	-6.156285	-1.951803	-0.485459
H	-4.654502	-2.515780	0.245810
C	-2.958396	0.192731	0.793606
C	-3.101633	1.566343	1.061733
C	-2.606869	-0.712623	1.817181

C	-3.010796	1.995147	2.387463
C	-2.525553	-0.233710	3.123118
C	-2.750146	1.103264	3.414123
H	-3.123103	3.047982	2.613874
H	-2.259373	-0.913062	3.922690
H	-2.684071	1.455057	4.436715
C	-3.240481	2.606447	-0.038334
H	-3.346918	2.080955	-0.984392
C	-2.225574	-2.158737	1.536788
H	-2.478917	-2.378625	0.501151
C	-1.950635	3.439130	-0.134753
H	-2.010877	4.135533	-0.975437
H	-1.788657	4.021610	0.775972
H	-1.091112	2.786527	-0.287301
C	-4.459897	3.519866	0.145876
H	-4.565101	4.185644	-0.714227
H	-5.383977	2.949198	0.247859
H	-4.356649	4.145338	1.035174
C	-0.705363	-2.341966	1.675787
H	-0.166094	-1.665019	1.014145
H	-0.379757	-2.147118	2.699493
H	-0.420128	-3.365428	1.420886
C	-2.971355	-3.158948	2.431896
H	-4.052038	-3.012940	2.392629
H	-2.751909	-4.181064	2.114296
H	-2.661701	-3.068136	3.475141
B	0.654466	0.562807	-1.076859
B	-0.743124	0.078437	-1.206302
C	2.005144	1.078944	-1.029608
C	2.461028	2.378956	-1.693771
C	3.991916	2.181755	-1.769108
C	4.383987	1.190326	-0.654768
H	4.534188	3.123608	-1.678021
H	4.247925	1.748911	-2.738140
N	3.072268	0.525476	-0.383492
C	2.924856	-0.559004	0.544293
C	2.617188	-0.278272	1.893294
C	3.059002	-1.889456	0.102074
C	2.582669	-1.336958	2.799518
C	3.018541	-2.912899	1.050061
C	2.809383	-2.642010	2.391730
H	2.349825	-1.138637	3.838174
H	3.127794	-3.940269	0.726272
H	2.781447	-3.450160	3.112684
C	1.845449	2.585382	-3.080427
H	2.086624	1.754447	-3.742930
H	0.758269	2.652313	-3.012100
H	2.223084	3.508403	-3.528999
C	2.083684	3.581127	-0.804058
H	1.000241	3.676013	-0.734815
H	2.472446	3.480179	0.208031
H	2.487772	4.502921	-1.231521
C	5.430673	0.187077	-1.147245
H	6.359948	0.713227	-1.375322
H	5.645073	-0.561753	-0.383578
H	5.098919	-0.321226	-2.051074
C	4.946573	1.888309	0.591519
H	5.099422	1.168002	1.395697
H	5.912850	2.336249	0.352204
H	4.292203	2.677196	0.956155
C	3.124101	-2.261404	-1.370422
H	3.188585	-1.339488	-1.944726
C	2.218382	1.108431	2.372509
H	2.404778	1.807219	1.562813
C	0.709066	1.152254	2.658204
H	0.404137	2.162050	2.942563
H	0.135288	0.862562	1.778595
H	0.443608	0.478472	3.475087
C	3.014251	1.579779	3.596823
H	4.090250	1.538726	3.423002
H	2.748405	2.611420	3.839921
H	2.793796	0.970123	4.475710
C	1.815701	-2.955921	-1.782719
H	1.689689	-3.901906	-1.250108
H	0.959813	-2.318653	-1.562842
H	1.820702	-3.172776	-2.853333
C	4.332514	-3.138513	-1.723946
H	4.372874	-3.302224	-2.803531
H	5.272143	-2.680540	-1.414426
H	4.264775	-4.117829	-1.245431
N	1.484362	-0.775851	-4.871817
N	2.515989	-1.027313	-4.622697

TS_1

108

C	-4.374191	2.616346	-0.578263
C	-4.243411	1.552623	-1.688733
C	-2.213815	1.543486	-0.428362
C	-2.960787	2.811336	0.030013
H	-5.058138	2.246033	0.188303
H	-4.788453	3.551755	-0.957029
N	-3.089271	0.765213	-1.187540
C	-5.505610	0.706555	-1.839286
H	-5.803120	0.249715	-0.896753
H	-6.326152	1.338483	-2.184143
H	-5.359648	-0.088831	-2.572476
C	-3.027555	2.888809	1.564955
H	-3.618197	3.754809	1.880278
H	-3.482142	1.992527	1.986416
H	-2.024894	2.985305	1.986849
C	-2.289899	4.094575	-0.486811
H	-1.295235	4.201221	-0.052060
H	-2.179102	4.085227	-1.570558
H	-2.880844	4.972238	-0.208376
C	-3.925703	2.212999	-3.044344
H	-3.882842	1.471062	-3.840203
H	-4.701049	2.938183	-3.302254
H	-2.967774	2.730683	-3.011972
C	-2.968444	-0.655714	-1.269739
C	-3.468701	-1.449088	-0.210321
C	-2.348005	-1.266132	-2.375172
C	-3.350821	-2.834636	-0.294514
C	-2.281273	-2.660197	-2.428866
C	-2.774344	-3.442936	-1.400049
H	-3.720157	-3.451231	0.515367
H	-1.814910	-3.137749	-3.281755
H	-2.701156	-4.522704	-1.451805
C	-4.101925	-0.840761	1.030015
H	-4.314191	0.202634	0.810390
C	-1.709799	-0.463583	-3.493886
H	-1.780397	0.588577	-3.220971
C	-3.117059	-0.860593	2.206972
H	-2.222892	-0.293716	1.953747
H	-3.568887	-0.412819	3.096012
H	-2.823240	-1.883662	2.453325
C	-5.429681	-1.507501	1.415556
H	-6.122121	-1.542922	0.571943
H	-5.283047	-2.530630	1.767865
H	-5.905515	-0.948403	2.224650
C	-0.218248	-0.801070	-3.634084
H	0.244483	-0.160992	-4.388282
H	0.300125	-0.647048	-2.688633
H	-0.070845	-1.838723	-3.941627
C	-2.435379	-0.673759	-4.830825
H	-2.323040	-1.704353	-5.177162
H	-3.504050	-0.471430	-4.744950
H	-2.023333	-0.015742	-5.600174
B	0.638236	0.979333	0.139675
B	-0.845173	1.254931	-0.138302
C	2.016325	1.162217	-0.339685
C	2.308960	2.270929	-1.372476
C	3.854052	2.289084	-1.427305
C	4.342773	0.929696	-0.917700
H	4.228067	3.071352	-0.763591
H	4.224669	2.504780	-2.429725
N	3.182117	0.525737	-0.052221
C	3.284696	-0.515292	0.927773
C	3.660345	-0.162217	2.241030
C	2.956415	-1.846008	0.596321
C	3.769900	-1.173976	3.192557
C	3.088814	-2.819529	1.587160
C	3.500444	-2.495016	2.869399
H	4.050937	-0.923527	4.207556
H	2.841319	-3.847725	1.355585
H	3.590214	-3.267895	3.623107
C	1.687656	1.958857	-2.742692
H	1.939414	2.753349	-3.450619
H	2.049073	1.017044	-3.149484
H	0.603525	1.894422	-2.654499
C	1.785246	3.635788	-0.899119
H	2.129511	3.857696	0.112661
H	2.146318	4.421994	-1.569036
H	0.697216	3.646657	-0.897401
C	4.562207	-0.074865	-2.059201
H	5.354672	0.289586	-2.715408

H	4.871737	-1.041694	-1.665780
H	3.665538	-0.217070	-2.658609
C	5.652039	1.044682	-0.136360
H	5.916330	0.098818	0.338945
H	6.453557	1.308423	-0.827983
H	5.603998	1.817152	0.627423
C	2.386755	-2.261545	-0.752838
H	2.372585	-1.386956	-1.397624
C	3.864275	1.282026	2.679037
H	3.863252	1.906468	1.788525
C	2.697143	1.766659	3.556341
H	2.590029	1.144743	4.448038
H	2.875434	2.794925	3.880412
H	1.756806	1.746410	3.006519
C	5.201035	1.490758	3.405722
H	5.210327	0.987896	4.374654
H	6.040586	1.107944	2.823812
H	5.367406	2.555187	3.587127
C	0.931137	-2.745024	-0.627711
H	0.851335	-3.594183	0.054346
H	0.272264	-1.953663	-0.274622
H	0.558609	-3.062371	-1.602172
C	3.237599	-3.347629	-1.430301
H	3.175354	-4.292526	-0.886119
H	2.877096	-3.527191	-2.445768
H	4.290288	-3.068667	-1.487303
N	0.230748	-0.796705	2.560531
N	0.624540	-0.248314	1.692296

Int$_1$

108

C	-4.569234	2.205598	0.455958
C	-4.568369	1.043351	-0.562175
C	-2.330221	1.325764	0.234265
C	-3.086465	2.579281	0.722325
H	-5.034201	1.861996	1.382662
H	-5.147660	3.060779	0.101918
N	-3.276787	0.394483	-0.249157
C	-5.752184	0.098975	-0.364929
H	-5.799922	-0.276684	0.655804
H	-6.682321	0.628935	-0.578783
H	-5.687999	-0.757499	-1.038851
C	-2.845634	2.837251	2.220100
H	-3.441185	3.687675	2.568269
H	-3.110313	1.966567	2.819605
H	-1.792604	3.060992	2.405498
C	-2.680326	3.847666	-0.047435
H	-1.642038	4.105208	0.165903
H	-2.774301	3.716523	-1.124883
H	-3.304979	4.694602	0.252962
C	-4.599605	1.588005	-2.004971
H	-4.674053	0.777223	-2.728382
H	-5.461229	2.244682	-2.147557
H	-3.695846	2.155817	-2.224217
C	-3.049311	-1.009459	-0.166526
C	-3.195705	-1.673664	1.075221
C	-2.654648	-1.738930	-1.305930
C	-2.952531	-3.044091	1.141633
C	-2.458202	-3.117095	-1.198722
C	-2.598182	-3.769675	0.013431
H	-3.052854	-3.557020	2.090263
H	-2.168700	-3.683565	-2.075443
H	-2.425378	-4.837088	0.083261
C	-3.605911	-0.943335	2.341978
H	-3.929953	0.051810	2.049844
C	-2.401424	-1.068143	-2.643222
H	-2.485611	0.005773	-2.483204
C	-2.414539	-0.772945	3.294706
H	-1.627385	-0.195582	2.813352
H	-2.720933	-0.245868	4.202336
H	-2.005198	-1.742520	3.588684
C	-4.783853	-1.618845	3.059324
H	-5.622169	-1.790331	2.381364
H	-4.498736	-2.583396	3.484694
H	-5.132836	-0.988642	3.881196
C	-0.979250	-1.347653	-3.150550
H	-0.778397	-0.768085	-4.054669
H	-0.241744	-1.074315	-2.397113
H	-0.839857	-2.403146	-3.395308
C	-3.438392	-1.490277	-3.694634
H	-3.345972	-2.554525	-3.925719
H	-4.457270	-1.318310	-3.344245
H	-3.298003	-0.931107	-4.623435

B	0.629646	0.815904	0.365034
B	-0.923254	1.151174	0.233288
C	1.782514	1.137791	-0.561730
C	1.676613	2.169091	-1.689353
C	3.141285	2.324092	-2.162063
C	3.897518	1.071439	-1.706135
H	3.583225	3.200747	-1.684995
H	3.210540	2.467603	-3.240291
N	3.021199	0.622000	-0.563851
C	3.467303	-0.330134	0.418070
C	4.087526	0.157771	1.586359
C	3.213397	-1.703851	0.240297
C	4.521023	-0.766343	2.534977
C	3.674527	-2.583710	1.220435
C	4.333055	-2.127136	2.349913
H	4.993239	-0.415055	3.443219
H	3.489369	-3.644122	1.108085
H	4.675448	-2.829686	3.099861
C	0.755877	1.674713	-2.817947
H	0.722112	2.422921	-3.614529
H	1.106717	0.739223	-3.249072
H	-0.253180	1.518684	-2.438125
C	1.146009	3.514364	-1.171656
H	1.710940	3.852829	-0.300784
H	1.237673	4.269553	-1.957665
H	0.099755	3.429191	-0.888999
C	3.963163	-0.002508	-2.799902
H	4.546130	0.378767	-3.639911
H	4.454607	-0.900626	-2.430418
H	2.976423	-0.274042	-3.169042
C	5.325133	1.384814	-1.260288
H	5.803571	0.512502	-0.813208
H	5.909346	1.678936	-2.133773
H	5.358132	2.205650	-0.547885
C	2.390449	-2.271811	-0.908077
H	2.128797	-1.454993	-1.575977
C	4.224407	1.642616	1.896991
H	3.931114	2.203620	1.011336
C	3.265354	2.064589	3.023711
H	3.469893	1.511236	3.942942
H	3.386058	3.129510	3.236473
H	2.226408	1.892535	2.745622
C	5.666883	2.035216	2.251408
H	5.974178	1.600570	3.204710
H	6.375288	1.703149	1.491370
H	5.747974	3.120456	2.345304
C	1.068394	-2.878503	-0.406969
H	1.246104	-3.680593	0.311726
H	0.430598	-2.132433	0.062929
H	0.510694	-3.297245	-1.245197
C	3.169420	-3.321854	-1.715942
H	3.339620	-4.224332	-1.125131
H	2.600101	-3.610835	-2.602278
H	4.142117	-2.952396	-2.041652
N	0.933725	-0.648480	2.462243
N	0.871228	-0.027230	1.533852

Int$_1$'

108

C	-3.907152	-1.134981	-2.533096
C	-4.234873	0.334353	-2.207695
C	-2.051096	-0.075961	-1.381691
C	-2.402190	-1.345175	-2.204162
H	-4.132629	-1.385560	-3.571169
H	-4.519294	-1.782612	-1.901771
N	-3.275024	0.579925	-1.106653
C	-3.952021	1.238136	-3.425247
H	-4.535318	0.908726	-4.288592
H	-4.222521	2.271171	-3.211430
H	-2.895796	1.211527	-3.692907
C	-1.570750	-1.456466	-3.491065
H	-0.506394	-1.526568	-3.270612
H	-1.858121	-2.349414	-4.054690
H	-1.714661	-0.586718	-4.131132
C	-2.211065	-2.620768	-1.366065
H	-2.567216	-3.501694	-1.911889
H	-1.159108	-2.765608	-1.119618
H	-2.761901	-2.553020	-0.427836
C	-5.689525	0.533373	-1.786669
H	-5.869457	1.562862	-1.471535
H	-6.342240	0.322390	-2.635980
H	-5.973980	-0.127585	-0.970275
C	-3.519296	1.458577	-0.015555

C	-3.152496	2.818639	-0.053814
C	-4.155258	0.932888	1.135039
C	-3.482077	3.638739	1.028350
C	-4.453163	1.785835	2.194727
C	-4.134066	3.135321	2.140456
H	-3.212400	4.687443	1.001765
H	-4.935459	1.392461	3.080742
H	-4.380324	3.787254	2.970368
C	-2.370232	3.421828	-1.209064
H	-2.165421	2.625773	-1.922606
C	-4.453043	-0.552418	1.265105
H	-4.425573	-0.974708	0.263938
C	-1.015413	3.972714	-0.734472
H	-0.441637	4.344481	-1.585241
H	-1.148280	4.795565	-0.028119
H	-0.423345	3.196540	-0.251115
C	-3.165375	4.523822	-1.925474
H	-2.627275	4.867920	-2.812212
H	-4.149196	4.172214	-2.239300
H	-3.317023	5.385542	-1.271056
C	-3.340665	-1.245051	2.063018
H	-2.374170	-1.089409	1.587285
H	-3.290257	-0.851961	3.082299
H	-3.521992	-2.321847	2.123713
C	-5.831495	-0.854161	1.863137
H	-6.625028	-0.329908	1.326671
H	-6.034438	-1.926477	1.809485
H	-5.892736	-0.564959	2.914833
B	0.771617	0.785717	-1.420310
B	-0.712120	0.260894	-1.080367
C	2.151437	0.847608	-0.759374
C	3.395514	1.600534	-1.274768
C	4.512751	1.088324	-0.340492
C	3.839111	0.602653	0.940927
H	5.265350	1.851831	-0.147402
H	5.014980	0.244340	-0.817649
N	2.458257	0.276693	0.409670
C	1.627829	-0.716105	1.052212
C	0.795196	-0.367912	2.127724
C	1.707770	-2.042267	0.578815
C	0.059798	-1.382590	2.740081
C	0.925229	-3.008159	1.207773
C	0.114348	-2.688196	2.284886
H	-0.595105	-1.135768	3.564511
H	0.952005	-4.028934	0.849391
H	-0.484403	-3.454880	2.760997
C	3.802031	1.258163	-2.721468
H	3.737555	0.184963	-2.909341
H	3.205072	1.772804	-3.468328
H	4.841430	1.564493	-2.863987
C	3.185787	3.122935	-1.156559
H	2.388148	3.460199	-1.816687
H	2.926408	3.425044	-0.143300
H	4.106341	3.637953	-1.441723
C	4.533099	-0.619636	1.538954
H	5.519502	-0.317908	1.894519
H	3.975987	-1.021216	2.385916
H	4.670583	-1.409307	0.805640
C	3.802408	1.701702	2.012507
H	3.370129	1.337565	2.940020
H	4.827146	2.011592	2.224404
H	3.245751	2.578193	1.688985
C	2.589599	-2.466915	-0.589682
H	3.249815	-1.637961	-0.839229
C	0.610429	1.057868	2.618977
H	1.261500	1.703073	2.034276
C	-0.823386	1.544955	2.384530
H	-0.913365	2.603484	2.634669
H	-1.118450	1.415292	1.346407
H	-1.537275	0.995313	2.998043
C	0.976930	1.208916	4.103920
H	1.964745	0.805632	4.331799
H	0.962712	2.262713	4.390711
H	0.257127	0.684387	4.735845
C	1.756586	-2.763456	-1.843921
H	1.065348	-3.590014	-1.667768
H	1.179106	-1.889302	-2.135089
H	2.408421	-3.038871	-2.676602
C	3.475524	-3.674483	-0.242732
H	4.188994	-3.856848	-1.049380
H	4.033863	-3.518561	0.681411
H	2.881370	-4.582136	-0.120791

N	0.687110	1.974812	-2.675023
N	0.934618	0.943913	-3.141349

Int$_2$'

108

C	-4.433382	-1.226818	-2.608096
C	-4.724348	-0.950997	-1.119647
C	-2.515351	-0.177070	-1.644281
C	-3.172909	-0.414440	-2.989686
H	-5.289979	-0.980363	-3.236091
H	-4.227371	-2.291051	-2.739486
N	-3.384556	-0.476751	-0.645346
C	-5.798326	0.128035	-0.920269
H	-6.756337	-0.241225	-1.290417
H	-5.911844	0.360678	0.138389
H	-5.562750	1.047072	-1.452382
C	-3.524530	0.937220	-3.646239
H	-2.616252	1.511916	-3.832514
H	-4.035850	0.771362	-4.599001
H	-4.175476	1.538126	-3.011802
C	-2.267058	-1.206014	-3.943718
H	-2.795026	-1.420165	-4.877363
H	-1.365538	-0.638820	-4.180153
H	-1.960751	-2.152446	-3.494274
C	-5.182497	-2.213649	-0.386695
H	-5.274573	-2.033288	0.685388
H	-6.162218	-2.511681	-0.764251
H	-4.494813	-3.041881	-0.544561
C	-3.082163	-0.211392	0.733916
C	-3.246484	1.087936	1.256814
C	-2.565626	-1.251725	1.528990
C	-2.992436	1.286481	2.613851
C	-2.325575	-0.998982	2.879670
C	-2.556215	0.252378	3.426238
H	-3.113058	2.276185	3.035409
H	-1.926612	-1.787388	3.505155
H	-2.361838	0.431993	4.476862
C	-3.596783	2.296096	0.401663
H	-3.787787	1.949898	-0.610889
C	-2.150060	-2.593643	0.949346
H	-2.466592	-2.623377	-0.091694
C	-2.402632	3.263658	0.322200
H	-2.631253	4.081263	-0.366058
H	-2.188841	3.698827	1.301758
H	-1.506053	2.750597	-0.022864
C	-4.847859	3.035588	0.897848
H	-5.101949	3.845152	0.209357
H	-5.710736	2.373782	0.975996
H	-4.680190	3.481112	1.880934
C	-0.619472	-2.707919	0.956677
H	-0.157578	-1.871705	0.434145
H	-0.230913	-2.713278	1.975553
H	-0.304933	-3.635400	0.472270
C	-2.780388	-3.787351	1.679670
H	-3.867751	-3.716216	1.713850
H	-2.514812	-4.718949	1.173997
H	-2.418318	-3.858797	2.707619
B	1.008042	0.836233	-1.296077
B	-1.120382	0.298808	-1.468151
C	2.471750	1.057317	-1.300025
C	3.240060	1.660544	-2.465678
C	4.715826	1.439434	-2.049656
C	4.725865	1.211617	-0.524977
H	5.356165	2.273939	-2.337022
H	5.101246	0.545423	-2.543744
N	3.332786	0.710420	-0.304802
C	2.965640	-0.190775	0.748695
C	2.529016	0.293161	1.992860
C	3.038772	-1.577670	0.489293
C	2.261820	-0.633819	3.003560
C	2.758942	-2.458732	1.530174
C	2.393359	-1.993561	2.785358
H	1.928421	-0.280356	3.971059
H	2.806889	-3.525494	1.354431
H	2.180557	-2.695386	3.582852
C	2.922282	0.925157	-3.779151
H	3.112370	-0.145397	-3.683743
H	1.875473	1.059311	-4.053705
H	3.545541	1.315194	-4.589434
C	2.893352	3.152087	-2.640367
H	1.831654	3.265042	-2.863470
H	3.107942	3.725187	-1.738531
H	3.472078	3.581082	-3.463495

C	5.786426	0.199335	-0.093550
H	6.775928	0.619043	-0.282411
H	5.710900	-0.023204	0.971818
H	5.706156	-0.733376	-0.647213
C	4.962387	2.522853	0.242144
H	4.970864	2.348420	1.316385
H	5.931572	2.941076	-0.038176
H	4.194602	3.262030	0.019583
C	3.348318	-2.132746	-0.894057
H	3.749255	-1.321636	-1.498898
C	2.275192	1.766070	2.264663
H	2.535574	2.320309	1.364676
C	0.783444	2.023454	2.537353
H	0.609951	3.093662	2.678409
H	0.168223	1.680947	1.706138
H	0.451421	1.509381	3.442364
C	3.131803	2.295220	3.424738
H	4.193202	2.088352	3.277324
H	3.005596	3.375707	3.526459
H	2.835997	1.836583	4.371115
C	2.063239	-2.613454	-1.589019
H	1.619459	-3.448437	-1.043559
H	1.322030	-1.815488	-1.649659
H	2.287255	-2.949533	-2.604638
C	4.395679	-3.254488	-0.873092
H	4.673779	-3.524335	-1.894520
H	5.299600	-2.953397	-0.340463
H	4.010705	-4.155435	-0.391321
N	-0.151819	0.695710	-0.452971
N	-0.116826	1.021266	-2.209944

TS_2

110

C	-3.725326	2.673712	1.168782
C	-4.445728	1.994605	-0.004180
C	-2.194166	1.132818	0.104135
C	-2.212618	2.406167	0.991222
H	-4.064753	2.216214	2.100930
H	-3.947634	3.740172	1.230980
N	-3.508214	0.876195	-0.298876
C	-5.834399	1.491925	0.390105
H	-5.792410	0.845172	1.264104
H	-6.474093	2.344025	0.627856
H	-6.301175	0.936863	-0.425444
C	-1.572687	2.158902	2.367218
H	-1.745780	3.017683	3.023305
H	-1.993252	1.271714	2.842610
H	-0.497248	2.015276	2.277120
C	-1.488489	3.592709	0.334135
H	-0.424011	3.376792	0.247291
H	-1.865356	3.802405	-0.666321
H	-1.608852	4.494323	0.942187
C	-4.602798	2.969733	-1.188407
H	-5.186610	2.526369	-1.992229
H	-5.125399	3.870076	-0.857070
H	-3.636253	3.267607	-1.591491
C	-3.931300	-0.410959	-0.737324
C	-4.136355	-1.433117	0.220427
C	-4.101287	-0.681676	-2.110151
C	-4.525370	-2.695582	-0.220467
C	-4.512338	-1.957101	-2.500163
C	-4.721006	-2.960193	-1.568705
H	-4.676806	-3.488609	0.501299
H	-4.649533	-2.173660	-3.552436
H	-5.028319	-3.948014	-1.890499
C	-3.910272	-1.216857	1.709151
H	-3.734740	-0.154303	1.864991
C	-3.803395	0.348598	-3.186195
H	-3.442851	1.243852	-2.685849
C	-2.656115	-1.960156	2.195439
H	-1.776456	-1.634929	1.642947
H	-2.480927	-1.759563	3.255209
H	-2.767067	-3.040058	2.069882
C	-5.129305	-1.620066	2.552505
H	-6.041628	-1.132897	2.204272
H	-5.298251	-2.698633	2.522270
H	-4.970697	-1.341557	3.597071
C	-2.685735	-0.114607	-4.133842
H	-2.459952	0.671025	-4.858784
H	-1.771801	-0.337830	-3.585463
H	-2.973381	-1.009760	-4.690086
C	-5.062557	0.705938	-3.990514
H	-5.400277	-0.146689	-4.584585

H	-5.887201	0.998476	-3.338961
H	-4.858794	1.531234	-4.677030
B	0.658463	0.298256	0.251831
B	-0.933120	0.480204	-0.121000
C	1.904771	0.541005	-0.552117
C	1.891272	1.135810	-1.964547
C	3.378515	1.457690	-2.225204
C	4.205999	0.562568	-1.295809
H	3.566912	2.503414	-1.974121
H	3.652118	1.318899	-3.271246
N	3.188052	0.241030	-0.236510
C	3.551662	-0.400447	0.996100
C	3.890099	0.399301	2.106718
C	3.498503	-1.806005	1.099295
C	4.254646	-0.237236	3.292139
C	3.873763	-2.389489	2.309517
C	4.262749	-1.619074	3.393135
H	4.515158	0.358868	4.157352
H	3.838574	-3.466681	2.412198
H	4.546382	-2.094544	4.324158
C	1.349296	0.092040	-2.960487
H	1.465017	0.458835	-3.983595
H	1.865779	-0.864340	-2.880381
H	0.291468	-0.081667	-2.777500
C	1.050913	2.412352	-2.082942
H	1.374561	3.159969	-1.357009
H	1.169439	2.834544	-3.085280
H	-0.003763	2.205320	-1.910157
C	4.714703	-0.703980	-1.997798
H	5.421439	-0.420231	-2.779094
H	5.236604	-1.349010	-1.292438
H	3.912327	-1.273510	-2.460626
C	5.420904	1.301271	-0.730767
H	5.937382	0.701231	0.019504
H	6.122019	1.501493	-1.542654
H	5.142940	2.254533	-0.287797
C	2.971133	-2.711964	-0.004896
H	2.750042	-2.094337	-0.871544
C	3.790076	1.918778	2.099038
H	3.555860	2.236436	1.084896
C	2.633208	2.399906	2.990598
H	2.789186	2.110140	4.032027
H	2.557469	3.489209	2.950617
H	1.683338	1.983958	2.660968
C	5.101006	2.596349	2.526558
H	5.320044	2.402913	3.578628
H	5.950470	2.245546	1.940262
H	5.022555	3.678811	2.401487
C	1.651749	-3.387123	0.408109
H	1.794358	-4.035955	1.274663
H	0.888811	-2.652897	0.655105
H	1.274749	-4.000337	-0.413427
C	3.990641	-3.783511	-0.422204
H	4.158540	-4.500606	0.384051
H	3.618509	-4.339772	-1.285597
H	4.955495	-3.351430	-0.686940
N	0.721925	-0.668462	2.630090
N	0.777727	-0.268108	1.582311
N	-1.292810	-1.305159	-1.062667
N	-1.127925	-2.333443	-1.413690

Int_2

110

C	-3.470005	2.925940	0.926505
C	-4.307249	2.187120	-0.121824
C	-2.140258	1.129761	-0.003055
C	-2.004627	2.464280	0.745538
H	-3.815398	2.638866	1.921710
H	-3.573571	4.008379	0.845159
N	-3.451422	0.978751	-0.355660
C	-5.697670	1.818455	0.394490
H	-5.648647	1.284032	1.340216
H	-6.272157	2.732780	0.553350
H	-6.237601	1.200904	-0.324936
C	-1.373009	2.272571	2.133850
H	-1.485151	3.191779	2.715909
H	-1.856646	1.459995	2.678596
H	-0.314187	2.050197	2.059979
C	-1.157606	3.477086	-0.041471
H	-0.137730	3.107023	-0.139213
H	-1.549281	3.652537	-1.043582
H	-1.132328	4.432990	0.488818
C	-4.474041	3.034130	-1.394190

H	-5.077106	2.518612	-2.138008
H	-4.985233	3.964952	-1.139369
H	-3.513453	3.288035	-1.839775
C	-3.979238	-0.273755	-0.810374
C	-4.330078	-1.240029	0.157759
C	-4.077935	-0.557062	-2.185034
C	-4.816141	-2.469644	-0.281532
C	-4.586003	-1.798315	-2.570632
C	-4.955651	-2.747451	-1.632650
H	-5.077437	-3.228558	0.444930
H	-4.666946	-2.034752	-3.624104
H	-5.334518	-3.710476	-1.953307
C	-4.148146	-1.020807	1.653792
H	-3.878252	0.022168	1.811408
C	-3.583274	0.391047	-3.265232
H	-3.194390	1.279172	-2.773662
C	-2.993117	-1.872421	2.207566
H	-2.054788	-1.630703	1.710412
H	-2.867720	-1.685292	3.276913
H	-3.191850	-2.937961	2.072121
C	-5.438098	-1.296490	2.442464
H	-6.285943	-0.740155	2.039519
H	-5.698705	-2.356695	2.422127
H	-5.306262	-1.011085	3.488610
C	-2.419904	-0.221688	-4.059845
H	-2.028049	0.507222	-4.773373
H	-1.608490	-0.518473	-3.400485
H	-2.738452	-1.102613	-4.621533
C	-4.708178	0.812450	-4.223411
H	-5.042639	-0.034656	-4.826253
H	-5.577596	1.197995	-3.689207
H	-4.355415	1.588399	-4.907021
B	0.680158	0.324166	0.104968
B	-0.994180	0.199712	-0.243245
C	1.848657	0.524013	-0.808836
C	1.718810	0.792205	-2.314635
C	3.130493	1.273076	-2.707713
C	4.107813	0.694968	-1.676919
H	3.163284	2.363408	-2.656774
H	3.395259	0.984233	-3.725214
N	3.181899	0.434794	-0.524213

C	3.675294	0.017185	0.756654
C	4.001418	0.990079	1.724067
C	3.771725	-1.359721	1.052972
C	4.508474	0.561710	2.950562
C	4.280678	-1.731922	2.296901
C	4.663794	-0.785444	3.233405
H	4.763336	1.294836	3.705499
H	4.360078	-2.782900	2.544320
H	5.057172	-1.098739	4.193050
C	1.360473	-0.522802	-3.037016
H	1.350004	-0.362124	-4.118496
H	2.073426	-1.316931	-2.821055
H	0.378688	-0.875233	-2.735859
C	0.681953	1.858621	-2.681095
H	0.899500	2.802707	-2.180520
H	0.702255	2.031799	-3.760882
H	-0.322865	1.550881	-2.397436
C	4.799912	-0.587278	-2.162010
H	5.443076	-0.349914	-3.011008
H	5.426845	-1.001355	-1.372729
H	4.094659	-1.350499	-2.478580
C	5.207091	1.700452	-1.325236
H	5.836975	1.329749	-0.515555
H	5.841394	1.854965	-2.199993
H	4.794309	2.664475	-1.036675
C	3.274745	-2.456447	0.119713
H	2.952894	-1.988205	-0.805964
C	3.735320	2.476528	1.530544
H	3.376266	2.621443	0.513299
C	2.617388	2.958194	2.469740
H	2.913795	2.868445	3.517154
H	2.386735	4.007867	2.272448
H	1.708111	2.379676	2.327519
C	4.992504	3.336314	1.733522
H	5.325232	3.307142	2.773409
H	5.821910	3.002311	1.110817
H	4.778843	4.379130	1.486756
C	2.045885	-3.176992	0.701055
H	2.284910	-3.664285	1.649074
H	1.222300	-2.486870	0.869511
H	1.702549	-3.945637	0.004635

C	4.367215	-3.484625	-0.215592
H	4.645484	-4.065929	0.666051
H	4.001898	-4.185931	-0.969597
H	5.270342	-3.012089	-0.601135
N	0.963362	-0.304268	2.567962
N	0.925934	-0.032414	1.475650
N	-1.267648	-1.111431	-0.766792
N	-1.337397	-2.175235	-1.131851

Int$_3$

110

C	-5.052984	2.374544	-1.150591
C	-5.350971	0.907215	-0.803702
C	-2.979880	1.279789	-0.593989
C	-3.518943	2.509394	-1.319932
H	-5.383340	3.013307	-0.329356
H	-5.592356	2.692821	-2.043573
N	-4.012499	0.457002	-0.292122
C	-6.443572	0.769680	0.257588
H	-6.541405	-0.264249	0.591825
H	-6.246443	1.396829	1.123468
H	-7.399331	1.077927	-0.169387
C	-3.033615	3.818574	-0.673271
H	-1.957286	3.933787	-0.787344
H	-3.535170	4.669823	-1.143026
H	-3.268816	3.831864	0.393616
C	-3.091892	2.483040	-2.800995
H	-3.548074	3.321968	-3.333677
H	-2.007709	2.564365	-2.879041
H	-3.398707	1.559550	-3.294495
C	-5.781645	0.106913	-2.041173
H	-5.926606	-0.944638	-1.797157
H	-6.730110	0.498791	-2.412189
H	-5.051300	0.180382	-2.844956
C	-3.828651	-0.740248	0.476793
C	-3.875360	-0.647248	1.883303
C	-3.541415	-1.963060	-0.161489
C	-3.714639	-1.813667	2.628711
C	-3.391062	-3.100732	0.632866
C	-3.488339	-3.034536	2.012928
H	-3.749556	-1.761252	3.709210
H	-3.172850	-4.050509	0.162177
H	-3.368898	-3.929855	2.610537
C	-4.013497	0.676332	2.621938
H	-4.204014	1.457015	1.887757
C	-3.311474	-2.089718	-1.659202
H	-3.554216	-1.136319	-2.117813
C	-2.697635	1.041102	3.329865
H	-2.799682	2.001142	3.841513
H	-2.429904	0.287066	4.073104
H	-1.878191	1.123307	2.617124
C	-5.176800	0.672900	3.624643
H	-5.315109	1.673049	4.042376
H	-6.112958	0.366209	3.156740
H	-4.981318	-0.006413	4.456855
C	-1.833483	-2.363055	-1.975929
H	-1.199798	-1.544367	-1.645105
H	-1.483931	-3.279159	-1.494156
H	-1.700284	-2.473982	-3.054600
C	-4.191082	-3.173541	-2.299701
H	-5.244086	-3.053277	-2.041453
H	-4.096818	-3.137417	-3.387450
H	-3.881796	-4.170656	-1.978931
B	2.002177	1.421388	-0.458481
B	-1.528817	1.062105	-0.313396
C	3.445406	1.282487	-0.504635
C	4.373375	2.332000	-1.108681
C	5.766815	1.863915	-0.623679
C	5.659006	0.364478	-0.282961
H	6.037351	2.415374	0.278991
H	6.545041	2.049499	-1.364330
N	4.187175	0.222357	-0.067851
C	3.592918	-0.953851	0.493563
C	3.416695	-1.030879	1.888686
C	3.174008	-2.002406	-0.348662
C	2.945558	-2.225698	2.430206
C	2.705422	-3.176741	0.241678
C	2.616664	-3.300265	1.619132
H	2.814302	-2.307340	3.501665
H	2.388170	-3.999554	-0.386754
H	2.257054	-4.221636	2.060828
C	4.259175	2.311796	-2.646019
H	4.981450	3.004449	-3.086701

H	4.449324	1.318405	-3.053153
H	3.256539	2.611737	-2.954701
C	4.052688	3.745709	-0.603714
H	3.050781	4.050538	-0.912506
H	4.096681	3.787033	0.486391
H	4.769785	4.466307	-1.007847
C	6.159883	-0.531307	-1.426818
H	7.231696	-0.377524	-1.567122
H	6.002734	-1.583427	-1.189893
H	5.661489	-0.311162	-2.368663
C	6.455154	0.010404	0.975190
H	6.274107	-1.022587	1.276997
H	7.522063	0.119640	0.772177
H	6.198625	0.666714	1.803649
C	3.106450	-1.858753	-1.860852
H	3.591201	-0.923439	-2.130332
C	3.613287	0.170444	2.799813
H	4.074519	0.964318	2.213872
C	2.245320	0.697277	3.267876
H	1.724304	-0.051921	3.868713
H	2.377991	1.592883	3.879828
H	1.611058	0.949568	2.417360
C	4.518629	-0.122168	4.003376
H	5.483029	-0.526083	3.692968
H	4.697903	0.794675	4.570230
H	4.058458	-0.841835	4.684167
C	1.639064	-1.746345	-2.305804
H	1.085894	-2.657767	-2.069242
H	1.148284	-0.910621	-1.808111
H	1.581088	-1.585029	-3.385073
C	3.810476	-2.995387	-2.613279
H	3.310639	-3.952754	-2.449820
H	3.796383	-2.797638	-3.688022
H	4.849769	-3.103051	-2.300877
N	-0.451197	1.948164	-0.740295
N	0.760071	1.693440	-0.589863
N	-1.033671	-0.081561	0.429229
N	-0.329939	-0.786171	0.952423

TS_3

110

C	-4.843092	2.739326	-1.325564
C	-5.080724	1.328209	-0.760280
C	-2.709253	1.707968	-0.959730
C	-3.363392	2.819011	-1.770162
H	-5.018136	3.472982	-0.536424
H	-5.529874	2.967916	-2.141033
N	-3.681390	0.914956	-0.423783
C	-5.984217	1.355351	0.474743
H	-6.046622	0.369629	0.938233
H	-5.629954	2.067590	1.216394
H	-6.991262	1.653015	0.176950
C	-2.786367	4.206609	-1.452881
H	-1.747435	4.283955	-1.770593
H	-3.365887	4.975799	-1.971253
H	-2.830182	4.407828	-0.380734
C	-3.191606	2.537090	-3.276627
H	-3.720226	3.293140	-3.863953
H	-2.135131	2.563663	-3.544820
H	-3.582744	1.557494	-3.552841
C	-5.725623	0.392626	-1.793495
H	-5.815686	-0.616796	-1.394338
H	-6.729209	0.751944	-2.028802
H	-5.158469	0.350937	-2.720633
C	-3.355783	-0.236103	0.367519
C	-3.186865	-0.083934	1.759266
C	-3.110929	-1.475974	-0.257925
C	-2.856411	-1.208197	2.514960
C	-2.784790	-2.569168	0.544957
C	-2.668592	-2.444884	1.919613
H	-2.722700	-1.108782	3.584765
H	-2.595979	-3.529532	0.081888
H	-2.408058	-3.305635	2.523609
C	-3.271987	1.263811	2.461208
H	-3.577590	2.006427	1.726608
C	-3.105671	-1.660890	-1.768436
H	-3.405830	-0.720611	-2.222175
C	-1.894082	1.698198	2.985656
H	-1.961250	2.682360	3.456042
H	-1.514270	0.994827	3.730119
H	-1.167698	1.756608	2.177400
C	-4.299800	1.264840	3.602899
H	-4.407744	2.272170	4.012210

H	-5.280106	0.926381	3.266123
H	-3.984043	0.610526	4.418319
C	-1.691033	-1.970787	-2.284710
H	-0.993638	-1.178282	-2.021572
H	-1.311919	-2.905704	-1.867440
H	-1.701725	-2.067168	-3.373249
C	-4.083506	-2.752457	-2.229072
H	-5.091439	-2.585266	-1.847738
H	-4.132659	-2.778684	-3.320086
H	-3.761008	-3.739151	-1.889520
B	1.965709	1.340058	-0.679609
B	-1.252936	1.524025	-0.826665
C	3.398156	1.176264	-0.501190
C	4.423929	2.044864	-1.229467
C	5.718302	1.749784	-0.434790
C	5.548418	0.372788	0.233354
H	5.837702	2.508528	0.341031
H	6.605927	1.786057	-1.067564
N	4.060286	0.251973	0.265348
C	3.350487	-0.732099	1.028053
C	2.926473	-0.414139	2.333569
C	2.995136	-1.957506	0.432453
C	2.216014	-1.373793	3.053487
C	2.284942	-2.884656	1.195083
C	1.907172	-2.605214	2.498181
H	1.877570	-1.143875	4.056015
H	1.999144	-3.830070	0.751335
H	1.348848	-3.335703	3.071267
C	4.539406	1.607345	-2.703651
H	5.347443	2.152819	-3.199518
H	4.741723	0.540555	-2.795720
H	3.606643	1.813636	-3.230128
C	4.084330	3.539451	-1.182153
H	3.160188	3.747549	-1.722599
H	3.957126	3.876959	-0.152021
H	4.887845	4.122137	-1.641218
C	6.206257	-0.754926	-0.578328
H	7.288225	-0.609955	-0.590488
H	6.002976	-1.723527	-0.122006
H	5.859973	-0.778832	-1.608981
C	6.159127	0.346693	1.638181
H	5.921410	-0.585814	2.152956
H	7.245121	0.421239	1.560255
H	5.808473	1.177662	2.245741
C	3.265196	-2.271208	-1.031165
H	3.866683	-1.464525	-1.439768
C	3.132346	0.960260	2.952951
H	3.750779	1.543597	2.274154
C	1.795852	1.707057	3.085335
H	1.104699	1.169903	3.737695
H	1.958399	2.701348	3.509471
H	1.319284	1.823844	2.113684
C	3.844887	0.890702	4.311684
H	4.782773	0.337651	4.247639
H	4.065394	1.897737	4.673982
H	3.220386	0.400832	5.061972
C	1.955427	-2.296187	-1.835064
H	1.279123	-3.071201	-1.468651
H	1.439474	-1.340490	-1.764721
H	2.162918	-2.498417	-2.889087
C	4.036087	-3.585129	-1.220873
H	3.434533	-4.445424	-0.918839
H	4.298415	-3.720399	-2.272964
H	4.956519	-3.602154	-0.636103
N	-0.152412	2.310092	-1.449424
N	1.028190	2.110659	-1.281460
N	-0.535389	0.504857	-0.071871
N	0.555026	0.196209	0.155429

Pdt

110

C	-5.086631	-1.149605	-2.370232
C	-5.151980	-0.720903	-0.895927
C	-2.869536	-0.632829	-1.624883
C	-3.688408	-0.759035	-2.899836
H	-5.889042	-0.699095	-2.955298
H	-5.205441	-2.233136	-2.433709
N	-3.686063	-0.629395	-0.556701
C	-5.851488	0.630908	-0.702690
H	-6.906840	0.527422	-0.959243
H	-5.786792	0.951389	0.336866
H	-5.427457	1.409652	-1.331996
C	-3.700497	0.593176	-3.643485

H	-2.686056	0.872563	-3.926268
H	-4.307752	0.509950	-4.548751
H	-4.118732	1.391259	-3.029854
C	-3.141227	-1.840419	-3.843194
H	-3.830115	-1.984271	-4.680485
H	-2.166509	-1.552665	-4.233377
H	-3.031750	-2.793802	-3.321772
C	-5.879294	-1.754712	-0.037396
H	-5.852398	-1.480197	1.017320
H	-6.924692	-1.800092	-0.347803
H	-5.450499	-2.747692	-0.152853
C	-3.242754	-0.317911	0.781867
C	-3.012982	1.028532	1.136349
C	-3.033430	-1.360730	1.702862
C	-2.676055	1.307527	2.459464
C	-2.698381	-1.021762	3.013497
C	-2.542198	0.298699	3.398396
H	-2.492226	2.332574	2.753529
H	-2.531064	-1.808999	3.737148
H	-2.280704	0.540203	4.421361
C	-3.022660	2.176746	0.134512
H	-3.376495	1.795512	-0.819119
C	-3.043774	-2.831826	1.312553
H	-3.367011	-2.899704	0.274480
C	-1.598063	2.705813	-0.106389
H	-1.611255	3.473248	-0.884162
H	-1.186634	3.150677	0.801236
H	-0.921819	1.914934	-0.425913
C	-3.947070	3.328932	0.553369
H	-3.990712	4.081626	-0.237608
H	-4.962915	2.987119	0.751184
H	-3.581974	3.824633	1.455041
C	-1.619342	-3.411014	1.375495
H	-0.937135	-2.822542	0.762615
H	-1.243529	-3.408588	2.401867
H	-1.621691	-4.445747	1.022987
C	-3.995767	-3.675212	2.173742
H	-5.010460	-3.277893	2.182156
H	-4.033377	-4.699769	1.796298
H	-3.650618	-3.723533	3.208603
B	1.326599	0.347944	-1.472200
B	-1.371862	-0.493877	-1.588820
C	2.683692	0.993545	-1.317448
C	3.247274	2.024688	-2.284156
C	4.732091	2.121582	-1.871022
C	4.847638	1.562887	-0.444673
H	5.110251	3.142197	-1.934591
H	5.332703	1.510867	-2.546962
N	3.568105	0.773169	-0.329022
C	3.393611	-0.214279	0.709974
C	2.890760	0.160003	1.968229
C	3.727647	-1.554496	0.426871
C	2.825347	-0.813125	2.965884
C	3.626339	-2.487991	1.455897
C	3.200646	-2.122387	2.722531
H	2.441790	-0.544269	3.941803
H	3.872175	-3.523415	1.257890
H	3.135250	-2.863437	3.509772
C	3.137620	1.596438	-3.754745
H	3.609728	0.624621	-3.912365
H	2.101522	1.515300	-4.075109
H	3.650421	2.331728	-4.380850
C	2.496855	3.357849	-2.087177
H	1.445341	3.241255	-2.350001
H	2.548224	3.703056	-1.053513
H	2.932405	4.129427	-2.727912
C	6.076869	0.667384	-0.282649
H	6.975670	1.277072	-0.390127
H	6.101021	0.194332	0.699275
H	6.106404	-0.109269	-1.043459
C	4.922740	2.680940	0.600571
H	4.972162	2.270024	1.605854
H	5.829345	3.264742	0.431790
H	4.070560	3.355257	0.540064
C	4.108973	-2.047375	-0.962233
H	4.230521	-1.182142	-1.612638
C	2.301267	1.530711	2.264792
H	2.455692	2.161424	1.391882
C	0.783392	1.421528	2.479027
H	0.349843	2.415397	2.611712
H	0.296992	0.939249	1.636269
H	0.553354	0.837151	3.371976

C	2.944784	2.214270	3.480935
H	4.030823	2.264272	3.406798
H	2.564328	3.233285	3.585119
H	2.703734	1.680407	4.402489
C	2.979904	-2.904699	-1.560777
H	2.829562	-3.812170	-0.971319
H	2.038660	-2.358836	-1.586656
H	3.233702	-3.203315	-2.580754
C	5.429908	-2.832535	-0.970385
H	5.720216	-3.067864	-1.997030
H	6.240930	-2.272025	-0.504141
H	5.333203	-3.778824	-0.434501
N	-0.636150	0.013088	-2.717007
N	0.586003	0.258055	-2.752242
N	0.645956	-0.344217	-0.417335
N	-0.478258	-0.876195	-0.467633

LiCl

Li	-0.138476	3.393090	-0.142420
Cl	-0.972559	5.005074	-1.024511

RC_{LiCl}

110

C	3.996687	-4.682044	1.879115
C	4.291247	-4.198695	0.447891
C	2.028328	-3.648684	0.949788
C	2.459352	-4.613125	2.063639
H	4.481461	-4.011079	2.590808
H	4.386560	-5.684498	2.056041
N	3.111086	-3.297348	0.223723
C	5.629592	-3.476579	0.309625
H	5.766724	-2.701831	1.059531
H	6.435819	-4.202170	0.427087
H	5.726602	-3.022698	-0.677936
C	2.092469	-4.024632	3.436646
H	2.474498	-4.668948	4.233017
H	2.520480	-3.029551	3.566634
H	1.009531	-3.945783	3.544602
C	1.781129	-5.984948	1.919362
H	1.990240	-6.446633	0.954745
H	2.132988	-6.658807	2.704545
H	0.700831	-5.874362	2.007869
C	4.274186	-5.370839	-0.549151
H	4.504077	-5.032108	-1.557452
H	5.031900	-6.101652	-0.260534
H	3.307167	-5.870191	-0.567465
C	3.088468	-2.128129	-0.616725
C	3.439623	-0.897014	-0.019960
C	2.651138	-2.195190	-1.949730
C	3.320833	0.261335	-0.782408
C	2.573433	-1.004483	-2.675416
C	2.892546	0.212358	-2.099552
H	3.531844	1.220688	-0.331514
H	2.234282	-1.031853	-3.702811
H	2.789195	1.127806	-2.668168
C	3.895965	-0.783866	1.429739
H	4.136276	-1.781682	1.787071
C	2.270749	-3.496419	-2.635141
H	2.271370	-4.283585	-1.883049
C	2.776546	-0.259856	2.336546
H	1.946586	-0.968098	2.344183
H	3.132673	-0.158689	3.365047
H	2.400512	0.705181	1.995847
C	5.163118	0.070470	1.575906
H	5.532389	0.018681	2.602894
H	5.955453	-0.273321	0.908137
H	4.968157	1.120236	1.350326
C	0.857086	-3.450434	-3.229958
H	0.618728	-4.411029	-3.692444
H	0.121182	-3.249975	-2.452959
H	0.767893	-2.681252	-3.999803
C	3.294869	-3.857619	-3.723778
H	3.254189	-3.138276	-4.545394
H	4.315499	-3.854166	-3.337688
H	3.086027	-4.848403	-4.134757
B	-0.764119	-3.512747	0.319531
B	0.636273	-3.244330	0.729339
C	-2.082106	-3.994703	-0.076304
C	-2.332340	-5.420745	-0.576398
C	-3.741029	-5.305247	-1.201724
C	-4.446292	-4.115837	-0.524043
H	-4.314562	-6.226503	-1.098308
H	-3.640645	-5.098131	-2.268823
N	-3.264754	-3.338489	-0.024265

C	-3.332716	-2.001780	0.493288
C	-3.517788	-1.801880	1.876698
C	-3.139095	-0.912535	-0.384522
C	-3.600393	-0.490335	2.345740
C	-3.210268	0.376827	0.143740
C	-3.458413	0.590335	1.490304
H	-3.747203	-0.313711	3.403860
H	-3.032184	1.226207	-0.500595
H	-3.498975	1.600516	1.877133
C	-1.297916	-5.884594	-1.605222
H	-0.294075	-5.870871	-1.177612
H	-1.523934	-6.903683	-1.930384
H	-1.299305	-5.235972	-2.479939
C	-2.309501	-6.391031	0.622181
H	-2.610874	-7.390677	0.298040
H	-1.304246	-6.446761	1.038220
H	-2.980285	-6.074613	1.419780
C	-5.281001	-3.307374	-1.519783
H	-4.704609	-3.031638	-2.399636
H	-6.129401	-3.910465	-1.848056
H	-5.672087	-2.399215	-1.058953
C	-5.377079	-4.554345	0.615582
H	-6.205481	-5.129895	0.199084
H	-4.873271	-5.178184	1.350114
H	-5.794059	-3.686537	1.124954
C	-2.803228	-1.080976	-1.861071
H	-2.846259	-2.142676	-2.096279
C	-3.548689	-2.945738	2.879958
H	-3.537297	-3.877682	2.323136
C	-2.285673	-2.945171	3.756361
H	-2.280344	-3.824137	4.405880
H	-1.386950	-2.973024	3.138923
H	-2.248633	-2.059021	4.392945
C	-4.806687	-2.928899	3.759448
H	-4.834829	-3.817887	4.394090
H	-4.819640	-2.056034	4.415858
H	-5.719718	-2.908049	3.163556
C	-1.372825	-0.613153	-2.172780
H	-1.160962	-0.747583	-3.236102
H	-1.221821	0.436159	-1.918671
H	-0.639599	-1.200504	-1.619449
C	-3.806253	-0.346214	-2.764184
H	-3.604528	-0.575569	-3.813302
H	-4.836055	-0.629812	-2.544637
H	-3.724476	0.735621	-2.642188
N	-0.461770	0.205244	4.095330
N	-0.431994	-0.200154	3.084256
Li	-0.103115	-0.565883	0.874166
Cl	0.178294	1.526323	0.388929

TS_{1LiCl}

110

Li	0.408164	-1.828133	-0.139750
Cl	-0.427931	-3.787749	-0.339817
C	-3.079150	-0.685064	2.784403
C	-3.839905	-1.095343	1.519953
C	-1.780454	-0.072837	0.843491
C	-1.630595	-0.357172	2.355454
H	-3.111065	-1.463102	3.546764
H	-3.542680	0.207940	3.207845
N	-3.039519	-0.360114	0.466850
C	-3.785688	-2.614512	1.303636
H	-4.391759	-2.915002	0.452738
H	-2.770861	-2.973710	1.138445
H	-4.191058	-3.109981	2.188016
C	-0.667262	-1.520973	2.641616
H	-0.960977	-2.438652	2.134316
H	0.344950	-1.261363	2.328080
H	-0.634559	-1.717351	3.715629
C	-1.142411	0.898223	3.100714
H	-1.165417	0.711667	4.177812
H	-0.128989	1.160690	2.806559
H	-1.784536	1.754565	2.887652
C	-5.297010	-0.644477	1.543283
H	-5.797539	-0.865308	0.599699
H	-5.817263	-1.184579	2.335596
H	-5.390328	0.420809	1.743863
C	-3.643077	0.147387	-0.738728
C	-3.771530	-0.659244	-1.884010
C	-4.084112	1.490399	-0.734320
C	-4.378330	-0.104733	-3.012423
C	-4.663368	1.996343	-1.895445
C	-4.819175	1.207395	-3.025419

H	-4.485132	-0.709362	-3.903651
H	-4.995547	3.026089	-1.918754
H	-5.276345	1.619412	-3.916821
C	-3.227851	-2.073145	-1.976719
H	-2.728060	-2.317895	-1.043771
C	-3.919138	2.422453	0.459581
H	-3.602544	1.828229	1.314471
C	-2.167162	-2.204302	-3.080206
H	-2.598901	-2.056990	-4.073132
H	-1.366364	-1.478731	-2.951294
H	-1.718301	-3.196710	-3.035473
C	-4.347757	-3.098681	-2.211090
H	-5.156938	-2.994603	-1.486223
H	-4.783926	-2.982492	-3.206303
H	-3.947067	-4.111419	-2.134314
C	-2.821917	3.469907	0.204446
H	-2.714237	4.122860	1.074032
H	-1.859062	2.994514	0.020770
H	-3.072738	4.093056	-0.656866
C	-5.235339	3.115118	0.846070
H	-6.046121	2.397630	0.980495
H	-5.106484	3.664728	1.781335
H	-5.549015	3.831944	0.084980
B	0.802381	1.037565	0.194986
B	-0.610704	0.461290	0.101403
C	2.076469	1.684308	0.091616
C	2.281536	3.197989	0.237736
C	3.827041	3.328143	0.241239
C	4.394821	2.056000	-0.420357
H	4.180973	3.375271	1.273315
H	4.163728	4.235280	-0.262219
N	3.307550	1.087663	-0.113923
C	3.406792	-0.330461	0.028707
C	3.628423	-0.865556	1.317918
C	3.147044	-1.185123	-1.065780
C	3.588550	-2.250522	1.484085
C	3.114847	-2.565615	-0.845163
C	3.326931	-3.097427	0.418608
H	3.744976	-2.673102	2.468724
H	2.904895	-3.234576	-1.669366
H	3.269293	-4.167249	0.571072
C	1.627369	3.960804	-0.927371
H	1.826025	5.032855	-0.840685
H	1.995846	3.622992	-1.894940
H	0.546019	3.810398	-0.915771
C	1.687580	3.713175	1.558206
H	2.092056	3.160513	2.408054
H	1.917356	4.774324	1.693115
H	0.602339	3.593564	1.566371
C	4.587140	2.255835	-1.936157
H	3.647164	2.484865	-2.435293
H	5.275926	3.084763	-2.113221
H	5.012485	1.365538	-2.397057
C	5.739385	1.632036	0.170951
H	6.030275	0.644490	-0.192922
H	6.510194	2.341293	-0.134665
H	5.719132	1.607033	1.257681
C	2.902609	-0.662639	-2.471891
H	2.858745	0.421214	-2.414216
C	3.880712	0.008018	2.536912
H	3.931753	1.040171	2.198242
C	2.723423	-0.066886	3.541117
H	2.923811	0.580423	4.398434
H	1.797161	0.262175	3.075006
H	2.580619	-1.084054	3.913172
C	5.213050	-0.339520	3.218733
H	5.425009	0.375484	4.017131
H	5.181574	-1.335069	3.666638
H	6.044399	-0.319680	2.512296
C	1.559703	-1.133769	-3.043268
H	1.509810	-2.218922	-3.145309
H	0.738343	-0.813440	-2.401691
H	1.389491	-0.696226	-4.029458
C	4.052518	-1.058288	-3.410974
H	4.078473	-2.140519	-3.557676
H	3.925863	-0.588982	-4.389355
H	5.021009	-0.756270	-3.009491
N	-1.156421	0.925443	-1.713636
N	-1.080203	1.434037	-2.685753

Int$_{1LiCl}$

110

C	4.609485	-1.967693	1.582671

C	4.717308	-1.312887	0.185097
C	2.402898	-1.441020	0.769323
C	3.112767	-2.317879	1.811980
H	4.937898	-1.248321	2.335777
H	5.249898	-2.846123	1.672331
N	3.370648	-0.711368	0.063851
C	5.824989	-0.265190	0.108883
H	5.734059	0.478496	0.898218
H	6.796300	-0.752191	0.211095
H	5.807431	0.251937	-0.852460
C	2.671042	-1.954943	3.239826
H	3.230847	-2.537457	3.977797
H	2.831562	-0.896686	3.444950
H	1.607477	-2.164495	3.378444
C	2.839997	-3.815040	1.585285
H	3.122830	-4.127920	0.581048
H	3.399705	-4.419656	2.305181
H	1.777610	-4.031615	1.711015
C	4.971373	-2.379676	-0.898422
H	5.105869	-1.919922	-1.876599
H	5.876323	-2.945789	-0.665875
H	4.138024	-3.078976	-0.963939
C	3.024491	0.549650	-0.506074
C	2.983841	1.697029	0.319294
C	2.647291	0.639553	-1.862298
C	2.589974	2.915515	-0.242394
C	2.261434	1.877344	-2.379547
C	2.235434	3.013273	-1.584221
H	2.588990	3.810396	0.370752
H	1.970848	1.954722	-3.419619
H	1.933351	3.966775	-2.000375
C	3.359192	1.653642	1.791882
H	3.781665	0.671662	1.986931
C	2.640102	-0.578557	-2.767802
H	2.849847	-1.442586	-2.141337
C	2.129731	1.800654	2.699973
H	1.406881	1.010333	2.502488
H	2.425956	1.736477	3.749868
H	1.641512	2.767237	2.552642
C	4.423528	2.700742	2.153727
H	4.763409	2.549091	3.180919

H	5.290984	2.637960	1.494379
H	4.028009	3.716377	2.086055
C	1.268213	-0.812118	-3.413862
H	1.275838	-1.745715	-3.981153
H	0.492036	-0.883467	-2.652363
H	1.000620	-0.005897	-4.099522
C	3.734561	-0.476658	-3.840800
H	3.529615	0.348071	-4.527354
H	4.716270	-0.300190	-3.397125
H	3.785377	-1.398001	-4.426375
B	-0.506169	-0.941580	0.266350
B	1.011165	-1.388301	0.482329
C	-1.758168	-1.675612	-0.192559
C	-1.761070	-3.149738	-0.580241
C	-3.175063	-3.350531	-1.172844
C	-4.055757	-2.207865	-0.646007
H	-3.591096	-4.325978	-0.921234
H	-3.119803	-3.289454	-2.260776
N	-3.002025	-1.202408	-0.242798
C	-3.312421	0.165998	0.097348
C	-3.545251	0.511159	1.443201
C	-3.259129	1.142879	-0.917231
C	-3.795728	1.852032	1.735777
C	-3.503850	2.468452	-0.563176
C	-3.781219	2.822263	0.747424
H	-3.969093	2.145793	2.762844
H	-3.423069	3.247596	-1.308230
H	-3.946143	3.862094	0.999312
C	-0.686726	-3.490899	-1.621288
H	0.313472	-3.322997	-1.222485
H	-0.782356	-4.541294	-1.908278
H	-0.803511	-2.880940	-2.517768
C	-1.532829	-4.005403	0.682520
H	-1.623722	-5.064231	0.429155
H	-0.535082	-3.826694	1.082307
H	-2.254806	-3.778785	1.467327
C	-4.988083	-1.651432	-1.720607
H	-4.452111	-1.404078	-2.633781
H	-5.733900	-2.409174	-1.964169
H	-5.512623	-0.762315	-1.368391
C	-4.907812	-2.630829	0.555728

H	-5.635560	-3.373724	0.226628
H	-4.312161	-3.074609	1.350344
H	-5.454931	-1.781052	0.960199
C	-2.849054	0.846495	-2.353733
H	-2.721193	-0.229385	-2.461292
C	-3.434658	-0.470317	2.602689
H	-3.245414	-1.460329	2.196423
C	-2.240134	-0.131851	3.513164
H	-2.171388	-0.863895	4.321392
H	-1.301142	-0.152142	2.961936
H	-2.353468	0.855844	3.964193
C	-4.723885	-0.533569	3.435664
H	-4.648465	-1.322527	4.187547
H	-4.898152	0.407275	3.961634
H	-5.599926	-0.733823	2.818502
C	-1.494996	1.494043	-2.689505
H	-1.220117	1.264950	-3.721057
H	-1.531591	2.579625	-2.581876
H	-0.700806	1.111253	-2.049702
C	-3.914743	1.305322	-3.361091
H	-3.640278	0.987059	-4.369440
H	-4.897819	0.895086	-3.128221
H	-4.000797	2.393305	-3.370339
N	-0.564210	0.461924	0.544168
N	-0.459328	1.576774	0.696442
Li	-0.097140	3.378682	-0.060844
Cl	-0.963216	5.081078	-1.001817

TS_{2LiCl}

112

Li	1.963259	-1.518929	-1.734996
Cl	2.364981	-3.550511	-2.218264
C	3.900283	0.279498	-0.074465
C	3.146107	0.304552	1.261950
B	0.182365	0.114203	-1.076789
C	1.500126	0.205758	-0.501565
C	1.695852	-0.155125	0.996523
H	3.134382	1.328089	1.642475
H	3.633250	-0.314339	2.016039
N	2.775503	0.480725	-1.074875
C	4.948453	1.387621	-0.149110
H	4.517954	2.367620	0.038420
H	5.708798	1.201363	0.611004
H	5.441665	1.409253	-1.121445
C	0.728837	0.616560	1.908709
H	1.008010	0.465725	2.955101
H	0.747892	1.686670	1.700678
H	-0.293836	0.265147	1.780091
C	1.508400	-1.657672	1.279080
H	0.472101	-1.945882	1.103411
H	2.138667	-2.295492	0.658504
H	1.748958	-1.872533	2.323561
C	4.615304	-1.064274	-0.296851
H	5.197551	-1.054812	-1.216745
H	5.305286	-1.248045	0.528969
H	3.933073	-1.912379	-0.349197
C	2.920004	1.437634	-2.147301
C	2.534321	2.789322	-1.963506
C	3.442117	1.017358	-3.389230
C	2.652277	3.669882	-3.036176
C	3.554542	1.946634	-4.424937
C	3.157092	3.260931	-4.260963
H	2.347598	4.700782	-2.909091
H	3.949327	1.626938	-5.380724
H	3.242213	3.964998	-5.079583
C	1.974820	3.340685	-0.660442
H	2.022185	2.555332	0.088070
C	3.864206	-0.412237	-3.684532
H	3.702959	-1.018983	-2.796511
C	0.500354	3.740244	-0.817044
H	-0.089953	2.912403	-1.201796
H	0.077558	4.036371	0.144964
H	0.394761	4.581193	-1.505723
C	2.784089	4.535779	-0.129763
H	3.846109	4.305085	-0.038745
H	2.690668	5.403631	-0.785485
H	2.413564	4.826081	0.855987
C	3.038412	-1.038188	-4.819396
H	3.279367	-2.097418	-4.910921
H	1.966193	-0.963021	-4.629066
H	3.240146	-0.546936	-5.773569
C	5.365449	-0.510973	-3.993312
H	5.611017	0.008553	-4.922205

H	5.966987	-0.068998	-3.196954
H	5.657594	-1.557148	-4.104101
B	-1.446695	0.021791	-0.877236
C	-2.460002	-0.973303	-1.392180
C	-2.087485	-2.334114	-1.990662
C	-3.454308	-3.044334	-2.136481
C	-4.533072	-1.958949	-2.131906
H	-3.607572	-3.704388	-1.281212
H	-3.502782	-3.658989	-3.034950
N	-3.800557	-0.841754	-1.433644
C	-4.488945	0.295827	-0.887451
C	-4.906173	0.240966	0.457952
C	-4.670040	1.457025	-1.664418
C	-5.571267	1.345862	0.986033
C	-5.346191	2.531437	-1.085481
C	-5.804318	2.477523	0.220549
H	-5.894389	1.328902	2.018730
H	-5.495278	3.435680	-1.661398
H	-6.321809	3.326678	0.649887
C	-1.383207	-2.175068	-3.350068
H	-1.247242	-3.155160	-3.810445
H	-1.949589	-1.544944	-4.036242
H	-0.394887	-1.737920	-3.225520
C	-1.194051	-3.145150	-1.043679
H	-1.641631	-3.210222	-0.049741
H	-1.064543	-4.156165	-1.435879
H	-0.204826	-2.709299	-0.948995
C	-4.946609	-1.547450	-3.550687
H	-5.411832	-2.400425	-4.047100
H	-5.674841	-0.738844	-3.522874
H	-4.096282	-1.231554	-4.151910
C	-5.789625	-2.394789	-1.378676
H	-6.495251	-1.570356	-1.267187
H	-6.281757	-3.185383	-1.946991
H	-5.555374	-2.790895	-0.393810
C	-4.091826	1.636517	-3.060926
H	-3.612833	0.705065	-3.351801
C	-4.590922	-0.930183	1.379784
H	-4.165594	-1.731534	0.778736
C	-3.525236	-0.546696	2.420665
H	-3.862281	0.283943	3.044512
H	-3.317654	-1.397956	3.073021
H	-2.592541	-0.255234	1.940349
C	-5.844619	-1.475302	2.080169
H	-6.242876	-0.755017	2.797739
H	-6.636524	-1.708496	1.368027
H	-5.599836	-2.385803	2.631797
C	-3.002743	2.723060	-3.079649
H	-3.410983	3.698600	-2.808553
H	-2.197362	2.490686	-2.386592
H	-2.574262	2.803871	-4.080878
C	-5.172074	1.970197	-4.101779
H	-5.600623	2.957990	-3.919114
H	-4.738629	1.977619	-5.104114
H	-5.990306	1.249770	-4.090354
N	-1.917492	1.192347	-0.169934
N	-2.151545	2.101614	0.444766
N	0.311265	0.778082	-2.945016
N	-0.031838	1.150443	-3.918835

Int$_{2LiCl}$

112

Li	-0.361759	3.912722	-0.436696
Cl	1.541698	4.822242	-0.161436
C	-3.438552	-3.194626	0.922003
C	-4.356624	-2.171168	0.240647
C	-2.116356	-1.277600	0.324094
C	-1.983797	-2.771482	0.620529
H	-3.649314	-4.211108	0.589909
H	-3.604820	-3.160658	2.000235
N	-3.417984	-1.000370	0.110047
C	-4.861244	-2.644488	-1.128530
H	-5.443716	-1.862170	-1.613295
H	-4.052676	-2.937741	-1.793342
H	-5.511776	-3.509022	-0.988349
C	-1.426830	-3.498590	-0.620735
H	-1.993532	-3.268730	-1.522326
H	-0.391541	-3.222726	-0.801646
H	-1.467517	-4.578622	-0.460123
C	-1.078032	-3.070987	1.819170
H	-1.084569	-4.145104	2.022371
H	-0.053460	-2.759744	1.622720
H	-1.424864	-2.551689	2.713470

C	-5.578201	-1.840207	1.098107
H	-6.158071	-1.024575	0.663999
H	-6.221593	-2.720097	1.149711
H	-5.300295	-1.571596	2.114485
C	-3.861948	0.310032	-0.278434
C	-3.829807	0.683934	-1.638008
C	-4.233972	1.226978	0.725053
C	-4.256950	1.968806	-1.972291
C	-4.650570	2.498491	0.332956
C	-4.676691	2.866013	-1.003022
H	-4.242096	2.278237	-3.009329
H	-4.940901	3.218451	1.087207
H	-5.004773	3.858544	-1.287499
C	-3.276139	-0.203157	-2.745495
H	-3.011574	-1.162804	-2.309943
C	-4.116228	0.928878	2.213585
H	-3.825211	-0.113395	2.328002
C	-1.985044	0.380455	-3.346101
H	-2.168033	1.350524	-3.812930
H	-1.213073	0.506698	-2.590353
H	-1.596176	-0.293248	-4.112897
C	-4.303161	-0.449268	-3.861929
H	-4.517593	0.471463	-4.408614
H	-3.911142	-1.174055	-4.579032
H	-5.246910	-0.831419	-3.473242
C	-3.001396	1.770127	2.855662
H	-2.903747	1.519298	3.914430
H	-2.044006	1.579010	2.375876
H	-3.215696	2.838299	2.780548
C	-5.440544	1.143119	2.961709
H	-6.259070	0.583928	2.508119
H	-5.340501	0.821062	4.000624
H	-5.723047	2.197781	2.970541
B	0.745624	-0.582355	0.161127
B	-0.944832	-0.330530	0.230250
C	1.840136	-0.088476	1.060909
C	1.605399	0.512751	2.453422
C	3.022834	0.520681	3.066588
C	4.013976	0.553408	1.898696
H	3.172557	-0.401119	3.632955
H	3.172019	1.355828	3.750699
N	3.172378	-0.055603	0.812663
C	3.734391	-0.524434	-0.421026
C	4.125614	-1.876265	-0.512750
C	3.812838	0.337278	-1.533650
C	4.671399	-2.326013	-1.713526
C	4.371740	-0.165463	-2.709550
C	4.809314	-1.476742	-2.800556
H	4.974590	-3.361096	-1.806609
H	4.444141	0.478545	-3.576559
H	5.235456	-1.844162	-3.726341
C	1.035476	1.939712	2.346451
H	1.038033	2.410186	3.333180
H	1.599629	2.575514	1.667560
H	0.007816	1.914734	1.995719
C	0.663721	-0.333440	3.316584
H	1.017033	-1.362891	3.393604
H	0.611607	0.088720	4.324065
H	-0.341983	-0.350997	2.899204
C	4.460176	1.978055	1.539445
H	3.627321	2.638363	1.310675
H	5.007367	2.403342	2.382750
H	5.129812	1.962185	0.680862
C	5.267760	-0.271270	2.193527
H	5.902944	-0.357969	1.310825
H	5.843537	0.228572	2.974343
H	5.023003	-1.269992	2.547849
C	3.248587	1.750792	-1.548135
H	2.872946	1.989537	-0.558016
C	3.898421	-2.883110	0.607598
H	3.542028	-2.340504	1.481051
C	2.797125	-3.889235	0.232264
H	2.631119	-4.588324	1.055672
H	1.856904	-3.380534	0.026343
H	3.074332	-4.467524	-0.651859
C	5.184264	-3.627063	0.998052
H	5.005121	-4.253259	1.875426
H	5.522979	-4.281049	0.191857
H	5.995660	-2.936967	1.230087
C	2.051270	1.870840	-2.507038
H	2.334857	1.626745	-3.533384
H	1.237373	1.209687	-2.214272

H	1.685016	2.898662	-2.487946
C	4.306048	2.802433	-1.916564
H	4.603280	2.712913	-2.964316
H	3.891117	3.799441	-1.761450
H	5.206225	2.706872	-1.308494
N	1.182227	-1.600042	-2.142268
N	1.075436	-1.127324	-1.124459
N	-1.157828	1.025781	-0.092720
N	-1.136338	2.139983	-0.327101

Int$_{3LiCl}$

112

C	4.239243	5.952810	-2.087564
C	4.448395	4.884847	-2.943476
C	4.490506	3.576610	-2.468693
C	4.339759	3.371389	-1.083879
C	4.040206	4.434166	-0.211389
C	4.015618	5.724235	-0.740753
N	4.459281	2.032557	-0.568588
C	3.400643	1.216944	-0.436371
C	3.835801	-0.126358	0.132063
C	5.378296	-0.045848	0.029429
C	5.757672	1.441708	-0.099406
B	2.013804	1.559640	-0.757011
N	0.788512	1.804078	-0.958459
N	-0.441485	2.085239	-1.164276
B	-1.467908	1.070290	-0.881299
N	-0.921387	-0.189666	-0.447134
N	-0.333204	-1.079094	-0.082730
C	3.347548	-0.257028	1.587503
C	3.288130	-1.296904	-0.694974
C	6.187220	2.053774	1.240268
C	6.881569	1.662525	-1.112759
C	4.579857	2.429027	-3.463855
C	5.747343	2.582414	-4.447805
C	3.632677	4.228239	1.239736
C	4.453802	5.072604	2.223873
C	-2.968046	1.130803	-0.892488
C	-3.901744	-0.064682	-0.649711
C	-5.285299	0.497687	-1.051677
C	-5.207660	2.024427	-0.953876
N	-3.722247	2.235990	-1.072030
C	-5.987676	2.708006	-2.078974
C	-5.747015	2.559821	0.379585
C	-3.587599	-1.284025	-1.531766
C	-3.876731	-0.481865	0.834897
C	-3.167387	3.544488	-1.284010
C	-2.975116	3.987119	-2.610078
C	-2.477982	5.274682	-2.806612
C	-2.161171	6.094075	-1.732595
C	-2.306611	5.622407	-0.438417
C	-2.796623	4.338991	-0.182657
C	-3.194546	3.088221	-3.821074
C	-4.066037	3.754094	-4.895400
C	-2.811021	3.840766	1.255873
C	-3.620607	4.751883	2.190776
C	-1.852523	2.637646	-4.424560
C	-1.378761	3.678856	1.792735
C	2.131199	4.515641	1.410910
C	3.247822	2.280280	-4.218945
Li	-0.024694	3.793993	-2.056004
Cl	0.933260	5.138094	-3.436231
H	-5.497216	0.218678	-2.085136
H	-6.085506	0.090644	-0.433415
H	-5.822709	3.786089	-2.079078
H	-5.721595	2.313782	-3.056452
H	-7.053357	2.530753	-1.925142
H	-2.671982	-1.793588	-1.239152
H	-4.403986	-2.006808	-1.449985
H	-3.495026	-0.991499	-2.578931
H	-4.620810	-1.262796	1.011207
H	-2.902224	-0.874975	1.122267
H	-4.103118	0.355920	1.493681
H	-5.617274	3.639383	0.438621
H	-6.815184	2.345738	0.443901
H	-5.260148	2.104878	1.238805
H	-2.285898	5.626922	-3.809875
H	-2.007683	6.251265	0.390464
H	-1.752388	7.079849	-1.911126
H	-3.704021	2.187605	-3.484513
H	-3.264842	2.853011	1.267452
H	-2.030045	2.007700	-5.299604
H	-1.244358	3.490856	-4.731359

H	-1.276881	2.055050	-3.703266
H	-4.296661	3.036672	-5.686057
H	-5.005943	4.123696	-4.483088
H	-3.548967	4.596662	-5.357907
H	-0.799017	3.003635	1.166395
H	-0.860376	4.639003	1.824708
H	-1.402077	3.275807	2.808289
H	-4.632879	4.921843	1.823548
H	-3.689863	4.302657	3.184127
H	-3.141188	5.726549	2.303637
H	5.706478	-0.577391	-0.865122
H	5.868767	-0.510833	0.884538
H	4.535533	5.068171	-4.005616
H	3.784717	6.559511	-0.092065
H	4.195051	6.960178	-2.481414
H	3.704652	-1.196230	2.016915
H	3.708288	0.562151	2.210081
H	2.256857	-0.253836	1.625739
H	2.199664	-1.339591	-0.647684
H	3.580061	-1.204077	-1.742359
H	3.681224	-2.240814	-0.309031
H	7.119595	1.588832	1.564650
H	6.362152	3.123672	1.134421
H	5.443360	1.898215	2.019131
H	7.061337	2.725610	-1.277395
H	7.799943	1.218596	-0.725435
H	6.659068	1.195071	-2.069463
H	3.781831	3.180652	1.490999
H	4.733816	1.505399	-2.907964
H	2.997054	3.191789	-4.763050
H	3.310255	1.450626	-4.928550
H	2.427520	2.086010	-3.528527
H	6.696507	2.725839	-3.928620
H	5.830622	1.690266	-5.073106
H	5.597062	3.435012	-5.112294
H	1.901547	5.558197	1.179836
H	1.536332	3.883372	0.753395
H	1.820860	4.318044	2.439820
H	4.268547	6.139270	2.082141
H	4.179508	4.824448	3.251903
H	5.525308	4.903980	2.108474

TS_{3LiCl}

112

C	-4.794053	2.509654	-1.843642
C	-5.044758	1.088900	-1.313131
C	-2.678993	1.514697	-1.309723
C	-3.282898	2.628015	-2.155809
H	-5.055380	3.232184	-1.068556
H	-5.410063	2.729371	-2.715782
N	-3.657882	0.700481	-0.876433
C	-6.032606	1.072641	-0.145638
H	-6.098404	0.079618	0.300815
H	-5.757055	1.785024	0.627551
H	-7.022764	1.343551	-0.515469
C	-2.773629	4.023714	-1.766017
H	-1.719270	4.148257	-2.005531
H	-3.345109	4.780720	-2.309214
H	-2.902861	4.199729	-0.696357
C	-2.969292	2.369412	-3.643357
H	-3.460487	3.124028	-4.262937
H	-1.894287	2.423310	-3.818340
H	-3.314186	1.386825	-3.965666
C	-5.586134	0.153870	-2.403274
H	-5.686087	-0.863467	-2.028477
H	-6.576316	0.499833	-2.703881
H	-4.953932	0.140758	-3.288569
C	-3.366638	-0.434330	-0.041807
C	-3.279069	-0.243901	1.353201
C	-3.061175	-1.673212	-0.634090
C	-2.936294	-1.338704	2.143465
C	-2.727457	-2.737247	0.205661
C	-2.666885	-2.576530	1.579761
H	-2.822422	-1.215015	3.210570
H	-2.486372	-3.699689	-0.227995
H	-2.379799	-3.403910	2.216305
C	-3.472756	1.107115	2.029615
H	-3.777915	1.827498	1.272889
C	-2.994504	-1.885739	-2.138908
H	-3.318604	-0.970437	-2.624883
C	-2.161425	1.630520	2.641078
H	-2.337811	2.593278	3.127504

H	-1.761922	0.939443	3.385373
H	-1.402756	1.779566	1.872766
C	-4.569467	1.052096	3.104791
H	-4.765017	2.053866	3.494611
H	-5.503608	0.649494	2.711154
H	-4.262939	0.425249	3.944113
C	-1.550495	-2.134883	-2.596869
H	-0.906868	-1.303910	-2.314176
H	-1.144869	-3.045374	-2.152097
H	-1.510393	-2.243784	-3.683645
C	-3.909077	-3.025605	-2.610379
H	-4.935910	-2.891853	-2.267362
H	-3.917966	-3.075972	-3.701638
H	-3.560239	-3.990969	-2.237886
B	1.971106	1.350825	-0.671006
B	-1.220763	1.372161	-1.020763
C	3.428252	1.234817	-0.750557
C	4.281807	2.308845	-1.435227
C	5.709214	1.947302	-0.957264
C	5.698128	0.466488	-0.544175
H	5.957027	2.556483	-0.085475
H	6.459200	2.149748	-1.721845
N	4.240906	0.269732	-0.251484
C	3.725159	-0.809692	0.535781
C	3.573443	-0.615360	1.926208
C	3.322307	-2.004137	-0.085221
C	3.075007	-1.673875	2.686582
C	2.821464	-3.030786	0.719499
C	2.707285	-2.875546	2.091527
H	2.929386	-1.550812	3.750113
H	2.497578	-3.956742	0.261663
H	2.303405	-3.673183	2.701697
C	4.130629	2.203993	-2.964942
H	4.800075	2.913999	-3.457716
H	4.365313	1.204686	-3.330246
H	3.105811	2.435471	-3.257717
C	3.920947	3.734953	-0.995622
H	2.925354	4.013483	-1.339585
H	3.942212	3.824425	0.091887
H	4.640829	4.443380	-1.414652
C	6.182397	-0.453439	-1.676011
H	7.227331	-0.229019	-1.897406
H	6.121316	-1.499797	-1.378675
H	5.608580	-0.318609	-2.590596
C	6.575357	0.201640	0.681463
H	6.450507	-0.819967	1.044136
H	7.622395	0.333468	0.404228
H	6.355785	0.888330	1.494700
C	3.320724	-2.184965	-1.593608
H	3.807321	-1.320634	-2.035614
C	3.859939	0.714870	2.611091
H	4.360513	1.361455	1.893257
C	2.554808	1.424296	3.011406
H	1.980157	0.829876	3.725511
H	2.779249	2.386906	3.477068
H	1.931983	1.619838	2.135976
C	4.778119	0.556796	3.831204
H	5.693062	0.017451	3.580562
H	5.054949	1.539229	4.220045
H	4.279244	0.014032	4.636068
C	1.882384	-2.201820	-2.127438
H	1.318159	-3.042903	-1.719602
H	1.360170	-1.285380	-1.857323
H	1.879240	-2.290004	-3.216356
C	4.081892	-3.440492	-2.039521
H	3.575853	-4.350744	-1.710825
H	4.145411	-3.474037	-3.129375
H	5.096228	-3.462503	-1.638482
N	-0.145871	2.353684	-1.388732
N	1.030329	2.141345	-1.234197
N	-0.546714	0.432813	-0.194081
N	0.539293	0.197073	0.220558
Li	0.549697	-0.837750	2.013142
Cl	-0.038221	-1.274419	4.045957

Pdt_{LiCl}

112

C	2.951899	-3.639391	0.130747
C	3.001112	-2.926197	1.315936
C	3.265907	-1.557415	1.326449
C	3.498375	-0.931144	0.090354
C	3.383870	-1.617203	-1.132205
C	3.122758	-2.984342	-1.076212

N	3.907260	0.460028	0.089724
C	3.116656	1.489550	-0.057820
C	3.929654	2.779468	-0.033988
C	5.321033	2.309724	0.442631
C	5.395113	0.803138	0.186185
B	1.540083	1.484695	-0.250293
N	0.984016	2.735539	-0.684474
N	-0.311576	2.863740	-0.737777
B	-1.138114	1.753770	-0.354752
N	-0.593710	0.458123	-0.134348
N	0.734308	0.326037	-0.083407
C	3.981678	3.387992	-1.452640
C	3.349452	3.809945	0.948554
C	6.097802	0.450624	-1.127416
C	6.090171	0.057520	1.321515
C	3.188440	-0.805402	2.649307
C	4.031131	-1.452695	3.758177
C	3.414034	-0.939127	-2.497119
C	4.390632	-1.612065	-3.473295
C	-2.691613	2.080237	-0.277245
C	-3.239562	3.498184	-0.399896
C	-4.721936	3.341944	0.006169
C	-5.070832	1.858395	-0.130876
N	-3.674866	1.235859	-0.110274
C	-5.938368	1.356305	1.020035
C	-5.778705	1.534501	-1.449174
C	-2.535636	4.481227	0.550540
C	-3.085171	3.982844	-1.857162
C	-3.527876	-0.196283	0.057701
C	-3.421074	-0.702270	1.363784
C	-3.369675	-2.087724	1.515051
C	-3.407430	-2.931821	0.418552
C	-3.458995	-2.402552	-0.859583
C	-3.506217	-1.027311	-1.076078
C	-3.267169	0.176109	2.599025
C	-4.241922	-0.201726	3.723972
C	-3.421737	-0.512192	-2.507483
C	-4.479200	-1.147760	-3.422874
C	-1.819355	0.133160	3.114174
C	-2.016903	-0.739137	-3.093191
C	2.002632	-0.899065	-3.111225
C	1.724770	-0.675632	3.102036
Li	-0.096266	-1.427425	-0.203372
Cl	-0.271223	-3.439022	-1.053592
H	-4.847171	3.647780	1.046027
H	-5.378177	3.965645	-0.599512
H	-6.083203	0.277220	0.974515
H	-5.518477	1.617191	1.987758
H	-6.915910	1.833791	0.941447
H	-1.505273	4.643916	0.245309
H	-3.073800	5.432501	0.534233
H	-2.544363	4.106911	1.577143
H	-3.549809	4.966849	-1.954161
H	-2.030710	4.055040	-2.114589
H	-3.572609	3.308998	-2.563237
H	-5.933300	0.462521	-1.555855
H	-6.756360	2.017352	-1.440893
H	-5.231442	1.899701	-2.315306
H	-3.274003	-2.510826	2.506659
H	-3.397006	-3.071316	-1.705425
H	-3.338274	-4.002821	0.554073
H	-3.468682	1.209976	2.319655
H	-3.590229	0.562562	-2.502436
H	-1.707051	0.783845	3.984860
H	-1.542380	-0.880564	3.411166
H	-1.123321	0.458499	2.344451
H	-4.174276	0.524122	4.537746
H	-5.274771	-0.231287	3.375714
H	-4.003852	-1.182226	4.140406
H	-1.268007	-0.174392	-2.541761
H	-1.731787	-1.791029	-3.047250
H	-1.992579	-0.412508	-4.135895
H	-5.486208	-1.058345	-3.013209
H	-4.464549	-0.663810	-4.401844
H	-4.277663	-2.209045	-3.578698
H	5.420879	2.498524	1.512698
H	6.126545	2.842527	-0.060974
H	2.803866	-3.436342	2.249671
H	2.993671	-3.537264	-1.995592
H	2.717563	-4.695060	0.141274
H	4.631728	4.265899	-1.435042
H	4.385389	2.684845	-2.182223

H	2.982994	3.682120	-1.766913
H	2.392988	4.186660	0.594720
H	3.211019	3.371240	1.939881
H	4.051295	4.642270	1.043632
H	7.146843	0.736998	-1.045705
H	6.053696	-0.620761	-1.313538
H	5.671528	0.972422	-1.980781
H	6.039049	-1.022579	1.186150
H	7.141391	0.349077	1.324167
H	5.672190	0.314178	2.291285
H	3.732882	0.093781	-2.369353
H	3.559810	0.207715	2.496667
H	1.283213	-1.660069	3.273322
H	1.662804	-0.109370	4.034502
H	1.130647	-0.166273	2.346833
H	5.064365	-1.610141	3.447517
H	4.032662	-0.816538	4.646176
H	3.621833	-2.421086	4.051381
H	1.578429	-1.900991	-3.193109
H	1.326142	-0.306916	-2.499233
H	2.042581	-0.452864	-4.107959
H	4.048782	-2.611820	-3.746344
H	4.459386	-1.027127	-4.393035
H	5.392849	-1.706082	-3.054005

System (2)

132

B	5.544169	11.990122	3.869717
B	4.100444	12.122307	3.954047
C	7.013172	11.852197	3.768048
N	7.991230	12.718708	4.227546
C	9.257142	12.230156	3.935760
H	10.146292	12.773066	4.199064
C	9.098254	11.056919	3.292493
H	9.821716	10.358203	2.914231
N	7.734395	10.823597	3.185223
C	2.629258	12.262816	4.043854
N	1.842173	13.321696	3.623689
C	0.503229	13.075643	3.891287
H	-0.261694	13.788945	3.642692
C	0.426115	11.864984	4.480858
H	-0.420917	11.306357	4.836209
N	1.716933	11.366897	4.573334
C	7.751279	14.031058	4.743843
C	7.707564	14.214290	6.132879
C	7.575513	15.517973	6.608157
H	7.545914	15.701191	7.673850
C	7.454344	16.586910	5.730494
H	7.340683	17.591930	6.118949
C	7.454540	16.372144	4.360279
H	7.330373	17.211140	3.687599
C	7.611721	15.089238	3.835761
C	7.812838	13.031231	7.081159
H	7.496487	12.145798	6.526410
C	6.880757	13.159122	8.291402
H	7.206197	13.946881	8.974817
H	6.869563	12.221996	8.851736
H	5.861384	13.377941	7.976313
C	9.265054	12.812679	7.535388
H	9.928643	12.641147	6.686467
H	9.333250	11.945701	8.197046
H	9.632224	13.686424	8.080168
C	7.607503	14.865749	2.332765
H	7.848822	13.819758	2.145233
C	8.674074	15.710113	1.621614
H	8.472774	16.779224	1.720833
H	8.689061	15.474019	0.554997
H	9.669697	15.516461	2.027600
C	6.208688	15.118210	1.756951
H	5.472246	14.483476	2.251075
H	6.192140	14.897675	0.686450
H	5.910172	16.159046	1.892327
C	7.178097	9.600905	2.693112
C	6.866182	9.500667	1.329608
C	6.431831	8.263641	0.857542
H	6.186720	8.143135	-0.188972
C	6.282605	7.184279	1.718798
H	5.936082	6.232853	1.333450
C	6.547613	7.326079	3.072493
H	6.393912	6.486544	3.738378
C	7.005339	8.538606	3.589596
C	7.005020	10.701698	0.407509
H	6.885821	11.594038	1.024869

C	5.913496	10.754518	-0.666790
H	6.039769	9.967686	-1.414357
H	5.954226	11.712089	-1.189599
H	4.923475	10.653188	-0.223652
C	8.399497	10.751250	-0.237543
H	9.187789	10.801654	0.514632
H	8.491977	11.629927	-0.880652
H	8.570571	9.862067	-0.850031
C	7.264269	8.693590	5.078652
H	7.713364	9.672995	5.243693
C	8.253035	7.646498	5.608830
H	7.853684	6.633808	5.518726
H	8.459167	7.827025	6.666665
H	9.200313	7.684171	5.065869
C	5.939502	8.667071	5.853343
H	5.264130	9.438700	5.481821
H	6.116857	8.846122	6.917086
H	5.443461	7.700230	5.750729
C	2.343696	14.575756	3.151148
C	2.373918	14.817646	1.769842
C	2.822769	16.066807	1.343749
H	2.864827	16.290342	0.286282
C	3.234051	17.027065	2.258296
H	3.587086	17.988871	1.906166
C	3.206821	16.756573	3.617787
H	3.542041	17.508765	4.320421
C	2.754253	15.526605	4.095127
C	1.986318	13.735877	0.774683
H	1.326859	13.033696	1.287762
C	3.231010	12.947831	0.336391
H	3.926366	13.597496	-0.201801
H	2.945157	12.130455	-0.330056
H	3.758417	12.528447	1.195283
C	1.217494	14.273795	-0.437925
H	0.351263	14.864880	-0.132501
H	0.864702	13.442250	-1.051946
H	1.847327	14.900776	-1.072667
C	2.716847	15.252812	5.588950
H	2.297061	14.258753	5.741688
C	1.807479	16.248856	6.323256
H	2.189266	17.269557	6.247862

H	1.749430	15.993205	7.384041
H	0.794689	16.237816	5.913763
C	4.132264	15.237908	6.177155
H	4.744518	14.490339	5.671744
H	4.096307	14.995382	7.241999
H	4.618875	16.208824	6.070432
C	2.038913	10.054160	5.041214
C	2.260135	9.858296	6.411397
C	2.530297	8.560083	6.842524
H	2.714682	8.371749	7.892065
C	2.585159	7.506362	5.941132
H	2.803364	6.505970	6.294992
C	2.378816	7.730964	4.588014
H	2.442658	6.903541	3.892900
C	2.097314	9.009805	4.108427
C	2.263385	11.021681	7.388263
H	1.783326	11.870028	6.898592
C	3.705916	11.442440	7.708166
H	4.243721	10.631497	8.206494
H	3.707168	12.309971	8.372374
H	4.247821	11.701988	6.796982
C	1.469531	10.728612	8.667177
H	0.448679	10.413369	8.439558
H	1.419191	11.625748	9.288411
H	1.938378	9.943810	9.264975
C	1.883552	9.243950	2.622779
H	1.599859	10.286886	2.482071
C	0.740886	8.382343	2.066983
H	0.973988	7.317006	2.130231
H	0.567134	8.621691	1.015067
H	-0.188771	8.555400	2.614237
C	3.189083	9.022998	1.849144
H	3.975570	9.671998	2.236178
H	3.044374	9.246285	0.789000
H	3.528336	7.989057	1.932288

RC_2

134

B	5.498896	12.538135	2.927494
B	4.054101	12.678385	2.961171
C	6.965729	12.363399	2.905175
N	7.935868	13.187858	3.456546

C	9.201222	12.646269	3.266606
H	10.086648	13.139830	3.623398
C	9.050126	11.497488	2.579392
H	9.774722	10.779381	2.240918
N	7.693130	11.329919	2.338122
C	2.582979	12.833307	3.002940
N	1.818434	13.900809	2.564781
C	0.471199	13.671376	2.803805
H	-0.279596	14.393469	2.538183
C	0.367205	12.461082	3.390499
H	-0.493273	11.912901	3.729310
N	1.649728	11.946640	3.507032
C	7.718079	14.541474	3.870550
C	7.675481	14.838554	5.238900
C	7.558249	16.177519	5.610979
H	7.531608	16.441135	6.660062
C	7.451715	17.175636	4.654023
H	7.346197	18.209179	4.961384
C	7.464214	16.853057	3.304589
H	7.365554	17.639067	2.566637
C	7.610606	15.532765	2.881799
C	7.771142	13.745008	6.287886
H	7.568814	12.798740	5.784633
C	6.722045	13.906533	7.393885
H	6.919673	14.782414	8.015465
H	6.727241	13.032464	8.048204
H	5.724787	14.005902	6.968627
C	9.186838	13.673523	6.880867
H	9.933210	13.480141	6.108594
H	9.255270	12.875066	7.623311
H	9.445604	14.615216	7.371631
C	7.653344	15.208447	1.397732
H	7.867125	14.146387	1.290532
C	8.780695	15.962758	0.678602
H	8.615714	17.042511	0.691529
H	8.832645	15.647548	-0.366310
H	9.749625	15.764284	1.142390
C	6.291840	15.468459	0.740872
H	5.510856	14.894855	1.241213
H	6.316113	15.173821	-0.311665
H	6.025869	16.526011	0.788101

C	7.146669	10.209250	1.634372
C	6.886922	10.333199	0.258459
C	6.458120	9.194095	-0.419043
H	6.248948	9.245061	-1.478387
C	6.273699	7.989863	0.248900
H	5.935938	7.117757	-0.298055
C	6.487183	7.909275	1.614686
H	6.298040	6.976930	2.131197
C	6.924360	9.020158	2.338353
C	7.055112	11.668029	-0.453939
H	6.736887	12.439506	0.250488
C	6.164759	11.806747	-1.692414
H	6.508464	11.171712	-2.512749
H	6.188672	12.838888	-2.046824
H	5.129532	11.551256	-1.469240
C	8.520357	11.937162	-0.834252
H	9.176417	11.930569	0.035085
H	8.612145	12.915320	-1.313225
H	8.877829	11.180736	-1.538062
C	7.071187	8.941667	3.847639
H	7.552096	9.858377	4.189020
C	7.951066	7.768572	4.297316
H	7.504451	6.806241	4.037838
H	8.078108	7.789019	5.381778
H	8.941227	7.814683	3.838457
C	5.679408	8.885657	4.492882
H	5.086136	9.755207	4.205883
H	5.756038	8.864620	5.581527
H	5.141001	7.989493	4.179162
C	2.348656	15.131176	2.063253
C	2.390934	15.332606	0.675586
C	2.886639	16.551511	0.215677
H	2.939796	16.743087	-0.847728
C	3.329574	17.522042	1.104468
H	3.719931	18.459393	0.726510
C	3.282338	17.294127	2.471132
H	3.637581	18.055640	3.153710
C	2.784018	16.095399	2.981518
C	1.970126	14.235893	-0.289136
H	1.280391	13.574663	0.237932
C	3.189992	13.392349	-0.689034

H	3.909023	13.999122	-1.244616
H	2.883387	12.559692	-1.327288
H	3.698672	12.987906	0.188110
C	1.232565	14.762480	-1.525731
H	0.384475	15.392099	-1.247894
H	0.857004	13.924926	-2.117738
H	1.889599	15.347405	-2.172991
C	2.720064	15.870390	4.482194
H	2.279390	14.889438	4.659016
C	1.817520	16.907040	5.167408
H	2.219460	17.917417	5.062178
H	1.739206	16.690075	6.235504
H	0.810982	16.900051	4.742788
C	4.124047	15.850369	5.096113
H	4.732834	15.078767	4.623894
H	4.064697	15.639849	6.166621
H	4.627943	16.810123	4.971848
C	1.941421	10.631320	3.987294
C	2.183567	10.448339	5.354344
C	2.387928	9.144754	5.806589
H	2.583102	8.968580	6.856833
C	2.360735	8.073212	4.926612
H	2.524275	7.067894	5.295707
C	2.154530	8.286524	3.571045
H	2.169888	7.444840	2.890415
C	1.945037	9.570322	3.070092
C	2.272442	11.620351	6.315396
H	1.971205	12.519253	5.776587
C	3.725421	11.832637	6.766013
H	4.102731	10.958414	7.302544
H	3.789416	12.692856	7.435609
H	4.370416	12.015739	5.904677
C	1.325650	11.466139	7.513166
H	0.292842	11.326170	7.185963
H	1.366228	12.359118	8.141231
H	1.599169	10.611573	8.136120
C	1.781640	9.797122	1.576514
H	1.501525	10.839274	1.421147
C	0.667945	8.931545	0.972146
H	0.903551	7.867049	1.038716
H	0.536941	9.173894	-0.085161
H	-0.284655	9.097111	1.480799
C	3.121673	9.576242	0.859670
H	3.890417	10.232028	1.271356
H	3.021770	9.788225	-0.207849
H	3.459698	8.543736	0.968366
N	8.059729	10.310523	6.911463
N	7.188145	10.248658	7.564626

TS1_2

134

B	5.439975	12.292448	3.263404
B	4.067862	12.151986	3.862401
C	6.846819	11.886445	3.358369
N	7.852618	12.516373	4.095149
C	9.032257	11.780512	4.039413
H	9.922448	12.106777	4.545887
C	8.807053	10.706019	3.264791
H	9.461882	9.907114	2.969201
N	7.489564	10.770591	2.815725
C	2.593543	12.462471	3.869979
N	2.007710	13.547000	3.256622
C	0.632490	13.531781	3.434008
H	0.005613	14.303003	3.025196
C	0.334570	12.442362	4.165171
H	-0.609080	12.055238	4.502021
N	1.529515	11.782590	4.435204
C	7.792353	13.849937	4.603053
C	7.573014	14.052843	5.972711
C	7.635170	15.360410	6.456266
H	7.482558	15.546620	7.512034
C	7.865998	16.427080	5.601754
H	7.898177	17.436609	5.993800
C	8.039526	16.206149	4.242149
H	8.203114	17.048428	3.581618
C	8.019636	14.916239	3.715585
C	7.271089	12.899712	6.912433
H	7.136833	12.006437	6.303627
C	5.961744	13.124013	7.681165
H	6.031746	13.982172	8.353570
H	5.725500	12.244792	8.283549
H	5.134417	13.296462	6.991989
C	8.441751	12.638474	7.871762

H	9.362866	12.430988	7.322685
H	8.226405	11.779944	8.512207
H	8.622635	13.503372	8.515309
C	8.237261	14.692863	2.227898
H	8.315266	13.620603	2.057947
C	9.548165	15.319596	1.733606
H	9.532532	16.408838	1.815924
H	9.708044	15.069795	0.681943
H	10.402988	14.950245	2.304375
C	7.032747	15.193617	1.420214
H	6.119042	14.696657	1.746325
H	7.175208	14.985892	0.356081
H	6.899376	16.271455	1.539548
C	6.912481	9.807436	1.932015
C	6.830117	10.104116	0.556190
C	6.336410	9.111332	-0.287390
H	6.256036	9.298161	-1.348855
C	5.950036	7.871208	0.207649
H	5.576389	7.113456	-0.470713
C	6.031715	7.606833	1.563455
H	5.715533	6.642191	1.938680
C	6.501631	8.572329	2.455756
C	7.284376	11.452475	0.009826
H	6.900969	12.213492	0.695468
C	6.732943	11.769236	-1.384463
H	7.214269	11.159372	-2.153702
H	6.932690	12.815259	-1.624999
H	5.658669	11.609022	-1.448017
C	8.818926	11.569860	-0.030895
H	9.272163	11.456990	0.951414
H	9.108193	12.550311	-0.417510
H	9.239924	10.808493	-0.692978
C	6.575362	8.263152	3.941490
H	6.735952	9.203229	4.468956
C	7.765089	7.340750	4.252659
H	7.649158	6.377453	3.749124
H	7.833294	7.155689	5.327444
H	8.708395	7.779452	3.923786
C	5.270286	7.662220	4.477881
H	4.416334	8.293463	4.241811
H	5.323511	7.565797	5.563740

H	5.081650	6.668347	4.065233
C	2.684575	14.584166	2.536130
C	2.795046	14.466804	1.145788
C	3.390514	15.520872	0.454240
H	3.500663	15.459391	-0.621208
C	3.853771	16.642433	1.126447
H	4.318828	17.449069	0.572836
C	3.738697	16.727606	2.506250
H	4.117027	17.601715	3.020951
C	3.151302	15.699334	3.241839
C	2.309504	13.234478	0.403412
H	1.840989	12.567485	1.125937
C	3.492323	12.476792	-0.208793
H	3.991830	13.086968	-0.964169
H	3.151003	11.555799	-0.688033
H	4.219090	12.219293	0.563178
C	1.249399	13.579950	-0.651701
H	0.398807	14.098396	-0.203492
H	0.880988	12.668690	-1.128833
H	1.659539	14.222736	-1.434017
C	3.038763	15.801220	4.751779
H	2.582799	14.881664	5.118739
C	2.132256	16.966663	5.173260
H	2.544738	17.925245	4.849851
H	2.032830	16.995045	6.261064
H	1.133286	16.868736	4.742075
C	4.425843	15.902084	5.393526
H	5.052489	15.068222	5.080169
H	4.345944	15.882124	6.482564
H	4.932118	16.827653	5.112993
C	1.555432	10.439806	4.939568
C	1.546609	10.233815	6.327319
C	1.535493	8.917877	6.783986
H	1.531979	8.718722	7.847363
C	1.551156	7.852972	5.891957
H	1.557724	6.836546	6.266418
C	1.564276	8.085412	4.525051
H	1.581812	7.246276	3.841147
C	1.556196	9.384076	4.015500
C	1.552664	11.405821	7.295624
H	1.945195	12.269622	6.755305

C	2.459093	11.179754	8.512086
H	2.062172	10.407696	9.175123
H	2.533120	12.101852	9.092478
H	3.464022	10.885722	8.210756
C	0.123583	11.746779	7.750052
H	-0.523773	11.979539	6.903497
H	0.130425	12.611675	8.417651
H	-0.317265	10.904058	8.288519
C	1.535135	9.612596	2.511443
H	1.475320	10.684910	2.332162
C	0.291133	8.980307	1.869556
H	0.301934	7.891932	1.963497
H	0.255204	9.221773	0.804714
H	-0.625890	9.349133	2.334531
C	2.823703	9.119137	1.841810
H	3.693798	9.638961	2.242642
H	2.788038	9.309647	0.767037
H	2.963820	8.046217	1.987422
N	4.305061	11.011395	5.359831
N	4.969664	10.454472	6.055475

Int1_2

134

B	5.593755	12.368962	3.737929
B	4.311648	11.848604	4.502575
C	7.043455	12.159141	3.694243
N	8.092682	12.898885	4.273437
C	9.327325	12.366770	3.887255
H	10.250189	12.796642	4.232697
C	9.097089	11.309546	3.093763
H	9.781626	10.637766	2.608627
N	7.718343	11.151016	2.977976
C	2.799381	12.112278	4.416115
N	2.181441	13.104332	3.703805
C	0.809329	13.075902	3.908314
H	0.157407	13.780919	3.426414
C	0.551152	12.058301	4.749778
H	-0.374874	11.680461	5.141933
N	1.768743	11.465408	5.060840
C	7.976273	14.169611	4.910248
C	7.624901	14.230312	6.267089
C	7.631779	15.477057	6.893341
H	7.383237	15.547295	7.944556
C	7.956104	16.627586	6.191775
H	7.955743	17.587344	6.694668
C	8.264134	16.553555	4.840756
H	8.487857	17.463004	4.298507
C	8.278037	15.330161	4.171464
C	7.288729	12.980160	7.058001
H	7.139500	12.168613	6.348487
C	5.986470	13.130662	7.850954
H	6.058642	13.909433	8.613787
H	5.751210	12.193712	8.354303
H	5.157853	13.375249	7.183948
C	8.456534	12.585985	7.975410
H	9.372001	12.430760	7.400262
H	8.226906	11.659327	8.507264
H	8.651990	13.365018	8.716907
C	8.539588	15.278509	2.674230
H	8.832999	14.261582	2.416583
C	9.681696	16.196682	2.223347
H	9.428462	17.253082	2.337735
H	9.896558	16.027169	1.165788
H	10.594734	16.005182	2.791545
C	7.241225	15.583684	1.913619
H	6.461681	14.865893	2.171699
H	7.407855	15.536677	0.834898
H	6.877847	16.584682	2.158624
C	7.076549	10.283079	2.047811
C	6.938838	10.706524	0.715926
C	6.330457	9.832939	-0.185918
H	6.217841	10.126500	-1.222079
C	5.864345	8.593604	0.227680
H	5.386407	7.931606	-0.484614
C	6.010571	8.195154	1.550019
H	5.645128	7.223380	1.853458
C	6.618431	9.029669	2.487316
C	7.495811	12.040515	0.242310
H	7.781126	12.612899	1.123099
C	6.465130	12.885748	-0.513023
H	6.088832	12.375148	-1.402408
H	6.919652	13.824303	-0.837935
H	5.622612	13.127543	0.132131

C	8.762452	11.821771	-0.600265
H	9.514567	11.258931	-0.043688
H	9.199576	12.781148	-0.888501
H	8.534609	11.265885	-1.513739
C	6.858524	8.575519	3.917674
H	6.919079	9.471310	4.534251
C	8.212843	7.851662	4.019283
H	8.212694	6.947705	3.404249
H	8.413471	7.560493	5.053497
H	9.031080	8.489922	3.683178
C	5.742134	7.699468	4.492272
H	4.773174	8.190503	4.429243
H	5.940074	7.499536	5.546646
H	5.674929	6.736255	3.980205
C	2.785702	14.003023	2.759040
C	2.730467	13.665630	1.402501
C	3.236683	14.592567	0.490561
H	3.220554	14.362683	-0.567344
C	3.766954	15.798431	0.921148
H	4.162481	16.502346	0.199211
C	3.807082	16.103439	2.275781
H	4.237537	17.042582	2.594965
C	3.323218	15.208636	3.225845
C	2.149511	12.348380	0.913651
H	1.829896	11.776229	1.783378
C	3.202115	11.499782	0.191436
H	3.561880	11.993616	-0.712813
H	2.776680	10.537466	-0.100734
H	4.058399	11.310283	0.837098
C	0.909119	12.572308	0.036938
H	0.145939	13.146205	0.567377
H	0.473408	11.613959	-0.256035
H	1.163107	13.116752	-0.875704
C	3.345466	15.534875	4.709014
H	3.329287	14.588093	5.249772
C	2.092871	16.331416	5.108243
H	2.061536	17.287931	4.580506
H	2.095875	16.535124	6.181982
H	1.178010	15.784823	4.870581
C	4.622114	16.259507	5.137234
H	5.506559	15.709735	4.820661
H	4.654390	16.347558	6.224390
H	4.679807	17.269555	4.725637
C	1.853716	10.175892	5.692160
C	2.077798	10.100836	7.074386
C	2.144791	8.832100	7.644722
H	2.326228	8.728209	8.705213
C	2.012577	7.689291	6.865868
H	2.086441	6.712216	7.327772
C	1.795385	7.793135	5.501277
H	1.703092	6.894193	4.905387
C	1.698097	9.040526	4.883500
C	2.254225	11.359505	7.909256
H	2.754611	12.097482	7.277477
C	3.134187	11.146868	9.145095
H	2.624905	10.548623	9.904339
H	3.374538	12.111167	9.595990
H	4.068346	10.649198	8.885017
C	0.893112	11.944420	8.323692
H	0.277950	12.194113	7.459177
H	1.034639	12.856031	8.909105
H	0.341681	11.227710	8.937424
C	1.440795	9.129487	3.386610
H	1.243351	10.170872	3.136889
C	0.194146	8.333975	2.973034
H	0.334509	7.260373	3.115376
H	-0.020089	8.501456	1.915110
H	-0.680846	8.638705	3.551187
C	2.671851	8.698023	2.579895
H	3.527125	9.339071	2.792173
H	2.467053	8.759774	1.509580
H	2.950969	7.667728	2.810075
N	4.718279	10.939774	5.558204
N	5.168874	10.280386	6.356818

TS2_2

136

B	-0.664071	0.370022	0.068510
B	0.891353	0.055770	-0.209585
C	-1.718153	1.382812	0.179334
N	-1.503343	2.622440	0.849454
C	-2.727065	3.264964	1.062373
H	-2.779885	4.203629	1.583525

C	-3.690883	2.539462	0.485244
H	-4.749706	2.710818	0.422952
N	-3.108965	1.413333	-0.106564
C	1.897215	-0.942286	0.372904
N	3.278780	-0.954275	0.264917
C	3.817560	-1.990791	1.018898
H	4.877128	-2.157968	1.064681
C	2.795573	-2.649743	1.585529
H	2.779006	-3.523904	2.209881
N	1.616558	-2.034137	1.172897
C	-0.288748	3.384004	0.823938
C	-0.042928	4.267129	-0.241533
C	1.092396	5.076910	-0.173401
H	1.304005	5.766326	-0.981011
C	1.943101	5.027672	0.918563
H	2.812269	5.673319	0.958708
C	1.682941	4.148124	1.960605
H	2.350680	4.121142	2.810401
C	0.578100	3.301784	1.924349
C	-0.999039	4.420379	-1.414284
H	-1.761839	3.649707	-1.331561
C	-0.307089	4.238078	-2.771330
H	0.499160	4.960251	-2.914022
H	-1.025974	4.384930	-3.579127
H	0.119009	3.240732	-2.866989
C	-1.719878	5.776924	-1.350848
H	-1.011747	6.602092	-1.459220
H	-2.241952	5.901127	-0.400689
H	-2.455119	5.854349	-2.155676
C	0.279581	2.354940	3.071182
H	-0.372669	1.577411	2.675934
C	-0.482101	3.085234	4.187891
H	-1.414519	3.510076	3.811829
H	0.120204	3.899550	4.599878
H	-0.726228	2.399156	5.002696
C	1.534704	1.667797	3.616414
H	2.056576	1.135441	2.820233
H	1.263192	0.946172	4.387746
H	2.230809	2.377886	4.067402
C	-3.885190	0.508693	-0.883386
C	-4.801934	-0.335005	-0.230947
C	-5.608178	-1.163904	-1.011816
H	-6.325325	-1.816783	-0.530148
C	-5.498245	-1.175811	-2.393457
H	-6.128297	-1.830749	-2.983087
C	-4.579020	-0.344172	-3.020498
H	-4.505668	-0.353385	-4.100625
C	-3.763097	0.511958	-2.286124
C	-4.926973	-0.371663	1.282973
H	-4.107369	0.215555	1.696224
C	-4.786768	-1.797459	1.830564
H	-5.629333	-2.427483	1.536426
H	-4.752068	-1.779167	2.922388
H	-3.871789	-2.264652	1.469900
C	-6.244643	0.262345	1.754758
H	-7.102240	-0.298045	1.373808
H	-6.340944	1.293294	1.410543
H	-6.299598	0.263314	2.846071
C	-2.801090	1.443141	-3.001934
H	-2.128050	1.860896	-2.257891
C	-3.575009	2.602211	-3.648005
H	-2.893288	3.285971	-4.156951
H	-4.128423	3.169095	-2.895973
H	-4.292044	2.230273	-4.384226
C	-1.918673	0.718213	-4.025860
H	-1.195001	1.416713	-4.451853
H	-2.502283	0.302387	-4.850198
H	-1.363780	-0.093696	-3.553715
C	4.111677	-0.129682	-0.567532
C	4.942307	0.824241	0.039879
C	5.794779	1.560906	-0.782750
H	6.449118	2.302552	-0.343605
C	5.814195	1.363325	-2.154421
H	6.479117	1.949934	-2.776525
C	4.982514	0.414503	-2.730899
H	5.009467	0.266462	-3.802313
C	4.119823	-0.355735	-1.954445
C	4.954715	1.058655	1.541213
H	4.085181	0.557363	1.966328
C	4.838362	2.547140	1.889867
H	3.991527	3.001129	1.379463
H	5.740400	3.096361	1.612070

H	4.696188	2.669563	2.965699
C	6.210934	0.453077	2.188445
H	7.111920	0.935016	1.801499
H	6.295834	-0.616622	1.991702
H	6.187959	0.597224	3.271238
C	3.269045	-1.431989	-2.607906
H	2.487831	-1.716420	-1.908406
C	4.124396	-2.677842	-2.888419
H	4.567683	-3.068090	-1.969800
H	4.935473	-2.441194	-3.581057
H	3.517321	-3.467870	-3.334966
C	2.554502	-0.951030	-3.877188
H	1.877743	-1.729881	-4.233654
H	3.256839	-0.730990	-4.683679
H	1.968389	-0.052994	-3.680724
C	0.386660	-2.786003	1.239134
C	0.224603	-3.846788	0.333555
C	-0.893266	-4.667663	0.491111
H	-1.054548	-5.484491	-0.200502
C	-1.801331	-4.449634	1.513503
H	-2.661951	-5.098292	1.621046
C	-1.614335	-3.394781	2.396918
H	-2.329897	-3.236627	3.190903
C	-0.523117	-2.537570	2.274901
C	1.215472	-4.143830	-0.784174
H	2.038166	-3.436137	-0.714235
C	0.586538	-3.962685	-2.172866
H	-0.288959	-4.601242	-2.302151
H	1.309737	-4.221838	-2.948466
H	0.276481	-2.930034	-2.330939
C	1.826410	-5.544943	-0.632381
H	1.071042	-6.325430	-0.745577
H	2.291701	-5.666541	0.347775
H	2.591149	-5.706828	-1.395342
C	-0.289770	-1.420677	3.273550
H	0.347525	-0.685484	2.783963
C	0.452962	-1.959945	4.506842
H	1.411656	-2.403013	4.230048
H	-0.141546	-2.726519	5.010168
H	0.645946	-1.158287	5.223292
C	-1.579776	-0.700377	3.673260
H	-1.349040	0.135775	4.334955
H	-2.269401	-1.355944	4.208767
H	-2.080438	-0.307181	2.787602
N	1.471089	1.045877	-1.081134
N	1.843178	1.878829	-1.744414
N	-1.811286	-1.306106	-0.978275
N	-2.092226	-2.333407	-1.241843

Int2_2

136

B	-0.842897	0.118736	-0.185684
B	0.842596	-0.118909	-0.185939
C	-1.713641	1.285677	0.245880
N	-1.334464	2.451551	0.909991
C	-2.455547	3.217067	1.228141
H	-2.360544	4.154540	1.744432
C	-3.533856	2.591084	0.737015
H	-4.573026	2.862021	0.752978
N	-3.098531	1.426413	0.113461
C	1.713562	-1.285709	0.245430
N	3.098472	-1.426326	0.112951
C	3.533804	-2.591147	0.736235
H	4.572966	-2.862039	0.752025
C	2.455565	-3.217258	1.227316
H	2.360630	-4.154842	1.743382
N	1.334454	-2.451710	0.909363
C	-0.042225	3.086348	0.941466
C	0.251882	4.038667	-0.047956
C	1.437752	4.763136	0.077246
H	1.694283	5.497060	-0.676070
C	2.291330	4.556916	1.147612
H	3.205335	5.132239	1.231075
C	1.980434	3.606445	2.110518
H	2.651940	3.458852	2.944410
C	0.815546	2.848016	2.023065
C	-0.676016	4.322493	-1.220190
H	-1.536932	3.663309	-1.141344
C	-0.006450	4.030938	-2.570083
H	0.884195	4.645155	-2.715922
H	-0.699790	4.247798	-3.385273
H	0.294479	2.986665	-2.644358
C	-1.212560	5.761266	-1.170387

H	-0.406730	6.490273	-1.281942
H	-1.718208	5.960099	-0.223343
H	-1.928297	5.925650	-1.979154
C	0.451171	1.849171	3.104528
H	-0.262611	1.152180	2.667944
C	-0.238194	2.563842	4.277563
H	-1.136843	3.087679	3.946354
H	0.433419	3.297884	4.730694
H	-0.528141	1.848238	5.050278
C	1.651290	1.028908	3.584818
H	2.120567	0.512540	2.747108
H	1.327698	0.281043	4.309972
H	2.404340	1.649101	4.074332
C	-3.996825	0.641853	-0.682700
C	-4.962241	-0.144511	-0.034270
C	-5.878481	-0.838857	-0.823469
H	-6.636077	-1.448681	-0.348306
C	-5.827080	-0.772314	-2.207277
H	-6.541983	-1.325650	-2.804121
C	-4.856187	0.000989	-2.826292
H	-4.824199	0.048903	-3.907247
C	-3.928598	0.729483	-2.083575
C	-5.030522	-0.262583	1.478893
H	-4.134728	0.201787	1.890677
C	-5.031098	-1.728463	1.929780
H	-5.959186	-2.233148	1.652549
H	-4.933055	-1.789148	3.016065
H	-4.202052	-2.271430	1.479243
C	-6.248080	0.482377	2.047654
H	-7.177222	0.047577	1.671408
H	-6.239726	1.538456	1.773083
H	-6.261064	0.416108	3.138234
C	-2.918573	1.607078	-2.803559
H	-2.217213	1.987727	-2.067328
C	-3.616816	2.813926	-3.448712
H	-2.884753	3.463768	-3.932869
H	-4.152951	3.402873	-2.701251
H	-4.336742	2.495172	-4.205963
C	-2.086195	0.825296	-3.828830
H	-1.329502	1.478909	-4.268061
H	-2.704741	0.438824	-4.641899

H	-1.576997	-0.013841	-3.355881
C	3.996775	-0.641465	-0.682894
C	4.962118	0.144710	-0.034177
C	5.878377	0.839291	-0.823129
H	6.635918	1.448984	-0.347718
C	5.827106	0.773149	-2.206954
H	6.542032	1.326663	-2.803556
C	4.856277	0.000001	-2.826243
H	4.824362	-0.047604	-3.907201
C	3.928624	-0.728704	-2.083795
C	5.030426	0.262200	1.479047
H	4.134511	-0.202061	1.890662
C	5.031485	1.727902	1.930500
H	4.202699	2.271345	1.480094
H	5.959796	2.232361	1.653595
H	4.933340	1.788185	3.016801
C	6.247797	-0.483318	2.047514
H	7.177049	-0.048641	1.671406
H	6.239150	-1.539303	1.772580
H	6.260821	-0.417459	3.138120
C	2.918756	-1.606257	-2.804048
H	2.216908	-1.986477	-2.068074
C	3.617122	-2.813496	-3.448364
H	4.152584	-3.402372	-2.700369
H	4.337658	-2.495118	-4.205200
H	2.885202	-3.463272	-3.932861
C	2.087115	-0.824657	-3.830053
H	1.330349	-1.478248	-4.269240
H	2.706124	-0.438841	-4.643071
H	1.578063	0.014927	-3.357732
C	0.042323	-3.086714	0.940634
C	-0.251704	-4.038620	-0.049200
C	-1.437465	-4.763299	0.075720
H	-1.693903	-5.496937	-0.677918
C	-2.290979	-4.557725	1.146262
H	-3.204903	-5.133200	1.229462
C	-1.980248	-3.607557	2.109507
H	-2.651735	-3.460455	2.943500
C	-0.815444	-2.848961	2.022353
C	0.676072	-4.321632	-1.221731
H	1.537152	-3.662726	-1.142429

C	0.006417	-4.028736	-2.571264
H	-0.884475	-4.642487	-2.717433
H	0.699487	-4.245127	-3.386805
H	-0.294148	-2.984301	-2.644633
C	1.212338	-5.760515	-1.173261
H	0.406453	-6.489304	-1.285417
H	1.718048	-5.960333	-0.226476
H	1.927997	-5.924366	-1.982218
C	-0.451244	-1.850473	3.104189
H	0.262589	-1.153339	2.667959
C	0.237962	-2.565509	4.277047
H	1.136678	-3.089204	3.945833
H	-0.433619	-3.299709	4.729855
H	0.527807	-1.850207	5.050089
C	-1.651429	-1.030387	3.584563
H	-1.327979	-0.282717	4.309999
H	-2.404531	-1.650717	4.073773
H	-2.120598	-0.513768	2.746973
N	1.588965	0.931729	-0.785000
N	2.119637	1.817705	-1.250855
N	-1.589614	-0.931858	-0.784395
N	-2.120502	-1.817852	-1.250015

RC$_{2LiCl}$

136

Li	1.780077	1.019999	-1.431417
Cl	3.305573	0.806298	-2.935053
B	0.495613	-0.339562	-0.063521
B	-0.360598	0.827626	-0.084266
C	1.354908	-1.562544	-0.109491
N	2.463852	-1.858545	0.652228
C	3.001905	-3.076963	0.264833
H	3.874199	-3.484695	0.740220
C	2.229884	-3.562412	-0.727495
H	2.294863	-4.477595	-1.286165
N	1.215641	-2.644363	-0.948072
C	-1.206116	2.055080	-0.105604
N	-0.807040	3.354166	-0.338227
C	-1.885760	4.220499	-0.256413
H	-1.773638	5.279228	-0.404184
C	-2.975694	3.475544	0.020074
H	-4.005349	3.755188	0.145528
N	-2.561944	2.154825	0.112747
C	2.974026	-1.100528	1.765108
C	2.316214	-1.200024	3.005900
C	2.895971	-0.550796	4.092845
H	2.420558	-0.598511	5.062128
C	4.080628	0.160886	3.957445
H	4.515730	0.651995	4.819557
C	4.694855	0.259373	2.721863
H	5.606979	0.832666	2.624454
C	4.150805	-0.358943	1.593798
C	1.039219	-2.011051	3.171613
H	0.455482	-1.885320	2.259832
C	0.154686	-1.522118	4.320828
H	-0.052489	-0.454849	4.235792
H	0.608156	-1.710156	5.296660
H	-0.796074	-2.056553	4.300349
C	1.347604	-3.507555	3.347950
H	1.915568	-3.908989	2.509504
H	0.419392	-4.078117	3.425684
H	1.925836	-3.673027	4.260650
C	4.841147	-0.217046	0.248739
H	4.145295	-0.516990	-0.534949
C	6.072246	-1.132746	0.155482
H	6.535116	-1.039061	-0.829251
H	5.810716	-2.181470	0.306819
H	6.814622	-0.860069	0.909951
C	5.235829	1.236514	-0.044770
H	6.051575	1.572468	0.600531
H	4.391458	1.911684	0.093359
H	5.554102	1.323577	-1.082829
C	0.055497	-2.951361	-1.743086
C	0.100091	-2.744667	-3.127791
C	-0.999745	-3.165126	-3.876099
H	-0.996031	-3.026888	-4.948955
C	-2.105057	-3.737637	-3.265732
H	-2.953073	-4.046594	-3.865150
C	-2.135631	-3.900514	-1.888648
H	-3.011698	-4.330826	-1.420558
C	-1.051575	-3.524520	-1.097107
C	1.296368	-2.101524	-3.802736
H	1.878323	-1.577321	-3.045297

C	0.886163	-1.050423	-4.840817
H	1.768228	-0.492765	-5.154281
H	0.177713	-0.339191	-4.416389
H	0.428911	-1.505218	-5.723091
C	2.212490	-3.159982	-4.436115
H	3.086534	-2.679971	-4.881432
H	1.685871	-3.710711	-5.220152
H	2.564103	-3.881776	-3.696234
C	-1.102485	-3.731539	0.407195
H	-0.119965	-3.501038	0.814606
C	-1.401442	-5.189726	0.782520
H	-0.675965	-5.871056	0.332772
H	-2.398098	-5.493830	0.456438
H	-1.356828	-5.312692	1.867305
C	-2.106009	-2.768361	1.054986
H	-1.869506	-1.734463	0.802743
H	-2.080507	-2.873778	2.141766
H	-3.123890	-2.977538	0.721488
C	0.542885	3.743398	-0.616505
C	0.912052	3.986264	-1.952516
C	2.242502	4.326482	-2.196219
H	2.578405	4.486746	-3.210258
C	3.154107	4.437416	-1.156086
H	4.185730	4.684998	-1.372017
C	2.754362	4.222568	0.154143
H	3.475048	4.323294	0.955610
C	1.439621	3.866758	0.455459
C	-0.086968	3.845029	-3.094290
H	-1.080472	4.059571	-2.696346
C	-0.109104	2.404655	-3.630646
H	-0.841786	2.309690	-4.436032
H	-0.382168	1.691954	-2.849654
H	0.871178	2.120302	-4.017795
C	0.159752	4.840384	-4.233880
H	1.076081	4.611063	-4.781210
H	0.232109	5.865066	-3.863508
H	-0.664883	4.791852	-4.947951
C	1.013879	3.661010	1.899243
H	-0.016897	3.307958	1.901136
C	1.049329	4.991998	2.667059
H	0.418541	5.744045	2.187366
H	2.065699	5.389395	2.717801
H	0.692587	4.848583	3.689749
C	1.855359	2.586592	2.596573
H	1.492048	2.425166	3.613881
H	2.905990	2.875099	2.663542
H	1.789095	1.640604	2.059458
C	-3.458500	1.042474	0.237534
C	-3.854400	0.633131	1.517864
C	-4.784120	-0.401559	1.604787
H	-5.119949	-0.742107	2.574536
C	-5.274963	-1.016291	0.461075
H	-5.989321	-1.825814	0.550248
C	-4.842833	-0.610688	-0.792181
H	-5.218183	-1.111328	-1.675668
C	-3.928662	0.433614	-0.933304
C	-3.305092	1.305119	2.764646
H	-2.352276	1.764171	2.493774
C	-3.021070	0.306757	3.891496
H	-3.942548	-0.090520	4.322288
H	-2.468544	0.796282	4.695780
H	-2.425122	-0.528622	3.528287
C	-4.246518	2.417974	3.253518
H	-3.830495	2.910539	4.135593
H	-5.220862	2.002638	3.523133
H	-4.405221	3.177152	2.486607
C	-3.472404	0.867875	-2.315312
H	-2.790843	1.709548	-2.200699
C	-4.649090	1.352607	-3.174702
H	-4.284825	1.712786	-4.139601
H	-5.187192	2.168514	-2.686651
H	-5.361858	0.547604	-3.367296
C	-2.692978	-0.255264	-3.007831
H	-2.322904	0.081618	-3.978368
H	-3.322143	-1.130552	-3.173910
H	-1.839536	-0.561298	-2.402035
N	-4.560983	-3.051660	4.194462
N	-3.582773	-3.498562	4.375906

TS1_2$_{LiCl}$

136

Li	1.894813	1.435514	-1.544436
Cl	3.313600	1.774987	-3.146776

B	0.713940	-0.071547	-0.252450
B	-0.637396	0.452212	0.356396
C	1.589946	-1.278090	-0.378702
N	2.760062	-1.554920	0.326580
C	3.362033	-2.714287	-0.157717
H	4.274488	-3.090655	0.265093
C	2.601313	-3.180091	-1.157540
H	2.722437	-4.038610	-1.791232
N	1.509758	-2.322054	-1.298735
C	-1.503988	1.741398	0.304702
N	-1.152647	3.052658	0.073677
C	-2.257741	3.888276	0.208322
H	-2.174049	4.952234	0.082847
C	-3.314238	3.110331	0.500794
H	-4.349322	3.351008	0.656425
N	-2.856369	1.802986	0.549874
C	3.277454	-0.848351	1.462967
C	2.772237	-1.156658	2.739145
C	3.382386	-0.561759	3.841773
H	3.023167	-0.781058	4.837496
C	4.447578	0.312868	3.685017
H	4.909185	0.763313	4.555703
C	4.913656	0.620479	2.418044
H	5.737145	1.312802	2.309402
C	4.340471	0.051194	1.279188
C	1.625300	-2.136865	2.926793
H	1.013528	-2.095537	2.026603
C	0.719054	-1.777556	4.109566
H	0.370538	-0.745434	4.042411
H	1.231101	-1.903967	5.065682
H	-0.154379	-2.430457	4.116021
C	2.144397	-3.577449	3.069528
H	2.734020	-3.877890	2.203135
H	1.307753	-4.274117	3.163772
H	2.770822	-3.673221	3.959905
C	4.895486	0.384928	-0.094926
H	4.127344	0.167872	-0.838826
C	6.116052	-0.487110	-0.432845
H	6.483910	-0.240120	-1.430915
H	5.874116	-1.549969	-0.418127
H	6.923114	-0.312239	0.283693
C	5.261038	1.867593	-0.235511
H	6.153485	2.118535	0.343131
H	4.445879	2.510041	0.098200
H	5.450494	2.096643	-1.282878
C	0.424178	-2.653136	-2.181469
C	0.460784	-2.192488	-3.507865
C	-0.510221	-2.668100	-4.389200
H	-0.506210	-2.334386	-5.417441
C	-1.481372	-3.563355	-3.969734
H	-2.222611	-3.925842	-4.672173
C	-1.518338	-3.980157	-2.648755
H	-2.295300	-4.662249	-2.332302
C	-0.574865	-3.531194	-1.724559
C	1.529653	-1.227840	-3.985361
H	1.834614	-0.619782	-3.133795
C	1.027084	-0.267544	-5.068430
H	1.755357	0.532434	-5.196652
H	0.072173	0.181049	-4.791528
H	0.899341	-0.772925	-6.029072
C	2.784742	-1.962937	-4.481319
H	3.542329	-1.233146	-4.771929
H	2.548353	-2.588216	-5.346746
H	3.213949	-2.599244	-3.707292
C	-0.636395	-4.014902	-0.284106
H	-0.126595	-3.275185	0.333996
C	0.097473	-5.354900	-0.107641
H	1.146343	-5.280417	-0.390759
H	-0.369829	-6.128915	-0.722056
H	0.053593	-5.676051	0.935930
C	-2.076421	-4.138390	0.232499
H	-2.655994	-3.236133	0.035458
H	-2.069382	-4.303955	1.310634
H	-2.594148	-4.983133	-0.226984
C	0.164523	3.583421	-0.144293
C	0.504677	4.022260	-1.439080
C	1.783455	4.550450	-1.617107
H	2.107030	4.856241	-2.600066
C	2.666063	4.662630	-0.550410
H	3.660142	5.056245	-0.718644
C	2.284414	4.264830	0.720951
H	2.977738	4.373246	1.545108

C	1.021324	3.720271	0.955819
C	-0.476088	3.901798	-2.601371
H	-1.478862	4.072379	-2.204600
C	-0.454640	2.487780	-3.203602
H	-1.220229	2.390827	-3.977174
H	-0.640878	1.718032	-2.451031
H	0.516140	2.289243	-3.662121
C	-0.247453	4.946963	-3.698313
H	0.682169	4.764359	-4.240374
H	-0.212889	5.959156	-3.290301
H	-1.061792	4.898202	-4.424083
C	0.607110	3.336669	2.365666
H	-0.365867	2.849682	2.315162
C	0.449213	4.587474	3.244329
H	-0.275551	5.284392	2.816806
H	1.399928	5.114806	3.352170
H	0.104914	4.307294	4.242786
C	1.578105	2.328814	2.988140
H	1.214598	2.009513	3.967358
H	2.572718	2.755664	3.127844
H	1.673180	1.446690	2.357241
C	-3.745379	0.680795	0.644403
C	-4.234560	0.309068	1.904694
C	-5.108826	-0.774300	1.959945
H	-5.508344	-1.091764	2.914049
C	-5.460952	-1.466352	0.808952
H	-6.128013	-2.317142	0.874933
C	-4.966249	-1.071038	-0.425609
H	-5.257782	-1.613277	-1.315842
C	-4.109160	0.022827	-0.540225
C	-3.836544	1.055672	3.168467
H	-2.953800	1.654352	2.935870
C	-3.457144	0.120530	4.324831
H	-4.318060	-0.448073	4.681432
H	-3.081516	0.707352	5.165842
H	-2.682954	-0.587556	4.030021
C	-4.952620	2.019745	3.603879
H	-4.646503	2.585836	4.486613
H	-5.860440	1.465155	3.854288
H	-5.202041	2.730352	2.815011
C	-3.639440	0.484860	-1.910006
H	-2.963436	1.327253	-1.774290
C	-4.825063	0.988349	-2.749067
H	-4.468773	1.377760	-3.705399
H	-5.364237	1.786860	-2.234484
H	-5.533764	0.183719	-2.957360
C	-2.856089	-0.604850	-2.647997
H	-2.501377	-0.228486	-3.608977
H	-3.473760	-1.482842	-2.842653
H	-1.986393	-0.920772	-2.072527
N	-1.348372	-0.638234	1.270273
N	-1.513547	-1.569098	1.885470

Int1_2$_{LiCl}$

Li	2.115394	0.923899	-1.639046
Cl	3.511444	1.010809	-3.303742
B	0.679442	-0.204075	-0.220209
B	-0.674985	0.411567	0.360185
C	1.231895	-1.599740	-0.043691
N	2.270085	-1.976237	0.795002
C	2.603324	-3.312732	0.596728
H	3.390244	-3.783548	1.155975
C	1.799646	-3.789669	-0.363435
H	1.760343	-4.754365	-0.833009
N	0.944006	-2.757920	-0.755235
C	-1.433407	1.752771	0.221491
N	-0.937635	2.983414	-0.108270
C	-1.940565	3.942630	-0.079305
H	-1.736023	4.974261	-0.300149
C	-3.082661	3.314307	0.256614
H	-4.086659	3.679789	0.367047
N	-2.770714	1.975607	0.437182
C	2.890410	-1.190627	1.825255
C	2.372839	-1.263735	3.128237
C	3.078945	-0.623262	4.146912
H	2.712479	-0.672140	5.163696
C	4.240841	0.080396	3.875300
H	4.774770	0.573642	4.678685
C	4.716299	0.165503	2.574904
H	5.617679	0.728612	2.377437
C	4.058256	-0.470527	1.522751
C	1.106131	-2.035814	3.451766
H	0.581896	-2.227104	2.516499

C	0.152867	-1.236827	4.348816
H	-0.071510	-0.263151	3.911951
H	0.572971	-1.076488	5.344009
H	-0.786374	-1.778052	4.466397
C	1.437318	-3.398335	4.080431
H	2.067431	-3.996044	3.419738
H	0.520743	-3.960609	4.274505
H	1.965630	-3.270272	5.028703
C	4.625864	-0.417269	0.114300
H	3.796356	-0.544494	-0.585140
C	5.607823	-1.573792	-0.137186
H	5.982607	-1.525702	-1.161589
H	5.134402	-2.545693	0.002397
H	6.459828	-1.507735	0.544566
C	5.301235	0.920687	-0.206605
H	6.246873	1.031000	0.329562
H	4.659838	1.762874	0.056427
H	5.501045	0.979171	-1.275692
C	-0.120188	-3.024637	-1.689972
C	0.038358	-2.655874	-3.039171
C	-0.932972	-3.078756	-3.945277
H	-0.841839	-2.810491	-4.987669
C	-2.013483	-3.847385	-3.538919
H	-2.748728	-4.173239	-4.264871
C	-2.159518	-4.183943	-2.205014
H	-3.014534	-4.770081	-1.895866
C	-1.225478	-3.776621	-1.250453
C	1.234265	-1.845844	-3.508378
H	1.432105	-1.096452	-2.739055
C	0.985450	-1.093229	-4.818760
H	1.804375	-0.394211	-4.982900
H	0.052895	-0.527357	-4.788209
H	0.942188	-1.775175	-5.671927
C	2.503853	-2.702210	-3.640995
H	3.339503	-2.059726	-3.923015
H	2.369598	-3.467471	-4.410587
H	2.765018	-3.199560	-2.707342
C	-1.434303	-4.179061	0.201608
H	-0.795206	-3.549704	0.821033
C	-1.032434	-5.644014	0.444743
H	0.011996	-5.828707	0.196090
H	-1.648033	-6.314375	-0.160284
H	-1.179356	-5.905378	1.495481
C	-2.885107	-3.960766	0.658228
H	-3.243358	-2.965248	0.401495
H	-2.953060	-4.070293	1.741973
H	-3.557967	-4.694603	0.208747
C	0.435605	3.322052	-0.354884
C	0.838230	3.549404	-1.683806
C	2.176000	3.883913	-1.892960
H	2.545800	4.023847	-2.896760
C	3.057797	4.007290	-0.825199
H	4.095975	4.245976	-1.016956
C	2.616812	3.821500	0.475103
H	3.311289	3.940187	1.296948
C	1.290145	3.479586	0.741624
C	-0.140403	3.412532	-2.845925
H	-1.120888	3.734176	-2.489427
C	-0.272442	1.948849	-3.296355
H	-1.044765	1.855747	-4.063821
H	-0.534750	1.286931	-2.469514
H	0.670211	1.602349	-3.725201
C	0.214118	4.302934	-4.041277
H	1.122780	3.961443	-4.540360
H	0.358143	5.343160	-3.741928
H	-0.593261	4.267248	-4.775677
C	0.813075	3.331163	2.175572
H	-0.213801	2.967286	2.158194
C	0.806004	4.693501	2.886738
H	0.181894	5.415576	2.355441
H	1.815225	5.106350	2.952393
H	0.418030	4.587887	3.902654
C	1.639569	2.299079	2.946939
H	1.224702	2.151136	3.946540
H	2.677237	2.615490	3.063966
H	1.635952	1.340492	2.431170
C	-3.770987	0.961292	0.624105
C	-4.244369	0.707591	1.918672
C	-5.220853	-0.275675	2.062746
H	-5.612165	-0.502145	3.045421
C	-5.683729	-0.987545	0.964101
H	-6.429581	-1.761017	1.099622

C	-5.191806	-0.717896	-0.304270
H	-5.559882	-1.281935	-1.151508
C	-4.230353	0.272544	-0.506497
C	-3.719409	1.472695	3.123076
H	-2.772259	1.933260	2.834415
C	-3.431243	0.567082	4.327573
H	-4.349323	0.153720	4.750347
H	-2.939055	1.142608	5.114250
H	-2.779651	-0.261326	4.051589
C	-4.686708	2.602574	3.512968
H	-4.288075	3.174470	4.354109
H	-5.654761	2.191440	3.809159
H	-4.855361	3.292075	2.683980
C	-3.733096	0.579214	-1.909422
H	-2.992034	1.375237	-1.845551
C	-4.874241	1.096101	-2.799111
H	-4.487308	1.370205	-3.783121
H	-5.350831	1.976276	-2.361841
H	-5.642792	0.333292	-2.942647
C	-3.038881	-0.632400	-2.539440
H	-2.649934	-0.374268	-3.525846
H	-3.726748	-1.470136	-2.660937
H	-2.201934	-0.968252	-1.927711
N	-1.363801	-0.536916	1.201520
N	-1.786779	-1.344139	1.862218

XYZ coordinates for Figure S3:

In Gas Phase

TS_{1a}

108

C	-4.045918	-2.413141	-2.167472
C	-4.739266	-1.160171	-1.593034
C	-2.393173	-0.917092	-1.262271
C	-2.519945	-2.127221	-2.196897
H	-4.430755	-2.672231	-3.154933
H	-4.244979	-3.260186	-1.507986
N	-3.649421	-0.594945	-0.759301
C	-5.142982	-0.184398	-2.714421
H	-5.833612	-0.670221	-3.407941
H	-5.640669	0.693309	-2.303319
H	-4.271098	0.147325	-3.277602
C	-2.019338	-1.810484	-3.616926
H	-0.959581	-1.549732	-3.596334
H	-2.144804	-2.679857	-4.269206
H	-2.559333	-0.971715	-4.056834
C	-1.729846	-3.333890	-1.656801
H	-1.898065	-4.212118	-2.288528
H	-0.662468	-3.115524	-1.629537
H	-2.040353	-3.575675	-0.639378
C	-5.982195	-1.506466	-0.775426
H	-6.411826	-0.616511	-0.312523
H	-6.735968	-1.942574	-1.433483
H	-5.760915	-2.226376	0.009822
C	-3.860513	0.123125	0.452133
C	-3.999675	1.522733	0.440800
C	-3.939580	-0.596998	1.665633
C	-4.338797	2.171226	1.630378
C	-4.284619	0.093219	2.825433
C	-4.507747	1.462969	2.807461
H	-4.462518	3.247343	1.634007
H	-4.369975	-0.444052	3.761423
H	-4.783049	1.980043	3.718914
C	-3.716180	2.347512	-0.803141
H	-3.531458	1.655200	-1.621849
C	-3.592407	-2.075166	1.739266
H	-3.634067	-2.471909	0.726810
C	-2.432551	3.174354	-0.615400
H	-2.196536	3.722504	-1.530910
H	-2.547627	3.901998	0.192358
H	-1.584708	2.531735	-0.376703
C	-4.890668	3.253606	-1.195445
H	-4.685139	3.752758	-2.145382
H	-5.817179	2.687001	-1.302512
H	-5.063763	4.028959	-0.445896
C	-2.147451	-2.257933	2.233837
H	-1.434138	-1.761777	1.575747
H	-2.026994	-1.853227	3.241249
H	-1.888442	-3.319248	2.266041
C	-4.567000	-2.889856	2.599094
H	-5.604110	-2.731843	2.296568
H	-4.342822	-3.955098	2.508200
H	-4.486754	-2.632665	3.657675
B	1.131339	0.007469	0.025391

B	-1.136665	-0.260419	-1.011193
C	2.233416	0.788218	0.705767
C	2.960272	1.945898	0.061753
C	4.221192	2.080817	0.949288
C	3.919704	1.377002	2.289850
H	4.505358	3.122186	1.102847
H	5.059594	1.582169	0.460836
N	2.732885	0.528248	1.914691
C	2.089337	-0.385014	2.827557
C	1.016126	0.088393	3.607948
C	2.502524	-1.727933	2.879585
C	0.440842	-0.788137	4.525566
C	1.891188	-2.560330	3.817929
C	0.886107	-2.094405	4.647780
H	-0.387179	-0.449242	5.134260
H	2.191621	-3.598248	3.879155
H	0.422792	-2.760048	5.365587
C	3.319041	1.656364	-1.402623
H	3.909851	0.742557	-1.487149
H	2.417513	1.532092	-2.003467
H	3.901568	2.484014	-1.815548
C	2.056125	3.196903	0.120165
H	1.129514	3.020885	-0.427411
H	1.797375	3.464520	1.144836
H	2.572003	4.047428	-0.332982
C	5.088377	0.509083	2.756231
H	5.942525	1.149851	2.982218
H	4.829883	-0.041550	3.661312
H	5.391230	-0.200837	1.989526
C	3.575613	2.364995	3.411627
H	3.223882	1.836570	4.297743
H	4.475249	2.919248	3.683369
H	2.817093	3.084018	3.112139
C	3.498837	-2.334564	1.903761
H	3.879045	-1.536351	1.267163
C	0.394632	1.462306	3.411679
H	1.048167	2.043460	2.768949
C	-0.947751	1.334282	2.679357
H	-1.366606	2.320614	2.474124
H	-0.841141	0.811167	1.730270
H	-1.671611	0.784779	3.279017
C	0.223228	2.242685	4.721141
H	1.162257	2.329305	5.269600
H	-0.142169	3.250257	4.509720
H	-0.505576	1.765938	5.379303
C	2.799203	-3.346030	0.979517
H	2.412777	-4.194469	1.549286
H	1.967004	-2.887798	0.444233
H	3.508773	-3.732170	0.243418
C	4.691221	-2.996287	2.610502
H	5.417779	-3.345848	1.873641
H	5.198770	-2.312011	3.290720
H	4.371479	-3.863787	3.191389
N	0.034113	0.375678	-0.908593
N	0.686108	-1.224643	-0.306470

Int$_{1a}$

108

C	-4.898632	-0.609675	-2.700196
C	-5.232696	-0.623672	-1.191689
C	-2.940907	0.013439	-1.499523
C	-3.562375	0.154359	-2.874179
H	-5.705107	-0.168238	-3.286703
H	-4.767566	-1.636827	-3.045034
N	-3.882181	-0.375291	-0.599019
C	-6.244212	0.469255	-0.813650
H	-7.202341	0.252602	-1.289914
H	-6.403890	0.494413	0.263777
H	-5.924719	1.456359	-1.140401
C	-3.782488	1.643633	-3.210200
H	-2.824037	2.159624	-3.280312
H	-4.300976	1.743238	-4.168124
H	-4.374901	2.148136	-2.447075
C	-2.681932	-0.480554	-3.958449
H	-3.172850	-0.418416	-4.933426
H	-1.719797	0.030600	-4.026177
H	-2.483215	-1.530094	-3.736144
C	-5.805240	-1.970273	-0.742478
H	-5.912646	-2.007437	0.342712
H	-6.793430	-2.109136	-1.184937
H	-5.176290	-2.799643	-1.055805
C	-3.517830	-0.573580	0.776255
C	-3.617469	0.502517	1.680107

C	-2.957754	-1.806582	1.166446
C	-3.226853	0.291735	3.001786
C	-2.580887	-1.962371	2.500468
C	-2.725041	-0.932028	3.414963
H	-3.297388	1.105451	3.712603
H	-2.147838	-2.900943	2.821214
H	-2.423984	-1.075072	4.445760
C	-4.050558	1.897068	1.255058
H	-4.379877	1.843407	0.222275
C	-2.688664	-2.942818	0.191919
H	-3.085446	-2.652561	-0.779285
C	-2.864808	2.873254	1.281143
H	-3.183362	3.864206	0.947509
H	-2.452944	2.971657	2.287791
H	-2.071210	2.528571	0.619827
C	-5.216162	2.434118	2.096807
H	-5.567446	3.386325	1.693046
H	-6.058142	1.740200	2.112268
H	-4.913724	2.607799	3.131646
C	-1.180524	-3.170467	0.007654
H	-0.658217	-2.282617	-0.352680
H	-0.714120	-3.473571	0.947600
H	-1.009957	-3.969533	-0.717825
C	-3.385805	-4.243306	0.620984
H	-4.453966	-4.098914	0.791596
H	-3.261260	-5.007894	-0.149333
H	-2.953042	-4.636370	1.543081
B	1.112310	0.161982	-0.648624
B	-1.532005	0.262582	-1.161890
C	2.050302	1.215755	0.062450
C	1.633970	2.579358	0.565297
C	2.971107	3.250548	0.947415
C	4.016960	2.126603	1.063091
H	2.894176	3.819623	1.874171
H	3.269242	3.946133	0.161543
N	3.302797	0.983518	0.362445
C	3.987652	-0.273334	0.122018
C	3.946615	-1.275292	1.109841
C	4.722273	-0.430605	-1.066060
C	4.757792	-2.393027	0.928006
C	5.511327	-1.571849	-1.193436

C	5.553174	-2.533383	-0.197566
H	4.742116	-3.182096	1.668835
H	6.083164	-1.720526	-2.100078
H	6.176801	-3.411026	-0.315523
C	0.882118	3.387966	-0.500770
H	1.485991	3.494801	-1.404383
H	-0.051868	2.902961	-0.775257
H	0.666491	4.387297	-0.114237
C	0.725098	2.346829	1.791616
H	-0.130407	1.729330	1.524339
H	1.262631	1.848759	2.599437
H	0.361947	3.307087	2.164616
C	5.320738	2.484553	0.353133
H	5.783671	3.323480	0.875317
H	6.020635	1.648994	0.361519
H	5.146365	2.783502	-0.678227
C	4.332962	1.757886	2.513548
H	5.011434	0.907270	2.556221
H	4.821731	2.606566	2.994175
H	3.436906	1.516347	3.080985
C	4.589293	0.506352	-2.257016
H	4.012706	1.378158	-1.945884
C	2.965737	-1.268187	2.273837
H	2.459526	-0.304437	2.291336
C	1.879045	-2.333249	2.039749
H	1.090252	-2.236513	2.791172
H	1.444368	-2.214748	1.048570
H	2.301066	-3.338433	2.118446
C	3.631621	-1.479946	3.640906
H	4.412162	-0.746843	3.843632
H	2.883656	-1.406802	4.434286
H	4.082024	-2.471869	3.710777
C	3.783744	-0.184179	-3.373815
H	4.341942	-1.031175	-3.780890
H	2.832719	-0.551951	-2.987847
H	3.596670	0.519280	-4.189685
C	5.940370	0.998367	-2.796538
H	5.781129	1.727922	-3.593841
H	6.550031	1.465936	-2.022467
H	6.519448	0.176067	-3.221154
N	-0.311426	0.473254	-0.891544

N	1.462284	-1.073606	-1.034423

Pdt$_a$

108

C	-1.934871	-0.039015	-6.197104
N	-1.383125	-0.683173	-5.241711
B	-1.183981	-0.971001	-3.963355
N	-0.882037	-1.305884	-2.752138
B	-0.576316	-1.623544	-1.500796
C	1.419745	1.634044	-0.509358
C	-0.190448	-1.977253	-0.182090
H	2.511529	1.602705	-0.552242
C	2.220056	-2.683397	0.114808
H	2.397537	-1.764549	0.670966
H	2.879169	-3.459224	0.518643
H	2.502581	-2.497100	-0.922762
C	-1.544331	-0.220294	-7.656501
C	-0.142523	0.373782	-7.880050
H	-0.782924	-2.241769	-7.444793
H	-0.102995	1.433663	-7.626671
H	0.589826	-0.146923	-7.262163
H	0.144969	0.265111	-8.928180
C	-1.533236	-1.707064	-8.027195
H	-1.303018	-1.827026	-9.088440
H	-2.503815	-2.168301	-7.832810
C	-2.649611	0.554895	-8.399608
H	-2.269500	1.083872	-9.273682
H	-3.410316	-0.145364	-8.748824
H	-2.962266	3.359274	-8.482101
H	-1.629574	2.955711	-7.398585
C	-2.713207	2.945116	-7.503258
C	-3.290288	1.529103	-7.385032
H	-3.138583	3.598701	-6.743441
H	-4.596958	-0.836725	-6.135618
C	-4.806148	1.611789	-7.565246
H	-5.266152	0.626819	-7.561644
H	-5.025365	2.081490	-8.525747
H	-5.266667	2.213434	-6.780704
H	-6.904588	-1.613353	-6.139939
H	-6.927464	0.152740	-6.027839
C	-6.638379	-0.762264	-5.509186
H	-7.238611	-0.831112	-4.600247
C	-5.136661	-0.787923	-5.191053
C	-4.776007	-2.057454	-4.400918
C	-3.544514	1.204121	-4.829506
H	-5.061898	-2.948765	-4.964900
N	-2.912075	0.895584	-6.082387
H	-3.706592	-2.109196	-4.196997
H	-1.335019	2.583869	-5.242556
C	-1.705332	2.920239	-4.276162
H	-0.906984	4.908210	-4.624332
C	-1.851463	4.448263	-4.325355
H	-5.297806	-2.080532	-3.442808
C	-4.670153	0.450109	-4.439946
H	-2.115293	4.849190	-3.344627
C	-3.023975	2.216082	-4.002381
C	-3.714181	2.526323	-2.830447
H	-3.324822	3.289547	-2.169914
C	-4.867270	1.850266	-2.476131
H	-6.193668	0.245781	-2.947547
H	-5.384097	2.106106	-1.560217
C	-5.326993	0.809870	-3.265981
C	-0.659162	2.523818	-3.222656
H	0.299013	3.002218	-3.439192
H	-0.502516	1.446732	-3.196317
H	-4.875172	-0.689914	-0.883934
C	-4.120836	-1.466486	-0.746253
H	-4.605127	-2.435918	-0.889543
H	-0.969465	2.830575	-2.222504
C	-3.495711	-1.390165	0.655028
C	-2.700676	-0.104394	0.822568
H	-2.794337	-2.218167	0.733558
H	-4.456792	1.118361	0.689169
C	-3.377238	1.114764	0.774014
C	-2.694068	2.319052	0.807373
H	-3.238304	3.255755	0.769880
H	-5.355184	-0.793253	1.629553
C	-4.582288	-1.559838	1.726092
H	-4.173866	-1.493707	2.734230
H	-5.070985	-2.531717	1.619437
H	-0.775705	3.262678	0.843137
C	-1.308642	2.319310	0.858635
C	-0.588741	1.126239	0.917617

H	1.104058	2.657984	-0.723159
C	-1.293354	-0.098767	0.950424
H	1.029268	0.982727	-1.291240
C	0.742543	-3.120323	0.191173
C	0.552436	-4.358875	-0.690834
H	1.201453	-5.175313	-0.360751
H	0.802950	-4.136546	-1.731515
H	-0.483246	-4.700602	-0.662110
C	0.928462	1.174899	0.871895
H	1.282851	0.157971	1.007557
C	1.531812	2.046074	1.981136
H	2.622248	1.975910	1.966685
H	1.185734	1.736859	2.968211
H	1.269667	3.098917	1.851832
H	-2.214162	-2.923106	2.621895
H	0.432052	-0.375121	3.418211
C	-1.456692	-2.335918	3.137180
H	-1.906205	-1.394836	3.459849
H	-1.153981	-2.886729	4.030542
C	0.810668	-1.349282	3.106572
C	-0.236303	-2.076905	2.242090
H	1.748883	-1.198263	2.577489
H	1.023390	-1.930233	4.007043
C	0.307484	-3.398038	1.651645
H	1.120349	-3.806206	2.255371
N	-0.572949	-1.323294	1.012682
H	-2.622044	4.765220	-5.028722
H	-3.369794	-1.343626	-1.524968
H	-0.496164	-4.138128	1.641381

TS_{2b}

108

C	-4.993561	-0.754032	-2.901183
C	-5.151717	-0.042959	-1.537063
C	-2.828829	-0.264558	-2.078682
C	-3.552674	-0.498268	-3.395510
H	-5.742733	-0.414353	-3.617313
H	-5.133608	-1.827853	-2.764883
N	-3.724099	0.188217	-1.149113
C	-5.954844	1.259528	-1.642687
H	-6.997279	1.023350	-1.863133
H	-5.927127	1.806008	-0.699936
H	-5.588458	1.910968	-2.433062
C	-3.465403	0.768784	-4.274527
H	-2.430392	0.958237	-4.561922
H	-4.058966	0.639808	-5.183891
H	-3.833029	1.651325	-3.753306
C	-2.997219	-1.686416	-4.185521
H	-3.591979	-1.855018	-5.086622
H	-1.964950	-1.504092	-4.491184
H	-3.010788	-2.597546	-3.585389
C	-5.857456	-0.949882	-0.523534
H	-5.910513	-0.473934	0.456524
H	-6.877416	-1.146554	-0.859646
H	-5.344938	-1.904534	-0.420884
C	-3.319259	0.759735	0.103370
C	-3.180522	2.161715	0.195838
C	-3.027937	-0.065024	1.209502
C	-2.854907	2.718893	1.430900
C	-2.706248	0.544988	2.423412
C	-2.639683	1.922135	2.543875
H	-2.749542	3.792741	1.518162
H	-2.486882	-0.075307	3.283318
H	-2.389823	2.374588	3.495827
C	-3.286195	3.072300	-1.017244
H	-3.702721	2.488656	-1.830599
C	-2.968101	-1.582387	1.123107
H	-3.245116	-1.871339	0.110664
C	-1.892446	3.522977	-1.478431
H	-1.973438	4.158932	-2.363562
H	-1.379757	4.089637	-0.698377
H	-1.273610	2.664449	-1.733264
C	-4.203936	4.279714	-0.791335
H	-4.335757	4.830694	-1.725569
H	-5.188952	3.975595	-0.435166
H	-3.785807	4.976066	-0.061369
C	-1.532318	-2.081588	1.354709
H	-0.825094	-1.596522	0.683926
H	-1.210191	-1.892815	2.381370
H	-1.477640	-3.157831	1.180633
C	-3.926841	-2.271883	2.106059
H	-4.954540	-1.930162	1.985965
H	-3.907723	-3.353713	1.953715

H	-3.633552	-2.081274	3.140600
B	1.464666	-0.767964	-1.055652
B	-1.419653	-0.559882	-1.903666
C	2.164815	0.179866	-0.252924
C	1.806683	1.642605	-0.033808
C	3.014710	2.142057	0.801545
C	3.667756	0.902156	1.453615
H	2.718160	2.883884	1.543568
H	3.742926	2.614259	0.138418
N	3.281765	-0.155894	0.493297
C	4.033430	-1.332118	0.196893
C	3.809062	-2.519992	0.918165
C	4.971114	-1.298772	-0.859990
C	4.612186	-3.628697	0.644062
C	5.737777	-2.435561	-1.103421
C	5.580036	-3.585803	-0.344404
H	4.462764	-4.544901	1.202092
H	6.465407	-2.425000	-1.905381
H	6.195688	-4.455433	-0.540645
C	1.706841	2.390390	-1.373231
H	2.639948	2.308664	-1.932408
H	0.909835	1.973204	-1.991615
H	1.487996	3.449700	-1.210955
C	0.479851	1.790865	0.727969
H	-0.343092	1.359483	0.159897
H	0.507210	1.284145	1.690189
H	0.251905	2.844438	0.906277
C	5.185356	1.038889	1.565924
H	5.429917	1.831634	2.275284
H	5.635982	0.112522	1.926215
H	5.637576	1.287880	0.608416
C	3.096261	0.633256	2.857268
H	3.574964	-0.234840	3.308411
H	3.277178	1.492964	3.507127
H	2.023359	0.451308	2.824032
C	5.126209	-0.092084	-1.772407
H	4.566796	0.731276	-1.331457
C	2.676613	-2.662196	1.920498
H	2.172427	-1.700005	1.988363
C	1.639918	-3.681317	1.419003
H	0.798746	-3.736046	2.113180
H	1.257078	-3.397000	0.438730
H	2.074507	-4.680316	1.339264
C	3.174785	-3.045552	3.320855
H	3.941233	-2.356703	3.680417
H	2.348269	-3.036510	4.035462
H	3.606252	-4.048945	3.326813
C	4.503110	-0.377501	-3.149405
H	5.035902	-1.186130	-3.655633
H	3.456765	-0.665759	-3.054426
H	4.559729	0.511537	-3.782901
C	6.585449	0.360515	-1.924971
H	6.631654	1.292891	-2.492716
H	7.062518	0.525171	-0.957088
H	7.180678	-0.378427	-2.465135
N	-0.232547	-1.014453	-1.827314
N	0.927235	-1.650643	-1.951488

Pdt_b

108

C	-5.615112	-1.338586	-2.193091
C	-5.475253	-1.246899	-0.657125
C	-3.512601	-0.335038	-1.714932
C	-4.554289	-0.399309	-2.816913
H	-6.626433	-1.091374	-2.518886
H	-5.410156	-2.363550	-2.508132
N	-4.095647	-0.702779	-0.520480
C	-6.527313	-0.323409	-0.023635
H	-7.518764	-0.767750	-0.131319
H	-6.330428	-0.197055	1.041491
H	-6.547330	0.659201	-0.489056
C	-5.130218	1.004938	-3.095366
H	-4.352290	1.662279	-3.486759
H	-5.937945	0.946948	-3.831427
H	-5.528990	1.466079	-2.192693
C	-3.989354	-0.970718	-4.121876
H	-4.774789	-1.052632	-4.878199
H	-3.204018	-0.324006	-4.520076
H	-3.557821	-1.959635	-3.959819
C	-5.608218	-2.626718	-0.003684
H	-5.420451	-2.570051	1.069552
H	-6.622039	-3.006044	-0.149544
H	-4.913876	-3.342140	-0.440336

C	-3.412032	-0.539081	0.726806
C	-3.515241	0.686839	1.418859
C	-2.590318	-1.572938	1.220728
C	-2.902010	0.800407	2.665627
C	-1.991671	-1.406738	2.469659
C	-2.165792	-0.244417	3.201233
H	-2.980783	1.731007	3.213052
H	-1.356797	-2.190752	2.861098
H	-1.697157	-0.137757	4.172207
C	-4.173601	1.913935	0.809099
H	-4.665235	1.603146	-0.108034
C	-2.248550	-2.810656	0.405888
H	-2.830582	-2.774737	-0.513183
C	-3.103933	2.945294	0.411003
H	-3.568354	3.796919	-0.092452
H	-2.572877	3.319287	1.288923
H	-2.371993	2.504909	-0.266084
C	-5.229206	2.552749	1.720775
H	-5.728098	3.373737	1.200461
H	-5.989233	1.832705	2.025570
H	-4.779785	2.966470	2.626145
C	-0.764461	-2.788270	0.002178
H	-0.513165	-1.876003	-0.536444
H	-0.114193	-2.845507	0.876705
H	-0.536504	-3.638810	-0.644720
C	-2.580487	-4.117695	1.139895
H	-3.623038	-4.154289	1.456905
H	-2.391768	-4.974435	0.488570
H	-1.958681	-4.239994	2.029350
B	1.576208	0.746617	-1.693907
B	-2.125009	0.052822	-1.846582
C	2.985989	0.979533	-1.465395
C	3.976683	1.547348	-2.472069
C	5.328569	1.395585	-1.728471
C	5.013225	1.267771	-0.223383
H	6.004991	2.226529	-1.931288
H	5.823005	0.480954	-2.062751
N	3.631413	0.720718	-0.275209
C	2.999062	-0.021743	0.771691
C	2.205865	0.629557	1.735340
C	3.131669	-1.427164	0.778421
C	1.643031	-0.134479	2.758784
C	2.552932	-2.144169	1.823486
C	1.830259	-1.504380	2.818799
H	1.027039	0.351070	3.504517
H	2.650060	-3.222273	1.847542
H	1.387088	-2.078864	3.623305
C	3.966476	0.734202	-3.776813
H	4.162300	-0.321012	-3.579058
H	2.995023	0.808112	-4.270206
H	4.728476	1.105691	-4.468187
C	3.656355	3.017199	-2.802915
H	2.673523	3.093823	-3.270846
H	3.645124	3.639460	-1.908962
H	4.400580	3.423644	-3.494109
C	5.985246	0.328191	0.491684
H	6.977496	0.782324	0.511412
H	5.674774	0.151724	1.522991
H	6.064086	-0.631944	-0.012713
C	5.055297	2.632013	0.487840
H	4.811279	2.521520	1.543671
H	6.058589	3.058149	0.415960
H	4.354266	3.338835	0.047715
C	3.801502	-2.184431	-0.357222
H	4.311353	-1.456699	-0.986617
C	1.867990	2.108532	1.655333
H	2.405686	2.526618	0.807262
C	0.368043	2.303665	1.379681
H	0.142964	3.364467	1.247099
H	0.059585	1.776294	0.477937
H	-0.241092	1.930475	2.204874
C	2.285410	2.876590	2.917498
H	3.340305	2.728666	3.153790
H	2.113529	3.947292	2.784059
H	1.705389	2.555422	3.785498
C	2.741322	-2.870098	-1.235715
H	2.190139	-3.621560	-0.665922
H	2.025359	-2.143162	-1.619408
H	3.215912	-3.368580	-2.084757
C	4.842655	-3.201565	0.130301
H	5.371081	-3.636651	-0.721030
H	5.578731	-2.741268	0.791696

H	4.375916	-4.023634	0.677141
N	-0.884502	0.342084	-1.921905
N	0.331164	0.539045	-1.879684

Implicit toluene:

TS_{1a}

108

C	-4.045964	-2.413245	-2.157178
C	-4.731188	-1.149690	-1.596726
C	-2.380467	-0.929901	-1.248712
C	-2.517620	-2.143241	-2.179237
H	-4.427168	-2.676033	-3.144934
H	-4.257651	-3.252989	-1.492152
N	-3.640988	-0.585028	-0.761731
C	-5.124137	-0.179863	-2.727133
H	-5.812098	-0.668627	-3.421311
H	-5.621389	0.701988	-2.324075
H	-4.247607	0.145804	-3.286914
C	-2.011418	-1.831922	-3.599694
H	-0.948165	-1.584288	-3.578579
H	-2.147627	-2.699261	-4.252714
H	-2.541328	-0.986310	-4.039020
C	-1.743624	-3.359003	-1.638904
H	-1.921834	-4.235430	-2.270530
H	-0.673863	-3.152856	-1.612040
H	-2.058254	-3.595939	-0.621572
C	-5.981921	-1.483263	-0.784625
H	-6.412273	-0.586983	-0.334616
H	-6.731340	-1.923538	-1.445019
H	-5.769409	-2.196261	0.009454
C	-3.857843	0.127217	0.452653
C	-3.999782	1.526356	0.443813
C	-3.942109	-0.595145	1.665188
C	-4.339490	2.174180	1.633940
C	-4.286899	0.093866	2.826114
C	-4.508021	1.464476	2.810616
H	-4.461609	3.250570	1.638989
H	-4.371270	-0.443814	3.762029
H	-4.780348	1.980744	3.723560
C	-3.716427	2.351537	-0.799547
H	-3.532397	1.658935	-1.618021
C	-3.600485	-2.074659	1.737550
H	-3.643318	-2.470514	0.724781
C	-2.432879	3.178121	-0.611391
H	-2.196036	3.725194	-1.527421
H	-2.548903	3.906246	0.195704
H	-1.585424	2.535613	-0.369723
C	-4.891297	3.256770	-1.192611
H	-4.685818	3.753687	-2.143855
H	-5.817464	2.688931	-1.298579
H	-5.063534	4.033325	-0.443920
C	-2.156459	-2.263512	2.232718
H	-1.442739	-1.766673	1.575601
H	-2.034496	-1.860945	3.240737
H	-1.901342	-3.326022	2.261692
C	-4.578382	-2.887292	2.595857
H	-5.614205	-2.729573	2.288292
H	-4.354233	-3.952826	2.506479
H	-4.501890	-2.627398	3.653983
B	1.126658	0.018271	0.015242
B	-1.113919	-0.296453	-0.976323
C	2.238058	0.779116	0.699004
C	2.960788	1.943389	0.057691
C	4.218306	2.082730	0.949110
C	3.915267	1.380928	2.289587
H	4.497729	3.125303	1.101317
H	5.059440	1.585138	0.464631
N	2.730716	0.526185	1.910896
C	2.089753	-0.388511	2.823477
C	1.016702	0.081911	3.605995
C	2.510666	-1.728758	2.878387
C	0.446620	-0.796374	4.525729
C	1.905167	-2.563370	3.818761
C	0.898344	-2.100743	4.649306
H	-0.381271	-0.460720	5.136479
H	2.211925	-3.599374	3.882778
H	0.439459	-2.767243	5.369342
C	3.328872	1.656894	-1.404866
H	3.921494	0.744003	-1.487247
H	2.431869	1.536382	-2.013347
H	3.914164	2.486160	-1.810393
C	2.052663	3.191286	0.113716
H	1.127798	3.013018	-0.436285

H	1.791610	3.459797	1.137377
H	2.568678	4.041665	-0.338892
C	5.084237	0.515615	2.759496
H	5.935001	1.160402	2.986067
H	4.826060	-0.034237	3.665291
H	5.391687	-0.192940	1.993308
C	3.564857	2.368922	3.408613
H	3.213944	1.841360	4.295574
H	4.463200	2.925197	3.679792
H	2.805901	3.086329	3.106014
C	3.508143	-2.330249	1.900981
H	3.889278	-1.530210	1.267733
C	0.391611	1.454687	3.413097
H	1.041595	2.038214	2.768990
C	-0.953091	1.323792	2.686008
H	-1.375392	2.309362	2.484699
H	-0.849369	0.803785	1.734776
H	-1.672402	0.770360	3.287501
C	0.222337	2.232005	4.725040
H	1.164101	2.322041	5.268141
H	-0.148820	3.238362	4.517597
H	-0.500628	1.749143	5.385380
C	2.805056	-3.337311	0.974320
H	2.425552	-4.190585	1.541555
H	1.965997	-2.876991	0.451584
H	3.509354	-3.715381	0.229075
C	4.700892	-2.994763	2.604124
H	5.426411	-3.341158	1.864742
H	5.208502	-2.310330	3.284267
H	4.381572	-3.863533	3.183653
N	0.026053	0.426553	-0.902164
N	0.660426	-1.188539	-0.407301

Int$_{1a}$

108

C	-4.911570	-0.595755	-2.695908
C	-5.242008	-0.618221	-1.187066
C	-2.950102	0.010301	-1.492667
C	-3.569685	0.157986	-2.867178
H	-5.715097	-0.140314	-3.274992
H	-4.791604	-1.620984	-3.049841
N	-3.886215	-0.371760	-0.594785
C	-6.251187	0.471637	-0.798462
H	-7.209301	0.254012	-1.273813
H	-6.408283	0.489786	0.279468
H	-5.934814	1.460981	-1.121886
C	-3.776777	1.650905	-3.195155
H	-2.814530	2.161262	-3.256224
H	-4.287436	1.756293	-4.156275
H	-4.373327	2.153688	-2.433936
C	-2.692208	-0.480531	-3.951491
H	-3.185127	-0.414523	-4.924853
H	-1.728169	0.027231	-4.020540
H	-2.500595	-1.531916	-3.730584
C	-5.807986	-1.968620	-0.743236
H	-5.916996	-2.010886	0.341588
H	-6.794843	-2.107000	-1.188363
H	-5.176028	-2.793802	-1.061906
C	-3.524130	-0.567466	0.783173
C	-3.626476	0.510468	1.684285
C	-2.967749	-1.801257	1.175482
C	-3.238432	0.300644	3.007413
C	-2.593187	-1.955511	2.510470
C	-2.737838	-0.923120	3.423298
H	-3.309009	1.115202	3.717238
H	-2.161840	-2.893908	2.834081
H	-2.438587	-1.065041	4.454864
C	-4.059711	1.904973	1.258568
H	-4.388419	1.853350	0.225528
C	-2.697796	-2.939625	0.203446
H	-3.096102	-2.655446	-0.768700
C	-2.874337	2.881676	1.288356
H	-3.194020	3.872727	0.956380
H	-2.464146	2.977677	2.295829
H	-2.078660	2.539878	0.627953
C	-5.226455	2.440889	2.100035
H	-5.576222	3.393727	1.696333
H	-6.068633	1.746969	2.112621
H	-4.923670	2.612575	3.135186
C	-1.188868	-3.162582	0.019157
H	-0.672264	-2.272628	-0.344082
H	-0.720962	-3.459342	0.960390
H	-1.017852	-3.964439	-0.703578

C	-3.389709	-4.241680	0.635907
H	-4.458938	-4.100106	0.802176
H	-3.260515	-5.007147	-0.132781
H	-2.957125	-4.629379	1.560499
B	1.121884	0.129547	-0.687947
B	-1.533882	0.251378	-1.160865
C	2.048094	1.189723	0.043726
C	1.616567	2.550069	0.545335
C	2.945107	3.236257	0.928541
C	4.001018	2.124424	1.054949
H	2.858827	3.810551	1.850923
H	3.239503	3.928632	0.138710
N	3.301006	0.971568	0.349117
C	3.998943	-0.279228	0.114239
C	3.956976	-1.280870	1.102246
C	4.742008	-0.433644	-1.069134
C	4.765270	-2.401532	0.920827
C	5.528151	-1.577502	-1.196966
C	5.561808	-2.543690	-0.204428
H	4.749467	-3.190176	1.662158
H	6.105355	-1.724647	-2.100608
H	6.183288	-3.423091	-0.322608
C	0.859097	3.356993	-0.518061
H	1.459829	3.465777	-1.423539
H	-0.076761	2.873124	-0.787517
H	0.645848	4.355678	-0.128760
C	0.708846	2.307260	1.770845
H	-0.140611	1.681876	1.502322
H	1.250454	1.815295	2.579526
H	0.336783	3.264142	2.143273
C	5.305562	2.495346	0.353997
H	5.754418	3.338316	0.881661
H	6.015184	1.668007	0.367923
H	5.134879	2.795653	-0.677463
C	4.310711	1.762229	2.508329
H	4.992638	0.914769	2.558954
H	4.794767	2.615553	2.985060
H	3.413390	1.522914	3.074634
C	4.630181	0.512629	-2.255029
H	4.050832	1.383365	-1.947747
C	2.983858	-1.266564	2.272717
H	2.493987	-0.295358	2.301628
C	1.876777	-2.310628	2.042926
H	1.105320	-2.215286	2.812179
H	1.421572	-2.167072	1.064520
H	2.284682	-3.323506	2.094413
C	3.658728	-1.496947	3.632371
H	4.459919	-0.783308	3.824482
H	2.921614	-1.403368	4.433665
H	4.083976	-2.500447	3.696586
C	3.843760	-0.165371	-3.392541
H	4.402269	-1.015973	-3.791965
H	2.881350	-0.523618	-3.025935
H	3.680649	0.543884	-4.208546
C	5.992420	1.003977	-2.767016
H	5.848646	1.742461	-3.559077
H	6.589600	1.461924	-1.977822
H	6.574731	0.182520	-3.189179
N	-0.307980	0.446835	-0.913640
N	1.492103	-1.086624	-1.112497

Pdt_a

108

C	-1.936456	-0.037237	-6.198737
N	-1.384064	-0.682769	-5.241298
B	-1.188709	-0.966337	-3.963180
N	-0.878098	-1.311165	-2.755422
B	-0.576891	-1.622813	-1.500266
C	1.420036	1.634510	-0.508923
C	-0.190667	-1.977021	-0.182205
H	2.512021	1.602694	-0.551553
C	2.220346	-2.683768	0.115048
H	2.397306	-1.764851	0.671446
H	2.879368	-3.459701	0.519115
H	2.502719	-2.497732	-0.922881
C	-1.544628	-0.219706	-7.656915
C	-0.142298	0.374330	-7.879305
H	-0.781696	-2.241537	-7.445546
H	-0.102564	1.434340	-7.626418
H	0.590592	-0.147366	-7.262487
H	0.143354	0.264704	-8.927662
C	-1.533630	-1.707027	-8.026482
H	-1.303501	-1.825419	-9.087747

H	-2.504653	-2.168002	-7.833113
C	-2.650037	0.554931	-8.400772
H	-2.269380	1.083665	-9.274555
H	-3.410935	-0.145693	-8.748493
H	-2.962530	3.357968	-8.481757
H	-1.628783	2.955566	-7.399024
C	-2.712645	2.944975	-7.502809
C	-3.290555	1.529556	-7.386861
H	-3.138978	3.598638	-6.743333
H	-4.598220	-0.837852	-6.136088
C	-4.806321	1.611268	-7.564879
H	-5.265626	0.625863	-7.562106
H	-5.024775	2.080914	-8.525441
H	-5.266291	2.213987	-6.780569
H	-6.903887	-1.613121	-6.140454
H	-6.926489	0.152722	-6.028200
C	-6.638842	-0.762343	-5.508728
H	-7.238183	-0.830867	-4.599023
C	-5.136938	-0.788104	-5.191299
C	-4.776734	-2.057592	-4.400800
C	-3.543354	1.202723	-4.828569
H	-5.062064	-2.948625	-4.965413
N	-2.911030	0.894094	-6.082252
H	-3.706916	-2.108213	-4.197638
H	-1.333909	2.585552	-5.243332
C	-1.704988	2.920394	-4.276827
H	-0.906729	4.907559	-4.624859
C	-1.851474	4.448497	-4.325079
H	-5.298673	-2.079919	-3.442621
C	-4.669606	0.449259	-4.439663
H	-2.115452	4.848313	-3.343702
C	-3.023154	2.215555	-4.002441
C	-3.713762	2.526435	-2.830485
H	-3.325198	3.290831	-2.170612
C	-4.867108	1.850160	-2.475579
H	-6.194114	0.245849	-2.947425
H	-5.384164	2.105716	-1.559493
C	-5.327021	0.809485	-3.265742
C	-0.659021	2.524242	-3.222903
H	0.298991	3.002824	-3.440010
H	-0.503111	1.446894	-3.196068

H	-4.874650	-0.689441	-0.884443
C	-4.120951	-1.466316	-0.746094
H	-4.605470	-2.435804	-0.889590
H	-0.969921	2.830621	-2.222727
C	-3.495429	-1.390542	0.655200
C	-2.700562	-0.104631	0.823062
H	-2.794053	-2.218534	0.734571
H	-4.457065	1.118711	0.687839
C	-3.377690	1.114637	0.774827
C	-2.694324	2.319362	0.808222
H	-3.238360	3.256006	0.767173
H	-5.354671	-0.792942	1.628641
C	-4.582388	-1.560136	1.726084
H	-4.173238	-1.494567	2.734209
H	-5.070580	-2.532358	1.619300
H	-0.775689	3.263174	0.842536
C	-1.308420	2.319702	0.859749
C	-0.588510	1.126244	0.918152
H	1.103272	2.658014	-0.722975
C	-1.293034	-0.098975	0.950826
H	1.027775	0.983827	-1.290635
C	0.742492	-3.120338	0.191302
C	0.552679	-4.358889	-0.690932
H	1.201684	-5.175491	-0.360920
H	0.803720	-4.136146	-1.731679
H	-0.483196	-4.700557	-0.661197
C	0.928780	1.174819	0.872289
H	1.283329	0.157907	1.008067
C	1.532119	2.046388	1.981431
H	2.622617	1.975684	1.966786
H	1.185844	1.736312	2.968430
H	1.269280	3.099088	1.851452
H	-2.213974	-2.923364	2.621873
H	0.431943	-0.375034	3.417833
C	-1.456787	-2.336101	3.137631
H	-1.906638	-1.394866	3.459902
H	-1.153418	-2.886541	4.031080
C	0.810674	-1.349530	3.106891
C	-0.236330	-2.077081	2.242480
H	1.748587	-1.198307	2.577104
H	1.022923	-1.930932	4.007188

C	0.307451	-3.398315	1.651951
H	1.120412	-3.806395	2.255656
N	-0.572413	-1.323050	1.013215
H	-2.621980	4.764283	-5.029159
H	-3.370240	-1.341690	-1.524976
H	-0.496557	-4.138140	1.641460

TS_{2b}

108

C	-5.040655	-0.932224	-2.711813
C	-5.157501	-0.113231	-1.407064
C	-2.846217	-0.344047	-2.029840
C	-3.625901	-0.695298	-3.290915
H	-5.824045	-0.666067	-3.422125
H	-5.154327	-1.992982	-2.480405
N	-3.718837	0.147525	-1.087993
C	-5.965643	1.178488	-1.594396
H	-7.014157	0.925872	-1.761890
H	-5.909076	1.800543	-0.700734
H	-5.624557	1.762423	-2.446466
C	-3.605171	0.479812	-4.289536
H	-2.587175	0.665536	-4.635534
H	-4.228324	0.247247	-5.157932
H	-3.979562	1.400163	-3.844318
C	-3.087173	-1.947870	-3.989981
H	-3.715929	-2.206676	-4.846120
H	-2.071147	-1.784551	-4.357206
H	-3.064685	-2.797580	-3.305848
C	-5.828605	-0.931774	-0.298959
H	-5.856911	-0.372216	0.637152
H	-6.856558	-1.162356	-0.586468
H	-5.305180	-1.871139	-0.129030
C	-3.285905	0.852189	0.081890
C	-3.150656	2.257533	0.024568
C	-2.977752	0.151277	1.265254
C	-2.828623	2.943686	1.193980
C	-2.661925	0.886320	2.409596
C	-2.611137	2.269462	2.385686
H	-2.726287	4.020941	1.167817
H	-2.432775	0.362932	3.329310
H	-2.365751	2.820907	3.285261
C	-3.249213	3.031078	-1.280497
H	-3.674130	2.366954	-2.025250
C	-2.881582	-1.364605	1.323295
H	-3.185422	-1.756978	0.354078
C	-1.846701	3.407731	-1.783290
H	-1.915102	3.943988	-2.733103
H	-1.327045	4.048029	-1.066666
H	-1.243069	2.514333	-1.940719
C	-4.148391	4.269918	-1.190287
H	-4.268836	4.715771	-2.180314
H	-5.139089	4.020539	-0.807493
H	-3.719500	5.034015	-0.538574
C	-1.422442	-1.798543	1.548788
H	-0.757662	-1.379700	0.794341
H	-1.065057	-1.478769	2.530629
H	-1.344464	-2.886216	1.500443
C	-3.784833	-1.980410	2.401833
H	-4.825039	-1.677552	2.283496
H	-3.739347	-3.070819	2.352082
H	-3.462629	-1.683392	3.402322
B	1.377189	-0.676712	-1.113301
B	-1.425805	-0.590044	-1.883299
C	2.147500	0.209096	-0.254461
C	1.831941	1.662181	0.068365
C	2.992353	2.046719	1.023933
C	3.566229	0.729834	1.589571
H	2.663548	2.719715	1.816284
H	3.775190	2.560130	0.461748
N	3.231948	-0.211083	0.485648
C	4.004454	-1.349999	0.098191
C	3.745553	-2.616483	0.653186
C	4.999001	-1.184473	-0.891973
C	4.552647	-3.689465	0.266820
C	5.768579	-2.288331	-1.251963
C	5.562164	-3.529716	-0.667057
H	4.373302	-4.669239	0.691755
H	6.536460	-2.179359	-2.007414
H	6.175341	-4.374910	-0.955787
C	1.859479	2.514678	-1.212447
H	2.822936	2.430880	-1.717510
H	1.084559	2.190468	-1.909008
H	1.680988	3.568227	-0.977464

C	0.459218	1.813635	0.739735
H	-0.335735	1.445294	0.092805
H	0.400246	1.259182	1.673567
H	0.251995	2.864006	0.955536
C	5.069707	0.806472	1.845222
H	5.262114	1.514269	2.653221
H	5.466466	-0.165012	2.145198
H	5.612978	1.141811	0.963936
C	2.863919	0.327529	2.899348
H	3.296407	-0.584290	3.307489
H	2.982871	1.119193	3.642478
H	1.798968	0.160846	2.744799
C	5.214519	0.136092	-1.614198
H	4.661403	0.903589	-1.077034
C	2.589648	-2.874382	1.604457
H	2.059836	-1.934175	1.746620
C	1.588020	-3.870577	0.997095
H	0.743675	-4.011191	1.674899
H	1.206769	-3.506827	0.042729
H	2.049600	-4.847030	0.833231
C	3.073285	-3.370525	2.975136
H	3.813637	-2.697636	3.411332
H	2.233341	-3.451513	3.669239
H	3.533860	-4.357834	2.893570
C	4.631677	0.071833	-3.035487
H	5.156958	-0.674896	-3.636033
H	3.574249	-0.192335	-3.009321
H	4.734080	1.039630	-3.533519
C	6.687302	0.567228	-1.650367
H	6.772426	1.570579	-2.074097
H	7.128748	0.579432	-0.652038
H	7.287989	-0.101417	-2.270382
N	-0.179105	-0.914742	-1.773636
N	0.949004	-1.675845	-1.931324

Pdt_b

108

C	-5.616087	-1.338908	-2.193170
C	-5.476874	-1.247170	-0.657293
C	-3.514258	-0.335424	-1.713363
C	-4.555126	-0.399706	-2.816276
H	-6.626981	-1.091640	-2.519661
H	-5.410773	-2.363802	-2.507870
N	-4.096033	-0.702976	-0.520357
C	-6.527804	-0.323293	-0.023580
H	-7.518621	-0.768274	-0.133329
H	-6.331834	-0.197979	1.041960
H	-6.547169	0.659697	-0.488397
C	-5.130318	1.004690	-3.094619
H	-4.351920	1.661478	-3.486598
H	-5.938024	0.945222	-3.830494
H	-5.529489	1.465497	-2.191951
C	-3.989695	-0.970172	-4.121205
H	-4.775574	-1.051232	-4.877173
H	-3.205026	-0.321857	-4.518739
H	-3.558996	-1.959651	-3.959565
C	-5.608364	-2.626861	-0.004287
H	-5.421080	-2.570219	1.069123
H	-6.622089	-3.005738	-0.151512
H	-4.913520	-3.341393	-0.441683
C	-3.412226	-0.539021	0.726563
C	-3.515593	0.687101	1.418383
C	-2.590637	-1.573136	1.220530
C	-2.902763	0.800606	2.665710
C	-1.992126	-1.407042	2.469826
C	-2.166607	-0.244619	3.201729
H	-2.980100	1.731260	3.213252
H	-1.356428	-2.190105	2.861988
H	-1.696941	-0.137324	4.172166
C	-4.173850	1.914237	0.808220
H	-4.666090	1.604788	-0.109029
C	-2.249351	-2.810614	0.405215
H	-2.831751	-2.775353	-0.513599
C	-3.104213	2.945933	0.410959
H	-3.568968	3.797045	-0.093381
H	-2.574146	3.319440	1.289682
H	-2.371365	2.504543	-0.264805
C	-5.228841	2.553060	1.720366
H	-5.727752	3.373708	1.199451
H	-5.988364	1.831895	2.024002
H	-4.778325	2.965819	2.625758
C	-0.765207	-2.788708	0.002091
H	-0.513036	-1.875811	-0.535343

H	-0.115653	-2.845625	0.877131
H	-0.537396	-3.639918	-0.644224
C	-2.580227	-4.117425	1.139673
H	-3.622979	-4.153408	1.456264
H	-2.391564	-4.973979	0.488011
H	-1.957467	-4.237750	2.028826
B	1.576762	0.745012	-1.691368
B	-2.125896	0.052588	-1.844414
C	2.987053	0.979000	-1.463557
C	3.976380	1.547383	-2.471332
C	5.328833	1.395654	-1.729040
C	5.014774	1.267966	-0.223878
H	6.004286	2.226931	-1.932326
H	5.823144	0.481234	-2.063666
N	3.631549	0.720631	-0.275184
C	2.999089	-0.021861	0.771851
C	2.205994	0.629875	1.735169
C	3.132467	-1.427121	0.778451
C	1.643157	-0.134088	2.759119
C	2.553982	-2.144195	1.824101
C	1.830987	-1.504136	2.819530
H	1.026989	0.350508	3.505430
H	2.650398	-3.222328	1.848548
H	1.386714	-2.078791	3.623369
C	3.965358	0.734282	-3.775982
H	4.162777	-0.320621	-3.578147
H	2.993838	0.809047	-4.269476
H	4.727457	1.106806	-4.466565
C	3.655718	3.017126	-2.802077
H	2.673463	3.093774	-3.271655
H	3.645160	3.639006	-1.907880
H	4.400786	3.422596	-3.492764
C	5.986348	0.328212	0.491233
H	6.978464	0.782636	0.510197
H	5.675952	0.153481	1.522940
H	6.065077	-0.632030	-0.012943
C	5.055936	2.631842	0.487665
H	4.813248	2.520328	1.543783
H	6.059193	3.057709	0.414643
H	4.354338	3.338580	0.048182
C	3.802509	-2.184314	-0.357270
H	4.312834	-1.457846	-0.987384
C	1.868452	2.108821	1.654814
H	2.406152	2.527549	0.807190
C	0.368405	2.303672	1.380227
H	0.143362	3.364710	1.248499
H	0.059274	1.775004	0.479338
H	-0.239859	1.929827	2.205748
C	2.285078	2.876729	2.917224
H	3.340179	2.728666	3.152551
H	2.112935	3.947344	2.783024
H	1.703967	2.554651	3.784280
C	2.742679	-2.870930	-1.235604
H	2.192282	-3.622576	-0.665272
H	2.026165	-2.143832	-1.618193
H	3.218030	-3.368425	-2.084906
C	4.842747	-3.202022	0.130651
H	5.370999	-3.636302	-0.721157
H	5.578831	-2.740945	0.791553
H	4.374605	-4.023252	0.677672
N	-0.885696	0.344061	-1.927427
N	0.332448	0.538431	-1.885180

XYZ coordinates for Figure S2

Ts_1''

108

C	-1.779219	-1.957287	-4.433961
C	-2.765382	-2.298001	-3.312400
C	-0.970503	-0.961272	-2.424264
C	-0.940352	-0.748431	-3.954024
H	-2.286450	-1.754087	-5.377203
H	-1.119266	-2.813426	-4.590320
N	-1.982989	-1.821645	-2.122351
C	-4.112519	-1.568129	-3.421532
H	-4.627734	-1.883926	-4.330468
H	-4.744040	-1.824983	-2.571534
H	-4.004264	-0.488761	-3.451508
C	-1.584238	0.562625	-4.451780
H	-0.957672	1.422482	-4.238513
H	-1.724479	0.495901	-5.533419
H	-2.554264	0.748639	-3.995012
C	0.494129	-0.833348	-4.491811
H	0.484550	-0.810866	-5.584855

H	1.097689	0.003911	-4.141776
H	0.980307	-1.755589	-4.169229
C	-3.068440	-3.797125	-3.274674
H	-3.655149	-4.060408	-2.394101
H	-3.656104	-4.056423	-4.156995
H	-2.164233	-4.399908	-3.288455
C	-2.415568	-2.086284	-0.777819
C	-3.220394	-1.145155	-0.097723
C	-2.046564	-3.301337	-0.170465
C	-3.711895	-1.497106	1.158586
C	-2.568153	-3.600891	1.087453
C	-3.411778	-2.718280	1.739804
H	-4.332411	-0.792969	1.697899
H	-2.294094	-4.530632	1.568994
H	-3.811437	-2.967971	2.715106
C	-3.530571	0.246635	-0.631821
H	-3.107972	0.331555	-1.629983
C	-1.028559	-4.243021	-0.791335
H	-0.841335	-3.911352	-1.810166
C	-2.847986	1.325892	0.225107
H	-3.039286	2.313731	-0.199584
H	-3.231704	1.316308	1.248042
H	-1.770181	1.174751	0.252978
C	-5.039306	0.522038	-0.721666
H	-5.213587	1.489527	-1.198166
H	-5.562936	-0.239456	-1.299217
H	-5.496270	0.558745	0.269377
C	0.298157	-4.136325	-0.036022
H	0.675898	-3.114410	-0.025524
H	0.182615	-4.452249	0.999654
H	1.055515	-4.771497	-0.498252
C	-1.496600	-5.704074	-0.836581
H	-2.467741	-5.810213	-1.319705
H	-0.773755	-6.311364	-1.386324
H	-1.575735	-6.126307	0.167313
B	1.305184	0.270839	-0.847364
B	-0.101409	-0.335172	-1.394077
C	2.599407	0.436603	-0.167551
C	3.652587	1.486969	-0.601918
C	4.810981	1.256029	0.398500
C	4.211778	0.536162	1.609571
H	5.305110	2.188093	0.675945
H	5.566663	0.611124	-0.055574
N	3.083681	-0.197490	0.955945
C	2.676810	-1.473935	1.451610
C	1.729943	-1.577432	2.484265
C	3.341938	-2.621528	0.961904
C	1.544872	-2.820658	3.094217
C	3.128310	-3.836263	1.606774
C	2.259931	-3.932179	2.685425
H	0.827237	-2.915657	3.899352
H	3.644071	-4.723459	1.264327
H	2.116624	-4.884306	3.182246
C	4.154951	1.222844	-2.033945
H	4.457130	0.180725	-2.154763
H	3.378247	1.434699	-2.766664
H	5.020855	1.857320	-2.250325
C	3.147026	2.939487	-0.514115
H	2.347386	3.125166	-1.227547
H	2.772946	3.179608	0.480062
H	3.965928	3.627389	-0.742899
C	5.222681	-0.390740	2.284440
H	6.028128	0.214730	2.704211
H	4.766627	-0.956738	3.097464
H	5.664401	-1.095311	1.582512
C	3.690547	1.527013	2.665675
H	3.306808	0.998453	3.536342
H	4.502322	2.177272	2.998522
H	2.892411	2.151916	2.269332
C	4.244300	-2.553158	-0.261487
H	4.577481	-1.522222	-0.364547
C	0.866411	-0.404247	2.912435
H	1.227295	0.480798	2.391414
C	-0.584686	-0.633647	2.467294
H	-1.205765	0.226033	2.727009
H	-0.645080	-0.775449	1.389435
H	-1.011727	-1.515650	2.947715
C	0.923081	-0.138001	4.423352
H	1.947899	-0.041384	4.784738
H	0.388884	0.784184	4.663437
H	0.451274	-0.945812	4.987167
C	3.451063	-2.890784	-1.535776

H	3.094649	-3.922895	-1.506401
H	2.592112	-2.229326	-1.646709
H	4.086623	-2.776705	-2.417852
C	5.495182	-3.434294	-0.157573
H	6.163895	-3.229477	-0.996182
H	6.043567	-3.252486	0.768681
H	5.248053	-4.497521	-0.194465
N	-0.170286	1.624533	-2.146842
N	0.948605	1.394681	-1.861667

Int_1''

108

C	-4.391448	-1.172402	-2.441173
C	-4.547961	-0.664911	-1.001398
C	-2.248694	-0.349791	-1.701002
C	-3.045580	-0.644150	-2.979837
H	-5.232110	-0.872809	-3.068779
H	-4.366178	-2.263682	-2.434561
N	-3.132436	-0.313749	-0.654343
C	-5.489506	0.545628	-0.911927
H	-6.502121	0.233616	-1.175020
H	-5.515262	0.942168	0.102959
H	-5.198553	1.343470	-1.591740
C	-3.218717	0.669262	-3.770991
H	-2.246992	1.021948	-4.116427
H	-3.863090	0.507428	-4.640667
H	-3.665131	1.456077	-3.161289
C	-2.423380	-1.698036	-3.905928
H	-3.136415	-1.954225	-4.695721
H	-1.508185	-1.333474	-4.366655
H	-2.181969	-2.606942	-3.351718
C	-5.103784	-1.761722	-0.088219
H	-5.149479	-1.432993	0.950780
H	-6.116422	-2.016980	-0.406784
H	-4.496547	-2.662629	-0.143688
C	-2.806107	0.038156	0.694907
C	-2.951713	1.377331	1.112538
C	-2.428807	-0.962571	1.613388
C	-2.872651	1.663641	2.474463
C	-2.357684	-0.623796	2.965281
C	-2.615241	0.665542	3.400599
H	-3.001025	2.683252	2.813869
H	-2.087389	-1.383671	3.687412
H	-2.571760	0.902271	4.456919
C	-3.075374	2.516197	0.112642
H	-3.346961	2.088477	-0.848271
C	-2.029042	-2.366925	1.185548
H	-2.257034	-2.471037	0.124885
C	-1.697800	3.167712	-0.076529
H	-1.740017	3.936148	-0.853195
H	-1.351291	3.635061	0.848556
H	-0.968812	2.415553	-0.373060
C	-4.129204	3.566411	0.479747
H	-4.226202	4.295810	-0.327708
H	-5.108144	3.116373	0.647857
H	-3.855097	4.117839	1.381668
C	-0.509339	-2.549009	1.340612
H	0.030452	-1.809603	0.751654
H	-0.206008	-2.450584	2.385518
H	-0.209468	-3.541001	0.994937
C	-2.769446	-3.469288	1.957865
H	-3.851053	-3.338191	1.929358
H	-2.532139	-4.446943	1.532031
H	-2.465819	-3.490843	3.006663
B	0.835310	-0.215311	-1.322121
B	-0.772087	-0.266391	-1.682088
C	2.065761	0.765066	-1.161206
C	2.427394	1.995360	-1.982505
C	3.969900	2.002291	-1.892707
C	4.355190	1.214885	-0.629178
H	4.375768	3.013957	-1.874015
H	4.375672	1.499509	-2.771712
N	3.068823	0.452147	-0.364981
C	2.976109	-0.520783	0.710951
C	2.581944	-0.054376	1.981358
C	3.284134	-1.876815	0.489082
C	2.585047	-0.954664	3.044282
C	3.277249	-2.729304	1.595242
C	2.950948	-2.277416	2.861303
H	2.276248	-0.617568	4.024827
H	3.511364	-3.775673	1.451275
H	2.950984	-2.962941	3.699758
C	1.956486	1.964651	-3.438094

H	2.317078	1.071824	-3.946080
H	0.869574	1.959279	-3.504508
H	2.334825	2.852673	-3.951197
C	1.811519	3.234027	-1.293533
H	0.726884	3.208482	-1.360810
H	2.081302	3.311135	-0.240844
H	2.165378	4.135370	-1.798999
C	5.501304	0.242389	-0.897741
H	6.394120	0.813244	-1.158839
H	5.727498	-0.355452	-0.014720
H	5.269957	-0.423162	-1.726074
C	4.748435	2.115997	0.544053
H	4.874763	1.534172	1.456413
H	5.703863	2.589611	0.313497
H	4.022765	2.903529	0.729156
C	3.529068	-2.490570	-0.881786
H	3.474645	-1.704059	-1.632310
C	2.048080	1.349015	2.217012
H	2.273340	1.950971	1.343458
C	0.522028	1.305741	2.344648
H	0.118095	2.312645	2.460293
H	0.057794	0.852178	1.469589
H	0.221592	0.722606	3.214875
C	2.673087	2.048017	3.430934
H	3.762565	2.056357	3.382744
H	2.327193	3.082889	3.481966
H	2.383912	1.564695	4.365857
C	2.413969	-3.496718	-1.220465
H	2.454986	-4.364572	-0.557436
H	1.432374	-3.036121	-1.137427
H	2.532849	-3.845667	-2.247613
C	4.897055	-3.184481	-0.984615
H	5.055958	-3.539398	-2.005208
H	5.722508	-2.523666	-0.723187
H	4.948682	-4.053257	-0.324927
N	0.057424	-0.411495	-2.886775
N	1.239040	-0.936368	-2.537468

RC$_{KCl}$

110

C	3.927537	-4.919878	1.946843
C	4.350373	-4.286239	0.609824
C	2.009327	-3.928943	0.869124
C	2.376774	-4.947972	1.959539
H	4.290205	-4.297655	2.767250
H	4.355357	-5.914451	2.076826
N	3.150352	-3.438655	0.322108
C	5.642215	-3.478392	0.705052
H	5.624561	-2.761869	1.522302
H	6.475901	-4.161203	0.875083
H	5.834492	-2.936709	-0.222479
C	1.831068	-4.474650	3.318027
H	2.177081	-5.139977	4.113951
H	2.167188	-3.462102	3.546107
H	0.740447	-4.474582	3.316342
C	1.809453	-6.343091	1.656545
H	2.149976	-6.720360	0.692906
H	2.119611	-7.050274	2.430298
H	0.720340	-6.307536	1.634922
C	4.527571	-5.354793	-0.483071
H	4.820933	-4.900843	-1.428134
H	5.311455	-6.055346	-0.189298
H	3.609697	-5.918239	-0.644668
C	3.167575	-2.205867	-0.415752
C	3.410997	-1.013501	0.307214
C	2.889708	-2.179160	-1.792828
C	3.393861	0.196404	-0.385484
C	2.885558	-0.939050	-2.439522
C	3.134669	0.235259	-1.749284
H	3.508865	1.129424	0.147147
H	2.671347	-0.896348	-3.500248
H	3.095547	1.191151	-2.255395
C	3.606133	-0.993489	1.819279
H	3.799803	-2.012020	2.148360
C	2.580409	-3.429317	-2.599941
H	2.571283	-4.275363	-1.915250
C	2.328854	-0.529831	2.538733
H	1.504439	-1.218940	2.337927
H	2.484061	-0.517889	3.620318
H	2.050441	0.480828	2.227866
C	4.799805	-0.127555	2.245237
H	4.989192	-0.251544	3.313921
H	5.707472	-0.398310	1.702586

H	4.606710	0.931422	2.068251
C	1.187492	-3.364108	-3.242411
H	0.981992	-4.286224	-3.791356
H	0.420401	-3.247760	-2.477755
H	1.107493	-2.533794	-3.947560
C	3.654285	-3.681626	-3.669660
H	3.637042	-2.900010	-4.432875
H	4.657645	-3.695322	-3.241158
H	3.480840	-4.637942	-4.168749
B	-0.787721	-3.663181	0.086861
B	0.633516	-3.606011	0.518013
C	-2.153407	-4.036822	-0.234566
C	-2.495199	-5.371004	-0.918034
C	-4.024452	-5.263219	-1.136197
C	-4.543179	-4.186685	-0.168474
H	-4.528599	-6.218803	-0.988197
H	-4.224161	-4.943824	-2.161027
N	-3.309910	-3.354113	-0.003307
C	-3.326086	-1.970197	0.367798
C	-3.223721	-1.580326	1.717088
C	-3.419769	-1.005211	-0.662667
C	-3.296287	-0.217353	2.019887
C	-3.487732	0.342469	-0.305065
C	-3.440920	0.737179	1.026179
H	-3.219512	0.100626	3.051161
H	-3.559651	1.098221	-1.076687
H	-3.482978	1.788188	1.281703
C	-1.757387	-5.495991	-2.259972
H	-0.680287	-5.551988	-2.102661
H	-2.080874	-6.399312	-2.785252
H	-1.957707	-4.635375	-2.900467
C	-2.112391	-6.568664	-0.032349
H	-2.413306	-7.502594	-0.514440
H	-1.034030	-6.587759	0.121828
H	-2.587810	-6.521206	0.946363
C	-5.717844	-3.397518	-0.744402
H	-5.506105	-3.016548	-1.740268
H	-6.586259	-4.054078	-0.815612
H	-5.984796	-2.557765	-0.100901
C	-4.983152	-4.789988	1.175515
H	-5.806424	-5.488293	1.012681
H	-4.171677	-5.328247	1.661576
H	-5.331918	-4.012615	1.853380
C	-3.363768	-1.377730	-2.138282
H	-3.498371	-2.454969	-2.216293
C	-2.979199	-2.567970	2.846833
H	-2.967002	-3.568070	2.419864
C	-1.599765	-2.339055	3.486767
H	-1.424232	-3.076152	4.273556
H	-0.807096	-2.447484	2.745894
H	-1.526641	-1.346620	3.935953
C	-4.081610	-2.508695	3.914450
H	-3.938732	-3.304610	4.648383
H	-4.062368	-1.558175	4.451495
H	-5.076337	-2.620101	3.480414
C	-1.976399	-1.065626	-2.723293
H	-1.925345	-1.367805	-3.771726
H	-1.763486	0.006005	-2.679133
H	-1.199907	-1.611871	-2.186890
C	-4.462469	-0.701044	-2.969145
H	-4.460621	-1.097526	-3.986607
H	-5.451643	-0.864499	-2.539439
H	-4.306497	0.376915	-3.041165
N	-0.448820	2.021080	4.079453
N	-0.487079	1.249161	3.309825
Cl	1.021366	2.558841	0.531361
K	-0.033873	-0.043736	0.176416

Int$_{KCl}$

110

Cl	-1.378328	-4.671638	0.499078
C	-3.043261	-0.458325	3.083343
C	-3.946877	-0.817047	1.900236
C	-1.830953	-0.062877	1.036331
C	-1.606530	-0.322818	2.531537
H	-3.106954	-1.199994	3.879098
H	-3.359414	0.499745	3.501324
N	-3.119346	-0.273958	0.752454
C	-4.159813	-2.329368	1.768558
H	-4.825988	-2.555275	0.939018
H	-3.237082	-2.888591	1.621900
H	-4.636373	-2.692926	2.680981
C	-0.794421	-1.609506	2.755210

H	-1.242255	-2.484534	2.282844
H	0.215531	-1.472247	2.371437
H	-0.708615	-1.809261	3.825613
C	-0.894565	0.853962	3.216465
H	-0.881187	0.687715	4.297005
H	0.128636	0.959453	2.861855
H	-1.414548	1.794131	3.021788
C	-5.311932	-0.137372	1.990342
H	-5.895682	-0.293646	1.082951
H	-5.863019	-0.575364	2.824002
H	-5.226635	0.931088	2.172688
C	-3.712587	0.058260	-0.519345
C	-3.811867	-0.908795	-1.535441
C	-4.140599	1.387933	-0.719197
C	-4.407756	-0.528211	-2.738922
C	-4.711146	1.713002	-1.947395
C	-4.858242	0.763532	-2.946380
H	-4.496512	-1.257073	-3.534037
H	-5.035974	2.728992	-2.129982
H	-5.307454	1.037349	-3.893074
C	-3.234405	-2.308153	-1.422676
H	-2.808212	-2.443175	-0.433540
C	-3.954767	2.495334	0.311510
H	-3.614172	2.044256	1.242077
C	-2.087310	-2.525614	-2.422896
H	-2.437635	-2.470972	-3.456058
H	-1.304321	-1.774264	-2.309644
H	-1.660006	-3.515544	-2.253327
C	-4.290161	-3.405579	-1.618919
H	-5.173224	-3.238133	-1.000968
H	-4.621928	-3.455265	-2.659052
H	-3.855549	-4.367404	-1.343437
C	-2.865529	3.488970	-0.126572
H	-2.749629	4.275305	0.622941
H	-1.903525	2.992966	-0.243107
H	-3.125498	3.963592	-1.075183
C	-5.265264	3.241938	0.605050
H	-6.073748	2.556614	0.860524
H	-5.124332	3.934340	1.437982
H	-5.589075	3.829951	-0.255668
B	0.791189	0.857871	0.328193
B	-0.719011	0.398477	0.100219
C	2.028324	1.546083	0.203794
C	2.111481	3.071033	0.427151
C	3.638167	3.318644	0.475849
C	4.319231	2.167272	-0.285292
H	3.969921	3.293518	1.516113
H	3.909141	4.294743	0.069960
N	3.322682	1.083490	-0.092089
C	3.605153	-0.305774	-0.195207
C	3.990250	-1.017705	0.967935
C	3.473032	-0.986005	-1.427047
C	4.289859	-2.377107	0.860507
C	3.805120	-2.344343	-1.489020
C	4.218786	-3.036690	-0.359711
H	4.593509	-2.927316	1.742445
H	3.732555	-2.867264	-2.435003
H	4.478719	-4.086289	-0.427864
C	1.437815	3.832274	-0.729760
H	1.579333	4.911492	-0.615612
H	1.834703	3.537367	-1.699563
H	0.366391	3.627718	-0.740452
C	1.454319	3.511138	1.743025
H	1.870045	2.961183	2.588818
H	1.612110	4.580851	1.912141
H	0.378893	3.327013	1.721379
C	4.539135	2.516882	-1.770382
H	3.603453	2.730405	-2.282268
H	5.178797	3.398068	-1.855616
H	5.033557	1.696447	-2.289181
C	5.687917	1.821763	0.309270
H	6.090570	0.909443	-0.135114
H	6.390484	2.630641	0.101550
H	5.640807	1.688820	1.387164
C	2.924761	-0.312742	-2.674455
H	2.753659	0.732363	-2.431001
C	4.064871	-0.355102	2.334901
H	3.933113	0.714044	2.183509
C	2.910748	-0.818005	3.233420
H	2.971218	-0.338295	4.213022
H	1.958461	-0.542921	2.785119
H	2.931880	-1.900162	3.388328

C	5.416334	-0.584866	3.026595
H	5.476955	0.015085	3.937142
H	5.546659	-1.629688	3.316464
H	6.252326	-0.313173	2.380981
C	1.562454	-0.908629	-3.060203
H	1.646483	-1.963983	-3.332803
H	0.858550	-0.812208	-2.233636
H	1.134761	-0.379460	-3.913182
C	3.894567	-0.390419	-3.861578
H	4.012504	-1.418343	-4.211628
H	3.515992	0.200544	-4.698251
H	4.884872	-0.015478	-3.601152
N	-1.071708	0.712672	-1.296351
N	-1.205093	1.004582	-2.365979
K	0.693706	-2.815588	-0.002570

TS_{KCl}

110

Cl	-0.722311	-4.525396	0.074878
C	-3.095544	-0.782300	2.736487
C	-3.835413	-1.205544	1.465388
C	-1.761220	-0.160362	0.826361
C	-1.635438	-0.478412	2.333450
H	-3.152601	-1.545327	3.512625
H	-3.555414	0.125757	3.132341
N	-3.002591	-0.495570	0.420786
C	-3.822252	-2.725735	1.260335
H	-4.396025	-3.000806	0.377540
H	-2.822550	-3.142267	1.150674
H	-4.297858	-3.200413	2.120969
C	-0.721109	-1.686631	2.593682
H	-1.004783	-2.569324	2.020778
H	0.309575	-1.429281	2.350984
H	-0.746355	-1.945807	3.654719
C	-1.114536	0.729066	3.129273
H	-1.170157	0.510465	4.199421
H	-0.082843	0.959250	2.869070
H	-1.714960	1.618246	2.928298
C	-5.288601	-0.732556	1.463726
H	-5.769642	-0.925121	0.503967
H	-5.833833	-1.287225	2.228622
H	-5.378558	0.326629	1.691998
C	-3.553251	-0.130115	-0.855927
C	-3.489480	-1.019579	-1.944609
C	-4.129754	1.152735	-0.986437
C	-4.060295	-0.615782	-3.152573
C	-4.668390	1.507525	-2.220351
C	-4.645557	0.630392	-3.294408
H	-4.024091	-1.286857	-4.001110
H	-5.105029	2.489580	-2.347453
H	-5.074156	0.925157	-4.244483
C	-2.764243	-2.351803	-1.889253
H	-2.381479	-2.509984	-0.885881
C	-4.132036	2.184126	0.135673
H	-3.818526	1.688652	1.052858
C	-1.544197	-2.367558	-2.824615
H	-1.836321	-2.269900	-3.873089
H	-0.860071	-1.547670	-2.601249
H	-1.015758	-3.313799	-2.696763
C	-3.676898	-3.539844	-2.224523
H	-4.603674	-3.514344	-1.650706
H	-3.945374	-3.549308	-3.284123
H	-3.152810	-4.467281	-1.988974
C	-3.115020	3.306261	-0.131656
H	-3.149022	4.045500	0.672660
H	-2.101837	2.911518	-0.179961
H	-3.334161	3.820182	-1.070371
C	-5.527097	2.780044	0.378218
H	-6.282540	2.004255	0.507934
H	-5.517881	3.403089	1.275283
H	-5.841236	3.412411	-0.454309
B	0.744826	1.161666	0.220576
B	-0.611581	0.453832	0.114811
C	1.865342	2.045771	0.114692
C	1.696296	3.562585	0.302564
C	3.156316	4.043730	0.461813
C	4.062093	3.011423	-0.233435
H	3.406655	4.072611	1.524334
H	3.305512	5.048842	0.063749
N	3.203292	1.794703	-0.167456
C	3.687339	0.468313	-0.363000
C	4.141931	-0.272286	0.754001
C	3.682204	-0.117044	-1.648876

C	4.651550	-1.556452	0.547373
C	4.216020	-1.399985	-1.806293
C	4.709648	-2.112925	-0.723305
H	5.011821	-2.128707	1.392998
H	4.236528	-1.849905	-2.791149
H	5.126844	-3.102517	-0.866154
C	1.015584	4.176577	-0.935711
H	0.993129	5.267469	-0.859574
H	1.528497	3.910899	-1.859053
H	-0.010420	3.817703	-1.018014
C	0.865851	3.913347	1.543457
H	1.297730	3.465460	2.439612
H	0.824470	4.997889	1.681046
H	-0.155085	3.542350	1.444355
C	4.395128	3.411446	-1.681476
H	3.501893	3.557491	-2.284288
H	4.961692	4.344722	-1.687236
H	5.009321	2.647002	-2.156682
C	5.391737	2.836687	0.508335
H	5.966529	2.003907	0.098368
H	5.990401	3.741855	0.395937
H	5.244280	2.665139	1.571761
C	3.042781	0.556085	-2.852504
H	2.720744	1.546505	-2.542324
C	4.051822	0.259594	2.176449
H	3.754284	1.303883	2.117122
C	2.945923	-0.464475	2.956280
H	2.883541	-0.083714	3.978296
H	1.983264	-0.292171	2.477834
H	3.131711	-1.540684	3.012028
C	5.389991	0.176381	2.924561
H	5.311290	0.681716	3.889500
H	5.675759	-0.858857	3.121962
H	6.198375	0.642811	2.360297
C	1.781987	-0.210786	-3.277819
H	2.022822	-1.218591	-3.625463
H	1.086558	-0.283962	-2.442784
H	1.268770	0.307644	-4.089641
C	4.003690	0.702671	-4.040170
H	4.276758	-0.270084	-4.455177
H	3.529613	1.277822	-4.838426
H	4.925183	1.211790	-3.757294
N	-1.163517	0.931590	-1.700497
N	-1.095138	1.451095	-2.666861
K	1.143372	-2.422363	-0.239141

$RC_{LiClTHF}$

136

C	-0.151265	-5.183695	-0.646236
C	-1.589944	-4.646933	-0.562389
C	-0.050317	-2.851985	-0.090965
C	0.774433	-4.142032	0.020893
H	0.129257	-5.287289	-1.695635
H	-0.063601	-6.167907	-0.184529
N	-1.341956	-3.179975	-0.370432
C	-2.368784	-4.934458	-1.848041
H	-3.358864	-4.478097	-1.819243
H	-1.840149	-4.570658	-2.726832
H	-2.497869	-6.013199	-1.954259
C	2.113835	-4.046956	-0.721135
H	2.750831	-3.278840	-0.285910
H	2.636720	-5.006448	-0.672948
H	1.956520	-3.788196	-1.768174
C	1.042786	-4.472894	1.501949
H	1.647077	-3.689387	1.959082
H	0.119098	-4.558095	2.073590
H	1.579606	-5.422172	1.581659
C	-2.377130	-5.248045	0.609918
H	-2.530338	-6.313846	0.432968
H	-1.856434	-5.135933	1.558126
H	-3.356581	-4.776669	0.695186
C	-2.421987	-2.237529	-0.300610
C	-2.905262	-1.649050	-1.484524
C	-2.994931	-1.914199	0.949439
C	-4.038259	-0.836182	-1.414263
C	-4.122176	-1.093553	0.967808
C	-4.660481	-0.580689	-0.202476
H	-4.416046	-0.360519	-2.308371
H	-4.573823	-0.833374	1.916519
H	-5.537627	0.053189	-0.169278
C	-2.165962	-1.758631	-2.806773
H	-1.383139	-2.504517	-2.689533
C	-2.385948	-2.336641	2.277323

H	-1.565587	-3.018253	2.070720
C	-1.474329	-0.419301	-3.099598
H	-0.805309	-0.155631	-2.280772
H	-0.882808	-0.478051	-4.015633
H	-2.210359	0.380055	-3.220773
C	-3.060727	-2.173923	-3.980614
H	-2.456529	-2.310436	-4.880822
H	-3.585527	-3.108507	-3.779067
H	-3.807342	-1.408448	-4.200509
C	-1.779481	-1.112815	2.983815
H	-2.557183	-0.399268	3.265027
H	-1.254970	-1.419802	3.892301
H	-1.065947	-0.614947	2.327784
C	-3.381760	-3.049071	3.202965
H	-3.843662	-3.909439	2.718484
H	-2.869997	-3.400535	4.102280
H	-4.179945	-2.374883	3.521220
B	1.389075	-0.363839	0.466807
B	0.557610	-1.552290	0.125641
C	2.232609	0.700202	0.978301
C	1.984878	1.414139	2.315586
C	3.226781	2.329792	2.455756
C	3.840053	2.472955	1.049565
H	3.957420	1.860049	3.117224
H	2.973833	3.299287	2.885902
N	3.366807	1.209054	0.406881
C	4.121812	0.448427	-0.547197
C	4.998463	-0.551573	-0.067359
C	3.961543	0.664039	-1.924961
C	5.718227	-1.300415	-0.995803
C	4.717453	-0.102395	-2.815102
C	5.590427	-1.075323	-2.359542
H	6.388057	-2.077383	-0.649279
H	4.602959	0.052668	-3.880834
H	6.164170	-1.666130	-3.063823
C	0.668982	2.210465	2.286390
H	0.647468	2.942494	1.480132
H	-0.172797	1.538651	2.127256
H	0.526620	2.738725	3.233278
C	1.920151	0.396478	3.466738
H	1.075101	-0.279240	3.335395
H	2.828538	-0.206133	3.508446
H	1.806710	0.915872	4.422688
C	3.269899	3.700321	0.321966
H	3.509744	4.607959	0.879497
H	3.690799	3.797843	-0.678064
H	2.187910	3.631448	0.234994
C	5.362255	2.585687	1.075810
H	5.821804	1.770547	1.628837
H	5.774502	2.593743	0.065216
H	5.640576	3.522610	1.561288
C	2.946070	1.643704	-2.480880
H	2.454160	2.118370	-1.635650
C	5.162031	-0.867493	1.412762
H	4.684159	-0.071904	1.979310
C	4.441441	-2.171761	1.785172
H	3.380446	-2.097027	1.552409
H	4.861361	-3.019151	1.237564
H	4.549624	-2.372511	2.854603
C	6.634086	-0.924305	1.847173
H	7.180741	-0.028287	1.547177
H	6.698096	-1.015754	2.934069
H	7.146090	-1.786326	1.414920
C	1.865801	0.898422	-3.276747
H	1.082395	1.587274	-3.599573
H	2.284479	0.427239	-4.168310
H	1.410462	0.120714	-2.664026
C	3.597871	2.742991	-3.330836
H	4.371244	3.275883	-2.774210
H	4.062116	2.326160	-4.227526
H	2.847210	3.469473	-3.651257
N	0.779597	-3.174741	-4.134901
N	1.209746	-2.348492	-4.702137
Li	-3.083626	2.029303	-0.458781
Cl	-4.259732	2.619216	-2.238188
H	-0.982690	2.223785	-2.241398
O	-1.439521	3.014083	-0.385094
C	-1.672994	4.441062	-0.296432
C	-1.145994	5.016218	-1.601831
C	0.047451	4.099208	-1.892804
C	-0.467734	2.740132	-1.430547
H	-2.740765	4.602263	-0.149520

H	-1.127704	4.829681	0.569619
H	-0.869671	6.066671	-1.512145
H	-1.904721	4.912449	-2.379653
H	0.916097	4.409575	-1.310146
H	0.333295	4.089187	-2.944058
H	0.290101	2.079011	-1.011841
O	-3.858427	2.295920	1.250023
C	-3.177705	2.721347	2.448699
C	-3.996264	3.900461	3.015854
C	-5.197610	4.024620	2.054478
C	-5.245031	2.657865	1.386257
H	-3.139527	1.883039	3.148723
H	-5.753971	1.915388	2.011865
H	-2.161231	2.984112	2.164133
H	-5.680571	2.658046	0.388715
H	-5.009923	4.789868	1.299748
H	-6.127240	4.270635	2.567258
H	-3.410894	4.818608	3.049473
H	-4.326697	3.678863	4.030795

Int$_{LiClTHF}$

136

Li	-3.144760	1.524471	0.167284
Cl	-5.312353	1.320686	-0.450429
C	0.358469	-5.127643	-1.451702
C	-0.634423	-4.987972	-0.291983
C	0.181252	-2.801632	-0.855760
C	1.111061	-3.781558	-1.569811
H	-0.193638	-5.314490	-2.374293
H	1.038352	-5.965247	-1.300251
N	-0.777790	-3.483587	-0.230448
C	-1.961895	-5.688537	-0.573725
H	-2.369252	-5.413749	-1.543399
H	-1.792650	-6.766355	-0.575923
H	-2.701357	-5.464714	0.196116
C	1.294517	-3.401525	-3.046519
H	0.330999	-3.262244	-3.540798
H	1.864857	-2.479893	-3.136408
H	1.833343	-4.201126	-3.561867
C	2.491746	-3.827565	-0.892345
H	2.423031	-4.126415	0.151880
H	3.123075	-4.552720	-1.411615
H	2.966358	-2.848040	-0.937061
C	-0.075046	-5.528560	1.029130
H	0.081027	-6.603867	0.933325
H	0.877160	-5.071654	1.291722
H	-0.778528	-5.361490	1.842619
C	-1.846396	-2.834435	0.487922
C	-3.031334	-2.532497	-0.212366
C	-1.654865	-2.427841	1.821969
C	-4.045610	-1.866549	0.471903
C	-2.709923	-1.773575	2.460043
C	-3.894461	-1.497827	1.799228
H	-4.944320	-1.574492	-0.052122
H	-2.584766	-1.440031	3.482301
H	-4.685665	-0.955064	2.300269
C	-3.218948	-2.799723	-1.700461
H	-2.376376	-3.390210	-2.055357
C	-0.338344	-2.564101	2.572889
H	0.373916	-3.075792	1.929811
C	-3.214462	-1.486621	-2.504929
H	-3.987152	-0.801324	-2.149828
H	-2.250408	-0.983031	-2.430573
H	-3.400023	-1.698553	-3.560795
C	-4.503719	-3.592121	-1.985502
H	-4.547795	-3.866687	-3.041887
H	-4.553470	-4.505629	-1.391846
H	-5.391184	-2.997459	-1.760859
C	0.260741	-1.185459	2.895671
H	0.466311	-0.611865	1.993436
H	-0.409472	-0.601869	3.528422
H	1.204367	-1.302801	3.427940
C	-0.489192	-3.381050	3.865519
H	0.490622	-3.551780	4.317368
H	-1.103073	-2.848221	4.595028
H	-0.955167	-4.350414	3.686345
B	1.632777	-0.399808	-1.323962
B	0.379190	-1.290217	-0.900310
C	2.645614	0.544719	-1.631265
C	2.964147	1.080156	-3.043838
C	4.190723	1.997859	-2.790127
C	4.775532	1.603402	-1.415881
H	3.868465	3.040404	-2.750339

H	4.931123	1.917785	-3.588215
N	3.559613	1.119858	-0.725019
C	3.213503	1.361850	0.636069
C	2.433070	2.496485	0.965044
C	3.610806	0.467950	1.650351
C	2.071157	2.706485	2.294302
C	3.253094	0.737597	2.973362
C	2.482517	1.840977	3.298289
H	1.464166	3.565243	2.553184
H	3.562173	0.057639	3.757465
H	2.195985	2.023684	4.327267
C	3.297551	-0.044020	-4.038483
H	4.099456	-0.683845	-3.671234
H	2.422503	-0.673130	-4.209481
H	3.604371	0.376416	-5.001023
C	1.784630	1.887434	-3.614661
H	1.496900	2.695294	-2.942510
H	2.046679	2.323918	-4.584173
H	0.912616	1.244239	-3.754550
C	5.825964	0.485548	-1.577412
H	6.292850	0.245476	-0.623239
H	5.368684	-0.422191	-1.969992
H	5.800187	-0.700678	3.007271
C	5.420925	2.784900	-0.695208
H	5.752281	2.498986	0.304975
H	6.294243	3.122511	-1.256506
H	4.732795	3.623293	-0.599044
C	4.394448	-0.795672	1.346875
H	4.455561	-0.881829	0.263235
C	1.961758	3.482662	-0.088587
H	2.502779	3.259844	-1.004308
C	0.470871	3.297615	-0.385294
H	0.146797	3.973302	-1.179826
H	0.263035	2.278315	-0.703701
H	-0.136657	3.511304	0.494778
C	2.262808	4.941484	0.282597
H	1.671516	5.270425	1.140373
H	3.316454	5.085238	0.530529
H	2.016831	5.598066	-0.555551
C	3.674928	-2.047853	1.867342
H	2.658380	-2.096642	1.480314
H	4.206378	-2.947831	1.549684
H	3.624773	-2.058822	2.958253
C	5.819544	-0.723250	1.914607
H	6.612709	0.801155	-2.266851
H	6.402236	-1.595359	1.606807
H	6.341659	0.173218	1.575913
N	-0.676269	-0.438942	-0.414489
N	-1.455119	0.301136	-0.084522
H	-0.846908	1.878106	2.088411
O	-2.894767	2.019198	2.007727
C	-3.874363	2.789745	2.722957
C	-3.133544	4.060533	3.155117
C	-1.643003	3.629568	3.192203
C	-1.668772	2.163784	2.740234
H	-4.715198	2.954674	2.052402
H	-4.215883	2.211594	3.589188
H	-3.488761	4.420262	4.120158
H	-3.284521	4.855395	2.424970
H	-1.202800	3.729396	4.184015
H	-1.051449	4.235151	2.505530
H	-1.689378	1.484166	3.598789
H	-2.905189	1.630483	-2.556710
O	-2.486800	2.929646	-1.003858
C	-2.985792	4.270814	-0.822387
C	-2.901824	4.925062	-2.195815
C	-3.159670	3.734579	-3.127170
C	-2.432316	2.602235	-2.411085
H	-4.019678	4.213281	-0.468301
H	-2.370519	4.758350	-0.065117
H	-1.901621	5.332258	-2.363075
H	-3.625690	5.730892	-2.314710
H	-2.787982	3.890043	-4.139840
H	-4.228623	3.518263	-3.175937
H	-1.380558	2.537021	-2.704194

XYZ Coordinates at PBE/TZVP/D3/(implicit THF: e= 7.58) level of theory

$LiCl[THF]_4$

54

Li	0.390080	0.306959	-0.148682

Cl	-0.049895	0.434300	-2.381043
O	0.467356	-1.520520	0.549192
O	-0.809652	1.458790	0.933098
C	0.702949	-2.687290	-0.272840
C	1.733142	-3.500393	0.513951
C	1.404621	-3.166000	1.995192
C	0.373598	-2.021584	1.901765
C	-0.326895	2.149846	2.109125
C	-0.111727	3.612529	1.676170
C	-0.943294	3.747391	0.370969
C	-1.675407	2.405886	0.262800
H	-0.246886	-3.238636	-0.387175
H	1.037235	-2.331295	-1.255609
H	1.658139	-4.573638	0.295109
H	2.751337	-3.173493	0.258841
H	0.979617	-4.027980	2.526296
H	2.305858	-2.850268	2.537833
H	0.570695	-1.181308	2.580623
H	-0.651846	-2.391306	2.072230
H	0.591755	1.644650	2.432771
H	-1.088212	2.071608	2.905618
H	-0.449952	4.307103	2.456683
H	0.951368	3.810640	1.485328
H	-1.645371	4.591243	0.401178
H	-0.283913	3.885757	-0.496966
H	-1.810124	2.045275	-0.765008
H	-2.649809	2.432822	0.783676
H	3.190128	2.886058	-0.150988
O	2.200876	1.104214	0.297911
C	3.344221	0.257999	0.552918
C	3.955063	0.003923	-0.827789
C	3.614900	1.297673	-1.616640
C	2.666298	2.070663	-0.680341
H	4.049702	0.792641	1.215197
H	2.980068	-0.641612	1.063805
H	3.475600	-0.866825	-1.296853
H	5.034516	-0.189262	-0.769056
H	3.106466	1.055762	-2.558728
H	4.511184	1.890263	-1.844919
H	1.779292	2.466882	-1.190149
H	-3.178692	-2.856981	-1.853582
O	-2.857418	-2.447676	0.140016
C	-3.172003	-1.278863	0.920001
C	-4.228963	-0.545949	0.095605
C	-3.719330	-0.755369	-1.346699
C	-2.824150	-2.013617	-1.243717
H	-3.522792	-1.620983	1.903920
H	-2.273177	-0.647872	1.054023
H	-4.318460	0.515282	0.367394
H	-5.210706	-1.022248	0.237837
H	-3.112388	0.097487	-1.682009
H	-4.544890	-0.888240	-2.058716
H	-1.789656	-1.763914	-1.536199

$RC_{LiCl[THF]4}$

162

C	6.736842	-2.308997	0.386194
C	5.656915	-3.303447	-0.088786
C	4.655457	-1.113854	0.209180
C	6.172437	-0.880225	0.187087
H	7.686955	-2.457693	-0.146661
H	6.926101	-2.473996	1.458378
N	4.421895	-2.452401	0.037375
C	5.883171	-3.780274	-1.534341
H	6.809193	-4.371685	-1.579660
H	5.056058	-4.423945	-1.864802
H	5.980457	-2.946481	-2.240403
C	6.590551	-0.281279	-1.172824
H	6.135373	0.708195	-1.308879
H	7.686482	-0.178885	-1.220342
H	6.267912	-0.911264	-2.012714
C	6.622174	0.057619	1.317325
H	6.325019	-0.345364	2.296154
H	7.717243	0.178665	1.301684
H	6.156393	1.046965	1.208915
C	5.593794	-4.544953	0.808697
H	6.508259	-5.138417	0.666705
H	5.527037	-4.274748	1.869466
H	4.734753	-5.180976	0.549243
C	3.086899	-2.977615	-0.020663
C	2.401729	-3.028592	-1.259634
C	2.457010	-3.393831	1.178821
C	1.118712	-3.596117	-1.285893

C	1.179610	-3.964689	1.093803
C	0.516475	-4.079648	-0.126422
H	0.579245	-3.649423	-2.234133
H	0.690411	-4.311696	2.006629
H	-0.481521	-4.521036	-0.166862
C	2.973915	-2.431362	-2.537975
H	4.011110	-2.139808	-2.331387
C	3.075350	-3.166850	2.552135
H	4.102362	-2.808784	2.399265
C	2.220051	-1.145552	-2.918408
H	2.273608	-0.407730	-2.105462
H	2.666874	-0.693841	-3.817711
H	1.160424	-1.355620	-3.133226
C	2.976152	-3.428341	-3.707692
H	3.489247	-4.365743	-3.447205
H	1.951610	-3.683478	-4.019647
H	3.486320	-2.989621	-4.578580
C	2.321650	-2.047685	3.294896
H	2.821165	-1.817919	4.249044
H	2.299315	-1.132564	2.684931
H	1.287582	-2.353480	3.519464
C	3.122528	-4.443938	3.405625
H	3.631910	-5.266438	2.883867
H	3.657718	-4.251341	4.348007
H	2.110077	-4.787910	3.666919
B	2.809756	1.157125	0.639052
B	3.672600	-0.040220	0.383910
C	2.143418	2.383649	1.085925
C	1.896190	2.709115	2.565266
C	1.584224	4.225846	2.523656
C	1.052920	4.545778	1.109811
H	2.518085	4.786735	2.687235
H	0.872021	4.527515	3.304501
N	1.677392	3.424448	0.322207
C	1.938650	3.433207	-1.093682
C	3.204188	3.876691	-1.558305
C	0.982135	2.907351	-1.993520
C	3.471128	3.806426	-2.932506
C	1.301624	2.851684	-3.358119
C	2.533851	3.296759	-3.829390
H	4.440950	4.144635	-3.304709

H	0.572599	2.441750	-4.060822
H	2.766377	3.243674	-4.895484
C	0.719475	1.873061	3.105969
H	0.480997	2.165459	4.140858
H	-0.187348	1.992148	2.497860
H	0.985481	0.807792	3.092705
C	3.140677	2.416108	3.418919
H	3.396771	1.347458	3.367177
H	4.007000	2.984896	3.051112
H	2.957382	2.689611	4.470601
C	-0.487575	4.518082	1.063161
H	-0.880022	5.284720	1.747138
H	-0.861937	4.744708	0.056181
H	-0.894795	3.548775	1.377306
C	1.509678	5.919730	0.611440
H	2.589808	6.058868	0.734563
H	1.254045	6.061180	-0.449274
H	0.997876	6.702570	1.189428
C	-0.379925	2.406434	-1.543061
H	-0.435783	2.543522	-0.456622
C	4.297229	4.392265	-0.631602
H	3.888112	4.417236	0.387815
C	5.493544	3.427423	-0.606580
H	5.152124	2.425171	-0.314421
H	5.975239	3.364056	-1.595328
H	6.249145	3.769103	0.118280
C	4.753012	5.812082	-1.010131
H	3.908063	6.513669	-1.064010
H	5.469899	6.191859	-0.265968
H	5.256463	5.819562	-1.988778
C	-0.567672	0.906994	-1.814425
H	-0.466163	0.675903	-2.885642
H	0.176711	0.314629	-1.265197
H	-1.572225	0.582240	-1.503930
C	-1.501795	3.231279	-2.198903
H	-1.338681	4.311916	-2.074031
H	-1.563196	3.026439	-3.278802
H	-2.479065	2.983351	-1.760507
N	-4.942510	1.520585	-3.402001
N	-4.859869	1.880897	-2.361519
Li	-3.825266	-1.357594	0.391631

Cl	-5.613066	-2.683166	0.916115
H	-3.096998	1.676343	-0.135051
O	-4.136106	0.449668	1.139832
C	-5.099969	0.715776	2.187109
C	-5.660672	2.090743	1.833604
C	-4.441557	2.825501	1.220260
C	-3.440828	1.693345	0.905535
H	-5.824071	-0.108675	2.165987
H	-4.582974	0.724857	3.165401
H	-6.065034	2.608784	2.713472
H	-6.464963	1.986400	1.091598
H	-4.000008	3.540952	1.927613
H	-4.722465	3.378601	0.314487
H	-2.559902	1.746247	1.571040
O	-2.134666	-2.051992	1.233961
C	-1.174678	-1.094754	1.749278
C	-1.531388	-0.904797	3.236962
C	-2.447216	-2.114111	3.564787
C	-2.344212	-2.991365	2.316303
H	-0.159581	-1.503836	1.619153
H	-1.479242	-3.675688	2.371191
H	-1.257579	-0.185148	1.142536
H	-3.256127	-3.554667	2.082280
H	-3.488533	-1.788619	3.700062
H	-2.135491	-2.649544	4.471743
H	-2.057158	0.045153	3.404583
H	-0.625945	-0.894995	3.856876
C	-2.274450	-2.183315	-1.985130
O	-3.404635	-1.378039	-1.567641
C	-4.508724	-1.825556	-2.399408
C	-2.849707	-3.591745	-2.126520
H	-1.893630	-1.797797	-2.948483
H	-1.496824	-2.087235	-1.219541
C	-4.303436	-3.339866	-2.606380
H	-2.269515	-4.210119	-2.824871
H	-2.852930	-4.086013	-1.144734
H	-4.439538	-3.610858	-3.662068
H	-5.018723	-3.912009	-2.002205
H	-5.434330	-1.581233	-1.863463
H	-4.475021	-1.276219	-3.355639
C	-7.705010	0.208478	-0.958086
O	-8.365007	1.263893	-0.219719
C	-9.126440	0.665135	0.865404
C	-8.522078	-1.052040	-0.699734
H	-7.669740	0.513374	-2.014162
H	-6.670501	0.080179	-0.585806
C	-8.921779	-0.852262	0.769697
H	-9.406634	-1.081677	-1.354671
H	-7.928162	-1.965436	-0.840946
H	-9.824202	-1.411620	1.051873
H	-8.091691	-1.183349	1.411756
H	-8.771850	1.080037	1.823335
H	-10.184005	0.949441	0.733388

$TS_{1LiCl[THF]4}$

162

C	-2.916882	-1.210152	2.726957
C	-3.659074	-1.585865	1.432812
C	-1.706415	-0.295749	0.845598
C	-1.508457	-0.712656	2.319252
H	-2.869676	-2.054610	3.428839
H	-3.460132	-0.392651	3.225785
N	-2.946763	-0.702000	0.430666
C	-3.478557	-3.080453	1.108154
H	-4.064207	-3.378683	0.230572
H	-2.425543	-3.333406	0.927861
H	-3.831911	-3.676799	1.962342
C	-0.424821	-1.803061	2.444594
H	-0.662835	-2.689218	1.840114
H	0.539746	-1.411468	2.093098
H	-0.325032	-2.117252	3.495681
C	-1.104362	0.492017	3.190101
H	-1.026808	0.183148	4.245218
H	-0.139669	0.897670	2.859179
H	-1.850588	1.297672	3.117554
C	-5.152978	-1.269859	1.503192
H	-5.650161	-1.459571	0.540941
H	-5.620658	-1.914956	2.260696
H	-5.331469	-0.225212	1.787758
C	-3.622777	-0.113726	-0.695400
C	-3.762463	-0.822606	-1.912766
C	-4.138501	1.204116	-0.556679
C	-4.459828	-0.211762	-2.965610

C	-4.816722	1.769071	-1.644510
C	-4.987938	1.069582	-2.837751
H	-4.576859	-0.748091	-3.910002
H	-5.223793	2.778783	-1.553433
H	-5.527185	1.527503	-3.668929
C	-3.158368	-2.199385	-2.141384
H	-2.622496	-2.480055	-1.225117
C	-3.975236	2.038991	0.707658
H	-3.546413	1.392553	1.485592
C	-2.129302	-2.196855	-3.285575
H	-2.596274	-1.928873	-4.245677
H	-1.314990	-1.488474	-3.093132
H	-1.688725	-3.199372	-3.396716
C	-4.249736	-3.249005	-2.420571
H	-5.035328	-3.241968	-1.650587
H	-4.737263	-3.055993	-3.388756
H	-3.813323	-4.258407	-2.458981
C	-2.988604	3.200292	0.484150
H	-2.854686	3.769552	1.417209
H	-2.005015	2.822574	0.171920
H	-3.366416	3.893496	-0.283112
C	-5.319201	2.576759	1.230268
H	-6.059494	1.774613	1.364033
H	-5.173297	3.074339	2.201028
H	-5.751626	3.318342	0.541212
B	0.806735	1.002596	0.212214
B	-0.605695	0.428748	0.180833
C	2.058596	1.716425	0.060834
C	2.168230	3.250536	0.119322
C	3.697401	3.494218	-0.015512
C	4.317997	2.202233	-0.593637
H	4.128512	3.680198	0.980607
H	3.922369	4.369080	-0.642649
N	3.325839	1.188047	-0.120207
C	3.597155	-0.189017	0.175094
C	3.959884	-0.533686	1.504852
C	3.425317	-1.193337	-0.805743
C	4.190318	-1.881387	1.810054
C	3.671460	-2.529061	-0.450781
C	4.054186	-2.875812	0.841930
H	4.470240	-2.159375	2.828725

H	3.544516	-3.312856	-1.201420
H	4.238965	-3.921513	1.098855
C	1.356997	3.909352	-1.007899
H	1.455261	5.005984	-0.963588
H	1.677642	3.582602	-2.004824
H	0.291996	3.652077	-0.905344
C	1.647645	3.775769	1.471258
H	2.172782	3.292034	2.308022
H	1.798251	4.865713	1.546181
H	0.573410	3.561055	1.577324
C	4.373073	2.256713	-2.134509
H	3.368798	2.376724	-2.563113
H	4.989222	3.109987	-2.456430
H	4.818126	1.342599	-2.549687
C	5.726937	1.930152	-0.065463
H	6.081395	0.938353	-0.382770
H	6.419665	2.682316	-0.469534
H	5.769392	1.982197	1.029448
C	2.955009	-0.890106	-2.218748
H	2.796420	0.194298	-2.287303
C	4.054473	0.499218	2.619111
H	3.974696	1.491941	2.155134
C	2.866228	0.359145	3.585638
H	2.899045	1.146374	4.355615
H	1.919254	0.447598	3.036967
H	2.881986	-0.617422	4.094322
C	5.388966	0.431199	3.379009
H	5.455834	1.257417	4.103436
H	5.483881	-0.509796	3.942064
H	6.249887	0.501977	2.698187
C	1.604039	-1.564205	-2.511549
H	1.691214	-2.661956	-2.504799
H	0.856305	-1.269893	-1.760723
H	1.236290	-1.264804	-3.505331
C	4.011566	-1.302643	-3.257452
H	4.176287	-2.390833	-3.241597
H	3.686518	-1.029592	-4.271676
H	4.980152	-0.816712	-3.067006
N	-1.177493	0.808314	-1.773149
N	-1.045735	1.381356	-2.724782
Li	-0.249390	4.344731	-5.471708

Cl	-0.235762	5.687372	-7.335624
O	0.627589	2.632888	-5.928632
O	-2.055675	4.440447	-4.641616
C	1.072388	2.314565	-7.272277
C	2.561694	2.029257	-7.107785
C	2.635852	1.342542	-5.723096
C	1.289729	1.694305	-5.050168
C	-2.332478	4.299072	-3.222947
C	-3.131066	5.552762	-2.800561
C	-3.281035	6.364187	-4.107096
C	-3.084455	5.302861	-5.182598
H	0.516407	1.431722	-7.633380
H	0.835132	3.185494	-7.896432
H	2.954607	1.396261	-7.914698
H	3.126837	2.972103	-7.106915
H	2.734868	0.254042	-5.828508
H	3.493069	1.699407	-5.136472
H	1.385923	2.180099	-4.069742
H	0.652758	0.801637	-4.930761
H	-1.364101	4.195578	-2.716210
H	-2.909435	3.374750	-3.060767
H	-4.115314	5.262771	-2.406333
H	-2.615598	6.125692	-2.018326
H	-4.253936	6.867399	-4.185011
H	-2.491095	7.124774	-4.190398
H	-2.711203	5.677854	-6.143828
H	-4.010226	4.718346	-5.341566
H	0.059270	6.274641	-2.678289
O	1.009152	5.146246	-4.142032
C	2.401186	5.195862	-4.571382
C	2.800212	6.667437	-4.536409
C	1.931860	7.206694	-3.390713
C	0.628127	6.445550	-3.603221
H	3.002179	4.591893	-3.869061
H	2.462427	4.750142	-5.573949
H	2.527861	7.153848	-5.485131
H	3.876742	6.803195	-4.366694
H	1.787601	8.294323	-3.432090
H	2.375535	6.950954	-2.415682
H	-0.017444	6.950491	-4.342008
H	-2.345845	2.134099	-9.246173
O	-2.030066	0.954008	-7.586460
C	-2.502077	1.006794	-6.224493
C	-3.873062	1.712953	-6.277968
C	-3.859731	2.444049	-7.646428
C	-2.429058	2.217137	-8.154191
H	-2.543420	-0.023883	-5.846800
H	-1.792309	1.585890	-5.608145
H	-3.978310	2.407774	-5.435075
H	-4.700462	0.992216	-6.223007
H	-4.094847	3.513602	-7.559700
H	-4.587832	1.992023	-8.334473
H	-1.756708	3.024454	-7.805722

Int$_{1LiCl[THF]4}$

162

C	-2.636682	-1.175282	2.624149
C	-3.529978	-1.542813	1.426179
C	-1.681784	-0.216428	0.624212
C	-1.308472	-0.615974	2.052412
H	-2.471761	-2.037588	3.284146
H	-3.134025	-0.393659	3.218762
N	-2.930669	-0.654021	0.349835
C	-3.388181	-3.031808	1.069914
H	-4.042233	-3.315971	0.237472
H	-2.353226	-3.293546	0.812178
H	-3.682505	-3.630283	1.944420
C	-0.164582	-1.650901	2.054771
H	-0.421213	-2.546582	1.470954
H	0.743916	-1.213016	1.617910
H	0.055220	-1.963075	3.087615
C	-0.873610	0.622273	2.859874
H	-0.678831	0.332423	3.904872
H	0.035830	1.059998	2.426966
H	-1.659878	1.392411	2.854844
C	-5.005249	-1.223018	1.661709
H	-5.602080	-1.391263	0.753886
H	-5.392158	-1.884816	2.449549
H	-5.148788	-0.186287	1.989934
C	-3.690505	-0.131937	-0.761816
C	-3.820212	-0.863082	-1.964655
C	-4.251108	1.165688	-0.622452
C	-4.557644	-0.289121	-3.011077

C	-4.968189	1.692575	-1.704799
C	-5.130806	0.973121	-2.886957
H	-4.667116	-0.837616	-3.949039
H	-5.407026	2.689203	-1.620096
H	-5.695367	1.403344	-3.716077
C	-3.165799	-2.217448	-2.191588
H	-2.597521	-2.469180	-1.286688
C	-4.091738	2.019700	0.630682
H	-3.628105	1.398831	1.409633
C	-2.165461	-2.191424	-3.360817
H	-2.671677	-1.985703	-4.316365
H	-1.391305	-1.427636	-3.217948
H	-1.669401	-3.169767	-3.450356
C	-4.222560	-3.311788	-2.430808
H	-4.986303	-3.328483	-1.639239
H	-4.743275	-3.146447	-3.386463
H	-3.747642	-4.303385	-2.472994
C	-3.151135	3.213293	0.382404
H	-3.016886	3.789147	1.311002
H	-2.161825	2.874930	0.044719
H	-3.570317	3.891077	-0.376219
C	-5.445422	2.515158	1.169748
H	-6.154947	1.688804	1.319887
H	-5.301990	3.025722	2.133882
H	-5.911770	3.235863	0.480903
B	0.757788	1.081173	-0.031303
B	-0.721269	0.541984	-0.275617
C	2.027456	1.739876	0.010924
C	2.188115	3.263832	0.213041
C	3.724825	3.469551	0.057297
C	4.301948	2.172800	-0.562000
H	4.180474	3.621310	1.048605
H	3.959075	4.353723	-0.554268
N	3.317834	1.178780	-0.056784
C	3.549788	-0.197200	0.252622
C	3.908154	-0.543040	1.585281
C	3.345566	-1.217160	-0.709915
C	4.079750	-1.894117	1.915110
C	3.529176	-2.556846	-0.332268
C	3.893862	-2.899482	0.967170
H	4.353173	-2.165276	2.937851
H	3.374947	-3.346166	-1.072146
H	4.030709	-3.947878	1.242611
C	1.379860	4.062560	-0.821589
H	1.512271	5.146498	-0.667612
H	1.672686	3.824047	-1.852104
H	0.306656	3.832056	-0.725807
C	1.732423	3.682508	1.625017
H	2.241078	3.080979	2.392299
H	1.956924	4.746573	1.811652
H	0.648131	3.531376	1.740842
C	4.290480	2.268385	-2.103657
H	3.262363	2.382216	-2.475556
H	4.876599	3.139285	-2.435992
H	4.727538	1.371449	-2.562773
C	5.725709	1.870668	-0.096267
H	6.048154	0.876405	-0.440286
H	6.417454	2.614652	-0.517088
H	5.811282	1.904747	0.997079
C	2.940776	-0.916007	-2.143718
H	2.774962	0.167590	-2.214174
C	4.072425	0.502792	2.677509
H	3.995919	1.485959	2.192274
C	2.930801	0.408343	3.702841
H	3.023930	1.204120	4.458931
H	1.956937	0.512452	3.208434
H	2.946852	-0.560922	4.225582
C	5.438252	0.412185	3.377947
H	5.559519	1.246090	4.086374
H	5.534968	-0.524149	3.948993
H	6.268839	0.450952	2.657865
C	1.620183	-1.605477	-2.524537
H	1.716223	-2.702354	-2.512417
H	0.819582	-1.320282	-1.826605
H	1.313974	-1.310025	-3.539672
C	4.065095	-1.313937	-3.116362
H	4.237961	-2.400914	-3.091107
H	3.805402	-1.039544	-4.148959
H	5.014577	-0.820748	-2.859693
Li	-0.254277	4.322849	-5.438030
Cl	-0.213254	5.637668	-7.315787
O	0.581357	2.586891	-5.830771

O	-2.063031	4.417974	-4.631775
C	1.053214	2.216657	-7.150779
C	2.521622	1.872004	-6.930514
C	2.511474	1.192894	-5.541874
C	1.187131	1.660816	-4.896698
C	-2.349874	4.243345	-3.219068
C	-3.130207	5.499738	-2.769337
C	-3.250803	6.351143	-4.052648
C	-3.069985	5.315487	-5.155506
H	0.471883	1.350020	-7.511876
H	0.873437	3.081120	-7.802692
H	2.919603	1.217671	-7.717786
H	3.123869	2.791665	-6.911579
H	2.517504	0.099362	-5.642187
H	3.384396	1.477341	-4.939965
H	1.313287	2.195999	-3.944866
H	0.497168	0.818551	-4.726680
H	-1.385326	4.111100	-2.710009
H	-2.938818	3.322408	-3.083726
H	-4.125119	5.217460	-2.396746
H	-2.614475	6.039604	-1.964260
H	-4.211170	6.878848	-4.123290
H	-2.442863	7.095267	-4.108651
H	-2.683239	5.707157	-6.104709
H	-4.006756	4.754827	-5.335386
H	0.098370	6.205047	-2.608330
O	1.019471	5.104140	-4.108187
C	2.400668	5.156906	-4.575984
C	2.818066	6.622084	-4.494547
C	1.974005	7.133538	-3.318144
C	0.659329	6.393399	-3.534583
H	3.012088	4.517491	-3.916211
H	2.428252	4.753419	-5.597949
H	2.539657	7.145502	-5.421520
H	3.898639	6.738111	-4.335997
H	1.841707	8.223510	-3.322613
H	2.428812	6.840859	-2.359021
H	0.012129	6.925930	-4.252670
H	-2.371066	2.106942	-9.253636
O	-2.080053	0.923320	-7.591599
C	-2.541405	1.000054	-6.226496
C	-3.897873	1.730704	-6.282484
C	-3.856634	2.481786	-7.640259
C	-2.439497	2.197587	-8.161165
H	-2.599772	-0.025026	-5.836341
H	-1.817995	1.572226	-5.621237
H	-4.002646	2.412174	-5.428515
H	-4.736731	1.021613	-6.250663
H	-4.038873	3.560040	-7.535377
H	-4.610207	2.077244	-8.330367
H	-1.735047	2.981266	-7.822795
N	-1.099892	0.854720	-1.650305
N	-1.283028	1.166819	-2.730238

$TS_{2LiCl[THF]4}$

164

C	3.852156	0.229184	0.154629
C	3.039924	0.297919	1.458668
B	0.164696	0.049790	-0.964796
C	1.487735	0.159002	-0.389130
C	1.597269	-0.158300	1.132064
H	3.013892	1.343955	1.802583
H	3.489655	-0.306015	2.260183
N	2.771481	0.455486	-0.862252
C	4.926978	1.317911	0.094240
H	4.502342	2.312186	0.282126
H	5.688180	1.120269	0.862669
H	5.428223	1.331455	-0.884736
C	0.601255	0.662538	1.968956
H	0.809892	0.536313	3.044270
H	0.668699	1.732314	1.722685
H	-0.430552	0.335608	1.782446
C	1.365853	-1.653083	1.425882
H	0.324621	-1.918736	1.193663
H	2.013824	-2.298440	0.818452
H	1.554874	-1.872836	2.489915
C	4.547268	-1.141379	0.007742
H	5.198049	-1.166789	-0.874959
H	5.177251	-1.327205	0.890756
H	3.822896	-1.961960	-0.073171
C	3.003404	1.114261	-2.110275
C	2.780212	2.515396	-2.213637
C	3.427596	0.376048	-3.244813

C	3.020692	3.146081	-3.440975
C	3.657697	1.057335	-4.450592
C	3.459403	2.431281	-4.554992
H	2.855922	4.222972	-3.530311
H	3.990062	0.495581	-5.327560
H	3.618683	2.940635	-5.507682
C	2.266608	3.356105	-1.053504
H	2.249063	2.713408	-0.162064
C	3.610885	-1.132990	-3.211958
H	3.361829	-1.464047	-2.196400
C	0.822743	3.822614	-1.309624
H	0.156504	2.961566	-1.447411
H	0.450779	4.409868	-0.456077
H	0.765857	4.456314	-2.209139
C	3.173839	4.564328	-0.764265
H	4.222944	4.265643	-0.625304
H	3.143539	5.295832	-1.586754
H	2.839456	5.080631	0.148432
C	2.647228	-1.849546	-4.171507
H	2.736985	-2.940947	-4.058420
H	1.605541	-1.569662	-3.967933
H	2.870392	-1.604296	-5.221547
C	5.062078	-1.535354	-3.529477
H	5.312484	-1.302979	-4.576419
H	5.783999	-1.003094	-2.893092
H	5.205774	-2.616943	-3.382803
B	-1.457739	0.069608	-0.942435
C	-2.492172	-0.899841	-1.491058
C	-2.128282	-2.245425	-2.132313
C	-3.497880	-2.959927	-2.242232
C	-4.576102	-1.866198	-2.262286
H	-3.645840	-3.591281	-1.352673
H	-3.558810	-3.608941	-3.126628
N	-3.839600	-0.748696	-1.549709
C	-4.519356	0.351066	-0.912288
C	-4.858308	0.226053	0.459574
C	-4.773203	1.546155	-1.626361
C	-5.499999	1.305313	1.081841
C	-5.418113	2.593092	-0.951924
C	-5.786933	2.477337	0.385600
H	-5.766861	1.230829	2.138066

H	-5.617970	3.525925	-1.483286
H	-6.285328	3.307431	0.890925
C	-1.466316	-2.035080	-3.510161
H	-1.370579	-3.001244	-4.028862
H	-2.033860	-1.352546	-4.156959
H	-0.464027	-1.611384	-3.374968
C	-1.168995	-3.059887	-1.249742
H	-1.586291	-3.201694	-0.241664
H	-1.002441	-4.051294	-1.701141
H	-0.204724	-2.540481	-1.153870
C	-4.967128	-1.463687	-3.694108
H	-5.415222	-2.332792	-4.196466
H	-5.712868	-0.658108	-3.691299
H	-4.102645	-1.142571	-4.288977
C	-5.848180	-2.283372	-1.521532
H	-6.548645	-1.441906	-1.418804
H	-6.349881	-3.072491	-2.099157
H	-5.628309	-2.684880	-0.525619
C	-4.332191	1.777641	-3.065197
H	-3.874263	0.848939	-3.429397
C	-4.503884	-0.993675	1.302795
H	-4.108101	-1.769023	0.632384
C	-3.386243	-0.661922	2.309056
H	-3.699527	0.132273	3.003361
H	-3.133531	-1.554239	2.902095
H	-2.478647	-0.330736	1.787792
C	-5.727538	-1.570612	2.035143
H	-6.096230	-0.876194	2.805280
H	-6.557877	-1.777371	1.345487
H	-5.456687	-2.509909	2.540530
C	-3.258543	2.877063	-3.162339
H	-3.641178	3.843474	-2.802141
H	-2.367166	2.621495	-2.576271
H	-2.947893	3.005209	-4.210525
C	-5.526929	2.124429	-3.970256
H	-5.961205	3.097067	-3.693948
H	-5.208988	2.191405	-5.020441
H	-6.327263	1.373452	-3.902393
N	-1.885522	1.315548	-0.322159
N	-2.063773	2.294628	0.239302
N	0.283585	0.858451	-3.169926

N	-0.271159	1.234146	-4.057570
Cl	0.424128	2.988691	-10.528562
Li	0.975249	2.473051	-8.380222
O	1.062785	0.534034	-8.083629
C	1.253775	-0.461989	-9.126276
C	1.361274	-1.793102	-8.391526
C	0.392005	-1.584683	-7.218130
C	0.624497	-0.120915	-6.855721
O	-0.190498	3.494166	-7.127293
C	0.218825	3.841776	-5.785451
C	0.613538	5.320426	-5.854318
C	-0.225317	5.870037	-7.041729
C	-0.958944	4.632663	-7.588174
H	0.386566	-0.437382	-9.807617
H	2.152645	-0.186066	-9.695356
H	1.089574	-2.643477	-9.030898
H	2.388063	-1.948293	-8.023966
H	-0.649927	-1.723635	-7.541911
H	0.587206	-2.256301	-6.371598
H	1.418785	0.008461	-6.103422
H	-0.285298	0.379500	-6.505134
H	1.032063	3.166667	-5.498722
H	-0.632281	3.683022	-5.099545
H	0.398230	5.837463	-4.909136
H	1.688804	5.419330	-6.056436
H	-0.937982	6.643292	-6.725899
H	0.426048	6.307871	-7.810189
H	-0.998258	4.568810	-8.682731
H	-1.981274	4.559750	-7.173851
O	2.854004	3.095415	-7.941830
C	3.929987	2.180320	-8.257250
C	4.248549	2.468683	-9.720499
C	4.069742	4.002257	-9.812101
C	3.195345	4.353951	-8.588141
H	3.737014	4.987509	-7.865257
H	4.798530	2.390164	-7.604961
H	3.560650	1.167491	-8.052717
H	3.512301	1.966088	-10.363813
H	5.255890	2.136786	-10.005358
H	3.573864	4.288036	-10.748310
H	5.036544	4.521461	-9.758053
H	2.248637	4.837786	-8.862134
O	-2.673554	0.059280	-7.686073
C	-3.003105	1.141504	-6.801172
C	-3.953315	2.026680	-7.609889
C	-3.383989	1.911987	-9.044946
C	-2.460382	0.672702	-8.980485
H	-2.685489	-0.088167	-9.740107
H	-3.443888	0.706129	-5.894048
H	-2.090650	1.703469	-6.519691
H	-3.979194	3.062125	-7.242221
H	-4.974958	1.621895	-7.562336
H	-2.798838	2.798924	-9.323160
H	-4.186048	1.791125	-9.785005
H	-1.404723	0.978271	-9.083158

Int$_{2LiCl[THF]4}$

164

C	-3.317639	-3.013142	0.813614
C	-4.364452	-2.150856	0.087932
C	-2.181605	-1.089082	-0.104500
C	-1.922333	-2.531196	0.350787
H	-3.470733	-4.084554	0.622238
H	-3.411433	-2.851893	1.898698
N	-3.532698	-0.943345	-0.273773
C	-4.948056	-2.842710	-1.155042
H	-5.623104	-2.165094	-1.695492
H	-4.172830	-3.192387	-1.846762
H	-5.535201	-3.715368	-0.834591
C	-1.406946	-3.358850	-0.847944
H	-2.080744	-3.296847	-1.713447
H	-0.421288	-3.004374	-1.171265
H	-1.316960	-4.417933	-0.558525
C	-0.916723	-2.637350	1.503487
H	-0.790607	-3.692967	1.792333
H	0.061360	-2.232839	1.209726
H	-1.268266	-2.076960	2.381078
C	-5.533649	-1.788189	1.009346
H	-6.222282	-1.081571	0.523417
H	-6.099700	-2.701297	1.243986
H	-5.184930	-1.354496	1.954111
C	-4.118367	0.244769	-0.833898
C	-4.196894	0.389910	-2.243303

C	-4.548050	1.282420	0.032263
C	-4.792164	1.551544	-2.756430
C	-5.141877	2.418193	-0.536132
C	-5.277249	2.551344	-1.916316
H	-4.861137	1.680974	-3.838579
H	-5.486560	3.223586	0.116045
H	-5.736529	3.447935	-2.338545
C	-3.603018	-0.615085	-3.223270
H	-3.236273	-1.469987	-2.642329
C	-4.311451	1.260925	1.538232
H	-3.880793	0.282967	1.794765
C	-2.389707	-0.027862	-3.969142
H	-2.682396	0.835111	-4.585414
H	-1.607347	0.294588	-3.271773
H	-1.958203	-0.789783	-4.636058
C	-4.640046	-1.124900	-4.238442
H	-4.963008	-0.317939	-4.913305
H	-4.201769	-1.920432	-4.860070
H	-5.535608	-1.529926	-3.747552
C	-3.280400	2.328793	1.948842
H	-3.079747	2.265919	3.029276
H	-2.330656	2.195395	1.417357
H	-3.652142	3.341746	1.732792
C	-5.608862	1.454984	2.341769
H	-6.381893	0.728514	2.057309
H	-5.408651	1.342138	3.418025
H	-6.023114	2.463493	2.187512
B	0.619813	-0.212369	-0.369657
B	-1.093683	-0.076732	-0.359193
C	1.664723	0.363264	0.553667
C	1.337975	1.151466	1.830413
C	2.689986	1.181528	2.580850
C	3.800468	1.006299	1.531315
H	2.727463	0.335395	3.284889
H	2.821897	2.104005	3.163247
N	3.026961	0.347416	0.413816
C	3.672538	-0.249590	-0.724592
C	4.007172	-1.627700	-0.686900
C	3.897465	0.523603	-1.892129
C	4.636317	-2.189368	-1.806918
C	4.523954	-0.092106	-2.985163

C	4.904371	-1.431331	-2.944204
H	4.903732	-3.248059	-1.793585
H	4.702584	0.487346	-3.893396
H	5.392736	-1.889605	-3.807076
C	0.874708	2.572129	1.444232
H	0.757448	3.188913	2.349429
H	1.595675	3.069665	0.782957
H	-0.086271	2.544514	0.917976
C	0.262279	0.496926	2.705083
H	0.570987	-0.510600	3.016574
H	0.098421	1.105610	3.608837
H	-0.688048	0.410394	2.160891
C	4.422771	2.345054	1.100559
H	3.673157	3.067637	0.757512
H	4.944466	2.788900	1.960535
H	5.160170	2.194645	0.300950
C	4.932543	0.113071	2.048171
H	5.657975	-0.114102	1.253759
H	5.465417	0.641825	2.851417
H	4.548229	-0.827564	2.460498
C	3.439833	1.969162	-2.045549
H	3.042765	2.298688	-1.077600
C	3.646186	-2.546558	0.474095
H	3.187066	-1.931060	1.260840
C	2.595913	-3.587172	0.042900
H	2.300874	-4.206538	0.903515
H	1.695498	-3.100796	-0.351292
H	2.994827	-4.255378	-0.735708
C	4.877030	-3.259912	1.060372
H	4.590891	-3.838169	1.952123
H	5.306991	-3.965653	0.333211
H	5.665870	-2.551919	1.347216
C	2.293658	2.097286	-3.065957
H	2.602670	1.743558	-4.061155
H	1.412296	1.523716	-2.750702
H	1.996155	3.152129	-3.161939
C	4.602337	2.896861	-2.439685
H	4.964984	2.671180	-3.453835
H	4.274207	3.947173	-2.436985
H	5.454959	2.806027	-1.752103
N	1.151781	-1.349642	-2.617948

N	1.007061	-0.827436	-1.607163
N	-1.435168	1.216230	-0.874425
N	-1.552427	2.286065	-1.269146
Li	-0.561210	6.748805	-1.999758
Cl	-1.392011	8.859298	-2.156702
C	2.035588	7.870631	-0.874100
O	1.281734	6.716497	-1.316692
C	2.502369	7.490895	0.530464
C	2.727036	5.959623	0.433008
C	2.027491	5.559649	-0.885373
C	-0.784295	4.344397	-3.873893
O	-0.869545	5.779574	-3.704958
C	-2.159092	3.889608	-4.402998
C	-2.874258	5.206964	-4.798560
C	-1.758221	6.251792	-4.743470
H	2.884515	8.026731	-1.562400
H	1.356231	8.731204	-0.921526
H	3.410868	8.032966	0.824695
H	1.716064	7.721478	1.263578
H	3.795750	5.709779	0.392043
H	2.295206	5.437554	1.297330
H	1.310703	4.732785	-0.784535
H	2.766572	5.300887	-1.662230
H	-0.532940	3.912867	-2.897648
H	0.020753	4.115339	-4.594453
H	-2.039062	3.217101	-5.263023
H	-2.712965	3.346471	-3.627055
H	-3.337448	5.156934	-5.793139
H	-3.660799	5.455331	-4.071833
H	-2.075625	7.262298	-4.455898
H	-1.207401	6.296079	-5.701346
C	-1.281172	5.818447	0.768386
O	-1.465282	5.606616	-0.646295
C	-2.439157	6.728660	1.164256
C	-3.600600	6.182326	0.304500
C	-2.894927	5.444343	-0.855182
H	-3.126365	4.368981	-0.870082
H	-1.322819	4.848450	1.296474
H	-0.286554	6.261214	0.907876
H	-2.202897	7.762144	0.873149
H	-2.652537	6.699236	2.241409
H	-4.248163	6.989797	-0.060852
H	-4.224975	5.485158	0.881816
H	-3.122514	5.874114	-1.839548
C	2.628946	6.187371	-4.590828
O	3.124187	7.455580	-4.127724
C	1.968934	6.479751	-5.947226
C	1.528805	7.964036	-5.820888
C	2.069178	8.389641	-4.441653
H	2.504225	9.397749	-4.422994
H	3.478844	5.492965	-4.643323
H	1.881392	5.798105	-3.875119
H	1.125713	5.801949	-6.140348
H	2.692826	6.356247	-6.764380
H	0.437726	8.083000	-5.865996
H	1.966494	8.570326	-6.625474
H	1.271584	8.330323	-3.678372

Int$_{3LiCl[THF]4}$

164

Cl	-1.109355	-2.092461	6.524419
Li	-0.367850	-1.241533	4.548010
O	-1.032160	0.544107	4.052755
C	-1.569322	1.495057	5.009714
C	-1.663240	2.797621	4.228702
C	-2.136131	2.303220	2.850711
C	-1.477799	0.920593	2.714409
O	-0.746428	-2.397568	3.005853
C	0.026925	-2.353055	1.782887
C	0.632072	-3.746585	1.668333
C	-0.512926	-4.643876	2.178082
C	-1.293961	-3.738149	3.152275
H	-2.562261	1.156931	5.352565
H	-0.881810	1.522876	5.865447
H	-2.358424	3.514027	4.686427
H	-0.671686	3.271238	4.158574
H	-3.232765	2.214679	2.838150
H	-1.842478	2.977459	2.035320
H	-0.592803	0.933429	2.063702
H	-2.171347	0.154292	2.339937
H	0.755779	-1.538027	1.885063
H	-0.634929	-2.123914	0.931278
H	0.939838	-3.989563	0.642638

H	1.513627	-3.834354	2.318878
H	-1.155925	-4.956602	1.342487
H	-0.142827	-5.552496	2.670087
H	-1.177335	-4.012151	4.210128
H	-2.370463	-3.713595	2.916568
O	1.643176	-1.176241	4.765304
C	2.071761	-0.341355	5.882230
C	2.684605	-1.296143	6.897500
C	3.326669	-2.352444	5.986521
C	2.298132	-2.475764	4.864361
H	2.747626	-2.708612	3.886656
H	2.807572	0.393148	5.511448
H	1.187569	0.190049	6.262478
H	1.887934	-1.747176	7.507169
H	3.407224	-0.797144	7.556974
H	3.499791	-3.311596	6.492379
H	4.288490	-1.989281	5.592901
H	1.528173	-3.229300	5.097465
O	-4.791804	0.034299	5.346953
C	-4.472796	-0.806670	4.225719
C	-5.090289	-2.159557	4.571190
C	-4.802378	-2.263667	6.083710
C	-4.649106	-0.791969	6.534168
H	-5.421040	-0.465189	7.245680
H	-4.894613	-0.329521	3.329454
H	-3.375463	-0.898806	4.109376
H	-4.646982	-2.988718	4.000655
H	-6.171717	-2.140136	4.370136
H	-3.855938	-2.794066	6.257653
H	-5.606292	-2.783807	6.621728
H	-3.649988	-0.632753	6.974928
C	-3.801275	-0.265461	-5.623646
C	-4.347465	-1.041239	-4.410390
C	-2.107430	-0.268573	-3.910627
C	-2.274832	-0.086702	-5.417415
H	-4.277755	0.725816	-5.659962
H	-4.031703	-0.779301	-6.567284
N	-3.257143	-0.782516	-3.395769
C	-5.701785	-0.499932	-3.943393
H	-6.020262	-0.975723	-3.004267
H	-5.676718	0.586930	-3.801864
H	-6.459064	-0.725446	-4.707702
C	-1.837193	1.308908	-5.896580
H	-0.756710	1.446907	-5.761112
H	-2.083061	1.427759	-6.963978
H	-2.360767	2.094647	-5.331833
C	-1.451753	-1.161677	-6.160730
H	-1.615978	-1.076316	-7.246570
H	-0.381374	-1.025195	-5.952458
H	-1.732403	-2.178246	-5.850865
C	-4.508925	-2.541941	-4.699601
H	-4.819485	-3.083667	-3.795867
H	-5.294386	-2.676308	-5.457251
H	-3.587389	-2.995053	-5.085159
C	-3.386665	-1.114191	-2.002786
C	-3.890782	-0.136448	-1.107857
C	-2.959655	-2.384619	-1.538093
C	-4.046281	-0.491698	0.239711
C	-3.147388	-2.686921	-0.181783
C	-3.697606	-1.759317	0.699193
H	-4.442974	0.245322	0.941534
H	-2.846019	-3.667273	0.192032
H	-3.829372	-2.019412	1.750826
C	-4.210303	1.292049	-1.533173
H	-4.100135	1.348959	-2.624506
C	-2.249562	-3.403564	-2.420497
H	-2.250808	-3.017282	-3.446933
C	-3.206424	2.289501	-0.925590
H	-3.440125	3.311101	-1.262324
H	-3.255607	2.278379	0.173003
H	-2.175931	2.058695	-1.223429
C	-5.646622	1.702705	-1.164724
H	-5.873262	2.696799	-1.578641
H	-6.388911	0.991121	-1.550903
H	-5.775087	1.763188	-0.073262
C	-0.776198	-3.566511	-2.002201
H	-0.241635	-2.608227	-2.023235
H	-0.701777	-3.982693	-0.986377
H	-0.265379	-4.260759	-2.686453
C	-2.945505	-4.775798	-2.412295
H	-4.011184	-4.700423	-2.667776
H	-2.464959	-5.447544	-3.139284

H	-2.871614	-5.252917	-1.422880
B	2.467155	1.283494	-2.547920
B	-0.843546	0.058857	-3.168745
C	3.723963	1.915714	-2.151664
C	4.810347	2.325389	-3.146808
C	5.694732	3.270706	-2.295228
C	5.470774	2.907712	-0.810209
H	5.370865	4.309599	-2.461007
H	6.756513	3.205214	-2.570738
N	4.136766	2.216557	-0.881836
C	3.344846	1.927895	0.280411
C	2.391216	2.877918	0.722921
C	3.515228	0.694604	0.955687
C	1.719052	2.626438	1.926124
C	2.815331	0.494125	2.153331
C	1.947502	1.461284	2.653536
H	0.987520	3.351825	2.289973
H	2.928300	-0.440046	2.705496
H	1.403577	1.274502	3.580484
C	5.582154	1.076703	-3.626483
H	6.422145	1.374562	-4.273981
H	5.985295	0.495697	-2.785342
H	4.915791	0.416178	-4.199811
C	4.224524	3.051464	-4.366884
H	3.548862	2.388254	-4.927313
H	3.653549	3.938827	-4.056869
H	5.030644	3.372863	-5.044892
C	6.564977	1.980596	-0.255028
H	7.521404	2.523121	-0.230602
H	6.326796	1.670818	0.772144
H	6.700435	1.081883	-0.869308
C	5.408071	4.158303	0.074370
H	5.133867	3.902781	1.108376
H	6.398016	4.636318	0.093201
H	4.684901	4.886726	-0.312398
C	4.326584	-0.452174	0.370226
H	4.895675	-0.058710	-0.481838
C	1.996365	4.094919	-0.101169
H	2.704223	4.180727	-0.938099
C	0.597427	3.875879	-0.711188
H	-0.165437	3.783870	0.078179
H	0.322670	4.725780	-1.354722
H	0.572731	2.957244	-1.313348
C	2.033706	5.404176	0.702975
H	3.011316	5.562518	1.179750
H	1.829290	6.260236	0.042663
H	1.269453	5.411255	1.495741
C	3.375774	-1.530486	-0.183380
H	2.810413	-2.007038	0.631385
H	2.653445	-1.094482	-0.888696
H	3.946481	-2.314754	-0.704163
C	5.317473	-1.067130	1.369032
H	4.794228	-1.553791	2.206761
H	5.928968	-1.836008	0.873141
H	5.994722	-0.311562	1.792133
N	0.398013	0.509022	-3.805464
N	1.427759	0.880334	-3.201235
N	-0.679998	-0.026768	-1.731735
N	-0.120359	0.080104	-0.734381

TS$_{3LiCl[THF]4}$

164

C	-4.643668	2.286055	-2.717077
C	-5.007679	0.905698	-2.146959
C	-2.675171	1.353750	-1.682964
C	-3.098991	2.397667	-2.721666
H	-5.058661	3.064758	-2.058907
H	-5.070571	2.435952	-3.718435
N	-3.752845	0.577644	-1.368227
C	-6.242521	0.971804	-1.245179
H	-6.427998	0.011513	-0.742516
H	-6.143338	1.757343	-0.486321
H	-7.121923	1.205882	-1.862021
C	-2.683611	3.827397	-2.327742
H	-1.592338	3.941501	-2.337504
H	-3.119833	4.546832	-3.039032
H	-3.050166	4.074869	-1.320223
C	-2.494513	2.055512	-4.100584
H	-2.863573	2.764258	-4.858913
H	-1.398555	2.123805	-4.061959
H	-2.761620	1.039531	-4.421911
C	-5.272243	-0.130936	-3.250012
H	-5.485140	-1.116069	-2.815232

H	-6.154204	0.178773	-3.829185
H	-4.426642	-0.227998	-3.942484
C	-3.740368	-0.489329	-0.405372
C	-4.074408	-0.193881	0.940061
C	-3.444484	-1.815746	-0.809121
C	-4.235327	-1.262210	1.833442
C	-3.636431	-2.847867	0.121724
C	-4.056047	-2.581040	1.422731
H	-4.510236	-1.056050	2.869047
H	-3.436634	-3.879409	-0.175924
H	-4.215868	-3.402336	2.124779
C	-4.215305	1.231272	1.459893
H	-4.205802	1.907610	0.593631
C	-2.854077	-2.160657	-2.170244
H	-2.875583	-1.251764	-2.785851
C	-3.004538	1.599147	2.338261
H	-3.090189	2.637195	2.694323
H	-2.950641	0.943387	3.220233
H	-2.063702	1.501564	1.780140
C	-5.522153	1.460921	2.237555
H	-5.622757	2.524639	2.500450
H	-6.405044	1.164855	1.654757
H	-5.537852	0.888847	3.176761
C	-1.377210	-2.573337	-2.015762
H	-0.792909	-1.790586	-1.518977
H	-1.285940	-3.493418	-1.418193
H	-0.930173	-2.763860	-3.003704
C	-3.627710	-3.269273	-2.903444
H	-4.699040	-3.042044	-2.990718
H	-3.222935	-3.402659	-3.918070
H	-3.531922	-4.233102	-2.380869
B	2.024497	1.392296	-0.661765
B	-1.269945	1.255262	-1.188341
C	3.485685	1.374561	-0.553513
C	4.346949	2.332654	-1.390521
C	5.725053	2.226705	-0.690500
C	5.774167	0.873192	0.045908
H	5.813904	3.038228	0.047215
H	6.556617	2.330979	-1.401484
N	4.305913	0.574067	0.197291
C	3.825989	-0.486399	1.038037

C	3.519316	-0.207176	2.391981
C	3.661676	-1.789480	0.506921
C	3.169412	-1.275826	3.229007
C	3.318069	-2.825653	1.387617
C	3.099128	-2.579205	2.741309
H	2.912008	-1.080263	4.271884
H	3.198379	-3.839966	1.000682
H	2.833383	-3.399983	3.411007
C	4.397511	1.845843	-2.855101
H	5.093837	2.468374	-3.439347
H	4.727464	0.800502	-2.928279
H	3.399703	1.913844	-3.311609
C	3.837804	3.781192	-1.363443
H	2.852455	3.860845	-1.843246
H	3.748284	4.145596	-0.329595
H	4.538112	4.436771	-1.904901
C	6.501721	-0.214066	-0.763109
H	7.563981	0.055027	-0.855771
H	6.443946	-1.184264	-0.249996
H	6.092284	-0.327357	-1.774673
C	6.470562	0.998370	1.405379
H	6.399847	0.062578	1.978170
H	7.536137	1.217975	1.245707
H	6.041757	1.813362	2.001205
C	3.740367	-2.081583	-0.985347
H	4.149157	-1.190433	-1.478264
C	3.466490	1.211323	2.944083
H	3.855465	1.887743	2.170042
C	2.004104	1.616101	3.211928
H	1.576453	0.994963	4.014718
H	1.950799	2.667900	3.532637
H	1.385226	1.488515	2.312896
C	4.304991	1.390297	4.219635
H	5.349145	1.082383	4.072187
H	4.296911	2.444416	4.535521
H	3.893748	0.795442	5.048798
C	2.323338	-2.295092	-1.549296
H	1.845282	-3.176674	-1.094077
H	1.687005	-1.423480	-1.346162
H	2.363541	-2.451703	-2.638285
C	4.642182	-3.278231	-1.324460

H	4.226720	-4.218719	-0.931957
H	4.729981	-3.387423	-2.415972
H	5.653857	-3.159317	-0.911008
N	-0.145372	2.097503	-1.677156
N	1.024031	1.964248	-1.341980
N	-0.652527	0.374419	-0.207669
N	0.392962	0.140418	0.270949
Cl	-1.212242	-2.958857	7.718159
Li	-0.471478	-1.461746	6.142117
O	-1.035868	0.390593	6.510812
C	-1.439864	0.832128	7.833751
C	-0.826558	2.235500	7.994752
C	-0.532271	2.691035	6.537597
C	-1.127585	1.567149	5.683172
O	-0.944790	-2.047038	4.339201
C	-0.484802	-1.455730	3.085857
C	-0.423057	-2.592275	2.054124
C	-0.557745	-3.867001	2.905512
C	-1.416499	-3.389412	4.068729
H	-2.542352	0.859224	7.869169
H	-1.078723	0.081465	8.547748
H	-1.526293	2.910417	8.505086
H	0.097473	2.200966	8.587406
H	-0.981611	3.663667	6.297244
H	0.550488	2.767697	6.364129
H	-0.586200	1.367363	4.749661
H	-2.189573	1.762967	5.447038
H	0.500210	-1.011460	3.278928
H	-1.187586	-0.665407	2.786373
H	-1.260149	-2.510585	1.349551
H	0.509854	-2.559514	1.479317
H	-1.022643	-4.697498	2.356172
H	0.424307	-4.200233	3.274497
H	-1.314552	-3.959281	5.001781
H	-2.483706	-3.353256	3.786358
O	1.565206	-1.378080	6.264888
C	2.152445	-0.507289	7.259610
C	2.145819	-1.335028	8.542601
C	2.404666	-2.773698	8.032326
C	2.140396	-2.690764	6.514361
H	3.075332	-2.782901	5.935338
H	3.183267	-0.245207	6.956064
H	1.545430	0.405756	7.291460
H	1.154212	-1.280298	9.013479
H	2.900656	-0.991633	9.262658
H	1.715633	-3.482226	8.508157
H	3.436751	-3.094909	8.228538
H	1.413974	-3.431370	6.155649
O	-4.683771	-0.249493	7.088525
C	-4.311099	-0.693982	5.771872
C	-5.020246	-2.037295	5.608062
C	-4.879083	-2.651828	7.017881
C	-4.651487	-1.426904	7.936030
H	-5.432357	-1.294353	8.698621
H	-4.634634	0.083323	5.064999
H	-3.214722	-0.815415	5.695958
H	-4.563444	-2.665521	4.830588
H	-6.077153	-1.876131	5.347576
H	-3.998690	-3.307505	7.066300
H	-5.768191	-3.229743	7.304301
H	-3.665903	-1.502531	8.426584

Pdt$_{LiCl[THF]4}$

164

Li	-0.969642	3.948096	-5.003659
Cl	-1.043325	5.212647	-6.890630
O	-0.548162	2.068980	-5.333953
O	-2.502045	4.076904	-3.770510
C	0.022698	1.485030	-6.530457
C	1.158232	0.577913	-6.028195
C	0.772673	0.270493	-4.553760
C	-0.561217	1.006594	-4.362717
C	-2.270438	3.907368	-2.335183
C	-3.016879	5.050172	-1.630181
C	-3.381713	6.017425	-2.771705
C	-3.537975	5.071707	-3.955727
H	-0.765403	0.908926	-7.044831
H	0.341664	2.315616	-7.172875
H	1.233825	-0.331964	-6.637952
H	2.125617	1.096141	-6.074912
H	0.668374	-0.805217	-4.359279
H	1.530860	0.665354	-3.864125
H	-0.688214	1.463468	-3.373742

H	-1.419845	0.339716	-4.566102
H	-1.183593	3.944582	-2.185941
H	-2.643812	2.923032	-2.022588
H	-3.918766	4.657877	-1.143730
H	-2.400377	5.521151	-0.853375
H	-4.297116	6.590193	-2.568606
H	-2.564686	6.728169	-2.965538
H	-3.370301	5.531127	-4.940128
H	-4.528199	4.580820	-3.945093
H	0.089672	6.221090	-2.626325
O	0.581068	4.664017	-3.920765
C	1.887789	4.401232	-4.506075
C	2.417423	5.755512	-4.961962
C	1.841510	6.692600	-3.890029
C	0.455702	6.096657	-3.655515
H	2.537335	3.948078	-3.735446
H	1.752139	3.679632	-5.323516
H	2.001845	6.003899	-5.949854
H	3.514028	5.778522	-5.016099
H	1.787704	7.739532	-4.216642
H	2.447509	6.646687	-2.971687
H	-0.283199	6.506353	-4.363844
H	-3.417279	1.914908	-9.241717
O	-3.289719	1.092026	-7.353454
C	-3.970687	1.495790	-6.151092
C	-5.023173	2.495739	-6.623410
C	-4.246407	3.280459	-7.696331
C	-3.211554	2.259542	-8.217132
H	-4.383936	0.588116	-5.687570
H	-3.263689	1.974728	-5.446103
H	-5.395670	3.141793	-5.815752
H	-5.879784	1.961768	-7.062082
H	-3.718548	4.135626	-7.249937
H	-4.899571	3.656164	-8.495531
H	-2.196033	2.687599	-8.175883
C	-5.143495	1.781756	-2.636972
C	-5.866110	2.433469	-1.640619
C	-5.825530	1.996018	-0.310349
C	-5.035777	0.859465	-0.004398
C	-4.247244	0.223987	-0.991098
C	-4.333401	0.699010	-2.308390

N	-5.048736	0.374159	1.354347
C	-4.271162	0.889087	2.337007
C	-4.740594	0.356743	3.692086
C	-6.069336	-0.359546	3.336114
C	-6.027184	-0.680908	1.832229
B	-3.083916	1.802522	2.144521
N	-2.664408	2.623238	0.961687
N	-1.432717	2.795301	0.802137
B	-0.367879	2.621414	1.753940
N	-0.972662	2.497476	3.125030
N	-2.113440	1.985339	3.193293
C	-3.698082	-0.602969	4.302962
C	-5.006710	1.504189	4.686047
C	-5.490489	-2.095545	1.559615
C	-7.395361	-0.540699	1.163725
C	-6.580087	2.787894	0.750058
C	-8.048104	3.044442	0.371568
C	-3.262743	-0.896898	-0.684654
C	-3.548748	-2.162088	-1.511536
C	1.081153	2.577632	1.328078
C	1.602515	3.101665	-0.007191
C	3.140341	3.010868	0.158785
C	3.422772	2.000222	1.285581
N	2.101171	2.030190	2.027896
C	3.724298	0.592594	0.749208
C	4.590583	2.436477	2.172781
C	1.082586	2.216884	-1.158675
C	1.176393	4.560181	-0.252865
C	1.940236	1.479284	3.353378
C	1.533395	0.134010	3.520882
C	1.430774	-0.362383	4.829184
C	1.693766	0.442344	5.934779
C	2.034916	1.780595	5.749325
C	2.151451	2.329925	4.465575
C	1.126589	-0.777097	2.368655
C	1.949247	-2.077383	2.334290
C	2.406752	3.825563	4.327726
C	3.652439	4.289850	5.100091
C	-0.374368	-1.112598	2.438310
C	1.166482	4.622803	4.774572
C	-1.814588	-0.432432	-0.921027

C	-5.860758	4.117677	1.043271
H	3.638691	2.721306	-0.777049
H	3.532176	3.995889	0.453635
H	3.869871	-0.117910	1.573525
H	2.927058	0.215877	0.096217
H	4.655331	0.626550	0.165710
H	-0.014262	2.226123	-1.168384
H	1.443300	2.606747	-2.121996
H	1.412771	1.174176	-1.056216
H	1.657768	4.938836	-1.167594
H	0.088070	4.631138	-0.372149
H	1.474837	5.202540	0.589163
H	4.694443	1.781606	3.049451
H	5.520365	2.372633	1.589470
H	4.477359	3.472153	2.514410
H	1.119730	-1.398136	4.982215
H	2.195751	2.419409	6.620238
H	1.609737	0.032496	6.943775
H	1.293752	-0.234499	1.428582
H	2.562287	4.044977	3.262069
H	-0.649804	-1.782354	1.610000
H	-0.621330	-1.624414	3.381151
H	-0.997932	-0.212522	2.371058
H	1.706568	-2.655328	1.429686
H	3.030748	-1.883876	2.336634
H	1.720902	-2.711069	3.204350
H	0.268844	4.281631	4.240014
H	0.987832	4.492086	5.853528
H	1.316802	5.696459	4.583084
H	4.545585	3.711990	4.823400
H	3.851585	5.352153	4.892713
H	3.509476	4.187366	6.186417
H	-6.911103	0.321606	3.531890
H	-6.224672	-1.263965	3.940012
H	-6.470726	3.305531	-1.899283
H	-3.735237	0.222431	-3.088188
H	-5.189455	2.137235	-3.667132
H	-4.081019	-1.010653	5.251339
H	-3.471507	-1.443537	3.633364
H	-2.760600	-0.066436	4.500911
H	-4.069303	2.005580	4.958994

H	-5.685762	2.252707	4.250159
H	-5.475988	1.103024	5.598600
H	-6.158722	-2.827313	2.036018
H	-5.468200	-2.311333	0.484459
H	-4.481439	-2.239826	1.967443
H	-7.323190	-0.654496	0.072890
H	-8.057391	-1.331365	1.544711
H	-7.861588	0.426326	1.387587
H	-3.354309	-1.146431	0.380943
H	-6.568308	2.203409	1.680268
H	-5.914426	4.786623	0.169759
H	-6.340559	4.630408	1.890974
H	-4.800283	3.950931	1.277522
H	-8.577634	2.113157	0.123872
H	-8.573579	3.526196	1.209649
H	-8.127782	3.715700	-0.496701
H	-1.660336	-0.156195	-1.974304
H	-1.559848	0.435875	-0.301116
H	-1.110248	-1.243627	-0.683551
H	-3.356016	-1.986427	-2.581116
H	-2.894285	-2.985266	-1.187451
H	-4.592183	-2.493857	-1.414275
